# Perioperative myeloid cell remodeling shapes CAR-T cell efficacy in glioblastoma

**DOI:** 10.1038/s41467-026-76206-y

**Published:** 2026-07-31

**Authors:** Martin Pedard, Luis Castillo Cantero, Ali Ghasemi, Eliana Marinari, Caterina Mollica, Suzel Davanture, Julie Pernot, Valérie Widmer, Doron Merkler, Kristof Egervari, Karl Schaller, Philippe Bijlenga, Andrea Bartoli, Shahan Momjian, Mikael J. Pittet, Valérie Dutoit, Denis Migliorini

**Affiliations:** 1 Brain Tumor and Immune Cell Engineering Laboratory, Center for Translational Research in Onco-Hematology, Faculty of Medicine, University of Geneva, Geneva, Switzerland; 2 Agora Cancer Research Center, Lausanne, Switzerland; 3 Swiss Cancer Center Léman, Lausanne and Geneva, Switzerland; 4 Division of Neurosurgery, Department of Clinical Neurosciences, Geneva University Hospitals, Geneva, Switzerland; 5 Division of Clinical Pathology, Department of Diagnostics, Geneva University Hospitals Geneva, Geneva, Switzerland; 6 Department of Pathology and Immunology, and Center for Translational Research in Onco-Hematology, Faculty of Medicine, University of Geneva, Geneva, Switzerland; 7 Service of Oncology, Geneva University Hospital, Geneva, Switzerland

**Keywords:** CNS cancer, Cancer microenvironment, Cancer immunotherapy

## Abstract

Glioblastoma (GBM) is characterized by a profoundly immunosuppressive tumor microenvironment (TME) that constrains the efficacy of chimeric antigen receptor (CAR)-T cell therapy. Here, we show that surgical resection in both male mice and human GBM ex vivo induces a rapid and sustained remodeling of the TME, marked by upregulation of TREM2 in myeloid cells followed by emergence of T cell exhaustion-like phenotypes. In male mice, targeting TREM2 reshapes the perioperative TME and potentiates tumor antigen-specific CAR-T cell responses, improving intratumoral persistence, proliferation, and effector differentiation, and resulting in enhanced survival. In parallel, we identify the timing of CAR-T cell administration as a critical determinant of therapeutic outcome, with neoadjuvant outperforming adjuvant treatment by preserving CAR-T cell effector function in mice. These findings establish perioperative myeloid cell remodeling and treatment timing as key determinants of CAR-T cell efficacy in GBM.

## Abstract (web summary)

Glioblastoma (GBM) is characterized by an immunosuppressive tumor microenvironment (TME). Here the authors report that surgical resection induces TREM2 associated immunosuppressive remodeling of the TME and test CAR-T based strategies to reshape the perioperative GBM TME.

## Introduction

Glioblastoma (GBM) is the most frequent and aggressive malignant primary brain tumor in adults, characterized by rapid progression and inevitable recurrence despite multimodal treatment. Standard-of-care therapy includes maximal surgical resection followed by radiotherapy and chemotherapy with temozolomide; yet the median overall survival remains below 15 months, with less than 5% of patients surviving beyond five years^1^. A growing body of evidence indicates that this poor prognosis stems not only from tumor-intrinsic resistance mechanisms but also from the profound immunosuppressive features of the GBM tumor microenvironment (TME), which severely limit the efficacy of immunotherapy approaches^2,3^.

In this context, chimeric antigen receptor (CAR)-T cell therapies have revolutionized the treatment of hematologic malignancies but have shown limited efficacy in solid tumors, including GBM^4,5^. Several major hurdles hinder CAR-T cell activity in the brain: reduced persistence and expansion, and rapid functional exhaustion induced in part by the immunosuppressive microenvironment^6,7^. The GBM TME is dominated by a heterogeneous myeloid cell compartment comprising brain-resident microglia, monocytes, blood-borne monocyte-derived macrophages, and potentially border-associated macrophages, which can represent up to 40–50% of the tumor mass^8,9^. This myeloid cell infiltrate contributes to the suppression of T cell function through cytokine release, expression of inhibitory ligands, and metabolic dysregulation^10^. In solid tumors^11^, including GBM^12^, myeloid cell-targeting strategies have shown limited or transient efficacy, highlighting the need for novel therapeutic modalities.

Recent studies suggest that surgical resection, while essential for cytoreduction and diagnostic purposes, may paradoxically exacerbate local immunosuppression or stimulate tumor cell proliferation. More precisely, surgical injury reshapes the TME through a wound-healing immune response that recruits pro-tumoral tumor-associated macrophages (TAM) into the resection site^13,14^. These TAM secrete pro-regenerative factors that drive GBM proliferation and enrich for stem-like cells in the remaining tumor population^13^. In parallel, the early post-surgical TME becomes immunosuppressive, highlighted by upregulation of immune checkpoint molecules in T cells^15^. Collectively, these immediate post-surgery immune alterations create fertile ground for residual tumor outgrowth and may ultimately undermine the efficacy of subsequent adjuvant therapies. However, the perioperative window also represents a therapeutic opportunity, as interventions applied in the immediate pre or postoperative phase may reshape the TME and enhance therapeutic response. In this context, local immunomodulation of the resection cavity has recently shown encouraging results. For example, post-resection delivery of a TLR7/8 agonist from a biodegradable scaffold achieved immune-mediated GBM clearance and protection against rechallenge in mice^16^. More recently, implant-mediated controlled release of small-molecule modulators of immunosuppressive myeloid cells into the resection cavity prevented postoperative GBM recurrence and enhanced CD8⁺ T cell infiltration in murine models^17^.

To circumvent the limitations imposed by the TME on CAR-T cell efficacy, multiple combinatorial strategies have emerged. These include the use of checkpoint inhibitors targeting the PD1/PD-L1 or Tim3 axes^18^, endogenous delivery of cytokine such as IL-12 and IL-15^19,20^, metabolic modulators^21^, cytokine-armed dendritic cells (DC)^22^, or PD-L1-targeting CAR-T cells designed to target both tumor and immunosuppressive stromal cells^23^. More generally, recent preclinical and clinical studies have begun to explore the benefits of neoadjuvant immunotherapy, wherein immune-based interventions are administered prior to surgery. This strategy has been shown to enhance tumor-specific T cell activation, increase immune infiltration, and improve survival in both GBM^24–28^ and other solid tumors^29^.

Here, we first characterize the immunological consequences of surgical resection in two clinically relevant murine GBM models (SB28 and CT2A) and in patient-derived organotypic tissue, revealing a surge of TREM2⁺ myeloid cells and early upregulation of PD1/Tim3/PD-L1 as dominant features of the post-resection TME. Building on these observations, we tested multiple strategies to reshape the perioperative GBM TME, including TREM2 CAR-T cells alone, in combination with GD2-targeting CAR-T cells, and with anti-PD1/Tim3 or anti-PD-L1 checkpoint inhibitors.

## Results

### GBM resection induces TREM2-associated remodeling of the TME

To evaluate the impact of resection on the GBM TME, we first characterized the composition of the immune landscape of two murine GBM models seven days after tumor implantation (Supplementary Fig. 1a). We used two well characterized murine GBM models, SB28^30,31^ and CT2A^15^, known to mimic the poor immunogenicity of human GBM and its resistance to immunotherapies. SB28 tumors displayed a marked enrichment of TAM (CD45^high^F4/80⁺CD11b⁺, defined as including both activated microglia and monocyte-derived macrophages; see Methods) compared to CT2A (Supplementary Fig. 1b, c) and elevated TREM2 expression within myeloid cell subsets (Supplementary Fig. 1d, e), including in CD206^-^MHCII^+^ microglia, CD206^+^ TAM and CD206^-^MHCII^+^ TAM. This core difference underscores the inherent variability in TME architecture across GBM models, with potential implications for immunotherapeutic responsiveness, including therapy with CAR-T cells. To complement these flow cytometry observations, we analyzed TREM2 expression across myeloid cell subsets using publicly available single cell RNA sequencing (scRNAseq) datasets from SB28 and CT2A. Consistent with flow cytometry data, TREM2 transcripts were detected across multiple myeloid cell populations, including microglia, TAM, monocytes, and DC, in both models (Supplementary Fig. 2a, b).

**Fig. 1:**
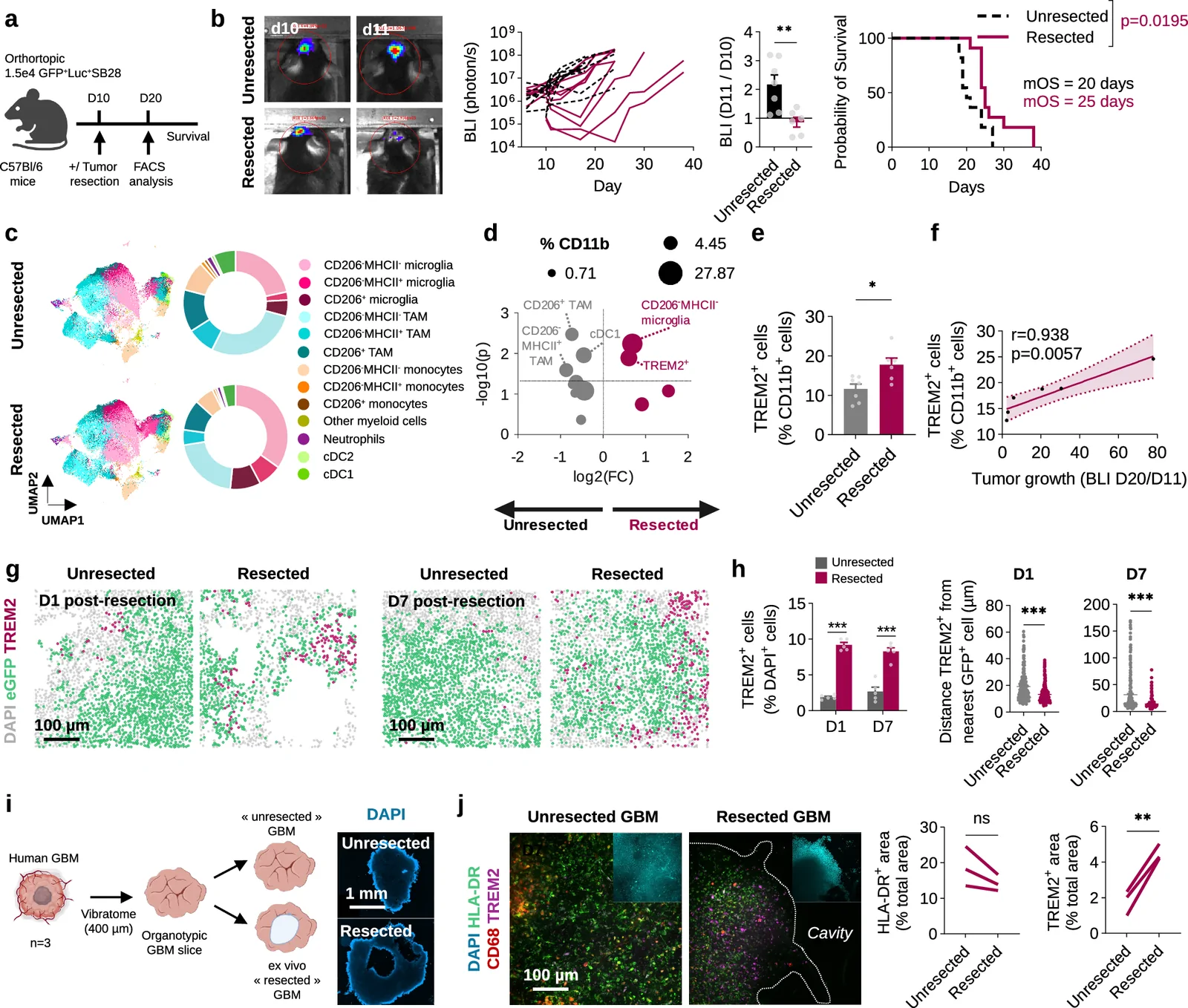
Tumor resection induces a dynamic and TREM2-driven remodeling of the myeloid cell compartment in murine GBM. **a** Scheme comparing unresected and resected orthotopic SB28 tumors at day 20 by flow cytometry. Created in BioRender. Nicola, C. (2026) https://BioRender.com/6o79xto. **b** Left to right: representative bioluminescence imaging (BLI) at days 9 and 11 post-implantation, longitudinal BLI quantification, comparison of BLI signal between days 10 and 11 (Unpaired two-tailed t-test; *n* = 6 unresected, 7 resected) and Kaplan–Meier survival of unresected and resected mice pooled from two independent experiments (*n* = 11 per group; log-rank test). mOS=median overall survival. **c** Representative UMAP of CD45⁺CD11b⁺ myeloid cell populations and donut charts showing myeloid subset distribution in unresected and resected SB28 tumors (*n* = 6 unresected, 7 resected). **d** Volcano plot of myeloid populations and TREM2⁺ cells among CD11b⁺ myeloid cells between unresected and resected tumors; dot size reflects mean population frequency from one experiment (Unpaired two-tailed t-test, *n* = 6 unresected, 7 resected). **e** Frequency of TREM2⁺ cells among CD11b⁺ myeloid cells in unresected and resected tumors (Unpaired two-tailed t-test, *n* = 6 unresected, 7 resected). **f** Pearson correlation between TREM2⁺ myeloid cell frequency and tumor growth (BLI ratio D20/D11) in the SB28 model (*n* = 7 mice); shaded area, 95% confidence interval. **g** Representative synthetic immunofluorescence images showing TREM2⁺ cells (magenta), GFP⁺ tumor cells (green) and DAPI (gray) in unresected (*n* = 5) and resected (*n* = 5) SB28 tumors, sacrificed at D1 and D7 after resection (scale bar = 100 µm). **h** Quantification of TREM2⁺ cells as a percentage of DAPI⁺ nuclei and violin plots of TREM2⁺ cell distance from the GFP⁺ tumor margin at D1 and D7 from one experiment (Unpaired two-tailed t-test, *n* = 5 per group; dots represent individual TREM2⁺ cells). **i** Scheme of ex vivo resection of human GBM organotypic slices and representative DAPI staining of unresected and resected sections at day 1 (scale bar = 1 mm, Created in BioRender. Nicola, C. (2026) https://BioRender.com/myhirie. **j** Immunofluorescence of human GBM slices stained for DAPI (blue), HLA-DR (green), CD68 (red) and TREM2 (magenta), with quantification of HLA-DR⁺ and TREM2⁺ areas (*n* = 3 patients, paired t-test). Data are shown as mean ± SEM unless indicated. *p < 0.05, **p < 0.01, ***p < 0.001. Source data and exact *p* values are provided as a Source Data file.

To then examine the impact of tumor resection on the GBM TME, SB28 and CT2A tumors were surgically removed by aspiration 10 and 12 days after tumor implantation, respectively (Fig. 1a and Supplementary Fig. 3a). This procedure induced substantial cytoreduction, extending median survival by five and two days in the SB28 and CT2A models, respectively (Fig. 1b and Supplementary Fig. 3b, two independent experiments). To examine the late effects of surgery, we profiled tumors on day 20 using flow cytometry. Across both models, resection was associated with a significant increase in CD206^-^MHCII^-^ microglia and a decrease in cDC1 frequency (Fig. 1c, d and Supplementary Figs. 3c, d, and 4). Model-specific changes were also evident: in the SB28 model, CD206^-^MHCII^+^ and CD206^+^ TAM were reduced (Fig. 1c, d and Supplementary Fig. 4a), whereas CD206^-^MHCII^+^ and CD206^+^ TAM were increased alongside a reduction in CD206^-^MHCII^+^ microglia in the CT2A model (Supplementary Fig. 3c, d and 4b). These data reveal that the immune consequences of tumor resection are both shared and model-specific, potentially reflecting distinct compensatory myeloid cell programs. Given the association of TREM2 with GBM aggressiveness and myeloid cell-mediated immunosuppression^32–34^, we quantified its expression in myeloid cell subsets after surgery. On day 20, resected tumors from both models exhibited marked upregulation of TREM2 (Fig. 1e and Supplementary Fig. 3e) across multiple myeloid cell populations (Supplementary Fig. 5), correlating positively with tumor growth kinetics (Fig. 1f and Supplementary Fig. 3f) and providing a rationale for targeting TREM2 in the perioperative setting. To determine whether TREM2 could be effectively targeted immediately after surgery, we analyzed tumors 24 h post-resection in mice bearing SB28 (Supplementary Fig. 6a) and CT2A (Supplementary Fig. 7a) tumors. In SB28-bearing mice, we observed expansion of cDC2 after resection (Supplementary Fig. 6b, c), whereas CD206^+^ microglia, CD206^-^MHCII^-^ monocytes and cDC1were increased, and neutrophils were decreased in CT2A-bearing mice (Supplementary Fig. 7b, c). Notably, tumors collected from both models exhibited pronounced TREM2 induction in myeloid cells within 24 h (Supplementary Figs. 6d, 7d), identifying this early post-operative window as a strategic target for immunomodulation. Immunohistofluorescence analysis of SB28 tumors at early (D1 post-resection) and late (D7 post-resection) time points in resected or unresected mice (Fig. 1g) showed an increase in TREM2⁺ cells in resected compared to unresected tumors at both time points, confirming these findings. Spatial mapping further showed that TREM2⁺ cells localized predominantly at the tumor periphery in both conditions but were positioned significantly closer to residual tumor cells after resection at both D1 and D7 (Fig. 1h). These results indicate that surgical resection not only increases the frequency of TREM2⁺ myeloid cells but also promotes their recruitment and retention near the residual tumor tissue, reinforcing their potential role in early post-operative immune remodeling. Prior to translating these observations to human tissue, we verified that TREM2 expression was distributed across comparable myeloid cell subsets in human GBM. Analysis of publicly available scRNAseq data from human GBM specimens confirmed TREM2 expression in microglia, TAM, monocytes, and DC populations (Supplementary Fig. 2c), supporting the relevance of our murine findings to the human setting. To then extend these findings to human tissue, we established an ex vivo “resection” model on patient-derived GBM organotypic slices. A circumscribed cavity was created in one region of each slice, leaving a paired unresected region as an internal control (Fig. 1i). Immunofluorescence analysis revealed accumulation of dense TREM2⁺/CD68⁺ cells along the cavity edge, whereas HLA-DR^+^ myeloid cells remained broadly distributed (Fig. 1j). Quantification across independent specimens showed no significant change in HLA-DR⁺ area after resection, but a significant increase in TREM2⁺/CD68⁺ area in resected *versus* unresected regions (Fig. 1j). Thus, results obtained using the human organotypic model confirm those obtained in mice showing that resection induces TREM2⁺ myeloid cells in the resection cavity.

### GBM resection induces late T cell exhaustion-like phenotypes in the TME

**Fig. 2:**
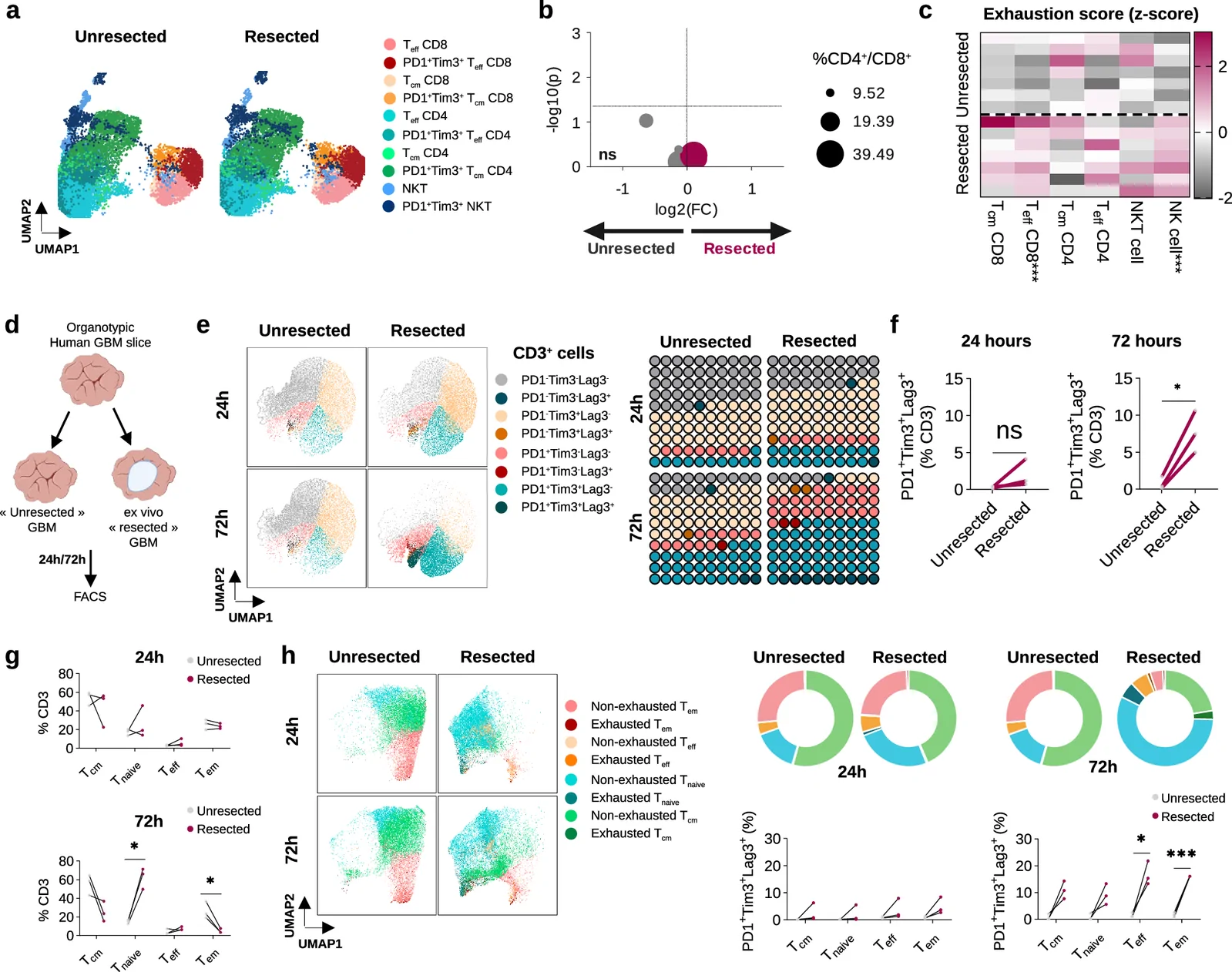
Tumor resection induces late lymphoid cell remodeling in GBM. **a** Representative uniform manifold approximation and projection (UMAP) visualization of CD45⁺CD3^+^ lymphoid cell populations in unresected and resected SB28 tumors (*n* = 7/group). **b** Volcano plot of differential lymphoid cell population abundance between unresected (*n* = 7) and resected (*n* = 7) SB28 tumors; dot size reflects mean population frequency. **c** Heatmaps showing exhaustion-like scores (z-scores; see Methods) for indicated lymphoid cell subsets in unresected and resected tumors in the SB28 model from one experiment (*n* = 7 mice per group, paired two-tailed t-test). **d** Experimental scheme of ex vivo resection of 400-µm-thick human GBM organotypic slices for CD3^+^ T cell analysis. Created in BioRender. Nicola, C. (2026) https://BioRender.com/myhirie. **e** UMAP visualization (left panel) and distribution (right panel) of CD3^+^ lymphoid cell populations in ex vivo unresected (*n* = 3) and resected (*n* = 3) human GBM colored by combinatorial PD-1/Tim-3/Lag-3 expression subsets. **f** Quantification of PD1⁺Tim3⁺Lag3⁺ cells among CD3⁺ T cells at 24 h and 72 h post-resection (*n* = 3 patients/group, paired two-tailed t-test). **g** Frequencies of T central memory (T_cm_), naive (T_naive_), effector (T_eff_) and effector memory (T_em_) cells among CD3⁺ cells at 24 h and 72 h in unresected versus resected slices (paired two-tailed t-test, *n* = 3 patients). **h** UMAP visualization of CD3^+^ lymphoid cell populations in ex vivo unresected and resected human GBM colored by T cell exhaustion-like states (left panel). Proportion of exhausted (PD-1⁺Tim-3⁺Lag-3⁺) cells within each T cell differentiation subset at 24 h and 72 h post-resection (right panel, paired two-tailed t-test, *n* = 3 patients). *p < 0.05, ***p < 0.001. Source data and exact *p* values are provided as a Source Data file.

We analyzed remodeling of the lymphoid cell compartment at the same early and late time points, with particular attention to subsets relevant to immune checkpoint blockade. Noteworthy, seven days after tumor inoculation, total T cell frequencies were comparable between models (Supplementary Fig. 1f). SB28 showed increased levels of CD4^+^ naïve T (*T*_naive_) cells and reduced levels of CD4⁺ effector T (*T*_eff_) cells compared to CT2A, with no change in the proportion of other lymphoid cell subsets (Supplementary Fig. 1g). PD1/Tim3-based scoring indicated higher expression of exhaustion-associated markers in CD4⁺ T_eff_ and NKT cells in SB28 relative to CT2A (Supplementary Fig. 1h). When tumors from mice having undergone tumor resection were analyzed on day 20, CT2A tumors showed increased frequencies of CD4^+^ T_eff_ and NKT cells and decreased frequencies of CD8^+^ T_eff_ cells in tumors isolated from resected *vs* unresected mice (Supplementary Figs. 3g, h, and 8c, d), whereas SB28 tumors displayed no changes in any lymphoid cell population (Fig. 2a, b and Supplementary Fig. 8a, b). However, analysis of the lymphoid cell exhaustion profile revealed that, in both models, CD8^+^ T_eff_ and NK cells exhibited increased exhaustion-like scores post-surgery (Fig. 2c and Supplementary Fig. 3i). In the CT2A model, CD8^+^ central memory T (*T*_cm_) and NKT cells also demonstrated elevated exhaustion-like phenotypes (Supplementary Fig. 3i). When focusing on early changes (24 h after resection), SB28 tumors showed a decrease in CD4^+^ T_eff_ and T_cm_ and NK cell subsets in resected tumors (Supplementary Fig. 9a, c), whereas no differences between resected and unresected CT2A tumors were observed (Supplementary Fig. 9a, d). Except for CD8^+^ T_eff_ cells displaying less exhaustion after resection in the CT2A model, no difference in lymphoid cell exhaustion-associated marker expression was observed between unresected and resected conditions 24 h post-surgery in either tumor model (Supplementary Fig. 9b). We then assessed whether surgical perturbation alone is sufficient to induce lymphoid cell remodeling in human GBM tissue using the ex vivo organotypic resection model (Fig. 2d). Phenotypic mapping of CD3^+^ T cells based on PD1, Tim3 and Lag3 expression revealed no major differences between unresected and resected regions at 24 h. In contrast, 72 h after resection, CD3⁺ T cells from resected regions displayed a marked shift toward an exhausted phenotype, characterized by an increased proportion of PD1⁺Tim3⁺Lag3⁺ triple-positive cells (Fig. 2e, f). We next examined whether these changes were associated with redistribution of canonical T cell differentiation states. While no differences were detected at 24 h, resection at 72 h was associated with an increased frequency of T_naive_ and a concomitant reduction in effector memory T cells (T_em_) compared with paired unresected regions (Fig. 2g). Subset-specific exhaustion analysis further showed that, at 72 h post-resection, T_eff_ and T_em_ cells exhibited increased exhaustion-like phenotypes, whereas T_cm_ and T_naive_ subsets remained largely unaffected (Fig. 2h). These ex vivo human data demonstrate that resection induces a late but coordinated remodeling of the lymphoid cell compartment, marked by accumulation of T cells co-expressing multiple checkpoint molecules and upregulation of exhaustion-associated markers in effector cells, mirroring the late lymphoid cell dysfunction observed in murine GBM models.

Together, these results establish that resection triggers a rapid and sustained remodeling of the GBM TME, characterized by early changes in myeloid cells and a later increase in T cell exhaustion-like phenotypes. TREM2 upregulation is a consistent and functionally relevant feature across models and time points, supporting its prioritization as a candidate target in the immediate post-resection window. Moreover, in the context of CAR-T cell therapy, the observed upregulation of exhaustion-associated markers provides a rationale for integrating immune checkpoint blockade with local CAR-T cell treatment to counteract resection-induced dysfunction.

### Local targeting of TREM2-expressing myeloid cells using CAR-T cells reshapes the GBM microenvironment

Given the consistent upregulation of TREM2 in myeloid cell populations after resection, we developed CAR-T cells directed against TREM2 using the 37012 scFv domain derived from the patent application WO2020123664A1. To model a GBM-educated myeloid cell state, bone marrow–derived macrophages (BMDM) were pre-exposed to conditioned media (CM) from SB28 or CT2A for 72 h and cultured with TREM2 CAR- or untransduced (UTD) T cells. SB28-CM exposure significantly increased TREM2 transcript and surface expression on BMDM, consistent with induction of a tumor-educated suppressive phenotype characterized by decreased iNOS and CD11c and increased Arg1, CD206 and PD-L1 expression (Supplementary Fig. 10a, b). TREM2 CAR-T cells reduced the number of viable CM-treated BMDM relative to UTD T cells in both tumor settings (Supplementary Fig. 10c, d). Phenotyping of residual macrophages after SB28-CM and CAR-T cell exposure showed increased expression of iNOS and CD11c and decreased expression of Arg1, CD206 and PD-L1 (Supplementary Fig. 10e), indicative of pro-inflammatory macrophages. Consistent with this, residual BMDM displayed higher uptake of GFP^+^ SB28 and CT2A tumor cells when pre-treated with TREM2 CAR-T cells compared to pre-treatment with UTD T cells (Supplementary Fig. 10f).

**Fig. 3:**
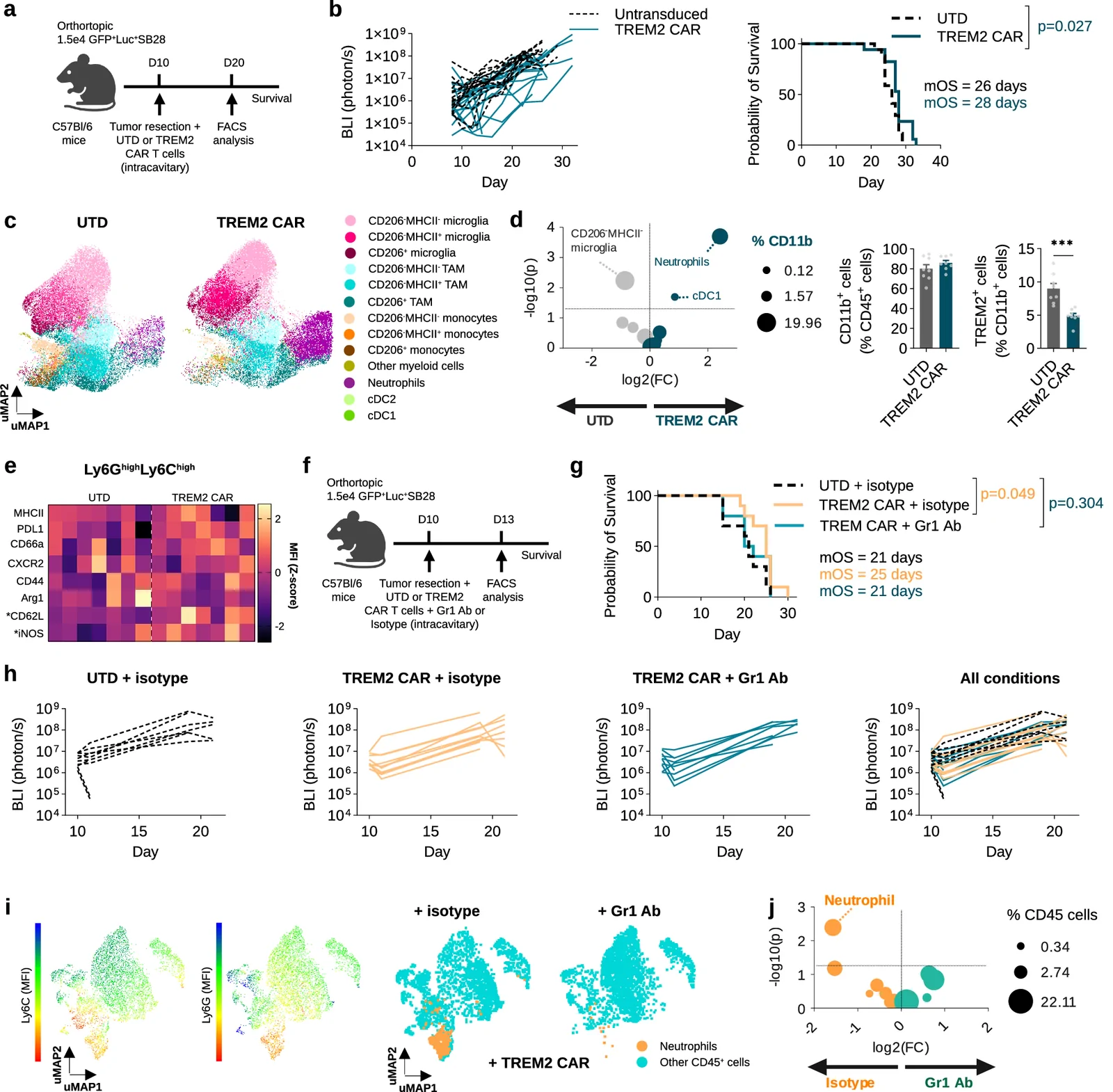
TREM2 CAR-T cells deplete TREM2⁺ myeloid cells and recruit pro-inflammatory neutrophils in vivo. **a** Experimental scheme of orthotopic SB28 tumor implantation, surgical resection, and local delivery of untransduced (UTD) or TREM2 CAR-T cells into the resection cavity. Created in BioRender. Nicola, C. (2026) https://BioRender.com/6o79xto. **b** Left to right: longitudinal bioluminescence imaging (BLI) quantification and Kaplan–Meier curves comparing UTD and TREM2 CAR-T cell-treated groups pooled from two independent experiments (*n* = 17 per group; log-rank test). mOS=median overall survival. **c** UMAP of CD45⁺CD11b⁺ myeloid cell populations showing major myeloid subset distribution in resected SB28 tumors treated with UTD (*n* = 8) or TREM2 CAR-T cells (*n* = 8). **d** Volcano plot of differential abundance of myeloid populations between resected SB28 tumors treated with UTD (*n* = 8) or TREM2 CAR-T cells (*n* = 8); dot size reflects mean population frequency. Frequency of CD11b⁺ myeloid cells among CD45⁺ immune cells and of TREM2⁺ cells among CD11b⁺ myeloid cells, pooled from two independent experiments (Unpaired two-tailed t-test, *n* = 8 per group). **e** Heatmap of z-scored mean fluorescence intensity (MFI) of indicated markers in neutrophils from UTD- and TREM2 CAR-T cell-treated SB28 tumors. **f** Experimental design for local neutrophil depletion by Gr1 antibody in SB28 tumors treated with TREM2 CAR-T cells. Created in BioRender. Nicola, C. (2026) https://BioRender.com/6o79xto. **g** Kaplan–Meier survival comparing UTD + isotype, TREM2 CAR-T cells + isotype, and TREM2 CAR-T cells + Gr1 antibody from one experiment (*n* = 10 per group; log-rank test). **h** Longitudinal BLI quantification for individual mice under the indicated conditions. **i** UMAP of CD11b⁺CD45⁺ myeloid cells in TREM2 CAR + isotype (*n* = 5) versus TREM2 CAR + Gr1 antibody (*n* = 4) groups. **j** Flow cytometry quantification of CD11b⁺ myeloid subsets in TREM2 CAR-T cells + isotype (*n* = 5) versus + anti-Gr1 (*n* = 4) antibody tumors (unpaired two-tailed t-test). Volcano plot of log2 fold-change in population frequencies; positive values, enrichment in anti-Gr1-treated mice (blue); negative values, enrichment in isotype-treated mice (yellow). Data are shown as mean ± SEM unless indicated. Unpaired or paired two-tailed t-tests were used as appropriate. *p < 0.05, **p < 0.01, ***p < 0.001. Source data and exact p values are provided as a Source Data file.

We then evaluated the therapeutic efficacy of TREM2 CAR-T cells in vivo in the SB28 model by injecting 1 × 10^6^ cells locally into the resection cavity (Fig. 3a). We focused on SB28 because it displayed high intratumoral TREM2 expression at baseline and after resection, together with a TAM-enriched TME. TREM2 CAR-T cell administration delayed tumor growth compared to UTD T cells, and survival was slightly prolonged (Fig. 3b, two independent experiments). Analysis of the TME three days after CAR-T cell injection revealed depletion of TREM2-expressing myeloid cell populations (Fig. 3d, right panel), notably TREM2^+^ TAM and TREM2^+^ neutrophils (Supplementary Fig. 11b). Remarkably, treatment with TREM2 CAR-T cells induced a loss of CD206^-^MHCII^-^ microglia and a substantial neutrophil infiltration (Fig. 3c, d and Supplementary Fig. 11a). Detailed phenotypic characterization by flow cytometry showed that infiltrating neutrophils expressed increased levels of CD62L and iNOS (Fig. 3e), markers that have been linked to antitumoral neutrophils^35,36^. To clarify the functional role of neutrophils in this context, we performed local neutrophil depletion using an anti-Gr1 antibody concomitant with TREM2 CAR-T cell administration (Fig. 3f). Mice treated with TREM2 CAR-T cells combined with isotype control showed a significant although small delay in tumor growth and improved survival compared to mice treated with UTD T cells (Fig. 3g, h), consistent with prior data (Fig. 3b). However, this therapeutic benefit was abrogated when neutrophils were locally depleted, suggesting that neutrophils are necessary mediators of TREM2 CAR-T cell efficacy. Flow cytometry analysis confirmed efficient depletion of Ly6C^high^Ly6G^high^ neutrophils (Fig. 3i, j) and showed that anti-Gr1 antibody administration had limited effects on the broader myeloid cell compartment (Fig. 3j and Supplementary Fig. 12). In addition to myeloid cell remodeling, TREM2 CAR-T cell treatment increased CD3⁺ T cell infiltration and reduced exhaustion-like phenotypes in CD8⁺ T_eff_ cells compared to UTD T cell-treated controls three days post-resection (Supplementary Fig. 11c, d), providing evidence that TREM2⁺ myeloid cells contribute to T cell dysfunction in the post-resection TME. These results show that local TREM2 CAR-T cell delivery is able to modify the GBM TME, resulting in a slight increase in survival, and support a role for neutrophils in the antitumor immune response mediated by TREM2 CAR-T cells.

### TREM2-directed myeloid cell modulation increases GD2 CAR-T cell function locally in GBM

Myeloid cells, and TAM in particular, are widely documented as key suppressors of T cell activity, impairing their proliferation, persistence, and effector function within the TME^37–39^. Despite the low efficacy of TREM2 CAR-T cells as monotherapy, the depletion of TREM2^+^ myeloid cells could be beneficial to improve GBM antigen-targeting CAR-T cell efficacy.

**Fig. 4:**
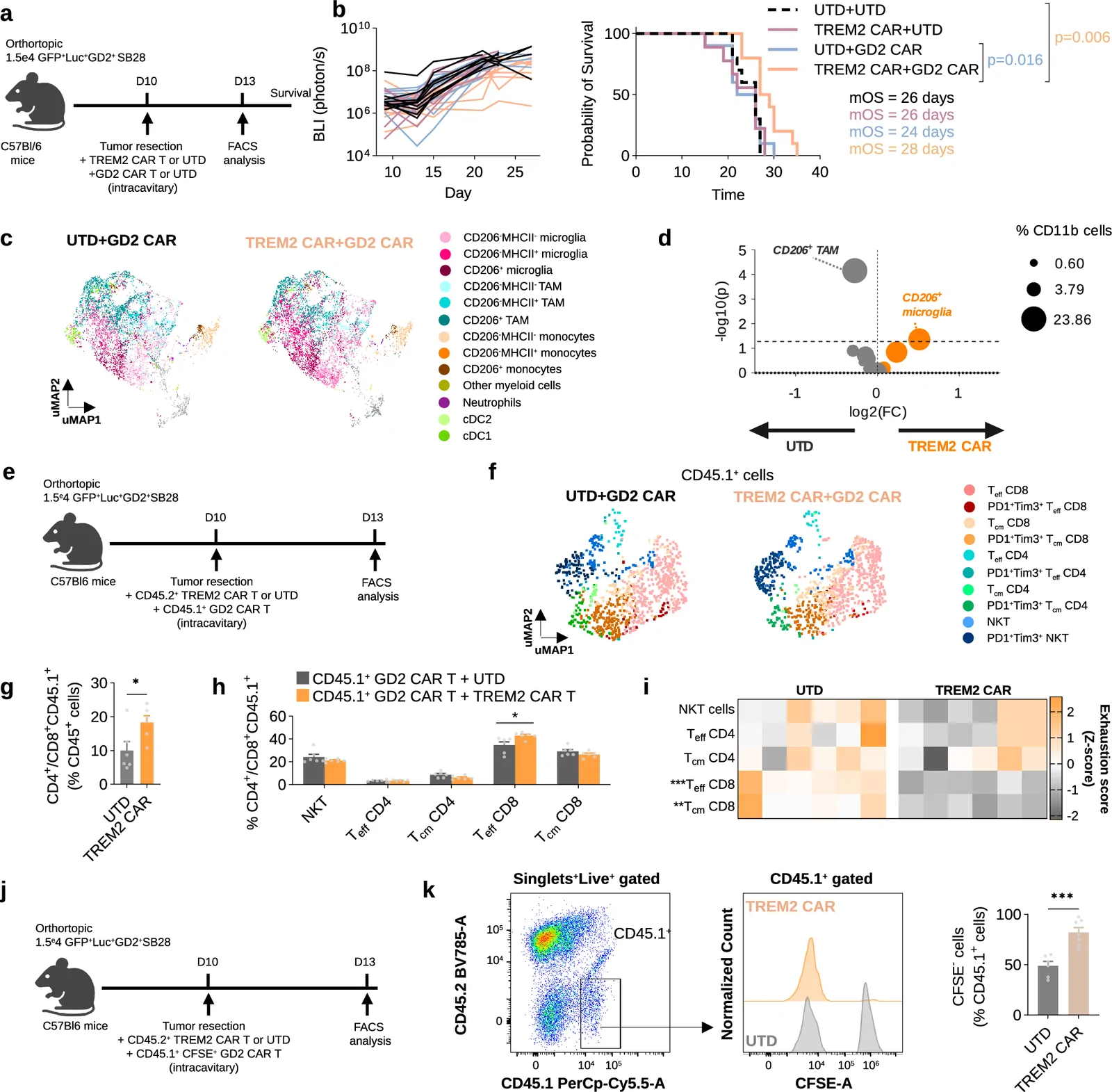
TREM2-targeting CAR-T cells enhance GD2-targeting CAR-T cell anti-tumor activity. **a** Schematic of experiments in mice bearing intracranial SB28 tumors treated with local co-injection of two T cell populations (5 × 10⁵ each): untransduced (UTD) + UTD, TREM2 CAR + UTD, UTD + GD2 CAR, or TREM2 CAR + GD2 CAR-T cells into the resection cavity. Created in BioRender. Nicola, C. (2026) https://BioRender.com/6o79xto. **b** Longitudinal bioluminescence imaging (BLI, left) and Kaplan–Meier survival of resected SB28-bearing mice treated with UTD + UTD, TREM2 CAR + UTD, UTD + GD2 CAR, or TREM2 CAR + GD2 CAR-T cells from one experiment (*n* = 10/ group; log-rank test). mOS=median overall survival. **c** UMAP of tumor-infiltrating CD45⁺CD11b⁺ cells following GD2 CAR + UTD versus GD2 + TREM2 CAR-T cell treatment (*n* = 6/group). **d** Volcano plot of log2 fold-change in population frequencies between groups from one experiment (*n* = 6/group; unpaired two-tailed t-test); positive values, enrichment in TREM2 CAR-T cell-treated mice (yellow); negative values, enrichment in UTD T cell-treated mice (gray). **e** Experimental design for adoptive transfer of GD2 CAR-T cells with UTD or TREM2 CAR-T cells. Created in BioRender. Nicola, C. (2026) https://BioRender.com/6o79xto. **f** UMAP of tumor-infiltrating CD4⁺/CD8⁺CD45.1⁺ T cells from GD2 CAR-T cell+UTD or GD2 CAR-T cell+TREM2 CAR-T cell groups, with cell-type cluster annotations. **g** Flow cytometry quantification of CD4⁺/CD8⁺CD45.1⁺ T cells in tumors of UTD- versus TREM2 CAR-T cell-treated mice from one experiment (*n* = 6/group, unpaired two-tailed t-test). **h** Frequencies of NKT, T_eff_ CD4, T_cm_ CD4, T_eff_ CD8 and T_cm_ CD8 subsets among CD3⁺CD45.1⁺ GD2 CAR-T cells co-administered with UTD or TREM2 CAR-T cells (*n* = 6/group, unpaired two-tailed t-test). **i** Heatmap of exhaustion-like scores (z-score; composite of exhaustion-associated markers, see Methods) in T cell subsets from GD2 CAR + UTD and GD2 + TREM2 CAR-T cell-treated mice (*n* = 6/group). **j** Experimental design for the in vivo persistence assay in which CFSE-labeled GD2 CAR-T cells are co-administered with UTD or TREM2 CAR-T cells. Created in BioRender. Nicola, C. (2026) https://BioRender.com/6o79xto. **k** Representative flow cytometry plots (left) and quantification (right) of GD2 CAR-T cell proliferation with UTD or TREM2 CAR-T cells (*n* = 6 UTD and 7 TREM2 CAR per group, unpaired two-tailed t-test). Data are shown as mean ± SEM. *p < 0.05, **p < 0.01, ***p < 0.001. Source data and exact *p* values are provided as a Source Data file.

To test this hypothesis, we investigated a combinatorial approach that used TREM2 CAR-T cells and GD2 CAR-T cells. In vitro assays confirmed that BMDM exposed to SB28-CM and pretreated with TREM2 CAR-T cells for 72 h significantly restored the cytotoxic activity and IFNγ secretion capacity of GD2 CAR-T cells after coculture with GD2^+^ SB28 cells, compared to untreated or UTD T cell-treated BMDM (Supplementary Fig. 13a–c). Additionally, BMDM pre-treatment with TREM2 CAR-T cells effectively reversed the suppression of GD2 CAR-T cell proliferation, despite no notable change in GD2 CAR-T cell phenotype, which remained predominantly T_eff_, either when BMDM were pre-treated with T cells or not (Supplementary Fig. 13d, e). In vivo, co-injection of TREM2 and GD2 CAR-T cells into the resection cavity in the GD2^+^ SB28 murine model led to a modest but significant improvement in survival compared to GD2 CAR-T cells injected alone (Fig. 4a, b, one independent experiment). Immunophenotypic analysis of the TME post-treatment revealed a less immunosuppressive profile when TREM2 CAR-T cells were combined with GD2 CAR-T cells compared to the combination with UTD T cells, with reduced CD206^+^ TAM infiltration (Fig. 4c, d and Supplementary Fig. 14). To assess the impact of TREM2 CAR-T cell co-administration on GD2 CAR-T cell persistence and phenotype, we used a dual congenic transfer model involving CD45.2^+^ TREM2 CAR-T cells and CD45.1^+^ GD2 CAR-T cells (Fig. 4e). Co-injection of TREM2 CAR-T cells enhanced GD2 CAR-T cell persistence in the tumor three days post-injection (Fig. 4g) and slightly increased CD8^+^ T_eff_ cells (Fig. 4f, h) while decreasing exhaustion-like phenotypes in CD8^+^ T_eff_ and T_cm_ cells (Fig. 4i). Finally, TREM2 CAR-T cell co-treatment enhanced in vivo proliferation of CFSE-labeled CD45.1^+^ GD2 CAR-T cells (Fig. 4j, k). Together, these findings suggest that targeting TREM2⁺ myeloid cells via TREM2 CAR-T cells is associated with improved functional state of the TME and enhanced proliferation, persistence, and differentiation of co-administered GBM-directed CAR-T cells.

### Local immune checkpoint inhibition does not improve CAR-T cell function in GBM

We next sought to investigate whether the addition of local immune checkpoint blockade could further enhance CAR-T cell efficacy, following our observations of increased exhaustion-like profiles after resection (Fig. 2c). Checkpoint blockade targeting PD1 and Tim3 has shown promising results in reinvigorating T cell function in various solid tumors^40^, and their expression is particularly enriched in T cells in GBM^10,41^.

**Fig. 5:**
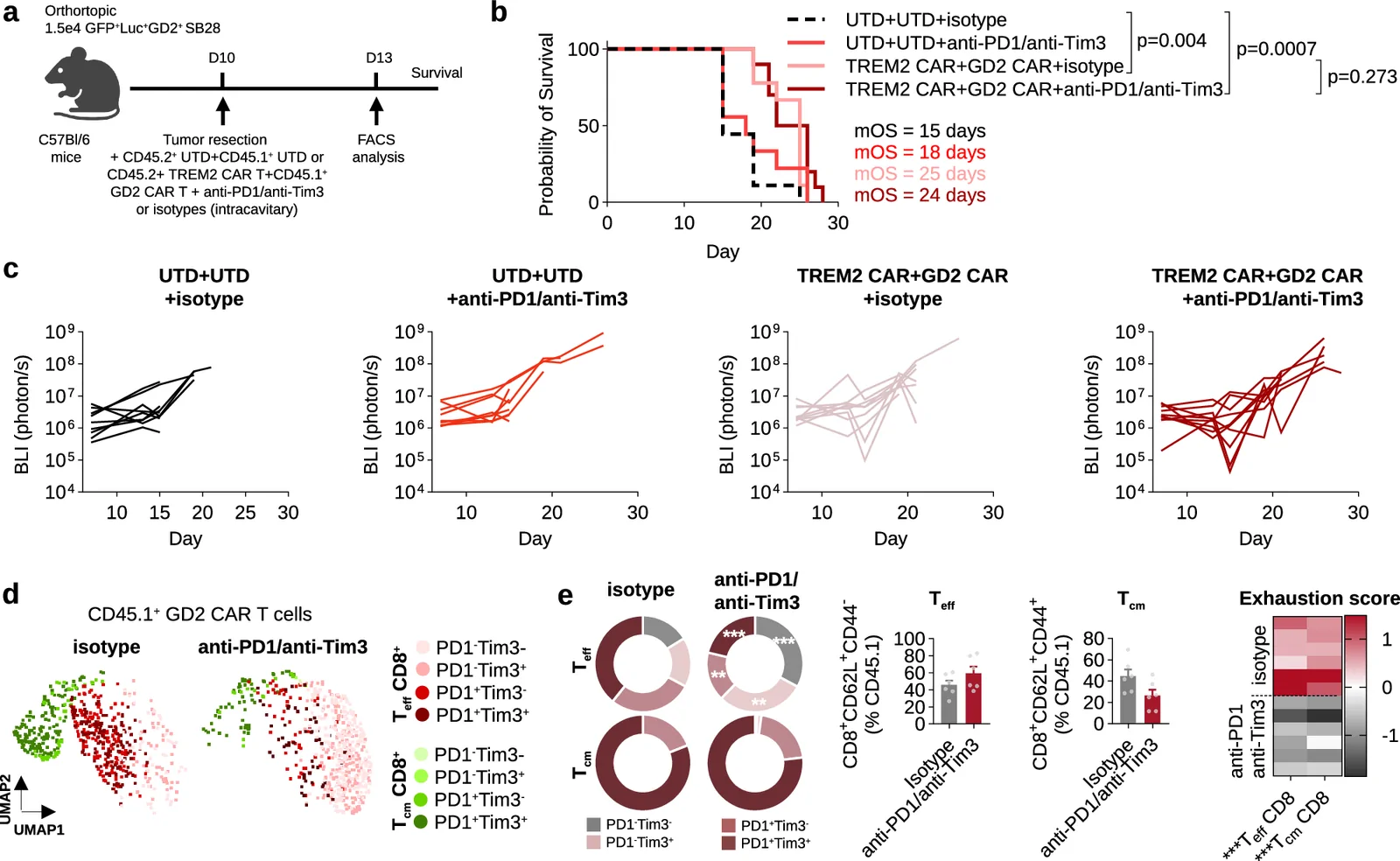
Anti-PD1/anti-Tim3 antibodies do not enhance efficacy of TREM2 and GD2 CAR-T cell co-administration, despite phenotypic modulation. **a** Schematic of the experiments performed in mice bearing intracranial SB28 tumors and treated with local co-injection of two T cell populations (5 × 10^5^ each): CD45.2⁺untransduced (UTD) or TREM2 CAR-T cells + CD45.1⁺ UTD or GD2 CAR-T cells into the resection cavity combined with anti-PD-1/anti-Tim-3 antibodies or isotype controls. Created in BioRender. Nicola, C. (2026) https://BioRender.com/6o79xto. **b** Kaplan–Meier survival analysis comparing UTD/UTD + isotype control, UTD/UTD + anti-PD1/anti-Tim3 antibodies, TREM2 CAR/GD2 CAR+ isotype control, and TREM2 CAR/GD2 CAR + anti-PD1/anti-Tim3 antibodies from one experiment (*n* = 10 mice per group, log-rank test). mOS=median overall survival. **c** Longitudinal BLI quantification for individual mice under the indicated treatment conditions. **d** Representative uniform manifold approximation and projection (UMAP) visualization of tumor-infiltrating CD45.1⁺CD8^+^ GD2 CAR-T cells in mice receiving TREM2 and GD2 CAR-T cells combined with anti-PD-1/anti-Tim-3 or isotype control antibodies, colored by clusters (*n* = 5/group). **e** Donut charts depicting the distribution of intratumoral T effector (T_eff_) and central memory (T_cm_) CD45.1⁺ GD2 CAR-T cells stratified by PD1 and Tim3 expression (double negative, single positive, or double positive) 72 h after surgery, comparing isotype- and anti-PD1/anti-Tim3 antibody-treated mice. Frequencies of T_eff_ CD8 (CD8⁺CD62L⁻CD44⁺) and T_cm_ CD8 (CD8⁺CD62L⁺CD44⁺) among CD45.1⁺ GD2 CAR-T cells injected with anti-PD1/anti-Tim3 antibodies or isotype controls (unpaired two-tailed t-test). Heatmap of exhaustion-like score (z-score; composite of exhaustion-associated markers, see Methods) for T_eff_ and T_cm_ CD8⁺CD45.1⁺ GD2 CAR-T cells, which was significantly lower in anti-PD1/anti-Tim3-treated mice (T_eff_ CD8, T_cm_ CD8; *n* = 6 mice per group, unpaired two-tailed t-test). Data are shown as mean ± SEM. Source data and exact *p* values are provided as a Source Data file.

We thus explored whether local delivery of anti-PD1 and anti-Tim3 antibodies enhanced the efficacy of combined TREM2 and GD2 CAR-T cell therapy in the SB28 model (Fig. 5a). Consistent with data shown in Fig. 4b, local co-injection of the two CAR-T cell populations with isotype controls induced a significant survival benefit over injection of UTD T cells. However, addition of anti-PD1 and anti-Tim3 antibodies did not further improve survival (Fig. 5b), nor significantly affect tumor growth (Fig. 5c). Despite this, flow cytometry analysis of CD8^+^ GD2 CAR-T cells (CD4^+^ GD2 CAR-T cells were undetectable in this experiment) recovered from the TME revealed a clear immunomodulatory effect of checkpoint blockade, with anti-PD1/anti-Tim3 therapy increasing the proportion of PD1^-^Tim3^-^ and PD1^-^Tim3^+^ CD8^+^ T_eff_ cells and reducing exhaustion-associated scores of CD8^+^ T_cm_ and T_eff_ cell subsets (Fig. 5d, e). Altogether, while PD1 and Tim3 blockade effectively modulated CAR-T cell exhaustion-associated marker expression, this did not translate into enhanced therapeutic efficacy in the SB28 model, suggesting that additional strategies are required to overcome the intrinsic resistance mechanisms in the immunosuppressive GBM TME.

**Fig. 6:**
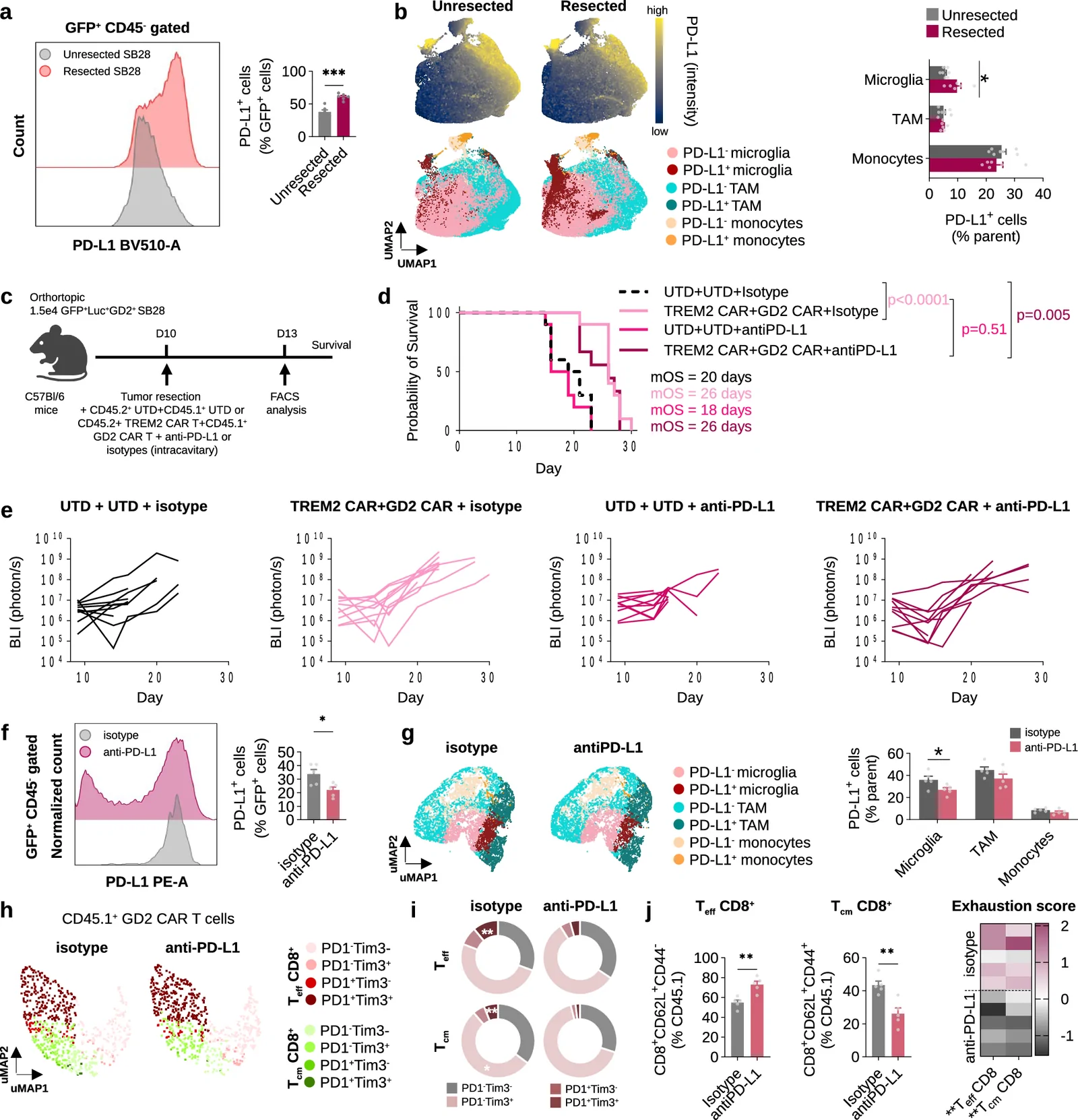
Anti-PD-L1 antibody administration does not enhance efficacy of TREM2/GD2 CAR-T cell co-administration, despite phenotypic modulation. **a** Representative histogram and quantification of PD-L1 MFI on SB28 GFP⁺ cells (*n* = 6 mice per group) 24 h after surgery in unresected and resected mice (unpaired two-tailed t-test). **b** Left: UMAP of tumor-infiltrating CD45⁺CD11b⁺ myeloid cells showing PD-L1 expression 24 h after surgery in unresected and resected SB28 mice, colored by cell type. Right: flow cytometry quantification of PD-L1⁺ cells across microglia, TAM and monocytes (*n* = 5 mice/ group, unpaired two-tailed t-test). **c** Schematic: mice bearing intracranial SB28 received local co-injection of CD45.2⁺ untransduced (UTD) or TREM2 CAR-T cells + CD45.1⁺ UTD or GD2 CAR-T cells into the resection cavity, combined with anti-PD-L1 antibody or isotype control. Created in BioRender. Nicola, C. (2026) https://BioRender.com/6o79xto. **d** Kaplan–Meier survival curves of SB28-bearing mice treated as in (**c**) from one experiment (*n* = 10 for all group except for TREM2 CAR + GD2 CAR + anti-PD-L1 (*n* = 9), log-rank test). **e** Longitudinal bioluminescence imaging (BLI) quantification of tumor growth following treatments in (**c**). **f** Representative histogram and quantification of PD-L1 MFI on SB28 GFP⁺ cells 72 h after surgery in PD-L1 and isotype control antibody-treated mice receiving TREM2 and GD2 CAR-T cells from one experiment (*n* = 5 mice per group, unpaired two-tailed t-test). **g** UMAP (left) and flow cytometry quantification (right) of CD45⁺CD11b⁺ myeloid cells showing PD-L1 expression in microglia, TAM and monocytes in anti-PD-L1 and isotype control-treated mice receiving GD2 and TREM2 CAR-T cells (*n* = 5/group). **h** UMAP of tumor-infiltrating CD45.1⁺CD3⁺CD8⁺ GD2 CAR-T cells colored by manually annotated clusters following TREM2/GD2 CAR-T cell treatment with anti-PD-L1 or isotype control antibodies (*n* = 5/group). **i** Donut charts of T_eff_ and T_cm_ CD45.1⁺ GD2 CAR-T cells stratified by PD1/Tim3 expression (double negative, single positive, double positive) 72 h after surgery, comparing anti-PD-L1 and isotype control mice (*n* = 5/group, unpaired two-tailed t-test). **j** Frequencies of CD8 T_eff_ (CD8⁺CD62L⁻CD44⁺) and T_cm_ (CD8⁺CD62L⁺CD44⁺) among CD45.1⁺ GD2 CAR-T cells comparing PD-L1 and isotype control mice (unpaired two-tailed t-test). Heatmap of exhaustion-like score (z-score; composite of exhaustion-associated markers, see Methods) for T_eff_ and T_cm_ CD8⁺CD45.1⁺ GD2 CAR-T cells (*n* = 5 mice/group). Data are shown as mean ± SEM. Data are shown as mean ± SEM. Source data and exact p values are provided as a Source Data file.

Beyond T cell intrinsic checkpoints, PD-L1 expression on GBM-associated myeloid cells has been shown to dampen T cell responses and promote tumor immune evasion^42^. We therefore evaluated the impact of surgical resection on PD-L1 expression in tumor and GBM-associated myeloid cells. Flow cytometry analysis of SB28 tumors 24 h post-resection revealed increased PD-L1 expression on tumor cells compared to unresected controls (Fig. 6a), as well as an increase in the proportion of PD-L1^+^ microglia, while no change in PD-L1 expression by monocytes and TAM was observed (Fig. 6b). To further assess the potential of PD-L1 inhibition on CAR-T cell efficacy, we treated SB28 tumor-bearing mice with a combination of TREM2 and GD2 CAR-T cells together with local co-administration of anti-PD-L1 or isotype control antibodies (Fig. 6c). Similar to PD1/Tim3 inhibition, addition of anti-PD-L1 treatment to the TREM2 and GD2 CAR-T cell combination did not improve survival (Fig. 6d) or delay tumor growth (Fig. 6e). Flow cytometry analysis of SB28 tumors on day 3 post-resection showed a reduction in the proportion of PD-L1^+^ tumor cells (Fig. 6f) and PD-L1⁺ microglia (Fig. 6g) in mice receiving TREM2 and GD2 CAR-T cells with anti-PD-L1 antibodies, while other subsets remained largely unchanged. In addition, anti-PD-L1 antibody treatment reduced the proportion of CD206⁺ and CD206^-^MHCII⁺ TAM (Supplementary Fig. 15). Analysis of intratumoral GD2 CAR-T cells showed a decrease in PD1⁺Tim3⁺ cells among CD8⁺ T_eff_ and T_cm_ cell subsets (CD4^+^ GD2 CAR-T cells were undetectable in this experiment) upon anti-PD-L1 antibody co-administration (Fig. 6h/i), concomitant with a reduction in exhaustion-like scores (Fig. 6j). Collectively, these results show that local targeting of PD1/Tim3 or PD-L1 does not further increase efficacy of local TREM2 and GD2 CAR-T cell treatment.

### Administration of GBM-targeting CAR-T cells is more efficient in the neoadjuvant than in the adjuvant setting

Given the remodeling of TME post-resection, we explored whether shifting the timing of CAR-T cell delivery to a neoadjuvant setting could improve therapeutic outcome. In GBM, neoadjuvant checkpoint blockade has shown superior immune activation and survival benefits compared to adjuvant regimens^24,25^, raising the possibility that CAR-T cell therapy may follow a similar pattern.

**Fig. 7:**
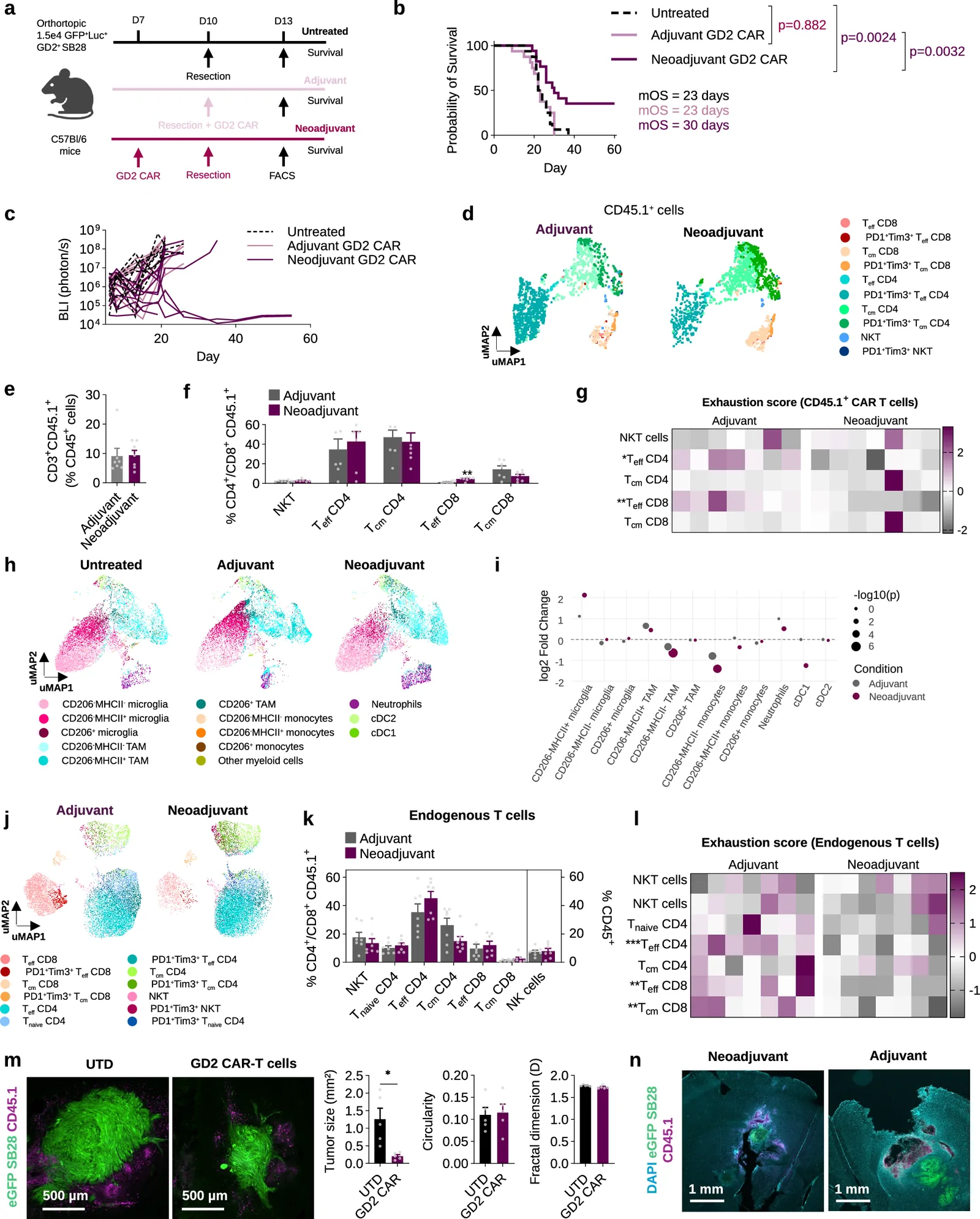
Neoadjuvant GD2 CAR-T cell therapy enhances tumor control, prolongs survival, and improves intratumoral T cell function compared to adjuvant treatment. **a** Experimental scheme of SB28-bearing mice untreated or given GD2 CAR-T cells neoadjuvant (3 days before resection) or adjuvant (resection cavity). Created in BioRender. Nicola, C. (2026) https://BioRender.com/6o79xto. **b** Kaplan–Meier survival of resected SB28-bearing mice untreated or treated with adjuvant or neoadjuvant GD2 CAR-T cells, pooled from two independent experiments (*n* = 16 for untreated and adjuvant groups and 17 for neoadjuvant group; log-rank test). mOS=median overall survival. **c** Longitudinal BLI quantification in the three groups from one experiment (*n* = 10 per group). **d** UMAP of tumor-infiltrating CD3⁺CD45.1⁺ GD2 CAR-T cells (three groups) colored by annotated clusters (*n* = 7/group). **e** Frequencies of CD3⁺CD45.1⁺ CAR-T cells in tumors, neoadjuvant versus adjuvant (*n* = 7/ group, unpaired two-tailed t-test). **f** Frequencies of NKT, T_eff_ CD4, T_cm_ CD4, T_eff_ CD8 and T_cm_ CD8 subsets among CD4⁺/CD8⁺CD45.1⁺ GD2 CAR-T cells, neoadjuvant versus adjuvant (*n* = 7/group, Unpaired two-tailed t-test). **g** Heatmap of exhaustion-like score (z-score, see Methods) in T cell subsets, neoadjuvant versus adjuvant (*n* = 7/group). **h** UMAP of tumor-infiltrating CD11b⁺CD45⁺ cells (three groups), colored by clusters. **i** Dot plot of myeloid subset frequencies: log2 fold-change (neoadjuvant/adjuvant) versus untreated (x-axis); positive/negative, higher/lower frequency versus untreated; colors, adjuvant (gray) and neoadjuvant (magenta); circle size reflects −log10(p) (*n* = 6 for untreated group, *n* = 7 for adjuvant and neoadjuvant groups, one-way ANOVA with Bonferroni correction). **j** UMAP of tumor-infiltrating CD4⁺/CD8⁺CD45.2⁺ endogenous lymphoid cells (three groups, *n* = 7/condition) colored by annotated clusters (see Methods, Supplementary Table 1). **k** Quantification of CD4⁺/CD8⁺CD45.2⁺ T cell subsets (NKT, naïve CD4, effector CD4, central memory CD4, effector CD8, central memory CD8) and NK cells, adjuvant (gray) versus neoadjuvant (purple) (mean ± SEM, *n* = 7/ group, unpaired two-tailed t-test). **l** Heatmap of exhaustion-like score (z-score, see Methods) in endogenous CD45.2⁺ T cells, neoadjuvant versus adjuvant (*n* = 7 per group, unpaired two-tailed t-test). **m** Representative immunofluorescence (GFP⁺ SB28, green; CD45.1, magenta; scale bar = 500 µm) and quantification of tumor size (mm²), circularity and fractal dimension in UTD- or GD2 CAR-T–treated mice (day 7 injection, day 10 analysis; *n* = 5 per group, unpaired two-tailed t-test). **n** Representative immunofluorescence (DAPI, cyan; GFP⁺ SB28, green; CD45.1, magenta; scale bar = 1 mm) showing GD2 CAR-T cell distribution relative to the resection cavity at 24 h post-resection, neoadjuvant versus adjuvant (representative of *n* = 5 per group). Data are shown as mean ± SEM. Source data and exact *p* values are provided as a Source Data file.

To directly test this, we compared the therapeutic efficacy of GD2 CAR-T cells administered either as neoadjuvant (3 days before resection) or adjuvant (into the resection cavity) in GD2^+^ SB28- (Fig. 7a) and CT2A-bearing mice. Across both models, the neoadjuvant CAR-T cell approach significantly improved antitumor responses and survival compared to adjuvant CAR-T cells or surgery alone (Fig. 7b, c and Supplementary Fig. 16a, b, two independent experiments in each model). To better understand this effect, we analyzed the phenotype and persistence of CAR-T cells administered in either setting three days after resection in the SB28 model (Fig. 7d). Flow cytometry analysis revealed no significant difference in CAR-T cell persistence between the two treatment arms (Fig. 7e). However, CD8^+^ T_eff_ cells were significantly reduced in the adjuvant setting (Fig. 7d, f), and these cells, along with CD4^+^ T_eff_ cells, exhibited higher exhaustion-like scores compared to the neoadjuvant condition (Fig. 7g). We then examined how neoadjuvant vs adjuvant CAR-T cell delivery impacted the broader TME. GD2 CAR-T cell administration induced a reduction in CD206^-^MHCII^-^ TAM and CD206^-^MHCII^-^ monocytes in both settings. In addition, neoadjuvant treatment was mostly associated with increased frequencies of CD206^-^MHCII^+^ microglia, whereas adjuvant treatment was associated with increased frequencies of CD206^-^MHCII^+^ TAM and neutrophils (Fig. 7h, i and Supplementary Fig. 17), suggesting no major differences in TME modulation between the two conditions. This contrasted with the CT2A model, in which only neoadjuvant CAR-T cell therapy led to myeloid cell modulation, including an increase in CD206^+^ and CD206^-^MHCII^+^ monocytes and cDC1 (Supplementary Fig. 18). Regarding the lymphoid cell compartment, although the overall proportions of endogenous T cell subsets and NK cells remained unchanged between treatment arms in SB28 tumors (Fig. 7j, k), endogenous CD4^+^ T_eff_, CD8^+^ T_eff_, and CD8^+^ T_cm_ cells showed higher exhaustion-like score in the adjuvant compared to the neoadjuvant group (Fig. 7l). Collectively, the survival benefit and reduced T cell exhaustion-like score support a timing-dependent model in which pre-operative delivery places CAR-T cells in a less suppressive environment, preserving effector fitness and improving tumor control. To investigate the impact of neoadjuvant GD2 CAR-T cell treatment on tumor structure at the time of resection, we performed immunofluorescence analysis of GFP⁺ tumor area and morphology at day 10 post-implantation. Neoadjuvant GD2 CAR-T cell treatment significantly reduced tumor size compared to UTD T cell-treated controls, while tumor circularity and fractal dimension remained unchanged, indicating a reduction in tumor bulk without alteration of invasion capacity (Fig. 7m). CD45.1⁺ GD2 CAR-T cells were detected at tumor borders, consistent with active engagement of residual tumor tissue prior to surgery. Furthermore, immunofluorescence performed 24 h post-resection revealed that neoadjuvant CAR-T cells were distributed around the resection cavity and along residual tumor margins, whereas adjuvant CAR-T cells appeared predominantly confined within the resection cavity, with limited engagement of the surrounding tumor parenchyma (Fig. 7n). These data provide a mechanistic basis for the superior efficacy of neoadjuvant delivery, in which CAR-T cells are pre-positioned at the tumor-parenchyma interface prior to surgery.

As TREM2⁺ myeloid cells are present pre-operatively and further intensify after surgery, and as adjuvant co-delivery of TREM2/GD2 CAR-T cells improved tumor control, we next tested whether pre-operative depletion/reprogramming of TREM2⁺ cells would augment the efficacy of neoadjuvant GD2 CAR-T cell therapy. We injected TREM2 CAR-T cells, GD2 CAR-T cells or the TREM2/GD2 CAR-T cell combination 3 days before resection in the SB28 model (Supplementary Fig. 19a). While neoadjuvant GD2 CAR-T cells improved survival relative to UTD T cell administration, treatment with TREM2 CAR-T cells alone conferred no survival advantage. Unexpectedly, addition of TREM2 CAR-T cells to GD2 CAR-T cells did not extend survival and blunted the benefit of GD2 CAR-T cell treatment (Supplementary Fig. 19b, c). Noteworthy, the limited effect of TREM2 CAR-T cell administration as a monotherapy was observed both in the neoadjuvant and adjuvant (Fig. 3b) settings, indicating that it was not timing-dependent. TME analysis 72 h after resection revealed higher frequencies of CD206^-^MHCII⁺ monocytes and CD206^-^MHCII⁺ TAM in the TREM2/GD2 CAR-T cell combination compared with UTD/GD2 CAR-T cell-treated tumors, together with reduced CD206⁺ microglia and neutrophils (Supplementary Figs. 19d and 20). The frequency of TREM2⁺ cells among CD11b⁺ myeloid cells was comparable between the TREM2/GD2 and UTD/GD2 CAR-T cell treatment groups (Supplementary Fig. 19e). Furthermore, addition of TREM2 CAR-T cells did not affect the persistence of CD8^+^ GD2 CAR-T cells (CD4^+^ GD2 CAR-T cells were undetectable in this experiment) nor influenced the proportions of CD8⁺ T_eff_ and T_cm_ cells (Supplementary Fig. 19f, g) but resulted in an increase in CD8⁺ T_cm_ exhaustion-like scores (Supplementary Fig. 19h). Collectively, these results show that delivering GBM-targeting CAR-T cells before surgery outperforms adjuvant administration by preserving CAR-T cell effector function, whereas pre-operative addition of TREM2 CAR-T cells to GD2 CAR-T cells suppresses this benefit, underscoring the timing-dependent nature of myeloid cell modulation in the perioperative window.

## Discussion

Here, we demonstrate that surgical resection is not an immunologically neutral intervention but a dominant and time-dependent driver of immune remodeling that critically conditions CAR-T cell efficacy. Through longitudinal immune profiling, spatial mapping, and functional CAR-T cell studies in murine models and human organotypic GBM tissue, we show that resection induces an early, myeloid cell-dominated immune remodeling in which TREM2⁺ cell expansion is a prominent and functionally relevant feature, followed by late upregulation of exhaustion-associated markers in lymphoid cells. Importantly, our data reveal that the therapeutic outcome of CAR-T cell therapy in GBM is highly contingent on when and where cells are delivered relative to surgery. These findings suggest that surgical timing is a key determinant of CAR-T cell functionality and provide a mechanistic rationale for perioperative and neoadjuvant strategies that target resection-induced myeloid cell niches to overcome resistance in GBM.

Surgical resection remains a cornerstone of GBM management, yet accumulating evidence indicates that it profoundly remodels the TME^14,15^ in ways that could either transiently support or ultimately hinder subsequent therapies. Here, we show that GBM resection triggers rapid and sustained immune remodeling dominated by early changes in the myeloid cell compartment. Across two immunologically distinct murine models, SB28 and CT2A, surgical resection consistently induced upregulation of TREM2 across multiple myeloid cell subsets, which correlated with accelerated tumor regrowth following surgery. These findings are consistent with prior reports demonstrating that surgical resection induces profound but model-dependent immune remodeling in GBM. Notably, Khalsa et al.^15^ reported that, in the CT2A model, resection was associated with increased infiltration of T cells, activated microglia and SiglecF⁺ macrophages, alongside a reduction in resident macrophages, resulting in a more immunostimulatory post-operative TME. In contrast, our data reveal a distinct remodeling trajectory characterized by enrichment of TREM2⁺ myeloid cell populations and delayed lymphoid cell exhaustion-like phenotypes, underscoring that baseline TME composition, tumor genetics and surgical context critically shape post-resection immune evolution. Similarly, longitudinal analyses by Bastiancich et al.^14^ in the GL261 model demonstrated a shift toward myeloid cell predominance after resection, with enrichment of suppressive macrophage states and limited recovery of effective antitumor immunity, supporting the concept that surgery can impose a durable myeloid cell-driven constraint on immune responsiveness. Mechanistically, surgical injury has been shown to induce hypoxia^43^ and a wound-healing response^14,44^ within the resection cavity, both mechanisms being known drivers of TREM2 expression through HIF-1α–dependent transcriptional pathways^45^ and TGFβ- and extracellular matrix–mediated signaling^46^, respectively. In this context, sustained exposure to hypoxic and fibrotic cues is likely to reinforce the survival, spatial retention and functional polarization of TREM2⁺ myeloid cells at the tumor margin, thereby stabilizing immunosuppressive myeloid cell niches in the post-operative tumor bed. In line with this, the accumulation and spatial repositioning of TREM2⁺ myeloid cells shown here in SB28 murine tumors and ex vivo human GBM slices support a conserved, surgery-induced program that may actively constrain local immune responses and contribute to early relapse. It should be noted that, because surgical cytoreduction removes the bulk tumor mass, the myeloid cell compartment profiled after resection is necessarily enriched for cells residing within the invasive tumor margin and the early recurrence niche rather than the original tumor core. The myeloid cell alterations described here may therefore partly reflect this shift in spatial sampling, in addition to genuine surgery-induced reprogramming, a distinction that motivated our spatial immunofluorescence characterization of the post-resection tumor bed.

TREM2 is a damage-sensing receptor that is upregulated in myeloid cells in response to diverse tissue injury signals, including lipid-associated damage-associated molecular patterns (DAMP), hypoxia, and extracellular matrix remodeling^47^, all of which are hallmarks of the post-surgical brain microenvironment. The observation that this TREM2⁺ myeloid cell expansion occurs in the absence of any vascular perfusion in our human organotypic model strongly suggests that local transcriptional reprogramming of tissue-resident populations, rather than peripheral monocyte recruitment, is the primary initiating mechanism. In vivo, blood-borne monocytes are known to infiltrate GBM and contribute to the TREM2-expressing myeloid cell compartment alongside microglia^33,48^, and likely further amplify this locally initiated response. We acknowledge that the organotypic model does not allow discrimination between microglia and monocyte-derived macrophages at the ontogenic level and does not fully recapitulate the vascular component of the post-resection niche in vivo, representing an important limitation when extrapolating these findings to the clinical setting.

Together, our findings provide a mechanistic framework linking surgical injury, myeloid cell reprogramming, and therapeutic resistance, and support TREM2⁺ myeloid cells as a rational candidate target for perioperative immunomodulatory strategies in GBM. These findings in murine models are broadly consistent with human GBM single-cell transcriptomic datasets, which have identified a TREM2^high^ macrophage state enriched in TAM, characterized by lipid metabolism gene signatures, immunosuppressive signaling, and spatial association with hypoxic tumor regions^8,49^. The cross-species scRNAseq analysis presented here confirmed that TREM2 transcripts are broadly distributed across microglia, macrophages, monocytes, and DC in both murine and human GBM, supporting conservation of TREM2 expression across myeloid cell lineages. Nevertheless, important differences between species warrant consideration. At the protein level, flow cytometry-based quantification suggests that TREM2 upregulation post-resection is most pronounced in infiltrating monocyte-derived populations in SB28 and CT2A, likely reflecting the greater contribution of blood-borne myeloid cells in rapidly growing syngeneic tumors. Additionally, human GBM harbors a more complex and heterogeneous myeloid cell landscape, including border-associated macrophages and ontogenically distinct TAM subpopulations^50^, which may not be fully recapitulated in transplantable murine models. These caveats should be borne in mind when extrapolating our findings to the clinical setting.

While direct targeting of TREM2 has only recently begun to be explored in solid tumors^39,51^, peri-operative modulation of myeloid cells has demonstrated therapeutic benefit in several cancer models^11,52^. More recently, two landmark studies demonstrated that IL-12-armored TREM2 CAR-T cells can effectively deplete immunosuppressive TAM and remodel the TME in peripheral tumor models, including metastatic ovarian and lung cancer, with durable antitumor efficacy and favorable safety profiles^53,54^. Time-resolved single-cell transcriptomic analyses in GBM further showed that TREM2 antagonism disrupts monocyte-to-TAM differentiation^55^, providing a direct proof-of-concept for targeting TREM2⁺ myeloid cells as a therapeutic strategy. In line with these observations, our findings in murine GBM show that TREM2 CAR-T cells effectively depleted TREM2⁺ myeloid cells and unexpectedly recruited pro-inflammatory neutrophils, which proved essential for local tumor control. While DAMP-mediated clearance of dying TREM2⁺ cells may partly contribute to neutrophil recruitment^56^, their requirement for therapeutic efficacy, as demonstrated by anti-Gr1 depletion, as well as their pro-inflammatory phenotype argue for an active antitumor role rather than passive tissue neutrophilia. While TREM2 targeting alone did not markedly prolong survival, its combination with GD2 CAR-T cells resulted in improved survival, increased GD2 CAR-T cell proliferation and persistence, and reduced exhaustion-like phenotypes. These effects are consistent with a broader paradigm in solid tumors whereby relieving myeloid cell-mediated suppression enhances antigen-specific T cell activity^57^ and preserves CAR-T cells in a less terminally exhausted state^58^. In this regard, neutrophils recruited by local TREM2 CAR-T cell delivery might contribute indirectly to the improved efficacy of GD2 CAR-T cells. Indeed, specific neutrophil subsets have been shown to promote T cell activation and antitumor immunity in solid cancers^59^, supporting a model in which TREM2-targeted myeloid cell remodeling creates a permissive inflammatory niche that amplifies CAR-T cell function. Notably, recent studies have reported context-dependent roles of TREM2 in glioma, ranging from immunosuppressive functions in myeloid cell-dominant SB28 tumors^33^ to MHCII-associated support of CD4⁺ T cell responses in the GL261 and CT2A models^34^, likely reflecting differences in tumor immunogenicity and myeloid cell ontogeny. An important methodological distinction is that prior studies relied on germline or conditional Trem2 deletion, which alters myeloid cell ecology throughout tumor development, whereas the CAR-T cell-based approach used here acutely targets established TREM2⁺ populations within a defined perioperative window. Moreover, our data reveal that timing is a decisive variable: TREM2 targeting in the adjuvant setting enhanced GD2 CAR-T cell activity, whereas neoadjuvant TREM2 depletion unexpectedly attenuated it, consistent with a temporally dynamic model in which TREM2⁺ cells may shift from antigen-presenting to predominantly immunosuppressive states following surgical injury. Compartment-specific targeting of TREM2⁺ populations thus represents an important direction for future investigation.

The TME is increasingly recognized as a key determinant not only of immune suppression but also of tumor antigen expression, with direct consequences for CAR-T cell efficacy. GD2, the target explored in this study and under clinical development for glioma^60^ and other solid tumors^61–63^, is regulated by the ST8SIA1 (GD3 synthase) pathway, whose transcription is sensitive to hypoxia, inflammatory cytokines, and epigenetic cues^64^. Similar plasticity has been described for other GBM CAR-T cell targets. IL13Rα2 expression is reduced under hypoxic conditions, suggesting selective antigen loss in poorly perfused tumor regions^65^, while inflammatory signaling and mesenchymal programs driven by NF-κB and TGF-β promote its expression^66^. EGFRvIII-expressing glioma cells display enhanced survival under hypoxia and metabolic stress, favoring clonal selection in hostile microenvironments rather than uniform expression^67^. B7-H3, another clinically advanced GBM CAR target, is strongly induced by hypoxia-associated lactate accumulation and TGF-β signaling through epigenetic and transcriptional mechanisms^68,69^, reinforcing immune exclusion in the post-surgical niche. Together, these studies indicate that antigen expression in GBM is dynamically shaped by hypoxia, wound-healing cytokines, and metabolic stress, features that are amplified following surgical resection, providing a mechanistic framework by which resection may limit CAR-T cell activity through spatial and temporal antigen heterogeneity.

Even though locally delivered checkpoint inhibitors (anti-PD1, anti-Tim3, or anti-PD-L1) after tumor resection modulated CAR-T cell phenotypes, they failed to improve survival in murine GBM models. A likely explanation is that resection induces a surge of immunosuppressive signals that overwhelm checkpoint blockade. HIF-1α directly transactivates PD-L1 in tumor and myeloid cells, bolstering local immunosuppression^70^. Simultaneously, extracellular matrix remodeling in solid tumors, which is common post-resection^44^, is linked to T cell exclusion and upregulation of inhibitory receptors on tumor-infiltrating lymphocytes^71,72^. Moreover, inflammatory cytokines released during the wound-healing response, particularly IFNγ, are potent inducers of PD-L1 expression across tumor and myeloid cells in GBM^73^. Notably, Khalsa et al.^15^ observed upregulated Tim3 expression in CD8 tumor-infiltrating T cells 4 days after GBM resection in the CT2A model, suggesting that surgery may induce T cell exhaustion via checkpoint upregulation, as we observed in both SB28 and CT2A models. In our model, the early emergence of exhaustion-like phenotypes in T_eff_ cell subsets is most consistent with local reprogramming of pre-existing tumor-infiltrating lymphocytes rather than de novo recruitment from the circulation. The relative enrichment of less-differentiated T cell subsets likely reflects phenotypic dedifferentiation rather than true influx of naive T cells. In vivo, a contribution from circulating T cells cannot be formally excluded, particularly at later timepoints, but the convergence between our murine and human ex vivo data strongly implicates local reprogramming as the dominant mechanism. Critically, early depletion of TREM2⁺ myeloid cells by TREM2 CAR-T cells was sufficient to increase CD3⁺ T cell infiltration and reduce exhaustion-like phenotypes in CD8⁺ T_eff_ cells three days post-resection, providing direct evidence that TREM2⁺ myeloid cells actively contribute to post-surgical T cell dysfunction rather than both effects arising independently from perioperative inflammation.

Finally, our results highlight the critical importance of timing in CAR-T cell therapy for GBM. Neoadjuvant administration preserved T cell function and fostered a more proinflammatory TME, whereas adjuvant infusion of CAR-T cells failed to confer survival benefit. This finding mirrors the advantages observed with neoadjuvant immune checkpoint blockade in GBM. Notably, in an early clinical study of recurrent GBM, neoadjuvant PD1 inhibition increased tumor-infiltrating lymphocyte activation and nearly doubled overall survival compared to the same treatment given post-resection^26^. Similarly, a patient treated with triple immune checkpoint inhibitors before surgery showed strong T cell tumor infiltration and activation^27^. These data suggest that avoiding the immediate post-operative suppressive TME is key for T cell-based strategies in GBM. Our study explores a neoadjuvant CAR-T cell approach in GBM, extending the neoadjuvant paradigm previously established with checkpoint inhibitors to adoptive cell therapy. Neoadjuvant CAR-T cell administration capitalizes on the presence of abundant tumor antigen, potentially triggering broader antigen release and endogenous T cell priming. It could thus not only debulk the tumor but also serve as an “in situ vaccine,” inducing a systemic immune response for long-term tumor control. Importantly, our study also provides insights regarding combination strategies in the neoadjuvant context. Indeed, we observed that TREM2 CAR-T cells, when given as neoadjuvant treatment, unexpectedly blocked the efficacy of GD2-directed CAR-T cells. In this regard, eliminating TREM2⁺ cells might deprive the host of antigen-presenting and phagocytic functions that are central to TREM2 biology. TREM2 signaling promotes lipid clearance, efferocytosis, and phagocytosis of tumor debris via Syk-dependent pathways, supporting sustained antigen uptake and MHCII-restricted presentation^74,75^. Consistent with this, recent work in GBM showed that TREM2⁺ TAM support MHCII-dependent CD4⁺ T cell responses, and that their depletion can impair rather than enhance antitumor immunity in immunogenic contexts^32^. These observations, together with the context-dependence of TREM2 function discussed above, underscore that therapeutic targeting of TREM2⁺ myeloid cells should be carefully timed and combined to avoid dismantling antigen-presenting niches that support endogenous and adoptive T cell responses.

While the survival benefits observed with our combinatorial strategies remain modest in absolute terms, this is consistent with the well-established resistance of the SB28 model to both immune checkpoint blockade and CAR-T cell therapy^21,31,76^, features that closely recapitulate the immunosuppressive landscape of human GBM. These results should therefore be interpreted as proof-of-concept evidence that perioperative myeloid remodeling combined with tumor-targeting CAR-T cells can reshape the post-resection TME and extend survival in a highly stringent preclinical setting, rather than as evidence of curative efficacy. In addition, future studies should validate these findings in GSC-based syngeneic models that more closely recapitulate the invasive growth and stem cell features of human GBM, and in patient-derived xenograft models where feasible. Thus, while neoadjuvant CAR-T cell administration is clearly advantageous, our results caution that the composition of neoadjuvant immunotherapy must be carefully considered. Altogether, our study provides a rationale to translate CAR-T cell therapy into the neoadjuvant setting for GBM, a strategy that should be tested in clinical trials in the future.

## Methods

### Cell lines and modifications

The murine GBM cell lines SB28 (kindly provided by H. Okada, UCSF) and CT2A (commercially available) were used in this study. They were stably engineered to express GFP and firefly luciferase to allow in vivo imaging. Both lines were cultured in DMEM (ThermoFisher) supplemented with 10% fetal bovine serum (FBS, Gibco), 1% L-glutamine (Gibco), and 1% penicillin–streptomycin (ThermoFisher). CT2A and SB28 cells were modified to express GD2 by transduction with lentiviruses encoding GD2S and GD3S synthases (Supplementary Fig. 21), as described previously^77^. GD2^+^ (transduced) cancer cells were sorted using a FACSAria II SORP (Becton Dickinson). Phoenix-Eco cells (ATCC CRL-3214) were used as packaging cells for retroviral vector production and maintained in RPMI 1640 (ThermoFisher) supplemented with 10% FBS, 10 mM HEPES (Gibco), and 1% penicillin–streptomycin. T2 cells (ATCC CRL-1992) were used as indicator cells for retroviral titration. They were cultured in RPMI 1640 supplemented with 10% FBS, 1% L-glutamine, and 1% penicillin–streptomycin. All cell lines were routinely tested for Mycoplasma contamination. Authentication of parental stocks was performed at the time of acquisition, and morphology, growth kinetics, and tumorigenicity remained consistent with original reports throughout the study.

### Generation of BMDM

Total bone marrow cells were extracted from the tibia and femur of C57Bl6/j mice between 7 and 9 weeks old under a flow of 0.1 M PBS (10 ml), pH 7.4, containing 2% FBS. Cells were centrifuged and resuspended in DMEM supplemented with 10% heat-inactivated FBS and 100 ng/ml M-CSF (Miltenyi Biotec) at a density of 3 × 10^5^ cells/mL, seeded onto noncoated 10-cm dishes, and cultured for 5 days in different flasks. The adherent cells are hereafter referred to as BMDM^78^.

### FACS-based phagocytosis assay

BMDM were first pre-exposed to conditioned media from SB28 or CT2A cells for 72 h to induce TREM2 expression. Following this preconditioning, BMDM were co-cultured with either TREM2 CAR-T cells or untransduced T cells at a 3:1 effector-to-target ratio for 72 h. Residual BMDM after T cell treatment were then harvested and immediately co-cultured with SB28-GFP⁺GD2⁺ or CT2A-GFP⁺GD2⁺ tumor cells (6 × 10⁴ BMDM:3 × 10⁴ target cells) for 24 h at 37 °C in triplicate wells. After co-culture, cells were detached with trypsin (Gibco), scratched and stained with anti-F4/80 PE (111604, BioLegend). Samples were acquired on a Cytoflex LX (Beckman Coulter) and analyzed using FlowJo software (FlowJo 10.10.0, LLC). The percentage of GFP⁺ events within the F4/80⁺ population was quantified as the phagocytic index.

### CAR design and production of retroviral vectors

The GD2 CAR was generated using the 14g2a scFv (GenScript)^22^, which was cloned into the pMSGV retroviral backbone in frame with the murine CD8α hinge and transmembrane regions, followed by the intracellular domains of murine CD28 and CD3ζ. The TREM2 CAR shared the same pMSGV backbone, CD8α hinge/transmembrane, and CD28-CD3ζ intracellular signaling domains but incorporated the murine scFv 37012-derived from patent US 10,508,148 B2. Design of both CAR constructs are summarized in Supplementary Table 1. Retroviral particles were generated by transient transfection of Phoenix-Eco packaging cells with the CAR-encoding plasmid together with the pCL-Eco helper plasmid using Turbofect (ThermoFisher). Briefly, 9 × 10^6^ Phoenix-Eco cells were seeded per T150 flask one day before transfection. At ~70–80% confluence, cells were transfected with 21.4 µg of CAR plasmid DNA and 14.3 µg of pCL-Eco in OptiMEM (Thermofisher) using Turbofect according to the manufacturer’s instructions. After overnight incubation, the medium was replaced with fresh Phoenix medium (RPMI, 10% FBS, 10 mM HEPES). Viral supernatants were harvested at 48 h and 72 h, filtered through 0.22 µm membranes, and concentrated by ultracentrifugation at 25,000 rpm for 2–2.5 h at 4 °C. Pellets were resuspended in 0.3–1 mL of cold Phoenix medium, pooled, and aliquoted in 250 µL volumes before storage at −80 °C. Retroviral titers were determined by transducing T2 cells with serial dilutions of viral preparations, followed by flow cytometric quantification of CAR expression after 72 h. Functional titers were calculated as transducing units per mL (TU/mL) and were used to standardize multiplicity of infection (MOI) for subsequent T cell transduction.

### Murine CAR-T cell production

Mouse spleens from CD45.1 or CD45.2 C57BL/6 mice were processed on a 70-µm cell strainer, followed by centrifugation to obtain a single-cell suspension. Red blood cell lysis was done using Qiagen Red Blood Cell Lysis Buffer. Murine T cells were purified using the EasySep Mouse T Cell Isolation Kit (Stemcell Technologies) and were seeded at 10^6^ cells per mL in RPMI supplemented with 10% FBS), 10 mM HEPES, 1 mM sodium pyruvate (Gibco), 1% penicillin-streptomycin, 1× non-essential amino acids (Gibco), 1% l-glutamine, 50 µM β-mercaptoethanol (Gibco) and 50 IU ml–1 rhIL-2 (Bio-Techne). T cells were activated with Activator CD3/CD28 Dynabeads (Gibco) at a ratio of two beads per cell. Retroviral vector transduction was conducted after 24 h in 24 or 48-well plates coated with 20 μg/mL recombinant human fibronectin (Takara Clontech). The medium was replaced on day three with medium containing 10 IU/mL rhIL-2, 10 ng/mL rhIL-7 (PeproTech) and 10 ng/mL rhIL-15 (PeproTech). Cells were passaged every second day. CAR expression was determined on day eight by flow cytometry before T cell collection for downstream experiments.

### CAR-T cell killing assay

To assess killing capacity, the IncuCyte® ZOOM System (Sartorius) was used. BMDM were stained with CellTrace™ Yellow Dye (Thermo Fisher Scientific) and seeded in 96-well plates at 1 × 10^4^ cells per well. After a 2 h adherence period, either untransduced T cells or TREM2 (37012) mouse CAR-T cells were added at effector-to-target (E:T) ratios of 1:1 or 3:1. Cytotoxicity was monitored using the IncuCyte® Cytotox Green Reagent (Sartorius, 4633), which fluoresces upon binding to nucleic acids in membrane-compromised (dead) cells. Plates were immediately placed in the IncuCyte® ZOOM System for live-cell imaging over 72 h. Cytotoxicity was quantified as the percentage of CellTrace-labeled BMDM co-expressing Cytotox Green, reflecting the proportion of tumor-educated macrophages undergoing cell death over time.

### Mice

All animal experiments were performed using 7–8-week-old male C57BL/6 mice (CD45.2, Janvier Labs, France or Charles River, France). For congenic transfer experiments, CD45.1 C57BL/6 mice (Charles River, France) were also used. Mice were housed under specific pathogen-free conditions with ad libitum access to food and water. All procedures were conducted in accordance with institutional guidelines and approved by the Veterinary Authority of the Canton of Vaud (authorization VD4017d).

### Orthotopic GBM mouse model and CAR-T cell injection

For intracranial GBM experiments, 7–8-week-old C57BL/6 mice (Janvier Labs) were implanted intracranially with either SB28 (1.5 × 10^4^) or CT2A (5 × 10^4^) cells using a stereotaxic apparatus (Stoelting). Mice were anesthetized with isoflurane and received subcutaneous injections of 2 µg buprenorphine (Bupaq, Streuli) in 100 µL PBS and 50 µL lidocaine 1% (Streuli) at the incision site before surgery. Cells were resuspended in sterile PBS and injected in a final volume of 3 µL into the right striatum at the following coordinates relative to bregma: +0.5 mm anterior, +2.0 mm lateral, and −3.0 mm ventral from the dura. The injection was performed using a 10 µL Hamilton syringe, and the needle was left in place for 1 min post-injection before slow withdrawal to minimize reflux. The burr hole was sealed with surgical adhesive (glue) and the skin closed. For neoadjuvant CAR-T cell therapy, 1 × 10^6^ untransduced or CAR-positive T cells in 5 µL PBS were injected stereotaxically into the same coordinates on day 7 after tumor implantation, using identical anesthesia, analgesia, and injection procedures as above. Mice were monitored until full recovery and then returned to their cages. Tumor burden was assessed at defined time points by in vivo bioluminescence imaging (IVIS, PerkinElmer), and animals were randomized to treatment groups based on tumor signal intensity. Mice were observed three times per week and euthanized upon reaching predefined humane endpoints (≥20% body weight loss, impaired mobility, hunched posture, or lethargy).

### Tumor resection model

From 10 to 12 days after tumor implantation, mice were anesthetized with isoflurane and received a subcutaneous injection of extended-release buprenorphine (Ethiqa XR, Fidelis, 3.25 mg/kg) for analgesia. Animals were positioned prone in a stereotaxic frame, and the previous incision was extended to a length of 15–20 mm. A craniectomy was performed using a 3 mm diameter trephine, centered on the prior burr hole. Following cortico-dural coagulation, macroscopically pathological tissue was removed by aspiration using a diaphragm vacuum pump. Hemostasis was achieved with Surgicel hemostatic gauze (Ethicon) followed by irrigation with sterile physiological saline. Encapgel (Sigma-Aldrich, 922412-1EA), containing either vehicle, 10^6^ untransduced or CAR-positive T cells and/or GoInVivo^TM^ purified mouse anti-PD1 (25 µg/mice, clone 29 F.1A12, rat IgG2a, Biolegend, 135235,), anti-Tim3 (25 µg/mice, clone RMT3-23, rat IgG2a, Biolegend, 119713), anti-CD274 (50 µg/mice, clone 10 F.9G2, IgG2b, Biolegend, 124329) or InVivoMAb anti-mouse Ly6G/Ly6C (Gr-1) (30 µg/mice, clone RB6-8C5, rat IgG2b, BioXCell, BE0075) blocking antibodies or isotype controls (Rat IgG2b κ, Biolegend, 400366; Rat IgG2b κ, Biolegend, 119712, depending on the blocking antibody administered) in 5 to 10 µL, was injected directly into the resection cavity.

### Organotypic slices from human GBM

To determine the effects of resection on human GBM tissue, we studied GBM sections obtained directly after resection. This study was approved by the Human Research Ethics Committee from Geneva, Switzerland (protocols 2022-02109 and 2023-01928) and written informed consent was obtained from all patients prior to tissue collection. GBM specimen obtained directly after resection were transferred from the operating room to the laboratory in a 50-mL conical tube containing ice-cold medium (high-glucose DMEM supplemented with 10% human serum, HEPES 1× and 1× penicillin–streptomycin solution). Pieces were punched from the tumor, embedded into 2% agarose, then sliced on a vibratome to a thickness of 400 µm. GBM slices were cultured on a polytetrafluoroethylene insert (pore size 0.4 µm) in 24-well plates with culture medium (neurobasal medium supplemented with B27 1×, D-glucose 1.5 mg/mL, Glutamax 600 µM, l-glutamine 400 µM and 1× penicillin–streptomycin solution). Tumor slices (*n* = 2–3 per patient) were cultured overnight, and the “ex vivo” resection was performed by punching the middle of the tissue with a pipette tip. GBM slices were kept from 24 to 72 h and were then fixed in paraformaldehyde 4% for immunohistofluorescence or dissociated for flow cytometry by using brain tumor dissociation kit (Miltenyi, 130-095-942).

### Immunohistofluorescence

Mice were deeply anesthetized with a ketamine/xylazine cocktail (100 mg/kg and 12.5 mg/kg, i.p.) and perfused transcardially with 15 mL ice-cold PBS followed by 25 mL of 4% paraformaldehyde (PFA). Brains were post-fixed overnight in 4% PFA at 4 °C, cryoprotected in 30% sucrose for 24 h at 4 °C and stored in PBS until processing. Free-floating coronal sections (50 µm) were cut on a vibratome (VT1200S, Leica Biosystems). Sections were permeabilized in PBS 1× containing 0.5% Triton X-100 (10717503, Invitrogen) and blocked for 1 h at room temperature in PBS 1× supplemented with 5% normal donkey serum and 10% BSA. Antibodies (see Reporting Summary and Supplementary Table 2) were diluted in the same blocking solution and incubated with sections overnight at 4 °C. After thorough PBS washes, sections were counterstained with DAPI (5 min; 28718-90-3, Sigma-Aldrich), and mounted in Fluoromount with DAPI (00-4959-52, Thermofisher). Images were acquired on a Nikon Ti2 Yokogawa spinning disk confocal microscope at the Cellular Imaging Facility (CIF, AGORA Cancer Research Center, Lausanne), using identical laser, gain, and exposure settings across samples. Z-stacks were reconstructed and processed using FIJI. Quantitative analyses, including TREM2⁺ cell density (as % of DAPI⁺ nuclei) and nearest-neighbor distance to GFP⁺ tumor cells, were performed using a custom R script that processed spatial coordinates exported from FIJI to compute cell-to-cell distances and generate synthetic spatial distribution maps of TREM2⁺ and GFP⁺ cells. All image analyses were performed blinded to experimental condition. To assess tumor invasiveness, fractal dimension analysis was performed on GFP⁺ tumor using the box-counting method implemented in FIJI, as previously described^79^. Briefly, binary masks of GFP⁺ tumor regions were generated by thresholding, and the fractal dimension (D value) of the tumor was calculated to quantify morphological complexity as a proxy for invasive capacity. Tumor circularity was additionally computed from the same binary masks using the standard FIJI shape descriptor (circularity = 4π × area/perimeter²).

### Flow cytometry

For both human and mice, samples were dissociated by using the brain tumor dissociation kit (Miltenyi, 130-095-942) in combination with the gentleMACS Octo Dissociator (Miltenyi, 130-134-029) after myelin removal with Percoll 30% gradient. For human T cell analysis, CD3 positive cells were isolated by using the easySep™ human T Cell isolation kit (Stemcell, # 17951). For mice brain tumor, single-cell suspensions were incubated in PBS with purified anti-mouse CD16/32 Fc block (1:100; clone 93, Rat IgG2a, Biolegend, 101301) and fixable live–dead colors and stained with antibodies (See Reporting Summary and Supplementary Table 2) before acquisition by flow cytometry. Stained samples were analyzed with the Cytoflex LX (Beckman Coulter). Analysis of flow cytometry data used FlowJo v10.1. Immune cell populations were identified as indicated in the Reporting Summary and Supplementary Figs. 22–24. Briefly, myeloid cell populations were identified within the CD11b^+^ CD45⁺ gate as follows: microglia (CD45^low^ F4/80⁺ CD11b^+^), TAM (CD45^high^ F4/80⁺ CD11b^+^), and monocytes (CD45^high^ F4/80⁻ CD11b^+^ Ly6C⁺ Ly6G⁻) were each further subdivided into CD206⁻MHCII⁻, CD206⁺MHCII^⁻/+^ (named as CD206^+^ throughout), and CD206^-^MHCII⁺ subsets. Of note, the CD45^high^ TAM gate comprises both activated microglia, which upregulate CD45 upon tumor-induced activation and monocyte-derived macrophages, which cannot be reliably discriminated by conventional flow cytometry in the GBM context^8,48^. Neutrophils were defined as CD45^high^ CD11b⁺ Ly6G^high^ Ly6C^high^. Conventional DC were identified as cDC1 (CD11c⁺ MHCII⁺ XCR1⁺) and cDC2 (CD11c⁺ MHCII⁺ XCR1^-^). Lymphoid cell populations were gated as follows: NKT cells (CD4⁺/CD8 + NK1.1⁺), CD4⁺ T cells (CD8^-^ CD4⁺ NK1.1^-^) and CD8⁺ T cells (CD4^-^ CD8⁺ NK1.1^-^), each subdivided into T_naive_ (CD44^-^CD62L⁺), T_cm_ (CD44⁺CD62L⁺) and T_eff_ (CD44⁺CD62L^-^) subsets; NK cells (CD4^-^ CD8^-^ NK1.1⁺). A complete description of all marker panels and gating hierarchy is provided in Supplementary Tables 3 and 4. TREM2 positivity thresholds were defined using Fluorescence Minus One (FMO) controls for each myeloid cell subset independently, as illustrated in Supplementary Fig. 1d. Following clustering into immune cell subsets, compensated and transformed data were exported from FlowJo and subjected to uniform manifold approximation and projection (UMAP) analysis using the FlowJo plugin for visualization. UMAP plots were generated to illustrate the phenotypic relationships among manually defined immune cell populations within each experimental group. To quantify T cell exhaustion-like phenotypes, we derived a composite score based on exhaustion-associated markers (Ex score) within both CD4⁺ and CD8⁺ T cell subsets using the following formula: Ex score = 0.5 × (% PD-1⁺Tim3⁺ double-positive) + 0.3 × (MFI Tim3) + 0.2 × (MFI PD-1). This weighted metric integrates both the frequency of co-inhibitory receptor co-expression and the relative intensity of PD-1 and Tim3, providing a continuous parameter for cross-sample comparison of exhaustion states in each T cell compartment.

### RNA extraction and qPCR

Total RNA was extracted from BMDM exposed or not to conditioned media from SB28 or CT2A cells for 72 h using the RNeasy Mini Kit (Qiagen) according to the manufacturer’s instructions. RNA concentration and purity were measured using a Nanodrop spectrophotometer (ThermoFisher Scientific). Complementary DNA (cDNA) was synthesized from 500 ng of total RNA using the iScript cDNA Synthesis Kit (Bio-Rad). Quantitative PCR (qPCR) was performed with SYBR Green PCR Master Mix (Applied Biosystems) on a QuantStudio™ 6 Flex Real-Time PCR System (Applied Biosystems). Relative mRNA expression levels were calculated using the ΔΔCt method with mEEf1a as the housekeeping gene. All reactions were performed in technical triplicates. Primer sequences are provided in Supplementary Table 5.

### scRNAseq database analysis

Publicly available scRNA-seq datasets were obtained from three sources. CT2A data were retrieved from Zenodo (https://doi.org/10.5281/zenodo.6654420), as originally published by Tomaszewski et al.^80^ SB28 datasets (GSM8703639 and GSM8703640) were downloaded from the Gene Expression Omnibus (GEO), as described in Ugolini et al.^81^. Human GBM immune data, originally published by Pombo Antunes et al.^8^, were retrieved from the Brain Immune Atlas (https://www.brainimmuneatlas.org/). All analyses were performed in R (v4.5.1; R Foundation for Statistical Computing) using Seurat (v5.4.0). Data were processed following a standard workflow including quality control filtering, normalization (SCTransform), dimensionality reduction (PCA and UMAP), and graph-based clustering. Cell-type annotation was performed using differential gene expression analysis (FindAllMarkers function) integrated with original annotations from source publications where available. Myeloid cell populations were annotated using curated marker gene signatures: microglia (P2ry12, Ccl4, Tmem119, Igf1), tumor-associated macrophages (Cd68, Spp1, Mrc1), monocytes (Plac8, Chil3, Ccr2, Ly6c2), neutrophils (S100a8, S100a9), DC (Ccl22, Ccr7, Fscn1, Batf3, Cd36, Flt3, Ffar2, Clec10a), and plasmacytoid DC (Siglech, Ccr9, Bcl11a, Runx2). Gene expression distributions across annotated myeloid cell populations were visualized using violin plots (VlnPlot).

### Statistics and reproducibility

Statistical analysis was performed using GraphPad Prism 10 (GraphPad Software Inc). Data are presented as mean ± SEM, unless otherwise indicated. Two-tailed unpaired Student’s t-tests were employed for comparisons of two groups, while one- or two- way ANOVA with a Bonferroni’s multiple-comparisons test was used for multiple-group comparisons. Correlations were assessed using Pearson’s correlation (two-sided), with r and corresponding P values reported. Survival data were analyzed using the Kaplan–Meier method. For two-group comparisons, the log-rank (Mantel-Cox) test was used. For experiments with more than two groups, pre-specified pairwise log-rank tests were performed between biologically relevant comparisons and exact p-values are reported in the Raw Data Excel file.

### Reporting summary

Further information on research design is available in the Nature Portfolio Reporting Summary linked to this article.

## Supplementary information

Supplementary Information

Reporting Summary

Transparent Peer Review file

## Source data

Source Data

## Data Availability

The CT2A scRNA-seq data used in this study, originally published by Tomaszewski et al.^80^ are available in the Zenodo database under https://doi.org/10.5281/zenodo.6654420. The SB28 scRNA-seq data, originally published by Ugolini et al.^81^ are available in the Gene Expression Omnibus (GEO) database under accession codes GSM8703639 and GSM8703640. The human GBM immune scRNA-seq data, originally published by Pombo Antunes et al.^8^ are available in the Brain Immune Atlas [https://www.brainimmuneatlas.org/]. The remaining data supporting the results in this study are available within the paper and the Supplementary Information and/or are available from the corresponding author upon request. Source data are provided with this paper.

## Code availability

The custom R script used for analysis of the immunohistofluorescence data is available from https://github.com/pedarmar-git/TREM2_GFP_analysis and archived on Zenodo (https://doi.org/10.5281/zenodo.21097173).
