# Supplementary material for: Perioperative myeloid cell remodeling shapes CAR-T cell efficacy in glioblastoma
Source: Nat Commun. 2026 Jul 31;17:9727. doi: 10.1038/s41467-026-76206-y (PMC13562681; doi:10.1038/s41467-026-76206-y)
Supplement: Supplementary file 1 [file 41467_2026_76206_MOESM1_ESM.pdf]

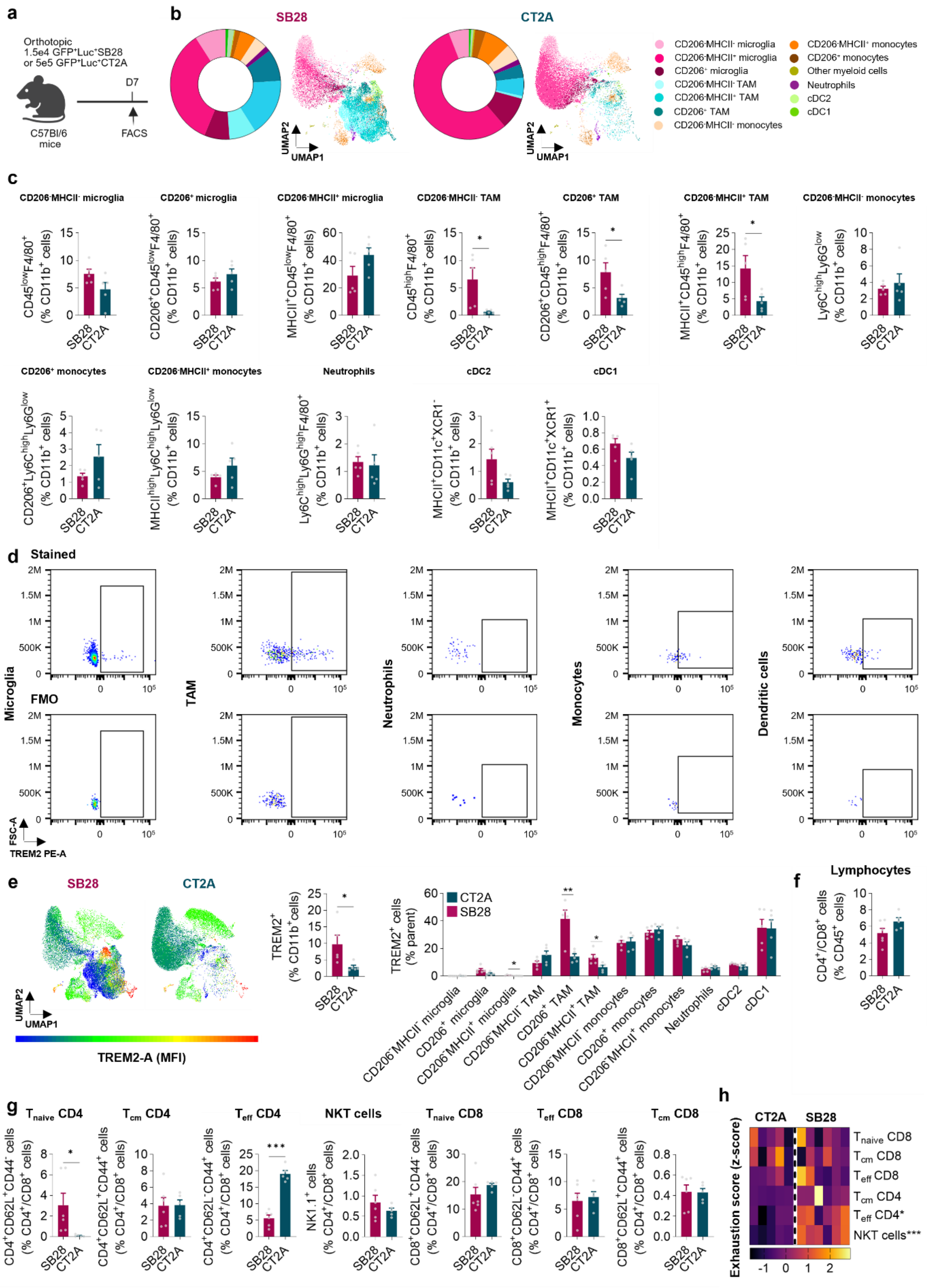

Supplementary figure 1. Baseline immune landscape of SB28 and CT2A tumors prior to resection. (**a**) Experimental scheme comparing orthotopic CT2A and SB28 tumors at day 7 (D7) by flow cytometry. Created in BioRender. Nicola, C. (2026) https://BioRender.com/6o79xto. (**b**) Representative uniform manifold approximation and projection (UMAP) visualization of flow cytometry analysis of CD45^{+}CD11b^{+} myeloid cell

populations and donut charts showing the distribution of major myeloid cell subsets in CT2A and SB28 tumors at D7 (n=5/group). (**c**) Frequency of microglia, tumor-associated macrophage (TAM), monocyte, neutrophil, and dendritic cell (cDC2 and cDC1) subsets in CT2A vs SB28 tumors, shown as percentage of $CD11b^+$ cells from one experiment (Unpaired two-tailed t-test; n=5 mice per group). (**d**) Representative flow cytometry plots showing TREM2-stained samples controls (top row) and Fluorescence Minus One (FMO) (bottom row) for each indicated myeloid cell subset. Gates defining $TREM2^+$ populations were set based on FMO controls. (**e**) UMAP visualization of flow cytometry analysis of $CD45^+CD11b^+$ myeloid cell population colored by TREM2 expression intensity in SB28 and CT2A tumors (left panel). Frequency of $TREM2^+$ cells among total $CD11b^+$ myeloid cells in SB28 and CT2A tumors, and distribution of TREM2 expression across individual myeloid cell subsets (right panel) (Unpaired two-tailed t-test; n=5 mice per group). (**f**) Frequency of total $CD4^+$/$CD8^+$ lymphocytes among $CD45^+$ cells in SB28 and CT2A tumors. (**g**) Frequency of lymphoid cell subsets, including $CD4^+$ and $CD8^+$ naïve (T_{naive}), central memory (T_{cm}), and effector (T_{eff}) populations, as well NKT cells, expressed as percentages of $CD4^+$/$CD8^+$ in SB28 and CT2A tumors at D7 from one experiment (Unpaired two-tailed t-test; n=5 for CT2A and 6 for SB28). (**h**) Heatmap showing exhaustion-like scores (z-scores; see Methods) for indicated lymphoid cell subsets in SB28 and CT2A tumors at D7. Data are shown as mean ± SEM with individual data points representing mice (Unpaired two-tailed t-test; n=5 for CT2A and 6 for SB28). Statistical analyses were performed using unpaired two-tailed t-tests. *$p < 0.05$, **$p < 0.01$, ***$p < 0.001$. Source data and exact p values are provided as a Source Data file.

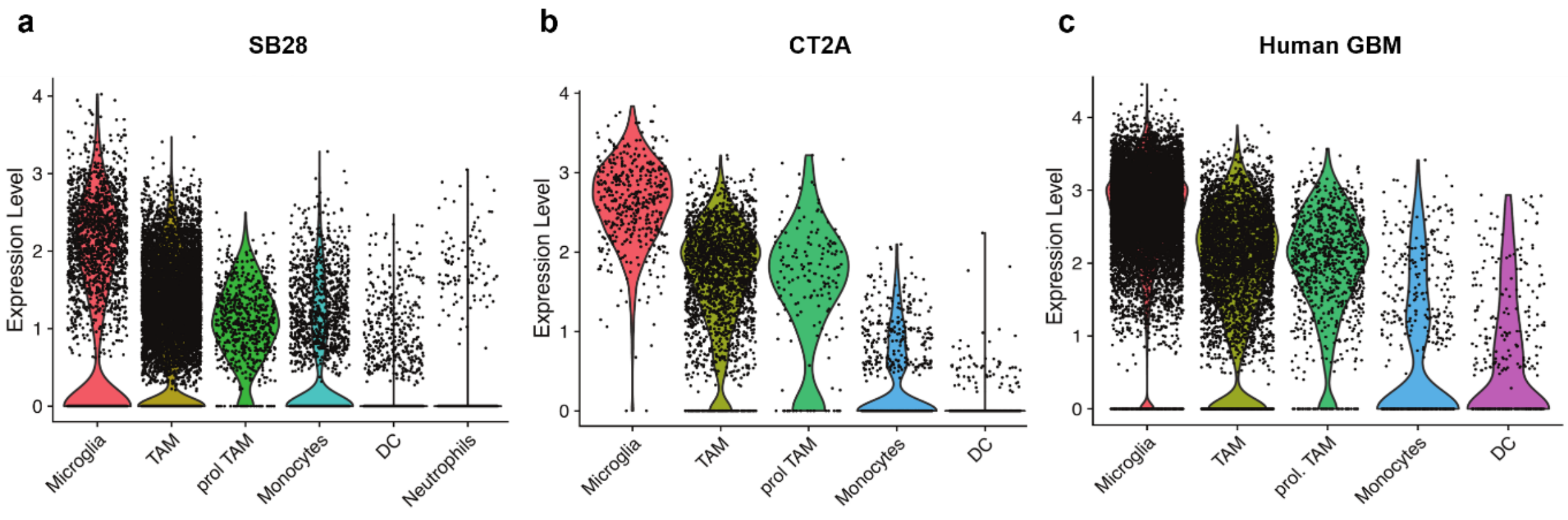

Supplementary figure 2. TREM2 expression across myeloid cell subsets in murine and human GBM by single-cell transcriptomic analysis. Violin plots showing TREM2 (murine or human) transcript expression levels across annotated myeloid cell populations in publicly available scRNAseq datasets from SB28 (**a**), CT2A (**b**), and human GBM (**c**) tumors. Each dot represents an individual cell. In SB28 (a), n = 3,960 cells (microglia n = 434, TAM n = 2,256, monocytes n = 661, DC n = 609). In CT2A (b), n = 15,673 cells (microglia n = 2,451, TAM n = 7,978, monocytes n = 2,214, DC n = 1,684, neutrophils n = 1,346). In human GBM (c), n = 18,639 cells (microglia n = 12,226, TAM n = 4,651, proliferating TAM n = 911, monocytes n = 515, DC n = 336). Populations shown include microglia, tumor-associated macrophages (TAM), proliferating TAM (prol TAM), monocytes, dendritic cells (DC), and neutrophils (for SB28 only).

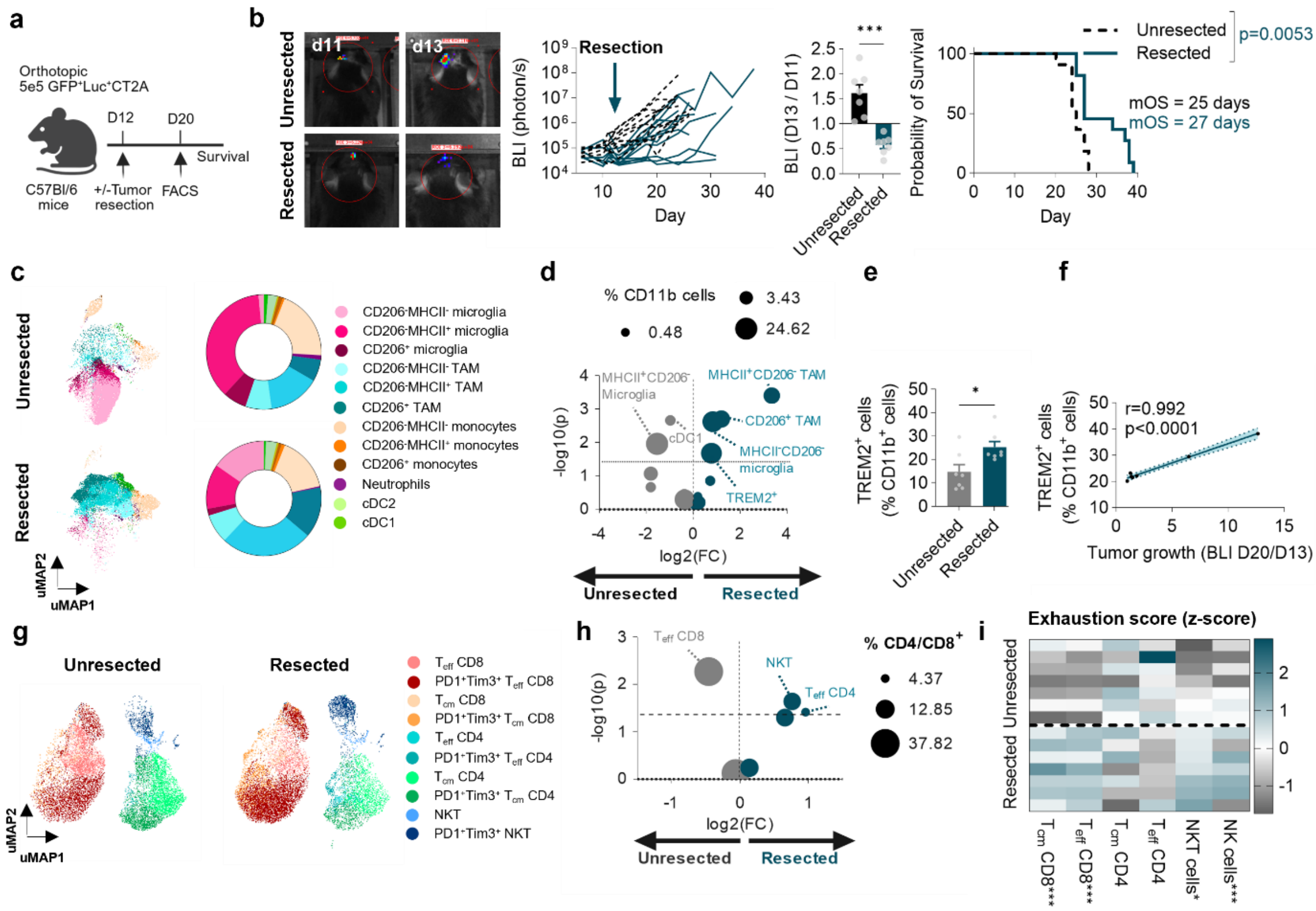

Supplementary figure 3. Tumor resection induces delayed myeloid and lymphoid cell remodeling and TREM2 upregulation in orthotopic CT2A model. (**a**) Experimental scheme comparing unresected and resected orthotopic CT2A tumors at day 20 (D20) by flow cytometry. Created in BioRender. Nicola, C. (2026) https://BioRender.com/6o79xto. (**b**) Left to right: representative bioluminescence imaging (BLI) at days 11 and 13 post-implantation, longitudinal BLI quantification, comparison of BLI signal between days 13 and 11 (Unpaired two-tailed t-test; n=7/group), and Kaplan-Meier survival analysis of unresected and resected mice pooled from two independent experiments (n=11 mice/condition; log-rank test). mOS=median overall survival. (**c**) Representative uniform manifold approximation and projection (UMAP) visualization of flow cytometry analysis of $CD45^{+}CD11b^{+}$ myeloid cell populations and donut charts showing the distribution of major myeloid cell subsets in unresected and resected CT2A tumors (n=7/group). (**d**) Volcano plot showing differential abundance of myeloid cell populations between unresected and resected CT2A tumors; dot size reflects mean population frequency (Unpaired two-tailed t-test, n=7/group). (**e**) Frequency of $TREM2^{+}$ cells among $CD11b^{+}$ myeloid cells in unresected and resected CT2A tumors from one experiment (Unpaired two-tailed t-test, n=7 mice/group). (**f**) Pearson correlation between $TREM2^{+}$ myeloid cell frequency and tumor growth (BLI ratio D20/D13) in the CT2A model; shaded area indicates 95% confidence interval (n=7 mice). (**g**) Representative UMAP visualization of $CD45^{+}$ lymphoid cell populations in unresected and resected CT2A tumors. (**h**) Volcano plot of differential lymphoid cell population abundance between unresected and resected CT2A tumors (Unpaired two-tailed t-test, n=7/group). (**i**) Heatmaps showing exhaustion-like scores (z-scores; see Methods) for indicated lymphoid cell subsets in unresected and resected tumors in CT2A model from one experiment (Unpaired two-tailed t-test, n=7 mice per group). Data are shown as mean ± SEM unless indicated. *$p < 0.05$, ***$p < 0.001$. Source data and exact p values are provided as a Source Data file.

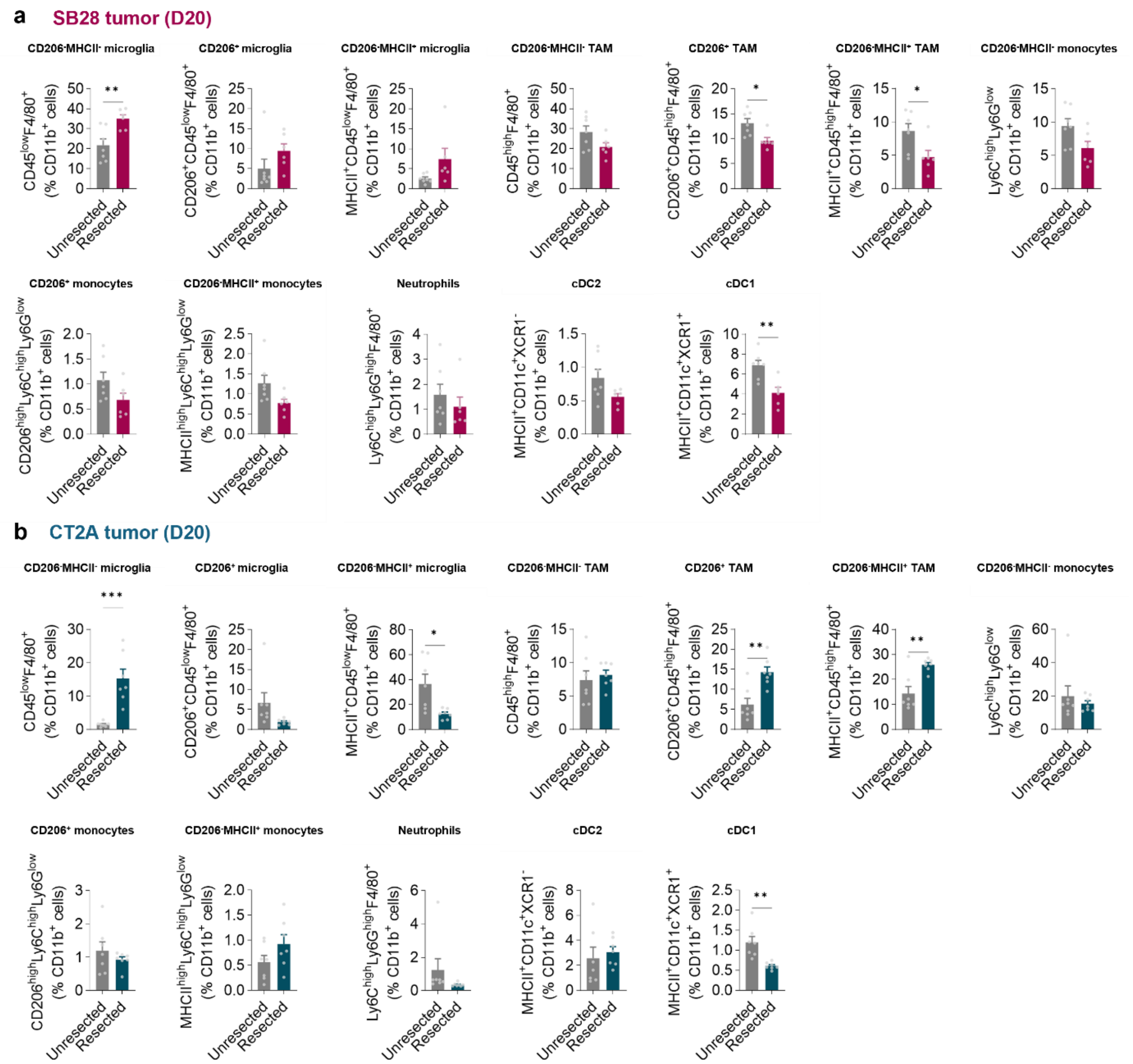

Supplementary figure 4. Tumor resection induces delayed myeloid cell remodeling in orthotopic GBM models. (**a**) Frequency of microglia, tumor-associated macrophage (TAM), monocyte, neutrophil and dendritic cell (cDC1 and cDC2) subsets in unresected vs resected SB28 tumors, shown as percentage of $CD11b^+$ cells. (**b**) Frequency of microglia, tumor-associated macrophage (TAM), monocyte, neutrophil and dendritic cell (cDC2 and cDC1) subsets in unresected vs resected CT2A tumors, shown as percentage of $CD11b^+$ cells. Data are shown as mean ± SEM with individual data points representing mice from one experiment (n=6 for SB28 resected group and n=7 for other groups). Statistical analyses were performed using unpaired two-tailed t-tests. $^*p < 0.05$, $^{**}p < 0.01$, $^{***}p < 0.001$. Source data and exact p values are provided as a Source Data file.

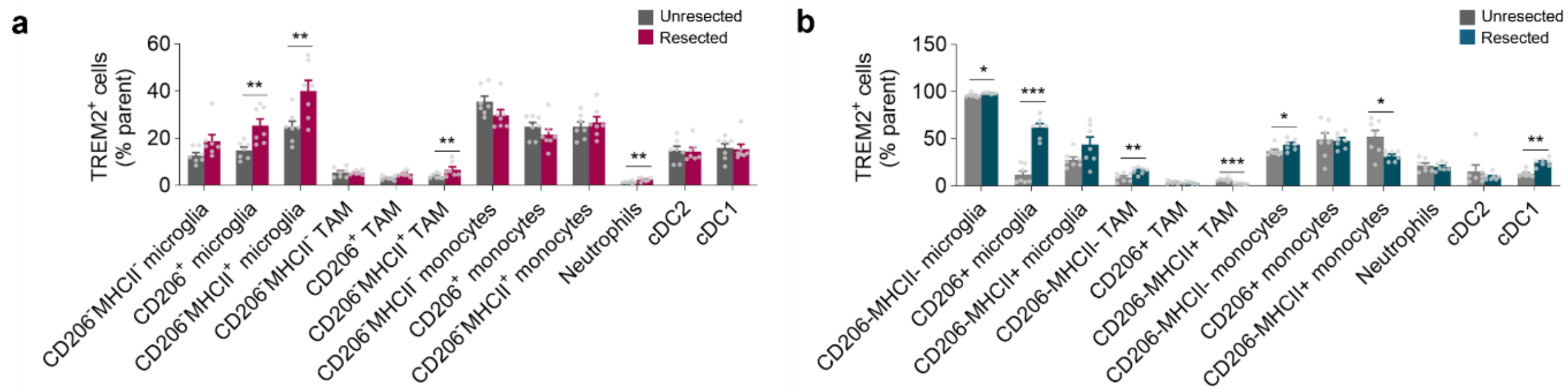

Supplementary figure 5. Tumor resection induces TREM2 upregulation in orthotopic GBM models. (**a**) Distribution of $TREM2^+$ cells across individual myeloid cell subsets in SB28 tumors at day 20. (**b**) Distribution of $TREM2^+$ cells across individual myeloid cell subsets in CT2A tumors at day 20. Data are shown as mean ± SEM with individual data points representing mice from one experiment (n=6 for SB28 resected group and n=7 for other groups). Statistical analyses were performed using unpaired two-tailed t-tests. *p < 0.05, **p < 0.01, ***p < 0.001. Source data and exact p values are provided as a Source Data file.

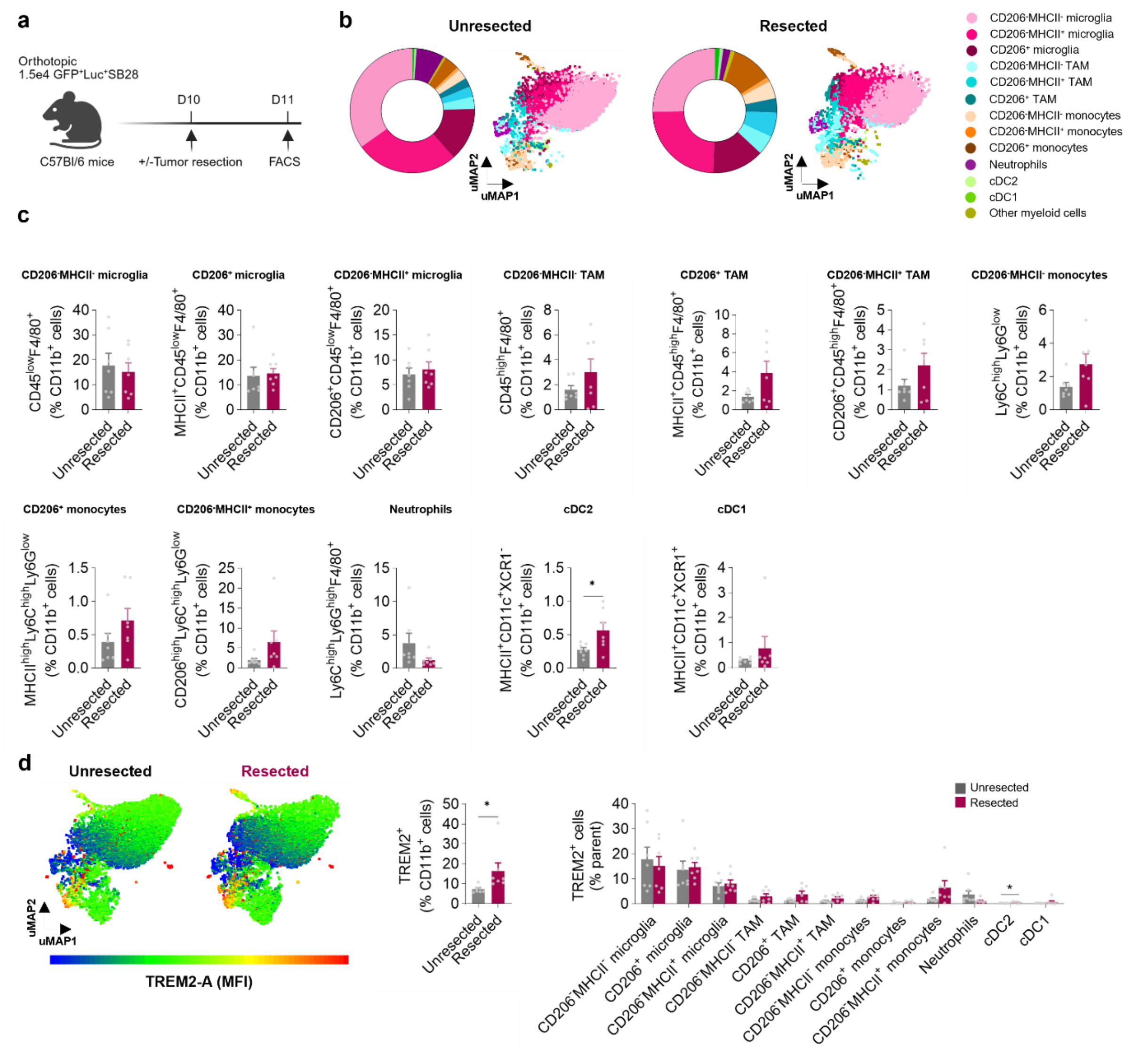

Supplementary figure 6. Tumor resection induces acute myeloid cell remodeling and TREM2 upregulation in the orthotopic SB28 model. (**a**) Experimental scheme comparing unresected and resected orthotopic SB28 tumors on TME at day 11 (D11) by flow cytometry. Created in BioRender. Nicola, C. (2026) https://BioRender.com/6o79xto. (**b**) Uniform manifold approximation and projection (UMAP) visualization of flow cytometry analysis of CD45⁺CD11b⁺ myeloid cell populations and donut charts showing the distribution of major myeloid cell subsets in unresected and resected SB28 tumors. (**c**) Frequency of microglia, tumor-associated macrophage (TAM), monocyte, neutrophil and dendritic cell (cDC2 and cDC1) subsets in unresected vs resected SB28 tumors at D11, shown as percentage of CD11b⁺ cells. (**d**) UMAP visualization of flow cytometry analysis of CD45⁺CD11b⁺ myeloid cell populations colored by TREM2 expression intensity in unresected vs resected SB28 tumors at D11 (left panel). Frequency of TREM2⁺ cells among total CD11b⁺ myeloid cells in unresected vs resected SB28 tumors at D11, and distribution of TREM2 expression across individual myeloid cell subsets (right panel). Data are shown as mean ± SEM with individual data points representing mice from one experiment (n=7 per group). Statistical analyses were performed using unpaired two-tailed t-tests. *$p < 0.05$, **$p < 0.01$, ***$p < 0.001$. Source data and exact p values are provided as a Source Data file.

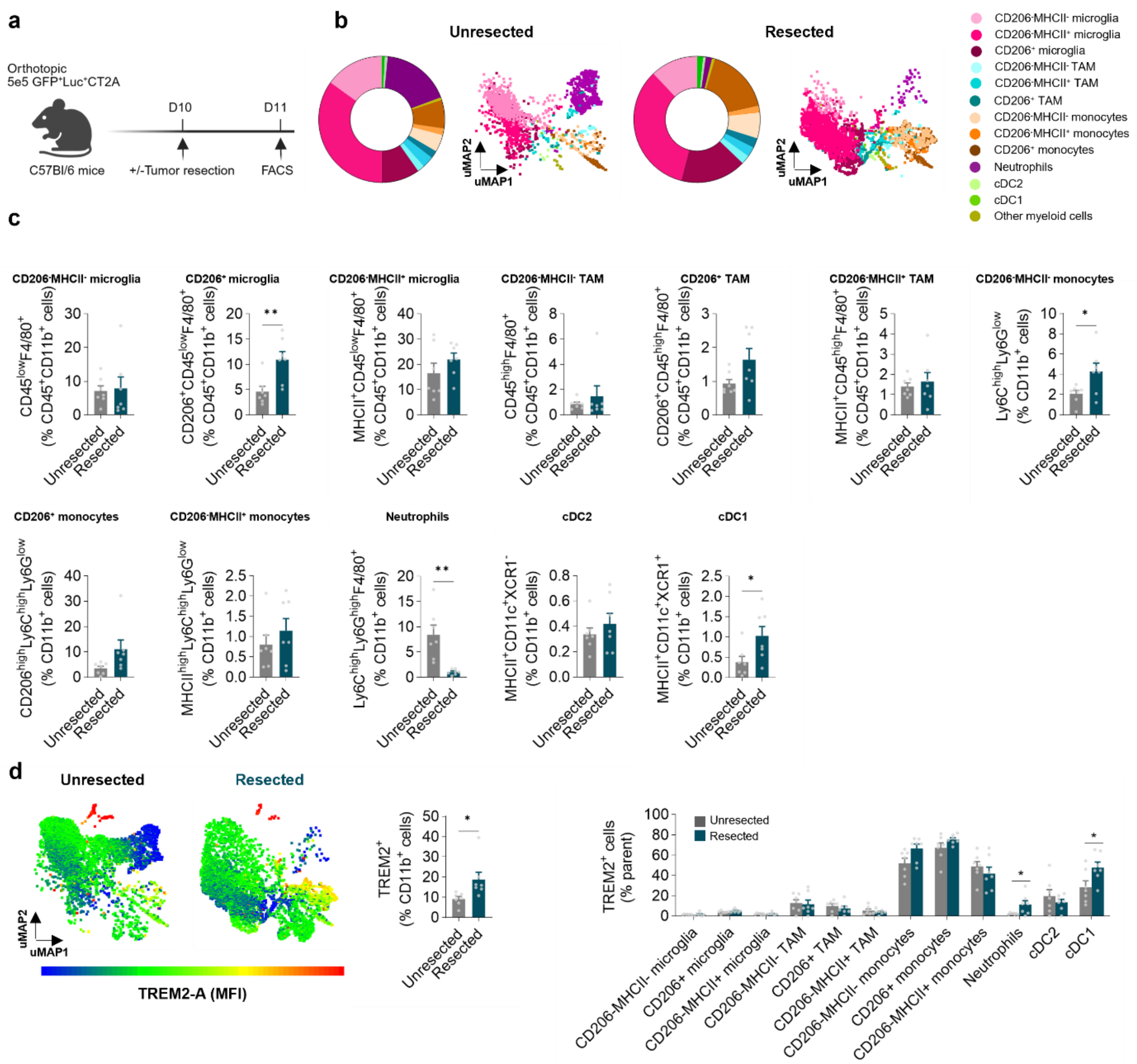

Supplementary figure 7. Tumor resection induces acute myeloid cell remodeling and TREM2 upregulation in the orthotopic CT2A model. (**a**) Experimental scheme comparing unresected and resected orthotopic CT2A tumors at day 11 (D11) by flow cytometry. Created in BioRender. Nicola, C. (2026) https://BioRender.com/6o79xto. (**b**) Uniform manifold approximation and projection (UMAP) visualization of flow cytometry analysis of $CD45^+CD11b^+$ myeloid cell populations and donut charts showing the distribution of major myeloid cell subsets in unresected and resected CT2A tumors. (**c**) Frequency of microglia, tumor-associated macrophage (TAM), monocyte, neutrophil and dendritic cell (cDC2 and cDC1) subsets in unresected vs resected CT2A tumors at D11, shown as percentage of $CD11b^+$ cells. (**d**) UMAP visualization of flow cytometry analysis of $CD45^+CD11b^+$ myeloid cell populations colored by TREM2 expression intensity in unresected vs resected CT2A tumors at D11 (left panel). Frequency of $TREM2^+$ cells among total $CD11b^+$ myeloid cells in unresected vs resected CT2A tumors at D11, and distribution of TREM2 expression across individual myeloid cell subsets (right panel). Data are shown as mean ± SEM with individual data points representing mice from one experiment (n=7 per group). Statistical analyses were performed using unpaired two-tailed t-tests. *$p < 0.05$, **$p < 0.01$, ***$p < 0.001$. Source data and exact p values are provided as a Source Data file.

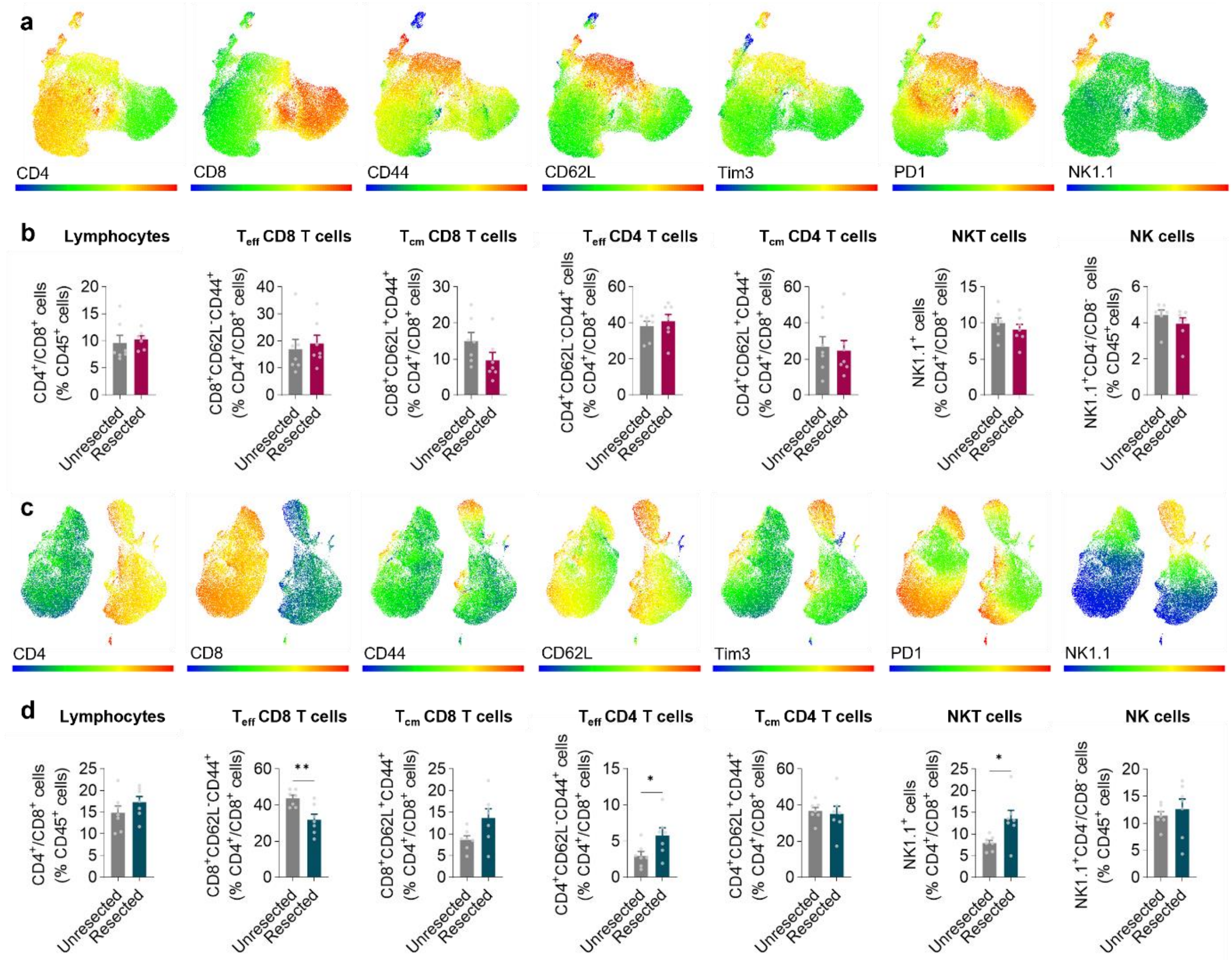

Supplementary figure 8. Expression intensity of T cell markers ten days after resection in SB28 and CT2A tumors. (**a**/**c**) Uniform manifold approximation and projection (UMAP) visualization of lymphoid cell populations gated as $CD45^+CD3^+$ cells from SB28 (**a**) and CT2A (**c**) tumors. Each dot represents an individual cell colored according to expression intensity of indicated markers (CD4, CD8, CD44, CD62L, Tim3, PD1 and NK1.1). **(c)** Quantification of total lymphocytes, T effector (T_{eff}) and central memory (T_{cm}) CD4, T_{eff} and T_{cm} CD8, NKT and NK cells in unresected vs resected SB28 (**b**) and CT2A (**d**) tumors. Data are shown as mean ± SEM with individual data points representing mice from one experiment (n=7 per group). Statistical analyses were performed using unpaired two-tailed t-tests. $*p < 0.05$, $**p < 0.01$. Source data and exact p values are provided as a Source Data file.

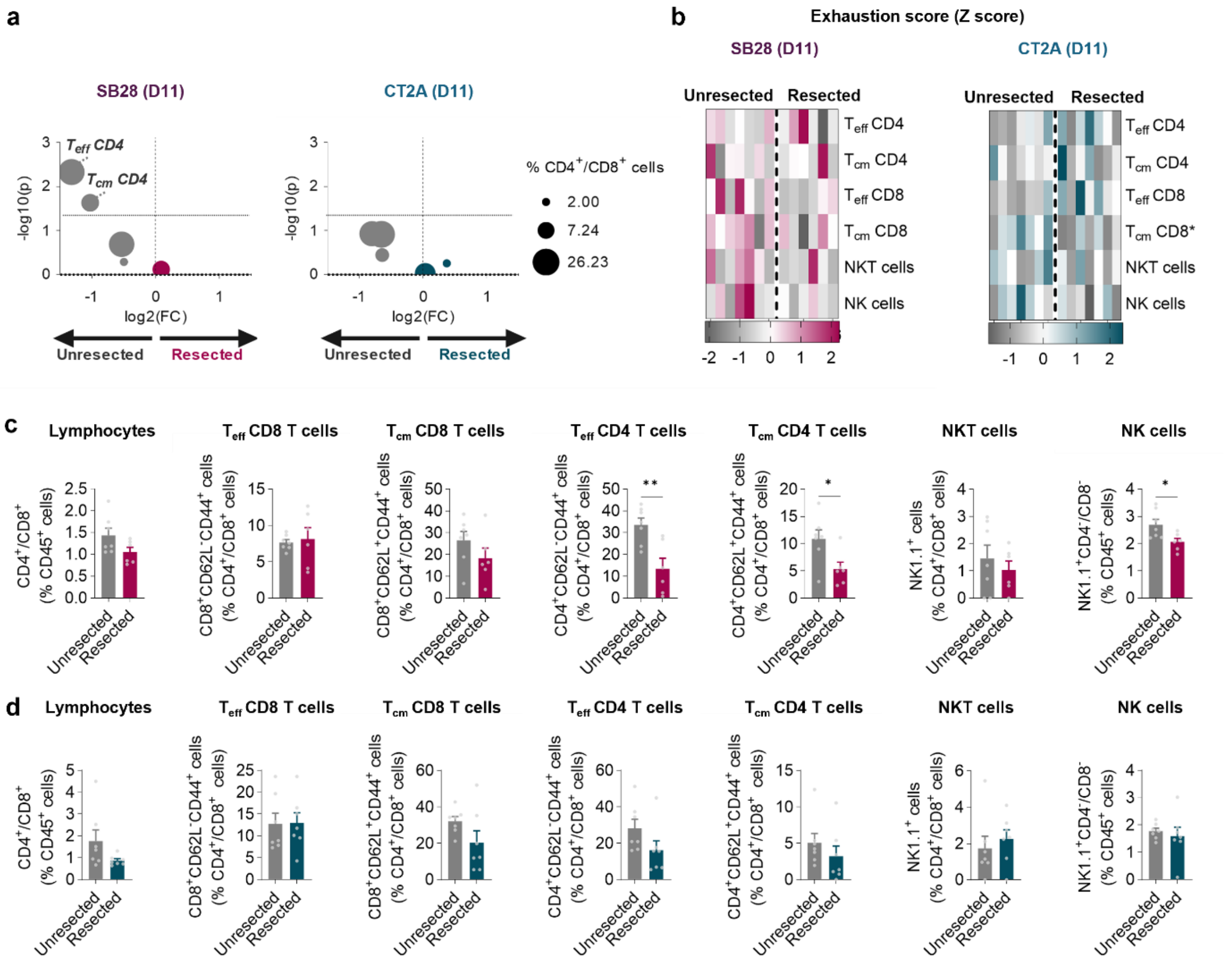

Supplementary Figure 9. Quantification of lymphoid cell populations and exhaustion-like profiles 24 hours after tumor resection in SB28 and CT2A models. (**a**) Volcano plot depicting differences in immune cell population abundance between unresected and resected tumors in SB28 (magenta) or CT2A (dark blue) models, as determined by high-dimensional flow cytometry. The x-axis shows the $\log_2$ fold-change (log2FC) in cell frequency, with positive values indicating enrichment in resected tumors (magenta or dark blue) and negative values indicating enrichment in unresected tumors (grey). The y-axis shows the -log10(p). Circle size is proportional to the mean frequency of each population across samples (n=6 for SB28 resected group and n=7 for other groups). (**b**) Heatmaps showing exhaustion-like scores expressed as z-scores (see Methods) for the indicated lymphoid cell subsets in unresected and resected tumors at day 20 post-implantation in SB28 (left panel) and CT2A (right panel) models. T_{cm}, central memory T cells; T_{eff}, effector T cells (n=6 for SB28 resected group and n=7 for other groups). (**c-d**) Frequencies of T_{eff} CD8, T_{cm} CD8, T_{eff} CD4, T_{cm} CD4, NK1.1^{+} cells, and NKT cells among CD45^{+} cells in unresected *versus* resected SB28 (c) and CT2A (**d**) tumors 24 h post-resection from one experiment (n=6 for SB28 resected group and n=7 for other groups, unpaired two-tailed t-test). Statistical significance was determined using unpaired two-tailed t-tests. *$p < 0.05$, **$p < 0.01$. Source data and exact p values are provided as a Source Data file.

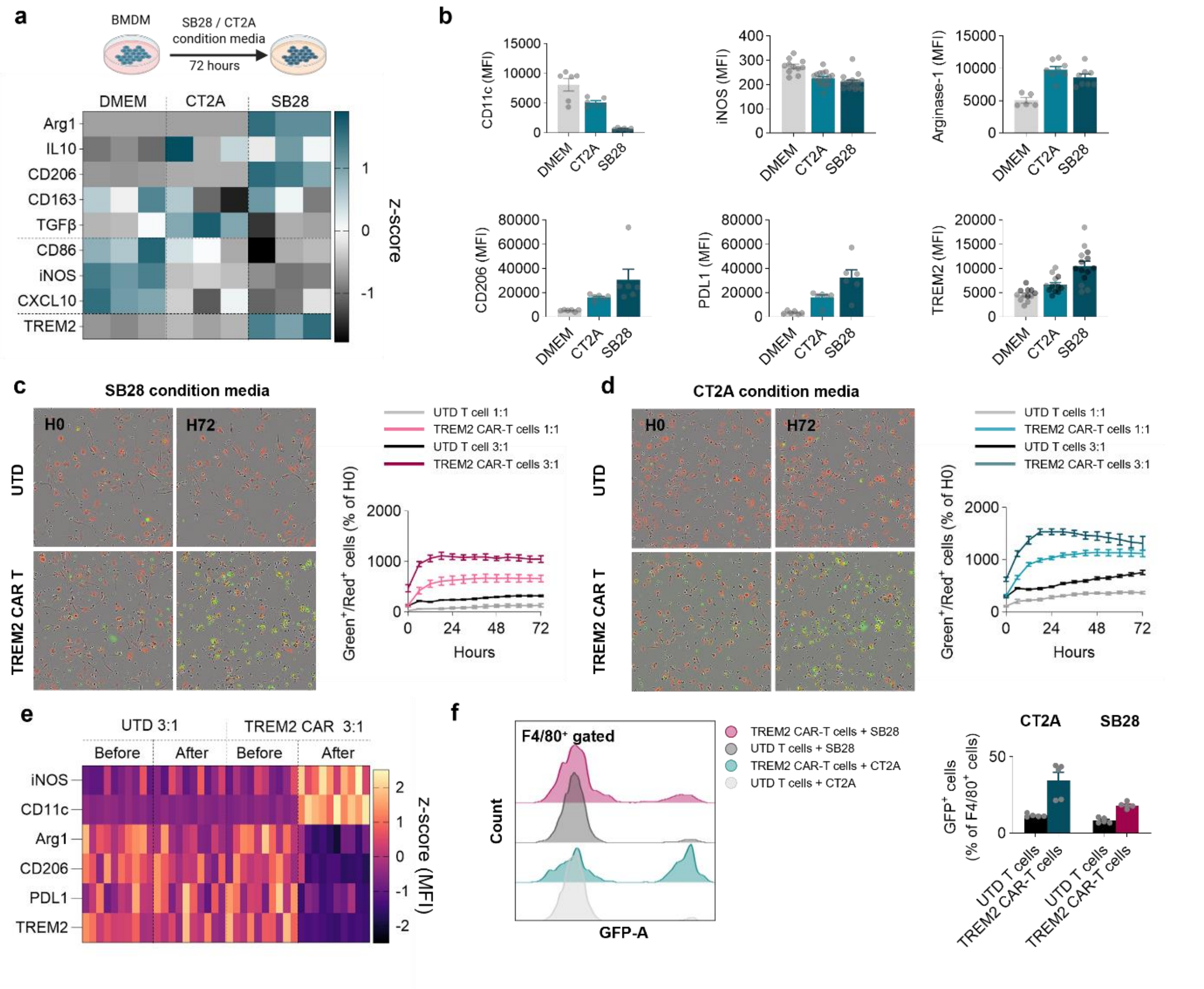

Supplementary Figure 10. TREM2 CAR-T cells eliminate and reprogram GBM-conditioned macrophages *in vitro*. (**a**) Heatmap of RT-qPCR gene expression (z-score) for Arg1, IL10, CD206, CD163, TGFβ, CD86, iNOS, CXCL10, and TREM2 in bone marrow–derived macrophages (BMDM) cultured in control medium (DMEM), CT2A-conditioned medium, or SB28-conditioned medium for 72 h (n=3 technical replicates per group). Created in BioRender. Nicola, C. (2026) https://BioRender.com/1lylymz. (**b**) Flow cytometry quantification of surface protein levels (MFI) for CD11c, iNOS, Arginase-1, CD206, PD-L1, and TREM2 in BMDM cultured in DMEM, CT2A-conditioned medium, or SB28-conditioned medium for 72 h (n=5-16 technical replicates per group). (**c-d**) Representative IncuCyte images of BMDM pre-exposed to SB28- (**c**) or CT2A- (**d**) conditioned media (CM) and co-cultured with untransduced (UTD) or TREM2 CAR-T cells at an effector-to-target (E:T) ratio of 1:1 or 3:1. Images are shown at the start of co-culture (H0) and after 72 h (H72). BMDM are labeled with CellTrace Yellow, and dead cells are detected using Cytotox Green. Quantification of macrophage killing over time, expressed as the normalized area of double-positive (CellTrace Yellow^{+}/Cytotox Green^{+}) signal relative to H0, for SB28-CM (**c**) and CT2A-CM (**d**)-conditioned BMDM co-cultured with UTD or TREM2 CAR-T cells (n=3-6 technical replicates per condition). (**e**) Heatmap showing z-scored mean fluorescence intensity (MFI) of indicated markers in F4/80^{+} BMDM before and after co-culture with SB28-CM and UTD or TREM2 CAR-T cells (E:T 3:1) (n=10 technical replicates/group). (**f**) Flow cytometry analysis of F4/80^{+} BMDM showing GFP^{+} target uptake following 24 h co-culture with GFP^{+}GD2^{+} SB28 or CT2A tumor cells after prior exposure to UTD or TREM2 CAR-T cells (3:1 E:T ratio). Quantification

of the percentage of GFP^+ cells within the $F4/80^+$ population is shown on the right (n= 5 technical replicates/group). Data are shown as mean +/- SEM. Source data and exact p values are provided as a Source Data file.

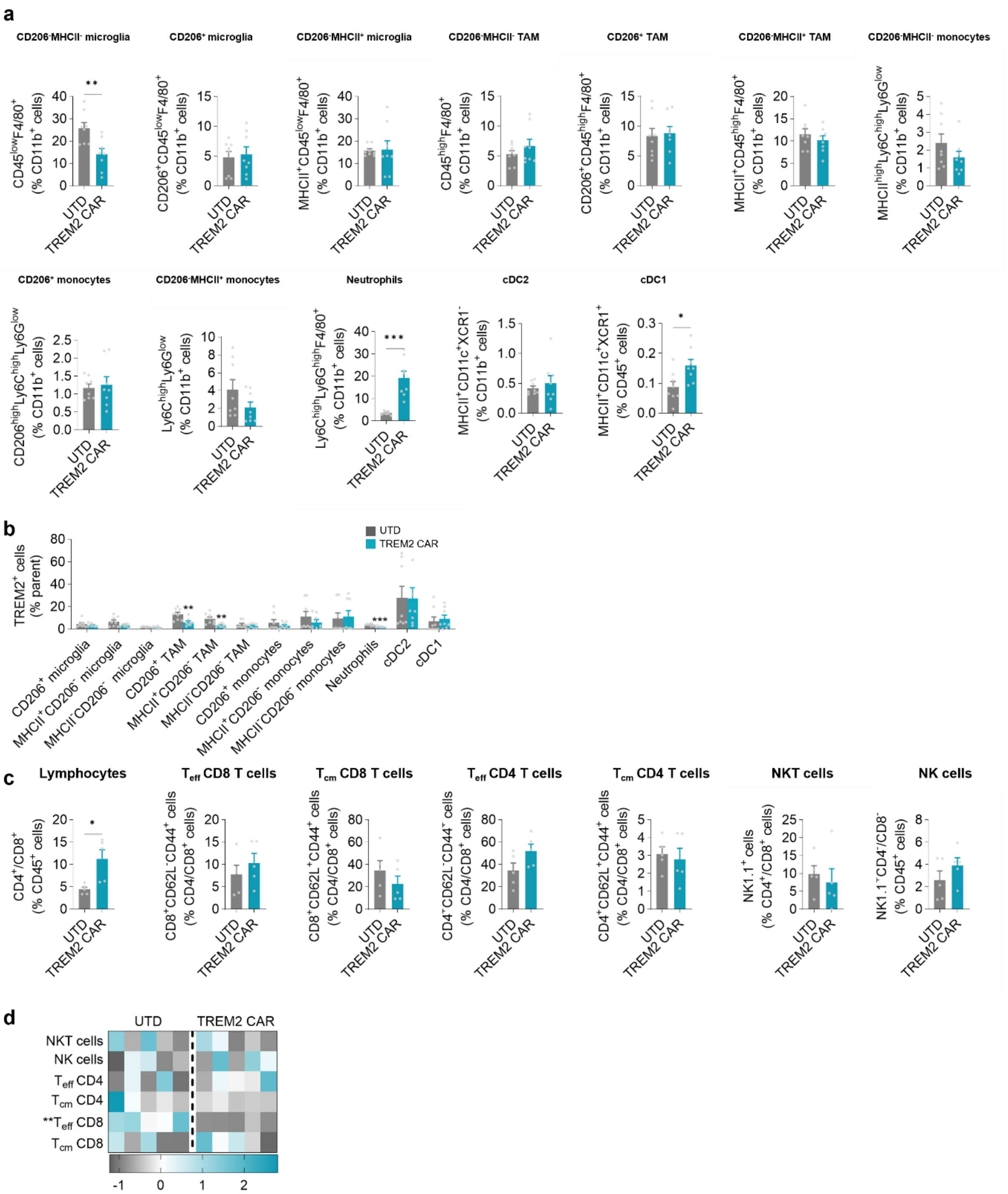

Supplementary Figure 11. TREM2 CAR-T cells deplete TREM2⁺ myeloid cells, recruit pro-inflammatory neutrophils, and reduce T cell exhaustion-like phenotypes *in vivo*. (**a**) Frequency of microglia, tumor-associated macrophage (TAM), monocyte, neutrophil and dendritic cell (cDC2 and cDC1) subsets in resected SB28 tumors treated with UTD or TREM2 CAR-T cells shown as percentage of $CD45^{+}CD11b^{+}$ cells (n=8 mice per group; data pooled from two independent experiments, unpaired two-tailed t-test). (**b**) Distribution of TREM2 expression across individual myeloid cell subsets in resected SB28 tumors treated with UTD or TREM2 CAR-T cells (n=8 mice per group; two independent experiments, unpaired two-tailed t-test). (**c**) Frequency of $CD3^{+}$ T cells (as % of $CD45^{+}$ cells) in resected SB28 tumors treated with UTD or TREM2 CAR-T cells three days post-resection from one experiment (n=5 mice

per group, unpaired two-tailed t-test). (**d**) Exhaustion-like scores of T_{eff} $CD8^+$ T cells in resected SB28 tumors treated with UTD or TREM2 CAR-T cells three days post-resection from one experiment (n=5 mice per group, unpaired two-tailed t-test). Data are shown as mean ± SEM. *p<0.05, **p<0.01, ***p<0.001. Source data and exact p values are provided as a Source Data file.

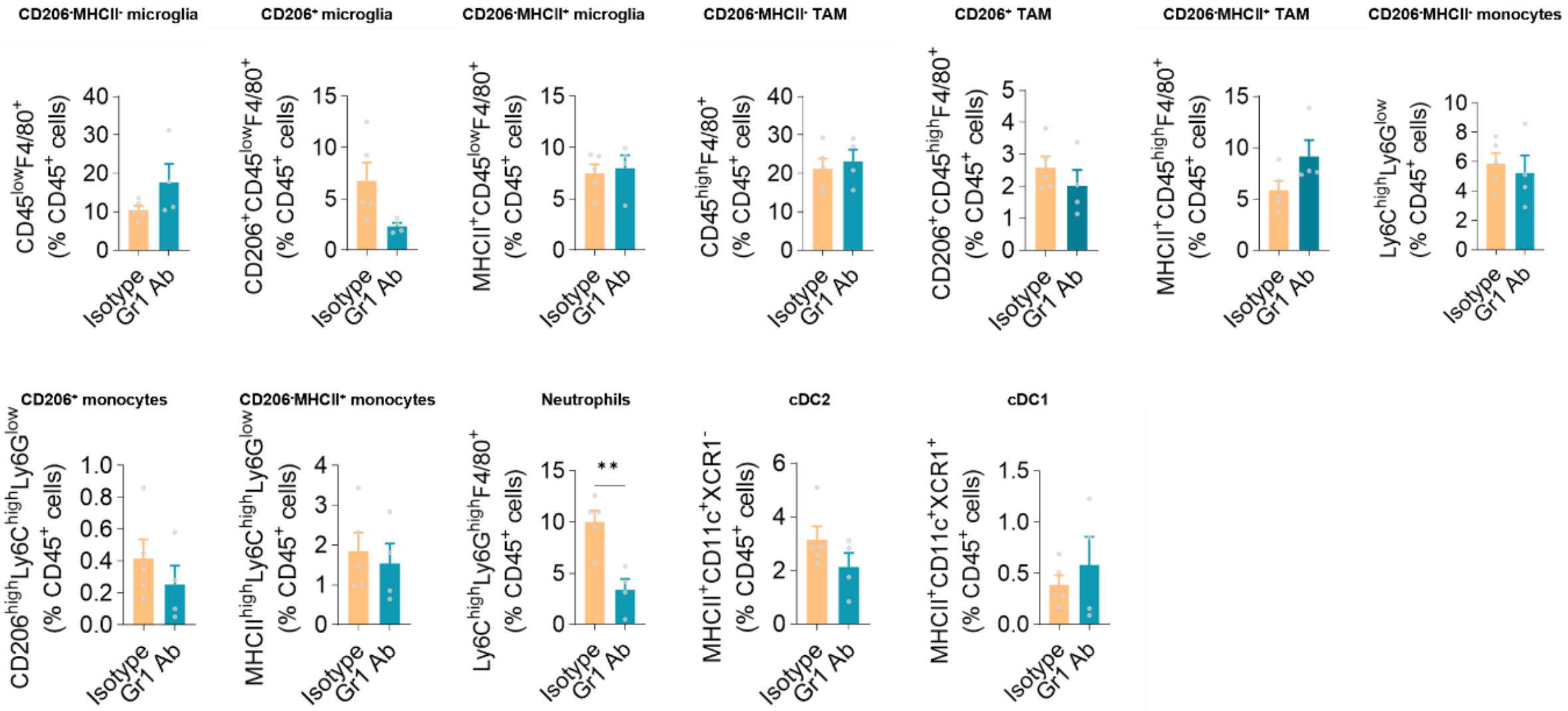

Supplementary figure 12. Impact of neutrophil depletion on myeloid cell compartment composition following TREM2 CAR-T cell therapy. Frequency of microglia, tumor-associated macrophage (TAM), monocyte, neutrophil and dendritic cell (cDC2 and cDC1) subsets in isotype *versus* anti-Gr1 antibody (Ab)-treated SB28-bearing mice treated with TREM2 CAR-T cells shown as percentage of $CD45^+$ cells from one experiment (n=5 mice for isotype and n=4 for Gr1 Ab group). Data are shown as mean ± SEM. Unpaired two-tailed t-tests were used. *$p < 0.05$, **$p < 0.01$. Source data and exact p values are provided as a Source Data file.

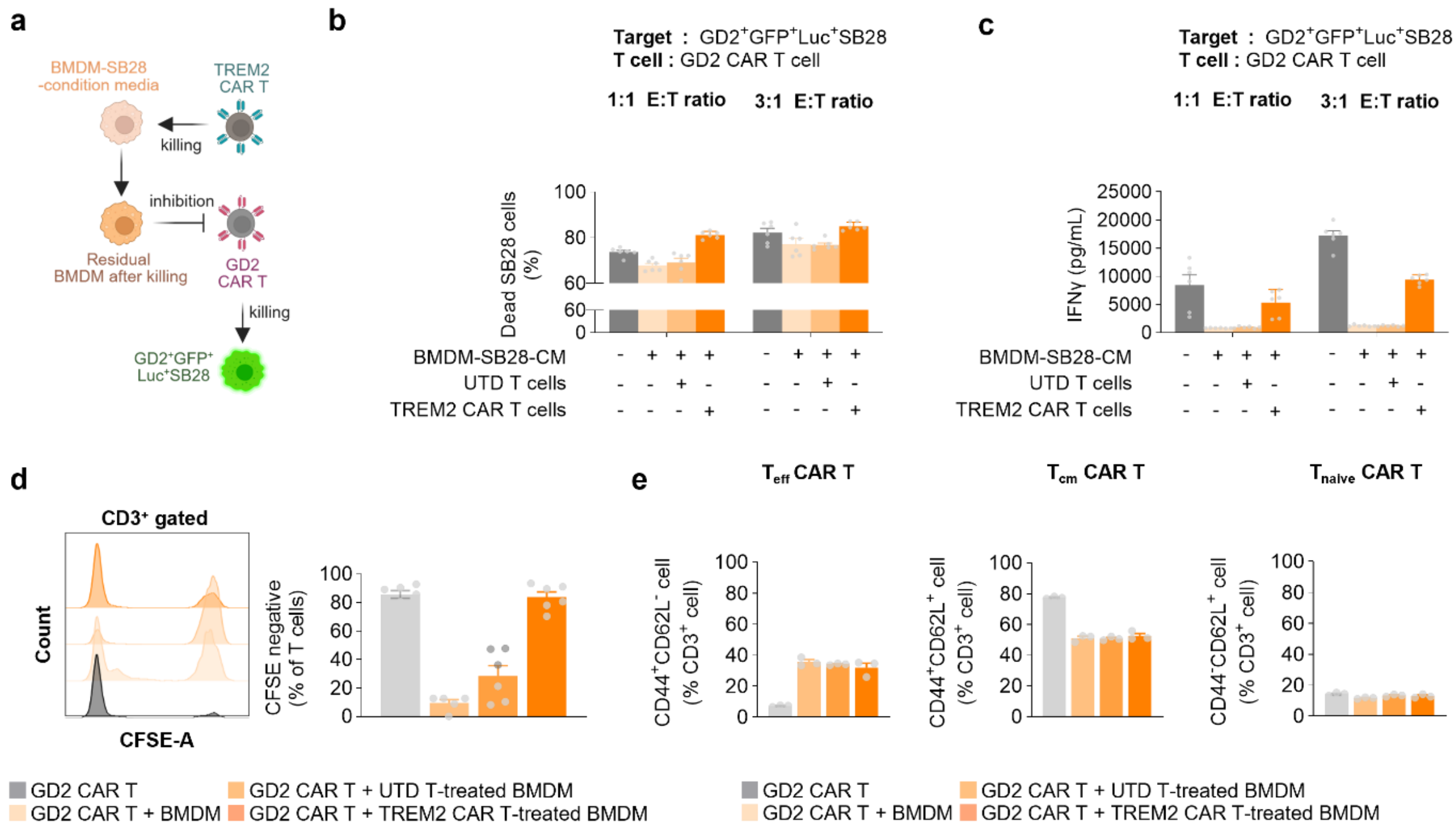

Supplementary figure 13. Impact of TREM2 CAR-T cell-conditioned macrophages on GD2 CAR-T cell activity. (**a**) Experimental scheme: bone marrow-derived macrophages (BMDM) were pretreated with conditioned medium from SB28 cells (SB28-CM), then exposed to either untransduced (UTD) or TREM2 CAR-T cells before co-culture with GD2 CAR-T cells and SB28 tumor cells. Created in BioRender. Nicola, C. (2026) https://BioRender.com/23710te. (**b**) Percentage of dead SB28 tumor cells after co-culture with GD2 CAR-T cells at effector:target (E:T) ratios of 1:1 and 3:1, in the presence of BMDM pretreated with UTD or TREM2 CAR-T cells (n=6 technical replicates from two independent experiments). (**b**) Quantification of IFNγ secretion (pg/mL) by GD2 CAR-T cells measured from the same co-culture experiments shown in (**a**) (n=6 technical replicates from two independent experiments). (**c**) Left: representative histograms of CFSE dilution in GD2 CAR-T cells after co-culture. Right: Quantification of CFSE⁺ GD2 CAR-T cells expressed as percentage of $CD3^+$ T cells (n=6 technical replicates from two independent experiments). (**d**) Frequency of effector T cells (T_{eff}), central memory T cells (T_{cm}), and naïve T cells (T_{naive}) among GD2 CAR-T cells in co-culture with UTD or TREM2 CAR-T cell-pretreated BMDM (n=3 technical replicates). Data are shown as mean ± SEM. Source data and exact p values are provided as a Source Data file.

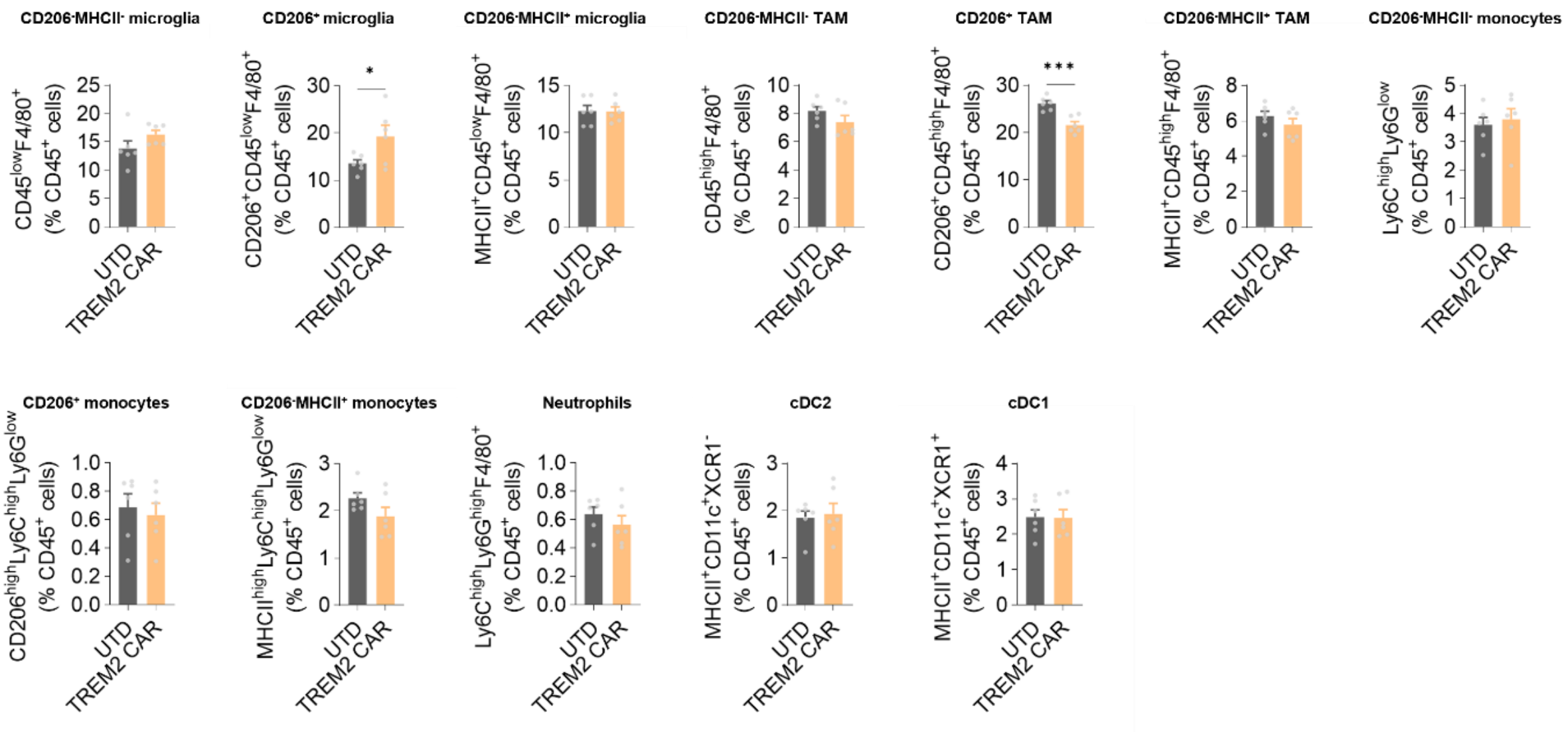

Supplementary figure 14. Myeloid cell remodeling following combined TREM2 and GD2 CAR-T cell therapy in SB28. Frequency of microglia, tumor-associated macrophage (TAM), monocyte, neutrophil and dendritic cell (cDC2 and cDC1) subsets in SB28 tumors treated with GD2 CAR-T cells combined with untransduced (UTD) or TREM2 CAR-T cells shown as percentage of $CD45^+$ cells from one experiment (n=6 mice per group). Data are shown as mean ± SEM. Unpaired two-tailed t-tests were used. *$p < 0.05$, ***$p < 0.001$. Source data and exact p values are provided as a Source Data file.

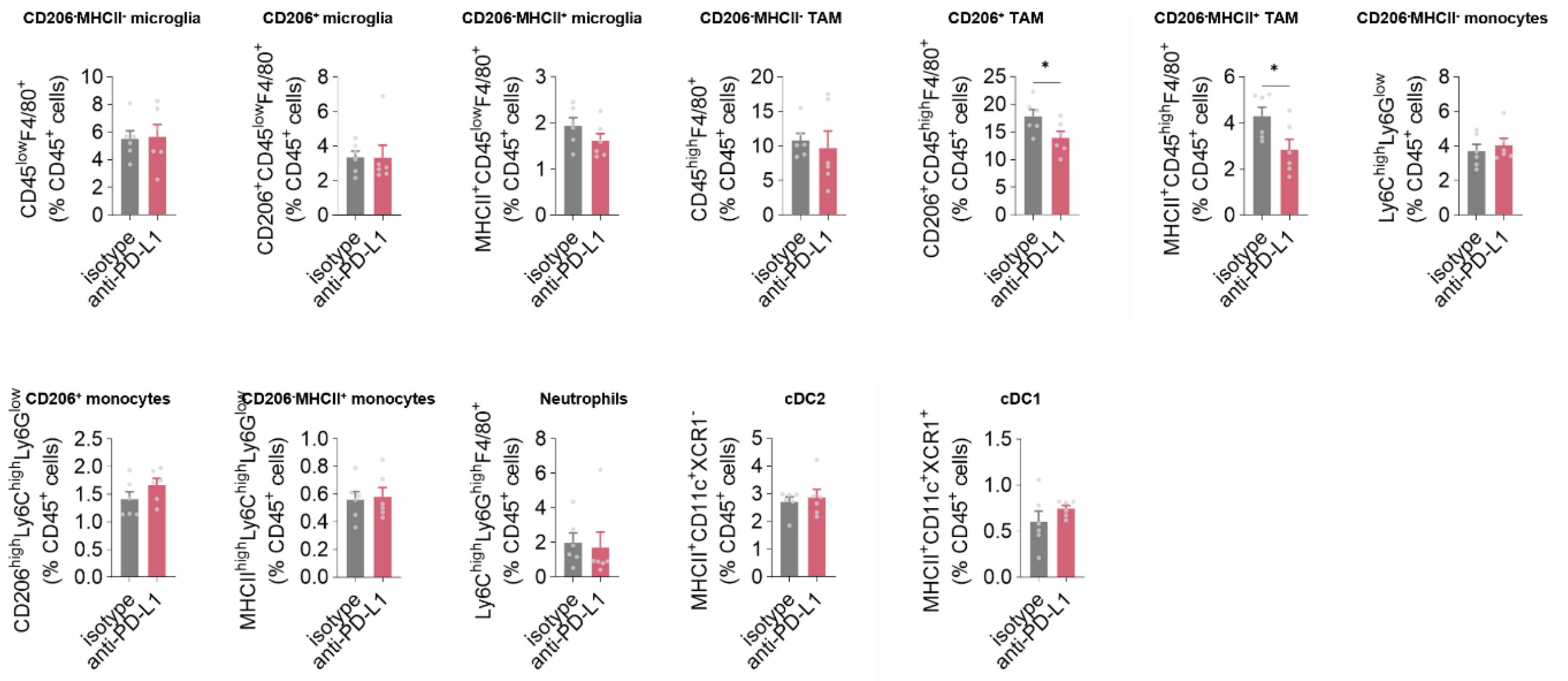

Supplementary figure 15. Effect of local injection of GD2 and TREM2 CAR-T cells combined with anti-PD-L1 in the SB28 model on the TME. Frequency of microglia, tumor-associated macrophage (TAM), monocyte, neutrophil and dendritic cell (cDC2 and cDC1) subsets in SB28 tumors treated with TREM2/GD2 CAR-T cell combined with anti-PD-L1 antibody or isotype control shown as percentage of $CD45^+$ cells from one experiment (n=6 mice per group). Data are shown as mean ± SEM. Unpaired two-tailed t-tests were used. *$p < 0.05$. Source data and exact p values are provided as a Source Data file.

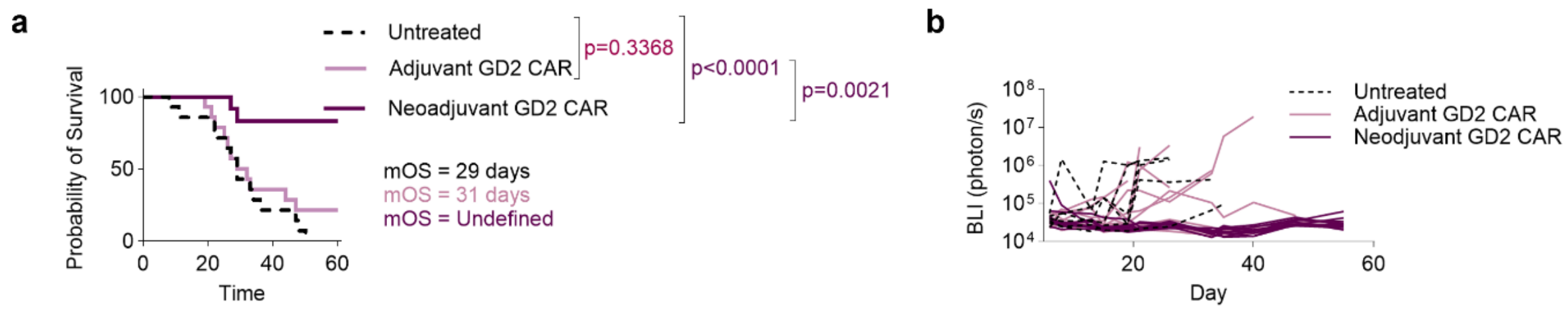

Supplementary figure 16. Neoadjuvant GD2 CAR-T cell therapy enhances tumor control and prolongs survival in the CT2A model. (**a**) Kaplan-Meier survival analysis of resected CT2A-bearing mice untreated or treated with adjuvant or neoadjuvant GD2 CAR-T cells pooled from two independent experiments (n=14 mice for untreated and adjuvant groups and n=12 mice for neoadjuvant group; log-rank test). mOS=median overall survival. (**b**) Longitudinal bioluminescence imaging (BLI) quantification of tumor growth in the three treatment groups (n=16 mice for untreated group and n=10 mice for adjuvant and neoadjuvant groups). Source data and exact p values are provided as a Source Data file.

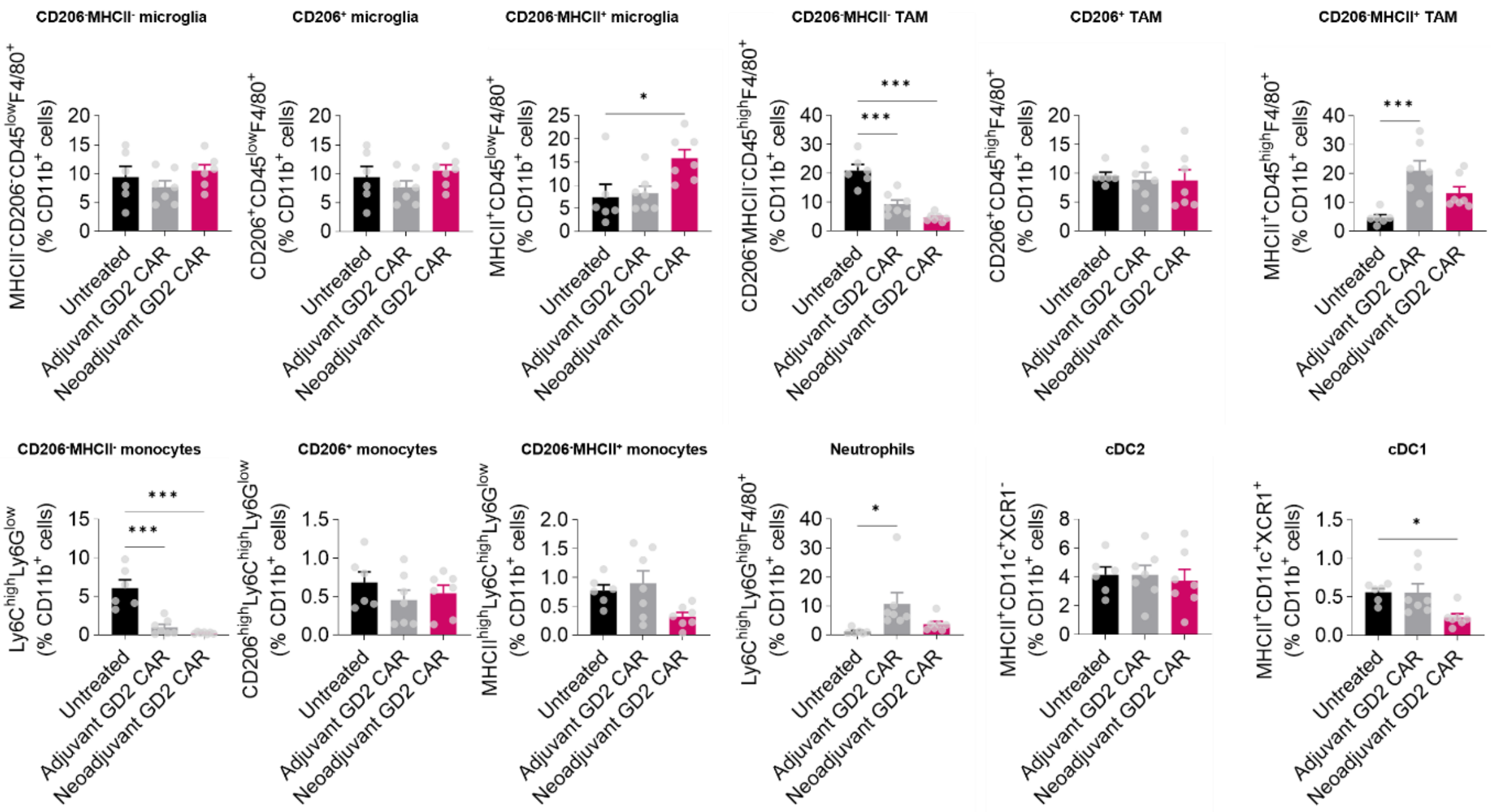

Supplementary figure 17. Myeloid cell compartment remodeling following neoadjuvant versus adjuvant GD2 CAR-T cell therapy in the SB28 model. Frequency of microglia, tumor-associated macrophage (TAM), monocyte, neutrophil and dendritic cell (cDC2 and cDC1) subsets in resected SB28-bearing mice untreated or treated with adjuvant or neoadjuvant GD2 CAR-T cells from one experiment. Data are shown as mean ± SEM (n=6 mice for untreated group and n=7 mice for adjuvant and neoadjuvant groups, unpaired t-test). *$p<0.05$, **$p<0.01$, ***$p<0.001$. Source data and exact p values are provided as a Source Data file.

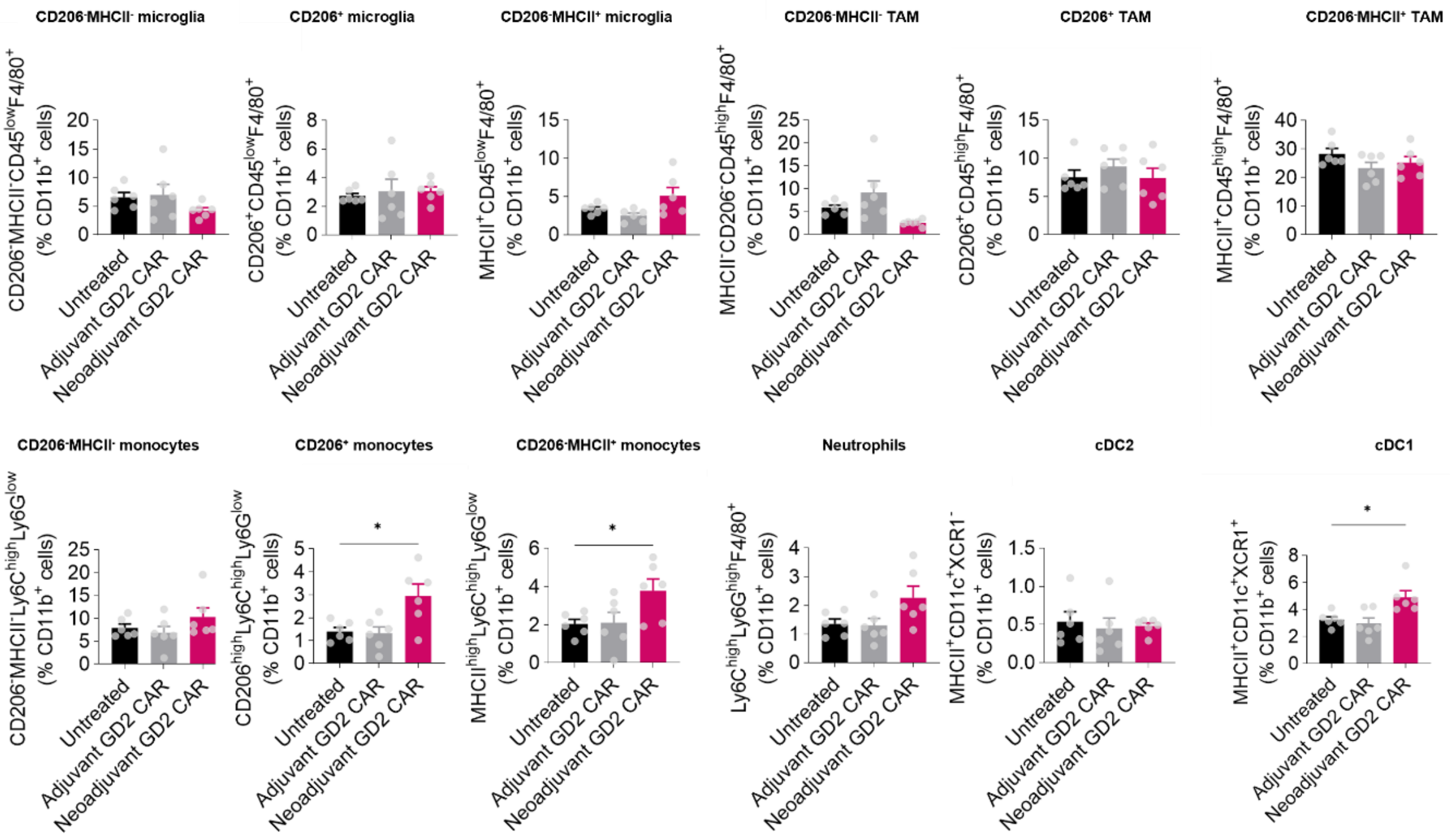

Supplementary figure 18. Myeloid cell compartment remodeling following neoadjuvant versus adjuvant GD2 CAR-T cell therapy in the CT2A model. Frequency of microglia, tumor-associated macrophage (TAM), monocyte, neutrophil and dendritic cell (cDC2 and cDC1) subsets in resected CT2A-bearing mice untreated or treated with adjuvant or neoadjuvant GD2 CAR-T cells from one experiment. Data are shown as mean ± SEM (n=6 per condition, unpaired t-test). *p<0.05, **p<0.01, ***p<0.001. Source data and exact p values are provided as a Source Data file.

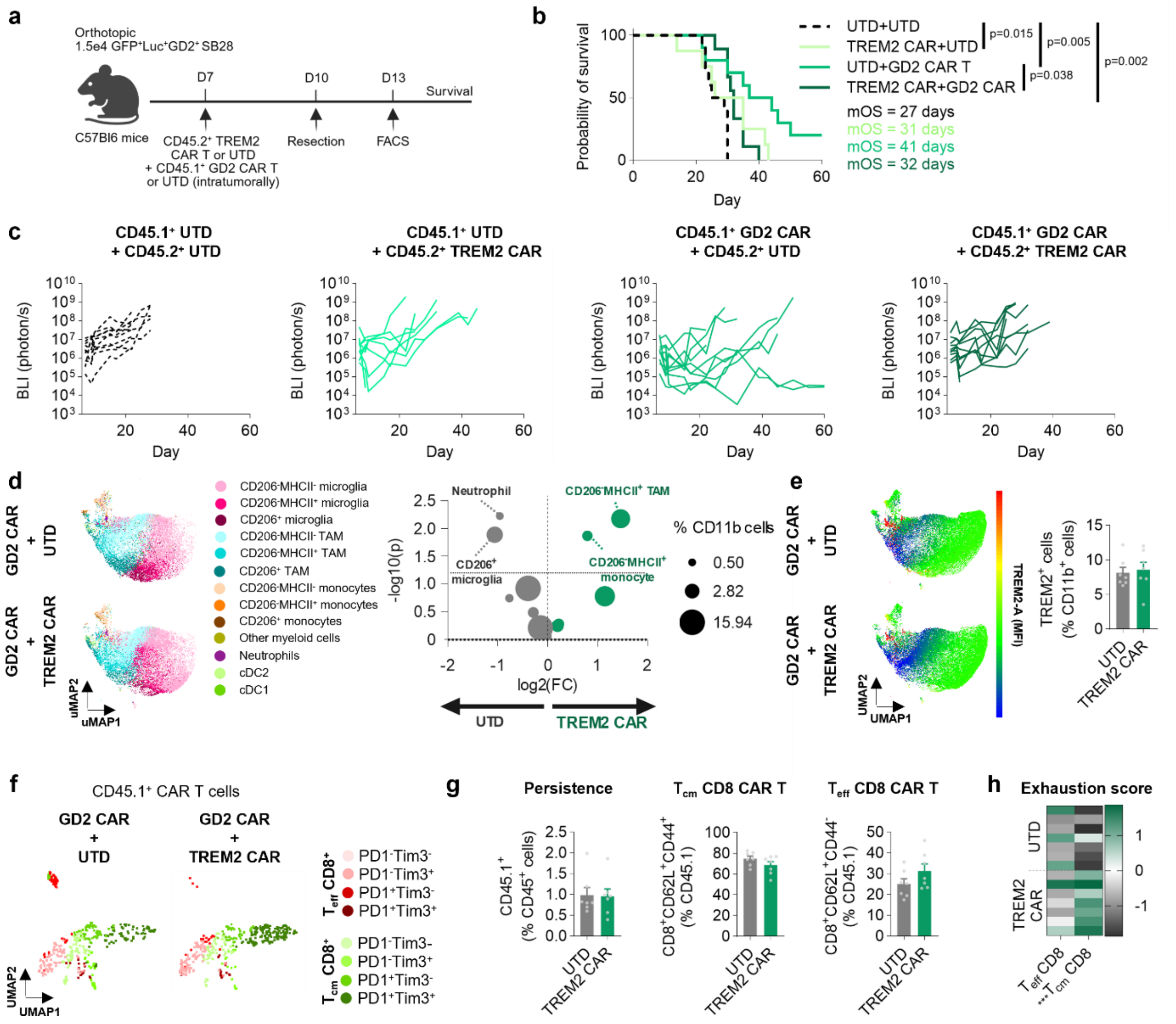

Supplementary figure 19. Effect of combining TREM2 and GD2 CAR-T cells as neoadjuvant therapy in SB28 GBM. (**a**) Experimental scheme of SB28-bearing mice treated with two T cell populations (5×10^5 each): untransduced (UTD)+UTD, TREM2 CAR+UTD, UTD+GD2 CAR, or TREM2 CAR+GD2 CAR delivered intratumorally as neoadjuvant on day 7 and resected on day 10. Created in BioRender. Nicola, C. (2026) https://BioRender.com/6o79xto. (**b**) Kaplan-Meier survival analysis of mice receiving the indicated neoadjuvant regimens from one experiment (n=10 for UTD+UTD and UTD+GD2 CAR groups, n=8 for TREM2 CAR+UTD group and n=9 for TREM2 CAR+GD2 CAR group; log-rank test). mOS=median overall survival. (**c**) Longitudinal bioluminescence imaging (BLI) quantification of tumor growth in individual mice from each treatment group. (**d**) Uniform manifold approximation and projection (UMAP) visualization of tumor infiltrating CD11b$^+$CD45$^+$ myeloid cells three days after resection in the UTD+GD2 CAR and TREM2 CAR+GD2 CAR neoadjuvant treatments (left). Volcano plot (right) showing log2 fold-changes (log2(FC)) and mean frequencies of major myeloid cell subsets in TREM2 CAR or UTD T cell-treated mice receiving GD2 CAR-T cells from one experiment (n=7, unpaired two-tailed t-test). (**e**) Left: UMAP visualization and quantification of TREM2 expression among CD11b$^+$CD45$^+$ myeloid cells three days post-resection in TREM2 CAR or UTD T cell-treated mice receiving GD2 CAR-T cells. Right: flow cytometry quantification of TREM2$^+$ cells among CD11b$^+$ cells in TREM2 CAR or UTD T cell-treated mice receiving GD2 CAR-T cells from one experiment

(n=7, unpaired two-tailed t-test). (**f**) UMAP of tumor-infiltrating $CD8^+CD45.1^+$ GD2 CAR-T cells colored by manually annotated clusters following TREM2 CAR or UTD T cell treatment. (**g**) Flow cytometry quantification of $CD45.1^+$ GD2 CAR-T cells among $CD4^+/CD8^+CD45^+$ cells and quantification of T_{eff} CD8 and T_{cm} CD8 among $CD45.1^+$ GD2 CAR-T cells in tumors of GD2 CAR-T cells treated in combination with UTD or TREM2 CAR-T cells from one experiment (n=7, unpaired two-tailed t-test). (**h**) Heatmap of exhaustion-like score (z-score; composite of exhaustion-associated markers, see Methods) for T_{eff} and T_{cm} $CD8^+CD45.1^+$ GD2 CAR-T cells (n=7 mice/condition, unpaired t-test). Data are shown as mean ± SEM. ***$p<0.001$. Source data and exact p values are provided as a Source Data file.

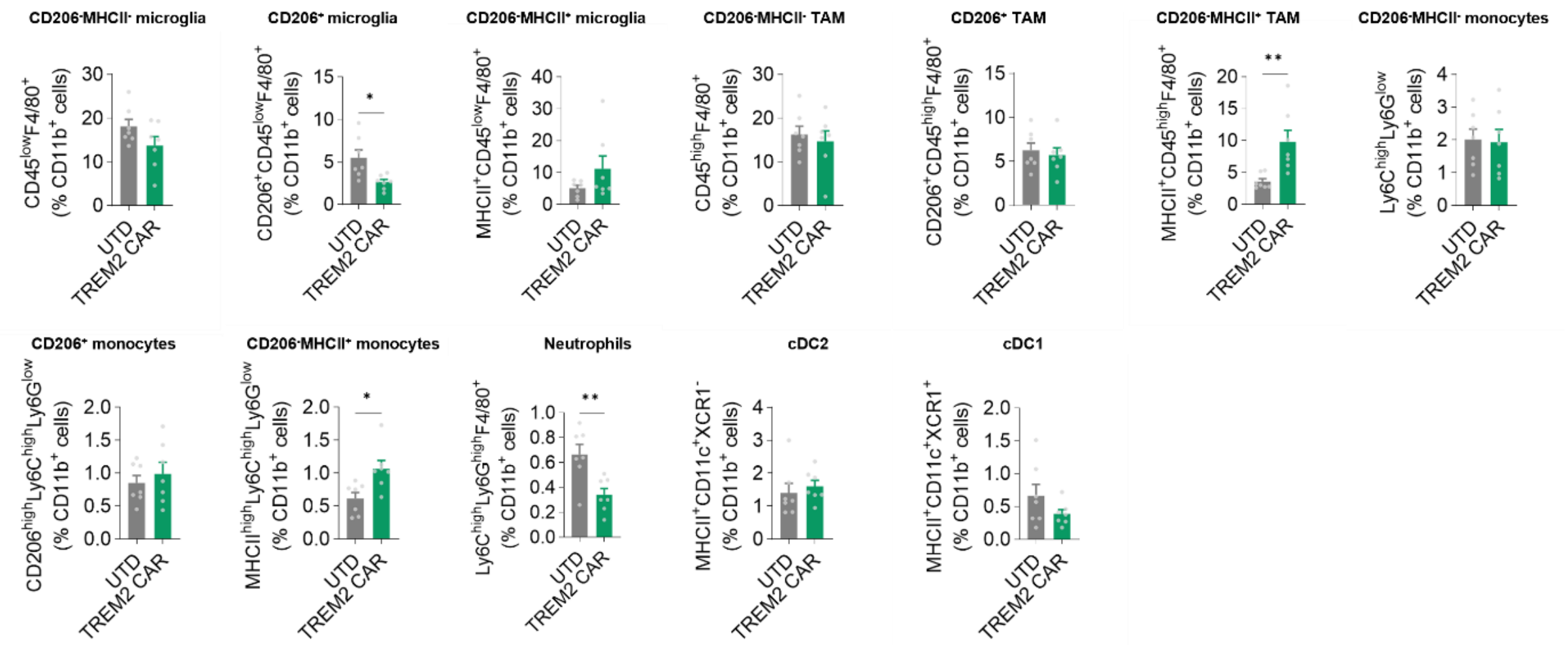

Supplementary figure 20. Effect of co-injection of TREM2 and GD2 CAR-T cells as neoadjuvant on the TME in the SB28 model. Frequency of microglia, tumor-associated macrophage (TAM), monocyte, neutrophil and dendritic cell (cDC2 and cDC1) subsets in GD2 CAR-T cell-treated mice bearing SB28 tumor receiving untransduced (UTD) or TREM2 CAR-T cells as neoadjuvant from one experiment (day 7, three days before resection). Data are shown as mean ± SEM (n=7/condition, unpaired t-test). *p<0.05, **p<0.01. Source data and exact p values are provided as a Source Data file.

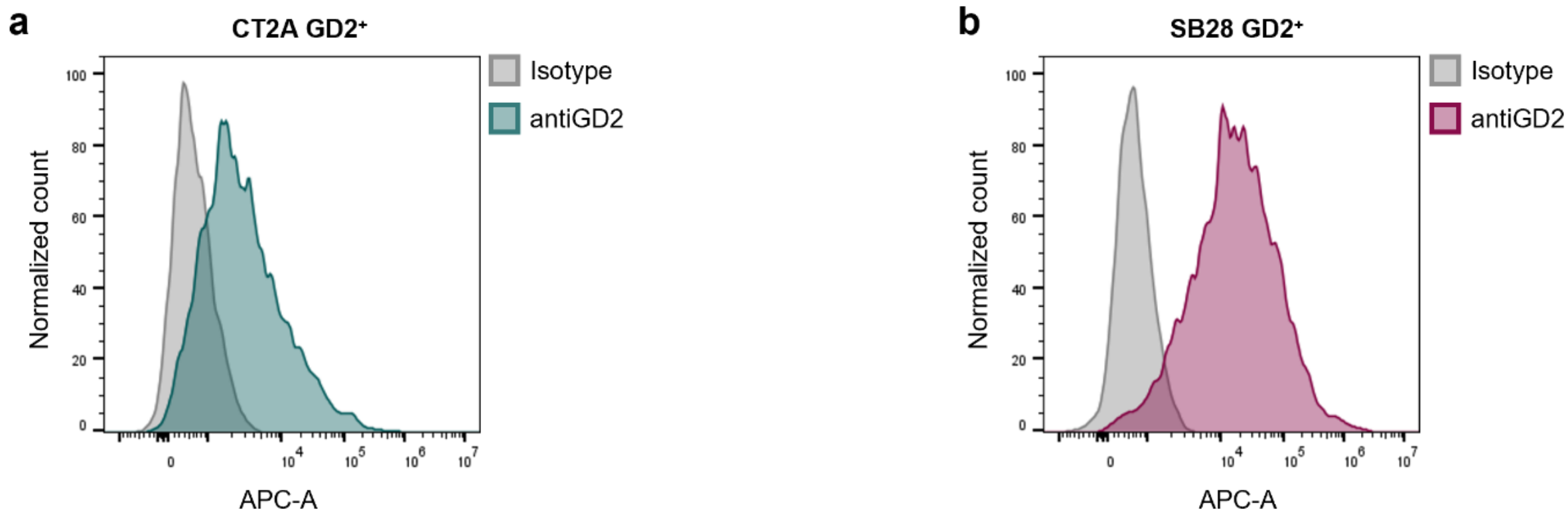

Supplementary figure 21. GD2 surface expression on murine GBM cell lines. Representative flow cytometry histograms showing GD2 surface expression on CT2A $GD2^+$ (**a**) and SB28 $GD2^+$ (**b**) cell lines cultured *in vitro*. Cells were stained with an anti-GD2 antibody (colored) or isotype control (grey).

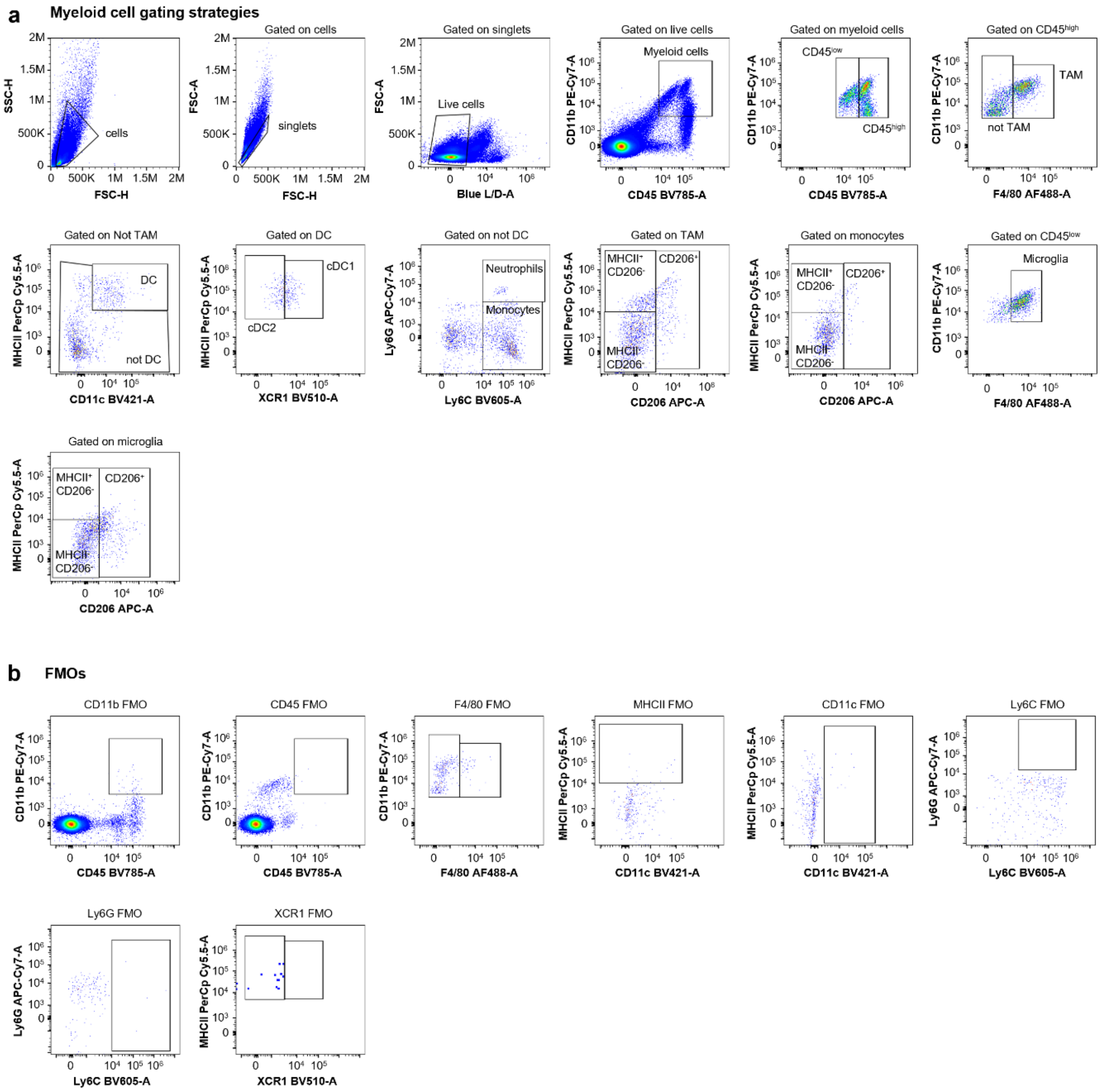

Supplementary Figure 22. Myeloid cell gating strategy and FMO controls. (**a**) Representative gating strategy for myeloid cell subsets. (**b**) Fluorescence Minus One (FMO) controls for each staining. This gating strategy was applied to all in vivo murine flow cytometry data of myeloid cell subsets, corresponding to Fig. 1c-e, Fig. 3c-e,i,j, Fig. 4c,d, Fig. 6a,b,f,g, Fig. 7h,i, and Supplementary Fig. 1b-e, 3c-f, 4, 5, 6, 7, 11a-b, 15, 17, 18, 19d-e and 20.

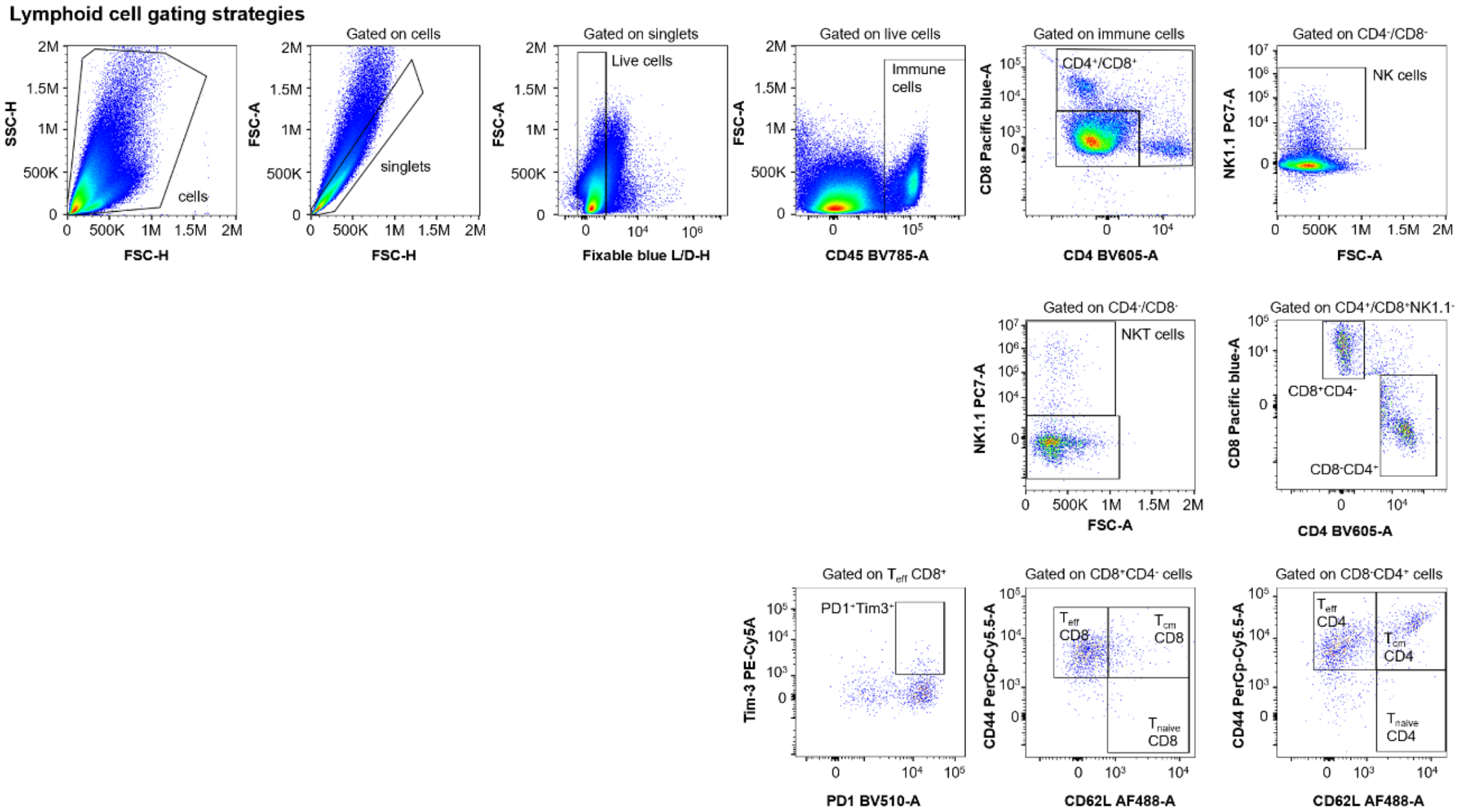

Supplementary Figure 23. Lymphoid cell gating strategy. Representative gating strategy for lymphoid cell subsets. This gating strategy was applied to all in vivo murine flow cytometry data of lymphoid cell subsets, corresponding to Fig. 2a-c, Fig. 4f-i, Fig. 5d,e, Fig. 6h-j, Fig. 7d-g,j-l, and Supplementary Fig. 1f-h, 3g-I, 8, 9, 11c-d and 19f-h.

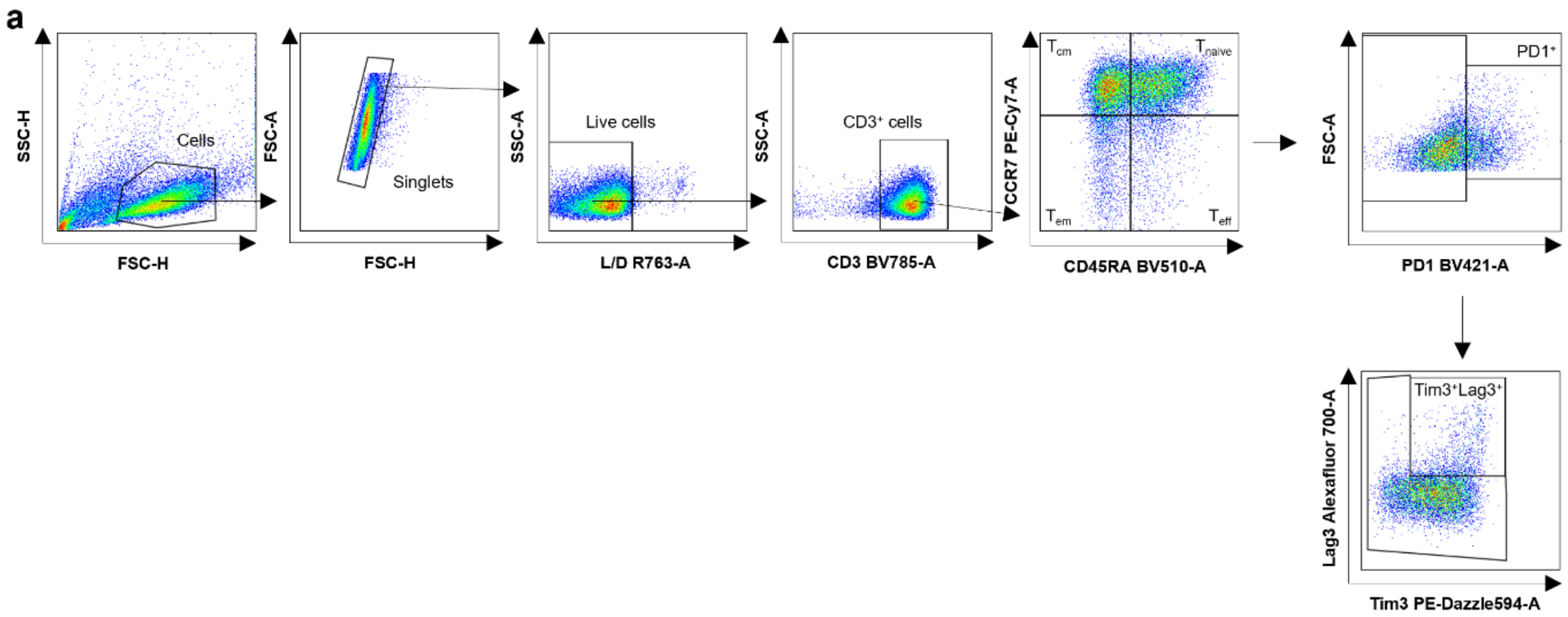

Supplementary figure 24. Gating strategy for flow analysis of human lymphoid cell subsets. Representative gating strategy for human lymphoid cell subsets. This gating strategy was applied to the ex vivo human GBM organotypic resection flow cytometry data of lymphoid cell subsets, corresponding to Fig. 2e-h.

Domain	TREM2 CAR	GD2 CAR
scFv	37012 (WO2020123664A1)	14g2a
Vector	pMSGV retroviral	pMSGV retroviral
Hinge/spacer	Murine CD28	Murine CD28
Transmembrane	Murine CD28	Murine CD28
Co-stimulatory	Murine CD28 intracellular	Murine CD28 intracellular
Signaling	Murine CD3ζ	Murine CD3ζ
Generation	2nd	2nd
Detection	Anti-$F(ab')_2$ staining	Anti-$F(ab')_2$ staining

Supplementary Table 1. Summary of CAR construct designs used in this study. Both TREM2 and GD2 CARs share an identical backbone architecture and differ only in their scFv domain.

Antigen	Fluorochrome	Reactivity	Clone	Provider	Reference	Dilution used	Validation
CD16/32	Unconjugated	Mouse	93	Biolegend	101301	1 :100	https://www.biolegend.com/en-us/products/purified-anti-mouse-cd16-32-antibody-190#productCertificate
CD45	BV785	Mouse	30-F11	Biolegend	103149	1 :100	https://www.biolegend.com/en-us/products/brilliant-violet-785-anti-mouse-cd45-antibody-10636
CD11b	PE-Cy7	Mouse/Human	M1/70	Biolegend	101216	1 :100	https://www.biolegend.com/en-us/products/pe-cyanine7-anti-mouse-human-cd11b-antibody-1921
F4/80	Alexafluor 488	Mouse	BM8	Biolegend	123120	1 :100	https://www.biolegend.com/en-us/products/alexa-fluor-488-anti-mouse-f4-80-antibody-4073
I-A/I-E	PerCp/Cy5.5	Mouse	M5/114.15.2	Biolegend	107626	1 :100	https://www.biolegend.com/en-us/products/percp-cyanine5-5-anti-mouse-i-a-i-e-antibody-4282
CD11c	BV421	Mouse	N418	Biolegend	117330	1 :100	https://www.biolegend.com/en-us/products/brilliant-violet-421-anti-mouse-cd11c-antibody-7149
CD206	Alexafluor 647	Mouse	C068C2	Biolegend	141712	1 :50	https://www.biolegend.com/en-us/products/alexa-fluor-647-anti-mouse-cd206-mmr-antibody-7427
Ly6C	BV605	Mouse	HK1.4	Biolegend	128036	1 :100	https://www.biolegend.com/en-us/products/brilliant-violet-605-anti-mouse-ly-6c-antibody-8727
Ly6G	APC-Cy7	Mouse	1A8	Biolegend	127624	1 :100	https://www.biolegend.com/en-us/products/apc-cyanine7-anti-mouse-ly-6g-antibody-6755
XCR1	BV510	Mouse	ZET	Biolegend	148218	1 :100	https://www.biolegend.com/en-us/products/brilliant-violet-510-anti-mouse-rat-xcr1-antibody-10751
TREM2	PE	Mouse	6E9	Biolegend	824805	1 :100	https://www.biolegend.com/en-us/products/pe-anti-trem-2-antibody-22902
CD3	APC-Cy7	Mouse	17A2	Biolegend	100222	1 :100	https://www.biolegend.com/en-us/products/apc-cyanine7-anti-mouse-cd3-antibody-6068
CD8	Pacific Blue	Mouse	53-6.7	Biolegend	100725	1 :100	https://www.biolegend.com/en-us/products/pacific-blue-anti-mouse-cd8a-antibody-2856
CD4	BV605	Mouse	GK1.5	Biolegend	100451	1 :100	https://www.biolegend.com/en-us/products/brilliant-violet-605-anti-mouse-cd4-antibody-10708
NK1.1	PC7	Mouse	PK136	Biolegend	108714	1 :100	https://www.biolegend.com/en-us/products/pe-cyanine7-anti-mouse-nk-1-1-antibody-2840
CD44	PerCp	Mouse/Human	IM7	Biolegend	103036	1 :100	https://www.biolegend.com/en-us/products/percp-anti-mouse-human-cd44-antibody-6895
CD62L	Alexafluor 488	Mouse	MEL-14	Biolegend	104420	1 :100	https://www.biolegend.com/en-us/products/alexa-fluor-488-anti-mouse-cd62l-antibody-3115
PD-1	BV510	Mouse	29F.1A12	Biolegend	135241	1 :100	https://www.biolegend.com/en-us/products/brilliant-violet-510-anti-mouse-cd279-pd-1-antibody-14923
Tim-3	PE	Mouse	B8.2C12	Biolegend	134004	1 :100	https://www.biolegend.com/en-us/products/pe-anti-mouse-cd366-tim-3-antibody-5908
CD45.1	PerCp-Cy5.5	Mouse	A20	BD Biosciences	560580	1 :100	https://www.bdbiosciences.com/en-eu/products/reagents/flow-cytometry-reagents/research-reagents/single-color-antibodies-ruo/percp-cy-5-5-mouse-anti-mouse-cd45-1.560580
CD45.2	BV785	Mouse	104	Biolegend	109839	1 :100	https://www.biolegend.com/en-us/products/brilliant-violet-785-anti-mouse-cd45-2-antibody-8924
PD-L1	BV785	Mouse	10F.9G2	Biolegend	124331	1 :100	https://www.biolegend.com/en-us/products/brilliant-violet-785-anti-mouse-cd274-b7-h1-pd-l1-antibody-13497
iNOS	PE	Mouse	CXNFT	Thermofisher	12-5920-82	1 :100	https://www.thermofisher.com/antibody/product/iNOS-Antibody-clone-CXNFT-Monoclonal/12-5920-82
Arginase-1	APC	Mouse	A1exF5	Thermofisher	17-3697-82	1 :100	https://www.thermofisher.com/antibody/product/Arginase-1-Antibody-clone-A1exF5-Monoclonal/17-3697-82
TREM2	Alexafluor 647	Mouse/Human	237920	R&D Systems	FAB17291R-025	1 :50	https://www.rndsystems.com/products/human-mouse-trem2-alexa-fluor-647-conjugated-antibody-237920_fab17291r
F4/80	BV421	Mouse	BM8	Biolegend	123137	1 :100	https://www.biolegend.com/en-us/products/brilliant-violet-421-anti-mouse-f4-80-antibody-7199
CD206	BV605	Mouse	C068C2	Biolegend	141721	1 :50	https://www.biolegend.com/en-us/products/brilliant-violet-605-anti-mouse-cd206-mmr-antibody-8729
CD11c	BV711	Mouse	N418	Biolegend	117349	1 :100	https://www.biolegend.com/en-us/products/brilliant-violet-711-anti-mouse-cd11c-antibody-10175
PD-L1	BV785	Mouse	10F.9G2	Biolegend	124331	1 :100	https://www.biolegend.com/en-us/products/brilliant-violet-785-anti-mouse-cd274-b7-h1-pd-l1-antibody-13497
CXCR2	APC	Mouse	M5/114.15.2	Biolegend	149312	1 :100	https://www.biolegend.com/en-us/products/apc-anti-mouse-cd182-antibody-15676
CD66a	APC-Cy7	Mouse	10F.9G2	Biolegend	134540	1 :100	https://www.biolegend.com/en-us/products/apccyanine7-anti-mouse-cd66a-ceacam1a-antibody-19636
CD44	BV510	Mouse	M1/70	Biolegend	103044	1 :100	https://www.biolegend.com/en-us/products/brilliant-violet-510-anti-mouse-human-cd44-antibody-7994
Ly6G	BV421	Mouse	1A8	Thermofisher	404-9668-82	1 :100	https://www.thermofisher.com/antibody/product/Ly-6G-Antibody-clone-1A8-Ly6g-Monoclonal/404-9668-82
Ly6C	PerCp/Cy5.5	Mouse	MAb-CC1	Biolegend	128012	1 :100	https://www.biolegend.com/en-us/products/percp-cyanine5-5-anti-mouse-ly-6c-antibody-5967
Arginase-1	FITC	Mouse	A1exF5	Thermofisher	53-3697-82	1 :100	https://www.thermofisher.com/antibody/product/Arginase-1-Antibody-clone-A1exF5-Monoclonal/53-3697-82
CD62L	PE-Cy7	Mouse	HK1.4	Biolegend	104418	1 :100	https://www.biolegend.com/en-us/products/pe-cyanine7-anti-mouse-cd62l-antibody-1922
I-A/I-E	BV785	Mouse	SA044G4	Biolegend	107645	1 :100	https://www.biolegend.com/en-us/products/brilliant-violet-785-anti-mouse-i-a-i-e-antibody-12087
PD-L1	PE	Mouse	10F.9G2	Biolegend	124308	1 :100	https://www.biolegend.com/en-us/products/pe-anti-mouse-cd274-b7-h1-pd-l1-antibody-4497
F4/80	PE	Mouse	W20065B	Biolegend	111604	1 :100	https://www.biolegend.com/en-us/products/pe-anti-mouse-f4-80-antibody-23902
PD-L1	BV510	Mouse	10F.9G2	BD Biosciences	571067	1 :100	https://www.bdbiosciences.com/en-de/products/reagents/flow-cytometry-reagents/research-reagents/single-color-antibodies-ruo/BV510-Rat-Anti-Mouse-CD274-(PD-L1).571067?tab=product_details
CD3	BV785	Human	SK7	Biolegend	344842	1 :100	https://www.biolegend.com/en-us/products/brilliant-violet-785-anti-human-cd3-antibody-13305
CD197 (CCR7)	PE-Cy7	Human	G043H7	Biolegend	353226	1 :100	https://www.biolegend.com/en-us/products/pecyanine7-anti-human-cd197-ccr7-antibody-7694
CD45RA	BV510	Human	Hl100	Biolegend	304142	1 :100	https://www.biolegend.com/en-us/products/brilliant-violet-510-anti-human-cd45ra-antibody-8007
PD-1	BV421	Human	EH12.2H7	Biolegend	329920	1 :100	https://www.biolegend.com/en-us/products/brilliant-violet-421-anti-human-cd279-pd-1-antibody-7191

Tim-3	PE-CF594	Human	7D3	BD Biosciences	2059595	1 :100	https://www.bdbiosciences.com/en-pl/products/reagents/flow-cytometry-reagents/research-reagents/single-color-antibodies-ruo/pe-cf594-mouse-anti-human-tim-3-cd366.565560?tab=product_details
Lag-3	Alexafluor 700	Human	11C3C65	Biolegend	369344	1 :100	https://www.biolegend.com/en-us/products/alexa-fluor-reg-700-anti-human-cd223-lag-3-21598
CD68	Alexafluor 555	Human	KP1	Thermofisher	754068882	1 :200	https://www.thermofisher.com/antibody/product/CD68-Antibody-clone-KP1-Monoclonal/754-0688-82
HLA-DR	Alexafluor 488	Human	LN3	Biolegend	327010	1 :200	https://www.biolegend.com/en-us/products/alexa-fluor-488-anti-human-hla-dr-antibody-4167

Supplementary Table 2. Antibodies used for flow cytometry and immunofluorescence staining.

Population	Defining markers	Parent gate
**Pre-gating sequence**		
Cells	FSC-H vs SSC-H	All events
Singlets	FSC-A vs FSC-H	Cells
Live cells	Fixable Blue L/D$^-$	Singlets
CD45$^+$ immune cells	CD45$^+$	Live cells
Myeloid cell gate	CD11b$^+$ F4/80$^+$	CD45$^+$
**Microglia (CD45^{low})**		
Microglia	CD45^{low} CD11b$^+$ F4/80$^+$	CD45$^+$
CD206$^–$ MHCII$^–$ microglia	CD206$^–$ MHCII$^–$	Microglia
CD206$^+$ MHCII$^{+/-}$ microglia	CD206$^+$ MHCII$^{+/-}$	Microglia
CD206$^-$ MHCII$^+$ microglia	CD206$^-$ MHCII$^+$	Microglia
**Macrophages (CD45^{high})**		
Macrophages	CD45^{high} CD11b$^+$ F4/80$^+$	CD45$^+$
CD206$^–$ MHCII$^–$ macrophages	CD206$^–$ MHCII$^–$	Macrophages
CD206$^+$ MHCII$^{+/-}$ macrophages	CD206$^+$ MHCII$^{+/-}$	Macrophages
CD206$^-$ MHCII$^+$ macrophages	CD206$^-$ MHCII$^+$	Macrophages
**Ly6C$^+$ monocytes**		
Ly6C$^+$ monocytes	CD45^{high} CD11b$^+$ F4/80$^–$ Ly6C$^+$ Ly6G$^–$	CD45$^+$
CD206$^–$ MHCII$^–$ monocytes	CD206$^–$ MHCII$^–$	Ly6C$^+$ monocytes
CD206$^+$ MHCII$^{+/-}$ monocytes	CD206$^+$ MHCII$^{+/-}$	Ly6C$^+$ monocytes
CD206$^-$ MHCII$^+$ monocytes	CD206$^-$ MHCII$^+$	Ly6C$^+$ monocytes
**Other myeloid cell populations**		
Neutrophils	CD45^{high} CD11b$^+$ Ly6G$^+$ Ly6C$^+$	CD45^{high} F4/80$^-$
cDC1	CD45^{high} CD11c$^+$ MHCII$^+$ XCR1$^+$	CD45^{high} F4/80$^-$
cDC2	CD45^{high} CD11c$^+$ MHCII$^+$ XCR1$^–$	CD45^{high} F4/80$^-$
Microglia is defined as CD45^{low}; macrophages and monocytes as CD45^{high}. All populations acquired on Cytoflex LX (Beckman Coulter). See Supplementary Fig. 22 for full gating strategy.		

Supplementary Table 3. Flow cytometry marker panel for myeloid cell populations

Population	Defining markers	Parent gate
**Pre-gating sequence**		
Cells	FSC-H vs SSC-H	All events
Singlets	FSC−A vs FSC-H	Cells
Live cells	Fixable Blue L/D$^-$	Singlets
CD45$^+$ immune cells	CD45$^+$	Live cells
**NKT cells**		
NKT cells	CD4$^+$/CD8$^+$ NK1.1$^+$	CD45$^+$ CD4/CD8$^+$
**CD4+ T cells**		
CD4$^+$ T cells	CD8$^-$ CD4$^+$ NK1.1$^-$	CD45$^+$ CD4/CD8$^+$
Tnaive CD4	CD44$^-$ CD62L$^+$	CD4$^+$ T cells
Tcm CD4	CD44$^+$ CD62L$^+$	CD4$^+$ T cells
Teff CD4	CD44$^+$ CD62L$^-$	CD4$^+$ T cells
**CD8+ T cells**		
CD8$^+$ T cells	CD4$^-$ CD8$^+$ NK1.1$^-$	CD45$^+$ CD4/CD8$^+$
T_{naive} CD8	CD44$^-$ CD62L$^+$	CD8$^+$ T cells
T_{cm} CD8	CD44$^+$ CD62L$^+$	CD8$^+$ T cells
T_{eff} CD8	CD44$^+$ CD62L$^-$	CD8$^+$ T cells
**NK cells**		
NK cells	CD45$^+$ CD4/CD8$^-$ NK1.1$^+$	Live cells
**Exhaustion markers (T_{naive}, T_{eff} and T_{cm} subsets)**		
PD1$^+$ Tim3$^+$ (co-expression)	PD1+ Tim3$^+$	T_{naive} / T_{eff} / T_{cm}
Exhaustion score	0.5 × (%PD1$^+$Tim3$^+$) + 0.3 × (MFI Tim3) + 0.2 × (MFI PD1)	T_{naive} / T_{eff} / T_{cm}
Exhaustion score is a composite weighted metric integrating co-inhibitory receptor co-expression frequency and mean fluorescence intensity. See Supplementary Fig. 23 for full gating strategy.		

Supplementary Table 4. Flow cytometry marker panel for lymphoid cell populations

Gene	Forward primer (5’→3’)	Reverse primer (5’→3’)
Eef1a1	ACGAGGCAATGTTGCTGGTGAC	GTGTGACAATCCAGAACAGGAGC
Arginase 1	TTTTAGGGTTACGGCCGGTG	CCTCGAGGCTGTCCTTTTGA
Il10	GGTTGCCAAGCCTTATCGGA	GGGGAGAAATCGATGACAGC
Cd206	GTTCACCTGGAGTGATGGTTCTC	AGGACATGCCAGGGTCACCTTT
Cd163	GGCTAGACGAAGTCATCTGCAC	CTTCGTTGGTCAGCCTCAGAGA
Tgfb	TGACGTCACTGGAGTTGTACGG	GGTTCATGTCATGGATGGTGC
Cd86	TGTTTCCGTGGAGACGCA	TTGAGCCTTTGTAAATGG
Inos	GCCACCAACAATGGCAACAT	TCGATGCACAACTGGGTGAA
Cxcl10	ATCATCCCTGCGAGCCTATCCT	GACCTTTTTTGGCTAAACGCTTTC
Trem2	CTACCAGTGTCAGAGTCTCCGA	CCTCGAAACTCGATGACTCCTC

Supplementary Table 5. Primer sequences used for quantitative RT-PCR.
