# Supplementary material for: Perioperative myeloid cell remodeling shapes CAR-T cell efficacy in glioblastoma
Source: Nat Commun. 2026 Jul 31;17:9727. doi: 10.1038/s41467-026-76206-y (PMC13562681; doi:10.1038/s41467-026-76206-y)
Supplement: Supplementary file 2 [file 41467_2026_76206_MOESM2_ESM.pdf]

nature portfolio

Corresponding author(s): Martin Pedard

Last updated by author(s): 2026/02/09

Reporting Summary

Nature Portfolio wishes to improve the reproducibility of the work that we publish. This form provides structure for consistency and transparency in reporting. For further information on Nature Portfolio policies, see our Editorial Policies and the Editorial Policy Checklist.

Statistics

For all statistical analyses, confirm that the following items are present in the figure legend, table legend, main text, or Methods section.

n/a	Confirmed	
☐	☒	The exact sample size (n) for each experimental group/condition, given as a discrete number and unit of measurement
☐	☒	A statement on whether measurements were taken from distinct samples or whether the same sample was measured repeatedly
☐	☒	The statistical test(s) used AND whether they are one- or two-sided<br>*Only common tests should be described solely by name; describe more complex techniques in the Methods section.*
☒	☐	A description of all covariates tested
☐	☒	A description of any assumptions or corrections, such as tests of normality and adjustment for multiple comparisons
☐	☒	A full description of the statistical parameters including central tendency (e.g. means) or other basic estimates (e.g. regression coefficient) AND variation (e.g. standard deviation) or associated estimates of uncertainty (e.g. confidence intervals)
☐	☒	For null hypothesis testing, the test statistic (e.g. F, t, r) with confidence intervals, effect sizes, degrees of freedom and P value noted<br>*Give P values as exact values whenever suitable.*
☒	☐	For Bayesian analysis, information on the choice of priors and Markov chain Monte Carlo settings
☒	☐	For hierarchical and complex designs, identification of the appropriate level for tests and full reporting of outcomes
☒	☐	Estimates of effect sizes (e.g. Cohen's d, Pearson's r), indicating how they were calculated

Our web collection on statistics for biologists contains articles on many of the points above.

Software and code

Policy information about availability of computer code

Data collection	Microscopy data were collected with a Ti2 Spinning Disk (Nikon). Flow cytometry data were collected using the Cytoflex. Luciferase radiance from brain tumors was acquired using an IVIS apparatus (PerkinElmer).
Data analysis	GraphPad Prism (version 10.6.1), Microsoft Excel (Version 2601), RStudio (R version 4.4.1), Seurat (version 5.4.0, R version 4.5.1) for single-cell RNA-sequencing analysis, FlowJo (Version v10.0.0), Fiji (Version 1.54f) and IVIS software (v4.7.3, PerkinElmer) were used for data analysis. The custom R script used for analysis of the immunohistofluorescence data is available from https://github.com/pedarmar-git/TREM2_GFP_analysis.

For manuscripts utilizing custom algorithms or software that are central to the research but not yet described in published literature, software must be made available to editors and reviewers. We strongly encourage code deposition in a community repository (e.g. GitHub). See the Nature Portfolio guidelines for submitting code & software for further information.

Data

Policy information about availability of data

All manuscripts must include a data availability statement. This statement should provide the following information, where applicable:

- Accession codes, unique identifiers, or web links for publicly available datasets
- A description of any restrictions on data availability
- For clinical datasets or third party data, please ensure that the statement adheres to our policy

The data supporting the results in this study are available within the paper, the supplementary information, the source data file or are available from the

corresponding author upon reasonable request.

Research involving human participants, their data, or biological material

Policy information about studies with human participants or human data. See also policy information about sex, gender (identity/presentation), and sexual orientation and race, ethnicity and racism.

Reporting on sex and gender	Information on sex and gender was not considered in the study design and analysis.
Reporting on race, ethnicity, or other socially relevant groupings	No race, ethnicity, or other social categorization variables were used in this study, as it involved ex vivo tissue from three individual patients without group-based comparisons.
Population characteristics	The study used ex vivo tissue obtained from three adult patients diagnosed with grade IV glioma.<br>No treatment history was available or considered relevant for the experimental objectives of this study.
Recruitment	Ex vivo tissue was obtained from three patients undergoing clinically indicated tumor resection, with informed consent for research use. Patients were not actively recruited for this study. Tissue availability was based on clinical scheduling and consent status.
Ethics oversight	This study was approved by the Human Research and Ethics Committee of the Geneva University in Switzerland (protocols 2022-02109 and 2023-01928).

Note that full information on the approval of the study protocol must also be provided in the manuscript.

Field-specific reporting

Please select the one below that is the best fit for your research. If you are not sure, read the appropriate sections before making your selection.

[x] Life sciences [ ] Behavioural & social sciences [ ] Ecological, evolutionary & environmental sciences

For a reference copy of the document with all sections, see nature.com/documents/nr-reporting-summary-flat.pdf

Life sciences study design

All studies must disclose on these points even when the disclosure is negative.

Sample size	Sample sizes were chosen to allow statistical comparison between treatment groups. No formal statistical methods were used to predetermine sample size. Instead, sizes were based on prior experience with similar experimental models, expected effect sizes, and feasibility constraints (e.g., animal avaibility ethical considerations, imaging resources).For in vitro experiments, if not stated otherwise, a sample size of 3 replicates was used. For in vivo experiments,on the therapeutic effects, the details regarding sample sizes are provided in the figure legends.
Data exclusions	No data were excluded from the experimental results or analyses.
Replication	The numbers of experimental replicates conducted are reported within the text, accompanying figures or under material methods.
Randomization	Mice were randomized into different groups before treatment. In the in vitro experiments, cultured cells or ther samples were randomly assigned to different experimental groups.
Blinding	Blinding was not performed as the work does not involve groups of participants.

Reporting for specific materials, systems and methods

We require information from authors about some types of materials, experimental systems and methods used in many studies. Here, indicate whether each material, system or method listed is relevant to your study. If you are not sure if a list item applies to your research, read the appropriate section before selecting a response.

Materials & experimental systems

n/a	Involved in the study	
[ ]	[x]	Antibodies
[ ]	[x]	Eukaryotic cell lines
[x]	[ ]	Palaeontology and archaeology
[ ]	[x]	Animals and other organisms
[x]	[ ]	Clinical data
[x]	[ ]	Dual use research of concern
[x]	[ ]	Plants

Methods

n/a	Involved in the study	
[x]	[ ]	ChIP-seq
[ ]	[x]	Flow cytometry
[x]	[ ]	MRI-based neuroimaging

Antibodies

Antibodies used	Mouse :<br>CD16/32 – Unconjugated – Mouse – Clone 93 – BioLegend – Ref 101301 – Dilution 1:100<br>CD45 – BV785 – Mouse – Clone 30-F11 – BioLegend – Ref 103149 – Dilution 1:100<br>CD11b – PE-Cy7 – Mouse/Human – Clone M1/70 – BioLegend – Ref 101216 – Dilution 1:100F<br>4/80 – Alexa Fluor 488 – Mouse – Clone BM8 – BioLegend – Ref 123120 – Dilution 1:100<br>I-A/I-E – PerCP-Cy5.5 – Mouse – Clone M5/114.15.2 – BioLegend – Ref 107626 – Dilution 1:100<br>CD11c – BV421 – Mouse – Clone N418 – BioLegend – Ref 117330 – Dilution 1:100<br>CD206 – Alexa Fluor 647 – Mouse – Clone C068C2 – BioLegend – Ref 141712 – Dilution 1:50<br>Ly6C – BV605 – Mouse – Clone HK1.4 – BioLegend – Ref 128036 – Dilution 1:100<br>Ly6G – APC-Cy7 – Mouse – Clone 1A8 – BioLegend – Ref 127624 – Dilution 1:100<br>XCR1 – BV510 – Mouse – Clone ZET – BioLegend – Ref 148218 – Dilution 1:100<br>TREM2 – PE – Mouse – Clone 6E9 – BioLegend – Ref 824805 – Dilution 1:100C<br>D3 – APC-Cy7 – Mouse – Clone 17A2 – BioLegend – Ref 100222 – Dilution 1:100<br>CD8 – Pacific Blue – Mouse – Clone 53-6.7 – BioLegend – Ref 100725 – Dilution 1:100<br>CD4 – BV605 – Mouse – Clone GK1.5 – BioLegend – Ref 100451 – Dilution 1:100<br>NK1.1 – PE-Cy7 – Mouse – Clone PK136 – BioLegend – Ref 108714 – Dilution 1:100<br>CD44 – PerCP – Mouse/Human – Clone IM7 – BioLegend – Ref 103036 – Dilution 1:100<br>CD62L – Alexa Fluor 488 – Mouse – Clone MEL-14 – BioLegend – Ref 104420 – Dilution 1:100<br>PD-1 – BV510 – Mouse – Clone 29F.1A12 – BioLegend – Ref 135241 – Dilution 1:100<br>Tim-3 – PE – Mouse – Clone B8.2C12 – BioLegend – Ref 134004 – Dilution 1:100<br>CD45.1 – PerCP-Cy5.5 – Mouse – Clone A20 – BD Biosciences – Ref 560580 – Dilution 1:100<br>CD45.2 – BV785 – Mouse – Clone 104 – BioLegend – Ref 109839 – Dilution 1:100<br>PD-L1 – BV785 – Mouse – Clone 10F.9G2 – BioLegend – Ref 124331 – Dilution 1:100<br>iNOS – PE – Mouse – Clone CXNFT – ThermoFisher – Ref 12-5920-82 – Dilution 1:100<br>Arginase-1 – APC – Mouse – Clone A1exF5 – ThermoFisher – Ref 17-3697-82 – Dilution 1:100<br>TREM2 – Alexa Fluor 647 – Mouse/Human – Clone 237920 – R&D Systems – Ref FAB17291R-025 – Dilution 1:50<br>F4/80 – BV421 – Mouse – Clone BM8 – BioLegend – Ref 123137 – Dilution 1:100<br>CD206 – BV605 – Mouse – Clone C068C2 – BioLegend – Ref 141721 – Dilution 1:50<br>CD11c – BV711 – Mouse – Clone N418 – BioLegend – Ref 117349 – Dilution 1:100<br>CXCR2 – APC – Mouse – Clone M5/114.15.2 – BioLegend – Ref 149312 – Dilution 1:100<br>CD66a (CEACAM1a) – APC-Cy7 – Mouse – Clone 10F.9G2 – BioLegend – Ref 134540 – Dilution 1:100<br>CD44 – BV510 – Mouse – Clone M1/70 – BioLegend – Ref 103044 – Dilution 1:100<br>Ly6G – BV421 – Mouse – Clone 1A8 – ThermoFisher – Ref 404-9668-82 – Dilution 1:100<br>Ly6C – PerCP-Cy5.5 – Mouse – Clone MAb-CC1 – BioLegend – Ref 128012 – Dilution 1:100<br>Arginase-1 – FITC – Mouse – Clone A1exF5 – ThermoFisher – Ref 53-3697-82 – Dilution 1:100<br>CD62L – PE-Cy7 – Mouse – Clone HK1.4 – BioLegend – Ref 104418 – Dilution 1:100<br>I-A/I-E – BV785 – Mouse – Clone SA044G4 – BioLegend – Ref 107645 – Dilution 1:100<br>PD-L1 – PE – Mouse – Clone 10F.9G2 – BioLegend – Ref 124308 – Dilution 1:100<br>F4/80 – PE – Mouse – Clone W20065B – BioLegend – Ref 111604 – Dilution 1:100<br><br>Human :<br>CD3 – BV785 – Human – Clone SK7 – BioLegend – Ref 344842 – Dilution 1:100<br>CCR7 (CD197) – PE-Cy7 – Human – Clone G043H7 – BioLegend – Ref 353226 – Dilution 1:100<br>CD45RA – BV510 – Human – Clone HI100 – BioLegend – Ref 304142 – Dilution 1:100<br>PD-1 – BV421 – Human – Clone EH12.2H7 – BioLegend – Ref 329920 – Dilution 1:100<br>Tim-3 – PE-CF594 – Human – Clone 7D3 – BD Biosciences – Ref 2059595 – Dilution 1:100<br>LAG-3 – Alexa Fluor 700 – Human – Clone 11C3C65 – BioLegend – Ref 369344 – Dilution 1:100<br>CD68 – Alexa Fluor 555 – Human – Clone KP1 – ThermoFisher – Ref 754-0688-82 – Dilution 1:200<br>HLA-DR – Alexa Fluor 488 – Human – Clone LN3 – BioLegend – Ref 327010 – Dilution 1:200
Validation	Only commercial available and routinely used antibodies have been used in this study. All antibodies were validated by the provider, as indicated in the references provided by the manufacturer (Provided in supplemental tables)

Eukaryotic cell lines

Policy information about cell lines and Sex and Gender in Research

Cell line source(s)	The murine GBM cell lines SB28 (kindly provided by H. Okada, UCSF) and CT2A (commercially available) were used in this study. They were stably engineered to express GFP and firefly luciferase to allow in vivo imaging. CT2A and SB28 cells were modified to express GD2 by transduction with lentiviruses encoding GD2S and GD3S synthases.<br>Phoenix-Eco cells were obtained from ATCC (CRL-3214)<br>T2 cells were obtained from ATCC (CRL-1992)
Authentication	Authentication of parental stocks was performed at the time of acquisition, and morphology, growth kinetics, and tumorigenicity remained consistent with original reports throughout the study.

Mycoplasma contamination	All cell lines have been tested negative for Mycoplasma contamination in tests routinely performed in the laboratory.
Commonly misidentified lines (See ICLAC register)	None

Animals and other research organisms

Policy information about studies involving animals; ARRIVE guidelines recommended for reporting animal research, and Sex and Gender in Research

Laboratory animals	All animal experiments were performed using 7–8-week-old male C57BL/6 mice (CD45.2, Janvier Labs, France or Charles River, France). For congenic transfer experiments, CD45.1 C57BL/6 mice (Charles River, France) were also used. Mice were housed under specific pathogen–free conditions with ad libitum access to food and water.
Wild animals	No wild mice have been used in this study.
Reporting on sex	For this study, male mice were used.
Field-collected samples	None
Ethics oversight	All studies were conducted in accordance with the guidelines and regulations set by the Cantonal Veterinary Office of Vaud, Switzerland, following authorizations VD4017d.

Note that full information on the approval of the study protocol must also be provided in the manuscript.

Plants

Seed stocks	The study did not involve plants
Novel plant genotypes	*Describe the methods by which all novel plant genotypes were produced. This includes those generated by transgenic approaches, gene editing, chemical/radiation-based mutagenesis and hybridization. For transgenic lines, describe the transformation method, the number of independent lines analyzed and the generation upon which experiments were performed. For gene-edited lines, describe the editor used, the endogenous sequence targeted for editing, the targeting guide RNA sequence (if applicable) and how the editor was applied.*
Authentication	*Describe any authentication procedures for each seed stock used or novel genotype generated. Describe any experiments used to assess the effect of a mutation and, where applicable, how potential secondary effects (e.g. second site T-DNA insertions, mosiacism, off-target gene editing) were examined.*

Flow Cytometry

Plots

Confirm that:

- [x] The axis labels state the marker and fluorochrome used (e.g. CD4-FITC).
- [x] The axis scales are clearly visible. Include numbers along axes only for bottom left plot of group (a 'group' is an analysis of identical markers).
- [x] All plots are contour plots with outliers or pseudocolor plots.
- [x] A numerical value for number of cells or percentage (with statistics) is provided.

Methodology

Sample preparation	For both human and mice, samples were dissociated by using the brain tumor dissociation kit (Miltenyi, 130-095-942) in combination with the gentleMACS Octo Dissociator (Miltenyi, 130-134-029) after myelin removal with Percoll 30% gradient. For human T cells analysis, CD3 positive cells were isolated by using the easySep™ human T Cell isolation kit (Stemcell, # 17951). For mice brain tumor, single-cell suspensions were incubated in PBS with purified anti-mouse CD16/32 Fc block (1:100; clone 93, Rat IgG2a, Biolegend, 101301) and fixable live–dead colors and stained with antibodies (See Reporting Summary and Supplemental Table 1) before acquisition by flow cytometry.
Instrument	Flow cytometry data were acquired using CytoFLEX LX2 (Beckman Coulter).
Software	For flow cytomentry analysis, we employed FlowJo, LLC V10.1
Cell population abundance	For flow cytomentry analysis, we employed FlowJo, LLC V10.1
Gating strategy	Gating strategies are shown in Supplementary Figures 22-24<br><br>HUMAN CELLS:<br>Total T cells : CD45+ CD3+

Naive T cells : CD45+ CD3+ CD45RA+ CCR7+
Central memory T cells (Tcm) : CD45+ CD3+ CD45RA- CCR7+
Effector memory T cells (Tem) : CD45+ CD3+ CD45RA- CCR7-
Effector T cells (Teff) : CD45+ CD3+ CD45RA+ CCR7-
PD-1+ T cells : CD45+ CD3+ PD-1+
Tim-3+ Lag-3+ T cells : CD45+ CD3+ Tim-3+ Lag-3+

MOUSE CELLS (Myeloid panel):
CD206-MHCII- microglia : CD45low CD11b+ F4/80+ MHCII- CD206-
CD206-MHCII+ microglia : CD45low CD11b+ F4/80+ MHCII+ CD206-
CD206+ microglia : CD45low CD11b+ F4/80+ MHCII+/- CD206+
CD206-MHCII- TAM : CD45high CD11b+ F4/80+ MHCII- CD206-
CD206-MHCII+ TAM : CD45high CD11b+ F4/80+ MHCII+ CD206-
CD206+ TAM : CD45high CD11b+ F4/80+ MHCII+/- CD206+
CD206-MHCII- Monocytes : CD45high CD11b+ Ly6C+ Ly6G- MHCII- CD206-
CD206-MHCII+ Monocytes : CD45high CD11b+ Ly6C+ Ly6G- MHCII+ CD206-
CD206+ Monocytes : CD45high CD11b+ Ly6C+ Ly6G- MHCII+/- CD206+
Neutrophils : CD45high CD11b+ Ly6G+
Dendritic cells : CD45high CD11c+ MHCII+
cDC1 : CD45high CD11c+ MHCII+ XCR1+
cDC2 : CD45high CD11c+ MHCII+ XCR1-

MOUSE CELLS (Lymphoid panel):
Total T cells : CD45+ CD4+/CD8+
CD4 T cells : CD45+ CD4+ CD8-
CD8 T cells : CD45+ CD8+ CD4-
NK cells : CD45+ CD4-/CD8- NK1.1+
NKT cells : CD45+ CD4+/CD8+ NK1.1+
Naive CD8 T cells : CD45+ CD8+ CD4- CD44low CD62Lhigh
CD8 T effector : CD45+ CD8+ CD4- CD44high CD62Llow
CD8 T central memory : CD45+ CD3+ CD8+ CD44high CD62Lhigh
Naive CD4 T cells : CD45+ CD4+ CD8- CD44low CD62Lhigh
CD4 T effector : CD45+ CD8- CD4+ CD44high CD62Llow
CD4 T central memory : CD45+ CD8- CD4+ CD44high CD62Lhigh

☒ Tick this box to confirm that a figure exemplifying the gating strategy is provided in the Supplementary Information.
