# Supplementary material for: Perioperative myeloid cell remodeling shapes CAR-T cell efficacy in glioblastoma
Source: Nat Commun. 2026 Jul 31;17:9727. doi: 10.1038/s41467-026-76206-y (PMC13562681; doi:10.1038/s41467-026-76206-y)
Supplement: Supplementary file 3 [file 41467_2026_76206_MOESM3_ESM.pdf]

nature portfolio

Peer Review File

Perioperative myeloid cell remodeling shapes CAR-T cell efficacy in glioblastoma

Corresponding Author: Dr Martin PEDARD

This file contains all reviewer reports in order by version, followed by all author rebuttals in order by version.

Version 0:

Reviewer comments:

Reviewer #1

(Remarks to the Author)
The study by Pedard et al. investigates immune microenvironment changes in two murine glioma models (SB28 and CT2A) in both unresected and post-operative settings. Using in vivo experiments, the authors characterize immune differences between these models and report an increase in TREM2⁺ myeloid populations associated with early immunosuppressive programs, followed by evidence of lymphoid exhaustion in post-operative conditions. Based on these observations, the authors explore multiple immunomodulatory strategies, including targeting TREM2⁺ cells using CAR approaches, GD2 CAR-T therapy, and immune checkpoint blockade in neoadjuvant and adjuvant settings.

The study addresses an important question regarding the timing and cellular targets of immunotherapy in glioma and presents several interesting observations. However, several issues limit the strength of the conclusions. In particular, the number of independent biological repeats for several key in vivo experiments is not clearly indicated, and the survival benefits reported in the TREM2-CAR experiments appear modest (approximately 2–5 days). Given the experimental and clinical heterogeneity of glioblastoma, some of the therapeutic claims may therefore be overstated. Clarification of experimental replication, improved methodological transparency, and a more cautious interpretation of the findings would strengthen the manuscript.

Line 100–101: For Supplementary Fig. 1b–d, inclusion of a gating strategy would help clarify the definitions of the indicated immune cell subsets. In the presented mouse datasets, TREM2 expression appears abundant in TAMs, MDSCs, and cDC1 populations but relatively scarce in microglia. This contrasts with human datasets where TREM2 is typically enriched in microglia. A gating strategy panel for TREM2⁺ cells across all subsets, particularly in Supplementary Fig. 1d, would therefore be helpful. In addition, please clarify whether the results represent pooled data or independent experimental repeats. Discussion of this apparent discrepancy between murine and human TREM2 expression patterns and its implications would strengthen the manuscript.

Line 107–108: Please indicate how many independent biological repeats were performed for the survival experiments.

Line 122–123: It may be informative to determine whether the increase in TREM2⁺ cells correlates with tumor burden (e.g., BLI signal) across CT2A and SB28 models. Such analysis could help clarify whether the expansion of TREM2⁺ cells reflects tumor burden rather than a distinct immunological program.

Line 213 (Fig. 3b): Survival appears only modestly prolonged. Please indicate how many independent experiments were performed. It would also be helpful to clarify whether this trend is reproducible, particularly since Fig. 3g appears to show minimal differences between UTD and TREM2-CAR groups.

Line 215–216: The manuscript reports increased neutrophil infiltration following targeting with TREM2 CAR-T cells. Please clarify how many times the experiments shown in Fig. 3g–h were repeated and provide further explanation for this observation.

Line 231: Increased neutrophil infiltration following TREM2 CAR-T therapy may reflect clearance of dying TREM2⁺ myeloid cells after CAR-mediated killing rather than a TREM2-specific immunological mechanism. Tissue neutrophilia is commonly observed following cytotoxic interventions and may represent a general response to cell death. This possibility should be

considered in the interpretation.

Line 248 (Fig. 4b): Please indicate how many independent experimental repeats were performed.

Line 259–261: It may be worthwhile to explore cell type–specific targeting of TREM2⁺ populations (e.g., microglia versus tumor-associated macrophages). Such approaches could help distinguish potentially protective versus pathogenic roles of TREM2 depending on the cellular compartment.

Although several immunotherapy strategies were explored in murine glioma models, the manuscript could benefit from validation in a glioma stem cell–based model that more closely reflects human GBM pathology. Replicating key findings in neoadjuvant and adjuvant settings using TREM2-CAR, TREM2-CAR combined with GD2 CAR-T, and combinations with immune checkpoint blockade would strengthen the translational relevance.

Discussion: The discussion would benefit from additional context in several areas:
a) comparison of TREM2⁺ myeloid populations observed in this murine study with published human datasets;
b) key differences between murine glioma models and human GBM immune microenvironments;
c) implications of targeting TREM2⁺ cells for phagocytosis and antigen presentation, which are central functions associated with TREM2 signaling.

Minor Comments

Line 86–89: The combined statement of hypothesis and rationale is somewhat difficult to follow and could benefit from clearer phrasing.

Supplementary Figure 3: Please include labels identifying SB28 and CT2A datasets in the bar graphs.

Throughout the manuscript, please clearly indicate the number of independent biological repeats or whether datasets represent pooled data.

Provide gating strategies distinguishing TREM2⁺ and TREM2⁻ populations where applicable and reference them in the main text.

Supplementary Figure 20: For MHCII and CD206 staining, please include FMO controls, particularly given the continuous distribution of CD206 expression.

(Remarks on code availability)
The authors has provided the required README , R script and example data files. However, running the code on example data is giving errors. The authors are suggested to make following changes to the code:

1. On line 17 of R script, change the path of 'img_dir' to "Example data/" folder provided. At differs instances, different paths are given in the script and git hub repository.
2. Debug the R script between lines 225 - 229, as the command is unable to parse the Example data/ input file names and generates empty 'dtp' variable (0 rows and 6 columns)
3. In Usage section, provide names and description of all 14 output files generated.

Reviewer #2

(Remarks to the Author)
Pedard et al., present a preclinical evaluation of the GD2 CAR T cell efficacy with use of in vivo syngeneic mouse models and patient tumor tissue slice cultures. The authors confirm that surgical resection may lead to increased immunosuppression in the remaining tumor tissue. They suggest that neoadjuvant CAR T cell administration may provide better benefit than an adjuvant setting.

Several points need to be addressed:
1. The overall translational claim of the work at the end of each result section and in the discussion should be revised. While the work shows that TREM2 CAR T cells deplete TREM2⁺ myeloid cells and recruit neutrophils in vivo, the anti-tumor activity of GD2-CART cells is minimal, based on minimal impact on the survival (Fig 4). Combined TREM2-CAR T cell + GD2 CAR T cell treatment appears to show minimal responses when combined with the surgery, with variable statistical outcomes (Fig. 6 significant, Fig 5 non-significant). Actually, anti-PD1/anti-TIM3 alone shows a higher impact on the survival in the assessed model (Fig 5), though non-curative as well. Later, the authors show that GD2 CAR T cells work better in the neoadjuvant application and TREM2 CAR T cells do not add the benefit. Interestingly, this regimen showed minimal advantage for approx. 70% of the neoadjuvant treated mice, and remaining 30% appeared to be cured (Fig 7). While the authors analyse in depth the immune compartment, no information is provided about the overall tumor structure and the impact on the follow-up surgery in the treatment experiments presented. E.g., do GD2 CAR T cells in the adjuvant setting attack the tumor core and borders? Is tumor structure or invasion capacity affected, allowing for more extensive surgical intervention in 30% of mice? Or do authors expect the systemic or local anti-tumor immunisation? Since the surgery likely removes the majority of the intratumoral myeloid cells and injected GD2 CAR T cells, the potential mechanism needs to be assessed in further detail.

2. Selection of GD2 as the target is questionable. The mouse models were modified to express GD2 artificially, while no information is available for the applied patient tumor tissue samples. The GD2 expression in patient samples should be presented and discussed.
3. The extent of the surgical resection in vivo and ex vivo should be further evaluated and discussed:
- It is unclear if the TREM+ myeloid cells are increased due to activation of the marker expression of pre-existing cells, or influx of the 'fresh' monocytes or microglia to the tissue surrounding the resection cavity.
- Lines 143- 148 and 171-183 - the ex vivo platform was used for validating in vivo findings. Importantly, the authors should comment on the lack of blood flow in patient tumor brain slices, since this limits the influx of the monocytes and T cells from the blood. It is unclear if the changes in the myeloid compartment are linked to the influx of normal brain-residing microglia or 'left-over' TAMs.
- Where do the T cells (particularly T naïve cells) come from 24h post-resection? Tumor borders typically left after the surgery are known to contain few T cells. Do the authors expect the influx from the circulation or reactivation of left-over TILs? Since human brain tissue slices showed relevant changes, the latter could be hypothesized.
- The in vivo tumors are analysed 7 days after the resection in the majority of experiments. It is unclear whether the tumor growth had already recovered and the resection cavity was resolved at this stage. It should be analysed how long the impact of the resection is retained. It is possible that the assessed differences result in the removal of the main tumor mass and assessment of the 'left-over' myeloid compartment in the more invasive tumor niche, thus the direct impact of the surgery should be assessed with caution, or the composition of the myeloid cells should be assessed in different tumor areas at the moment of the surgery.
- Neoadjuvant treatment requires intracranial injection to the developed tumor, which also may create a local wound healing response. However, since the main tumor mass has not been removed, the pool of myeloid cells/TILs and their responses may differ. These responses should be assessed correctly in order to claim the advancement of neoadjuvant treatment.

4. The main difficulty in assessing the obtained responses is the myeloid subset discrimination strategy and nomenclature presented by the authors. The discrimination of the myeloid subsets is not based on the commonly applied discriminations, unclear and inconsistent across the manuscript. In general many of the myeloid subsets are not well-defined in the gating strategy or another name is used, creating confusion. Moreover, flow graphs should display the fluorescence intensity recorded, to compare the expression levels across the gating strategies. E.g.:
- Lines 54-57 and definitions in 100-101: the authors should correct the description of the myeloid compartment and clarify the various origins of TAMs (microglia, monocytes, border-associated macrophages). In general, both microglia and blood monocytes (including MDSCs) contribute to the pool of so-called TAMs. Microglia, when in contact with GBM cells, upregulate CD45, thus becoming part of the 'macrophage' population in the gating presented.
- The nomenclature 'resting' TAMs, monocytes and MDSCs is unclear (lines 332). While microglia can be in a homeostatic 'resting' state, the terminology does not fit to other myeloid cells in this context.
- In general gating strategy and association of certain myeloid cells is unclear or not correct. E.g. :
CD206+ cells were previously annotated as border-associated macrophages (e.g. doi: 10.1038/s41593-020-00789-y, 10.1038/s41467-021-21407-w, 10.1101/2023.03.05.531162). Here the authors divided them into microglia and macrophage subsets.
'CD206+ microglia/macrophages' are also positive for MHCII.
Ly6G expression in MDSCs appears more as a background, compared to Ly6G positivity in neutrophils.
Ly6ChighLy6Ghigh neutrophil term is applied in the text, while gating indicates granulocytes.
- TREM2 positivity is accessed in general as % of CD11b+ (mouse) or %CD68+ (human) cells, which corresponds to the general myeloid pool. Suppl Fig 4 shows consistent activation only in subsets defined as microglia. It is surprising to see differences in the model with regard to certain myeloid subsets, expected to be similar across the mouse brains e.g. In SB28 'resting microglia' expresses TREM2 in approx. 20% of cells, whereas in CT2A it is 100%. These differences further question the gating strategy and applied nomenclature.

(Remarks on code availability)

Reviewer #3

(Remarks to the Author)
Using two immunocompetent orthotopic glioma mouse models and a human glioblastoma organotypic slice model, the authors demonstrate an early induction of TREM2+ myeloid cells and a late induction of exhausted lymphocytes following partial tumor aspiration. Subsequently, they generated TREM2-targeting CAR T cells and evaluated their anti-tumor efficacy in vitro and in vivo. While these cells showed minimal anti-tumor activity as a monotherapy, they had synergistic activity when combined with GD2 CAR T cells. Additional immune checkpoint inhibition did not further enhance survival and neoadjuvant therapy proved was more effective than adjuvant administration.

This is a comprehensive and timely study that provides valuable insights into the effects of dual myeloid/lymphoid targeting and the critical importance of therapeutic timing.

Major

Is the resection-mediated change tumor-specific, or would similar immunological shifts occur following a sham resection of a healthy, non-tumor-bearing brain?

Have the authors characterized the functional or exhaustion states of the CAR T cells when administered in a neoadjuvant versus adjuvant setting? Analyzing the T-cell state would provide essential mechanistic insights alongside the observed changes in bystander immune cell populations.

In Figure 3b, the Kaplan-Meier curves cross each other. Are the authors certain that the reported differences remain statistically significant despite this crossing? Please reassess and double-check

(Remarks on code availability)

Reviewer #4

(Remarks to the Author)
In this manuscript Pedard et al. address the clinically relevant question of how surgical resection reshapes the GBM tumor microenvironment (TME) and thereby affects CAR T cell efficacy. Using two syngeneic mouse GBM models, the authors have comprehensively explored the effect of surgical resection on myeloid cells, specifically TREM2+ TAMs, and evaluated the efficacy of TREM2+ CAR T cells combined with GD2 CAR T cells. This study represents a tremendous amount of work and employs quite technically challenging mouse models to understand the impact of surgical resection on the TME. Overall, the data are well presented and support the author's conclusion. Further, the finding that neoadjuvant CAR T cell therapy is superior to adjuvant CAR-T treatment could be important for the design of future clinical trials.

The manuscript would be further strengthened by addressing the following points:

1. The study identified interesting associations between surgical resection, TREM2+ myeloid cells, and T cell exhaustion marker expression, but the mechanism is poorly defined. Do the authors propose that TREM2+ myeloid cells drive T cell exhaustion or that both occur due to a perioperative inflammatory response? Additional studies to support this association would strengthen the paper. Alternatively, authors should temper statements that may imply a direct causal link. For example, the abstract states "marked upregulation of TREM2 in myeloid cells and the subsequent emergence of T cell dysfunction" seems to imply that TREM2+ macrophages contribute to dysfunction.

2. For Figure 1: It is somewhat surprising that 'resting microglia' represent the population most increased following tumor resection. Please comment on this observation. Also, in Fig 1d, please clarify which specific myeloid population corresponds to the highlighted TREM2+ population, as Supp Fig 4 indicates that TREM2 expression is increased across multiple myeloid subsets. Also the title for Figure 1 refers to "delayed myeloid remodeling," yet the data show changes as early as 24 hrs post resection.

3. The claim that resection induces T cell exhaustion/dysfunction seems overstated. The study relies mainly on PD1/Tim3/Lag3 expression by flow cytometry, which is insufficient to define exhaustion. The authors should either soften the language throughout (e.g. exhaustion-like phenotype etc) and/or provide additional supporting data (e.g. transcriptional profiling showing exhaustion signatures and/or functional readouts).

4. The human GBM organotypic resection model is quite novel, and it is notable that this ex vivo injury also results in increased TREM2+ myeloid cells and an exhaustion-like T cell phenotype. Can the authors comment on the mechanism underlying these changes in this slice model? Specifically, the data seem more consistent with local induction/upregulation of TREM2 and exhaustion markers versus recruitment of cells. Authors should comment on this, and if possible, provide supporting data regarding induction versus migration. Additionally, please also clarify whether the organotypic replicates are technical replicates (GBM slices from the same patient) or biological replicates (independent patients), and provide basic clinical annotations for the GBM samples, i.e. newly diagnosed versus recurrent, IDH-status or other available features.

5. There are general statistical concerns as some of the statistical comparisons seem questionable. Please verify stats for Figure 3b and 5b. Log-rank test is indicated in figure legend, but please provide more details.

6. While the methods provide details for the TREM2 CAR, the GD2 CAR design is not described with similar detail. Please include a schematic and/or a table summarizing both CAR constructs, including scFv clone, spacer/hinge, transmembrane, costim, and any tags or other features.

7. Please provide expression levels for the GD2-engineered SB28 and CT2A lines.

8. The gating strategy for the $CD45.1^{+}$ population in Figure 4j appears to include some $CD45.1^{-}CD45.2^{-}$ cells, which may represent GFP^{+} tumor cells. This raises the possibility that the CFSE-high population in the UTD group may contain residual tumor cells. A more stringent gating strategy should be used to exclude tumor cells.

9. Fig. S12 shows that the addition of TREM2+ CAR, compared to UTD T cells, increases IFNγ production by GD2 CAR T cells. This observation is consistent with the findings by Sun et al. (PMID: 37172094), which reported that TREM2- knockout microglia exhibit enhanced responsiveness to IFNγ. However, Fig S12b also shows that SB28-CM-conditioned treated BMDM suppresses IFNγ secretion. It would be helpful to clarify how the proportion of TREM2+ macrophages change following SB28-CM and after TREM2 CAR treatment. This could help determine whether changes in IFNg secretion

correlate with modulation of depletion of TREM2+ macrophages.

10. Prior studies have investigated the role of TREM2 in the TME of gliomas. As referenced in the manuscript (refs 31-33), Zheng et al and Zhong et al suggest that TREM2+ myeloid cells may promote anti-tumor immune responses, whereas Sun et al reports findings more similar to this study, showing that TREM2+ TAMs are immunosuppressive and that targeting this population may be beneficial. Interestingly, Zhong et al used GL261 and CT-2A model, Zheng et al. used GL261; Sun et al. used SB28 and this study used SB28 and CT-2A models. While GL261 has been considered as immune "hot" tumor and may not fully recapitulate the human GBM TME, Zhong et al also reported that TREM2 knockout promotes CT2A tumor growth, indicating an anti-tumor role of TREM2+ TAM. This appears to be inconsistent with the conclusions of the current study. Given the different effects observed when targeting TREM2+ myeloid cells in the neoadjuvant vs adjuvant setting and these other published results, the authors are encouraged to more fully discuss these discrepancies, and to comment on possible explanations, i.e., model specific TME context, timing, and/or methodologies.

11. In the discussion, the authors claim that resection induces "myeloid-dominant immunosuppressive programs centered on TREM2+ cells." However, the manuscript does not exhaustively evaluate myeloid functional states and marker expression or establish that TREM2 cells are the primary drivers of immunosuppression. These statements should be qualified throughout the manuscript.

Minor comments:
1. It was a bit challenging to follow how cell populations were defined by flow cytometry, and required going to the gating strategy in the supp figures. Please add a Table summarizing marker panels used to define the myeloid and lymphocyte populations.
2. Figure 1g; Unresected panels are titled as D1/7 post-resection. This seems to be a mislabeling.
3. Figure 5b please check groups, as it seems that TREM2/GD2 CAR+isotype and UTD+PD1/Tim3 may be switched.
4. Figure legends need proofreading. Illustrations of Fig 3j, Fig 4e-4k and Fig S8c-d are missing, and the legend of Fig 4a is duplicated with that of Fig 5a.

(Remarks on code availability)

Reviewer #5

(Remarks to the Author)
I co-reviewed this manuscript with one of the reviewers who provided the listed reports. This is part of the Nature Communications initiative to facilitate training in peer review and to provide appropriate recognition for Early Career Researchers who co-review manuscripts.

(Remarks on code availability)

Reviewer #6

(Remarks to the Author)
I co-reviewed this manuscript with one of the reviewers who provided the listed reports. This is part of the Nature Communications initiative to facilitate training in peer review and to provide appropriate recognition for Early Career Researchers who co-review manuscripts.

(Remarks on code availability)

Version 1:

Reviewer comments:

Reviewer #1

(Remarks to the Author)
The authors have satisfactorily addressed my concerns raised in the first submission. specifically, regarding the TREM2+ neutrophil population and the increased neutrophil infiltration observed following CAR-T treatment in Figure 3. The clarification that these cells exhibit an inflammatory (iNOS+) phenotype helps explain the observation and adds important context. Overall, the experiments are well executed and the data presented clearly, the conclusions are supported by the results, and the authors have been appropriately cautious in their interpretation of the findings. At this stage, I do not feel that additional experiments are needed to address this point.

Krishna Bhat

(Remarks on code availability)

The authors has provided the required README , R script and example data files. However, running the code on example data have one minor issue. The authors are suggested to fix the code:
1. R script run: line 260 is giving error.

CODE: if (!nrow(grp)) stop("Pas de triplet complet (C0 DAPI, C3 TREM2, C1/C2 GFP).")

ERROR: Pas de triplet complet (C0 DAPI, C3 TREM2, C1/C2 GFP).

COMMENT: This error is because of naming inconsistency of raw files. The DAPI file name is missing the descriptive suffix that the other channel file names have.

FIX: (1) For the given code, change input file name from "Anonymous.nd2 (series 12) - C=0.jpg" to "Anonymous.nd2 - Unresected 1-2.nd2 (series 12) - C=1.jpg"
(2) If there are multiple base images with unique series_id, then fix the code for grouping using series_id.

Reviewer #2

(Remarks to the Author)
I would like to thank the authors for providing the detailed response to my comments.
I have only one remaining concern. I acknowledge that flow cytometry based discrimination of myeloid cell subsets is challenging in the GBM context. I accept the CD206/MHCII nomenclature applied in the revised version. However, it would be important to uniform throughout the text that CD45high cells (as defined in the reply to the reviewer: TAM (CD45high $F4/80^{+}$ CD11b+)),contain both microglia and monocyte-derived tumor-associated macrophages. My recommendation would be to refer to this subset generally as TAMs uniformly in the manuscript.

(Remarks on code availability)

Reviewer #3

(Remarks to the Author)
The authors carefully revised the manuscript and addressed all reviewer points. The manuscript can be accepted for publication.

(Remarks on code availability)

Reviewer #4

(Remarks to the Author)

(Remarks on code availability)
The authors have adequately addressed all my concerns, and I appreciate the responsiveness of the revised manuscript to review comments.

Reviewer #6

(Remarks to the Author)
I co-reviewed this manuscript with one of the reviewers who provided the listed reports. This is part of the Nature Communications initiative to facilitate training in peer review and to provide appropriate recognition for Early Career Researchers who co-review manuscripts.

(Remarks on code availability)

Open Access This Peer Review File is licensed under a Creative Commons Attribution 4.0 International License, which permits use, sharing, adaptation, distribution and reproduction in any medium or format, as long as you give appropriate credit to the original author(s) and the source, provide a link to the Creative Commons license, and indicate if changes were made.
In cases where reviewers are anonymous, credit should be given to 'Anonymous Referee' and the source.
The images or other third party material in this Peer Review File are included in the article's Creative Commons license, unless indicated otherwise in a credit line to the material. If material is not included in the article's Creative Commons license and your intended use is not permitted by statutory regulation or exceeds the permitted use, you will need to obtain permission directly from the copyright holder.
To view a copy of this license, visit https://creativecommons.org/licenses/by/4.0/

REVIEWER COMMENTS

Reviewer #1 (Remarks to the Author):

The study by Pedard et al. investigates immune microenvironment changes in two murine glioma models (SB28 and CT2A) in both unresected and post-operative settings. Using in vivo experiments, the authors characterize immune differences between these models and report an increase in TREM2$^+$ myeloid populations associated with early immunosuppressive programs, followed by evidence of lymphoid exhaustion in post-operative conditions. Based on these observations, the authors explore multiple immunomodulatory strategies, including targeting TREM2$^+$ cells using CAR approaches, GD2 CAR-T therapy, and immune checkpoint blockade in neoadjuvant and adjuvant settings.

The study addresses an important question regarding the timing and cellular targets of immunotherapy in glioma and presents several interesting observations. However, several issues limit the strength of the conclusions. In particular, the number of independent biological repeats for several key in vivo experiments is not clearly indicated, and the survival benefits reported in the TREM2-CAR experiments appear modest (approximately 2–5 days). Given the experimental and clinical heterogeneity of glioblastoma, some of the therapeutic claims may therefore be overstated. Clarification of experimental replication improved methodological transparency, and a more cautious interpretation of the findings would strengthen the manuscript.

We thank Reviewer 1 for this thoughtful and constructive summary of our work. We address each specific concern raised in this general comment and in the subsequent point-by-point critique below.

Regarding experimental replication and methodological transparency, we have systematically reviewed all key experiments and updated the Results section and figure legends to explicitly state the number of independent replicates for each experiment. Specific details are provided in our responses to each individual point below.

As a general point, we wish to highlight that the neoadjuvant GD2 CAR T cell experiment (**Fig. 7 and Supplementary Fig. 16**), which represents one of the central findings of the manuscript, was performed in two independent biological replicates in both the SB28 and CT2A models (both from the initial version of the manuscript), yielding consistent results across cohorts concerning survival analysis. This has been added to the relevant Results section and figure legends as follows:

(**i**) In the Results text (**lines 328-331**), the sentence describing the neoadjuvant experiment now reads: "*Across both models, the neoadjuvant CAR-T cell approach significantly improved antitumor responses and survival compared to adjuvant CAR-T cells or surgery alone (Fig. 7b,c, Supplementary Fig. 16a,b, two independent experiments in each model).*"

(**ii**) In the legend of Fig. 7b (**lines 1247-1250**), the following has been added and highlighted in yellow: "***(b)*** *Kaplan-Meier survival analysis of resected SB28-bearing mice treated or not with adjuvant or neoadjuvant GD2 CAR-T cells pooled from two independent experiments (n=16-17 mice per group; log-rank test). mOS=median overall survival.*"

(**iii**) In the legend of Supplementary Fig. 16a,b, the same clarification has been added for the CT2A model.

Line 100–101: For Supplementary Fig. 1b–d, inclusion of a gating strategy would help clarify the definitions of the indicated immune cell subsets. In the presented mouse datasets, TREM2 expression appears abundant in TAMs, MDSCs, and cDC1 populations but relatively scarce in microglia. This contrasts with human datasets where TREM2 is typically enriched in microglia. A gating strategy panel for TREM2$^+$ cells across all subsets, particularly in Supplementary Fig. 1d, would therefore be helpful. In addition, please clarify whether the results represent pooled data or independent experimental repeats.

Discussion of this apparent discrepancy between murine and human TREM2 expression patterns and its implications would strengthen the manuscript.

Gating strategy for TREM2$^+$ cells.

We thank the reviewer for this comment. The gating strategy for TREM2$^+$ cells is now illustrated in **Supplementary Fig. 1d**, where the top row displays the stained samples and the bottom row the corresponding FMO controls across all myeloid subsets. Gates were systematically defined based on FMO controls. The following changes have been made in the revised manuscript:

(**i**) Supplementary Fig. 1 has been updated with the addition of panel (**d**), showing stained samples (top row) and FMO controls (bottom row) for TREM2 gating across all myeloid subsets.

(**ii**) The legend of Supplementary Fig. 1 has been updated to include a description of the new panel: "***(d)*** *Representative flow cytometry plots showing TREM2-stained samples controls (top row) and Fluorescence Minus One (FMO) (bottom row) for each indicated myeloid cell subset. Gates defining TREM2$^+$ populations were set based on FMO controls.*"

(**iii**) The Methods section (**Flow cytometry, lines 769-770**) has been updated with the following sentence: "*TREM2 positivity thresholds were defined using Fluorescence Minus One (FMO) controls for each myeloid subset independently, as illustrated in Supplementary Fig. 1d.*"

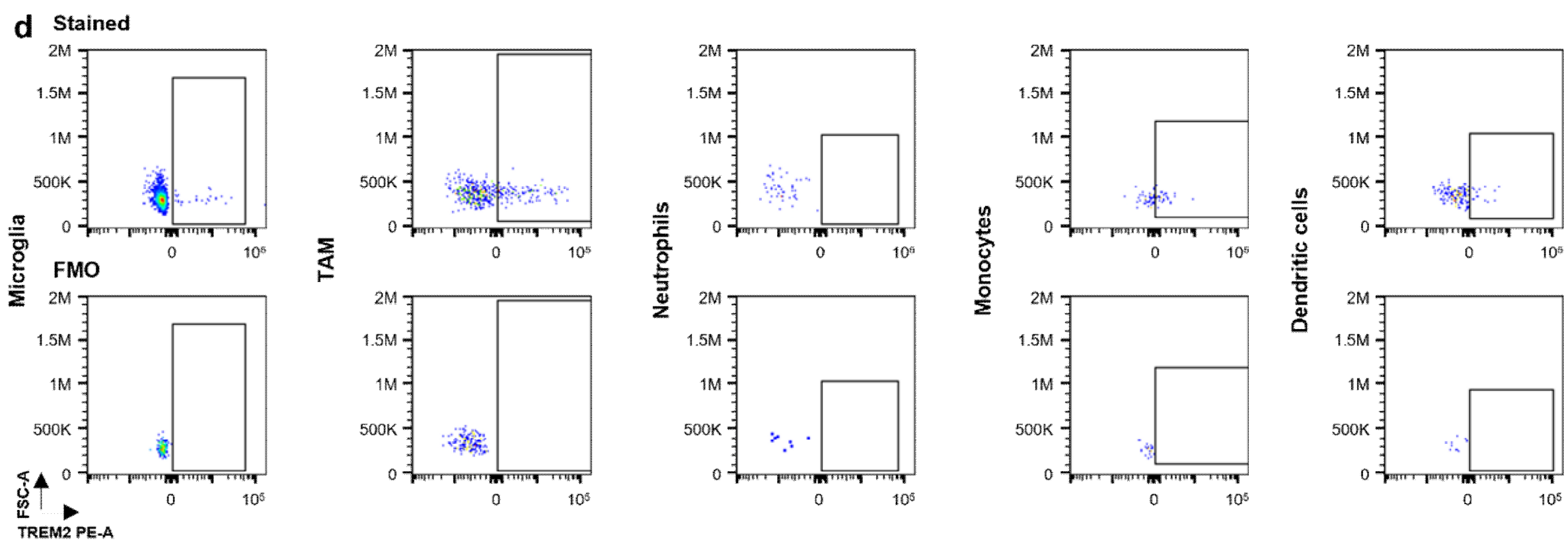

Supplementary Fig. 1d. *Representative flow cytometry plots showing TREM2-stained samples (top row) and Fluorescence Minus One (FMO) controls (bottom row) for each indicated myeloid cell subset (microglia, macrophages, monocytes, neutrophils, dendritic cells). Gates defining TREM2$^+$ populations were set based on FMO controls.*

Pooled data *versus* independent experimental repeats.

The data presented in Supplementary Fig. 1b-d represent a single baseline characterization experiment (n=5 mice per model per group) performed on day 7 post-implantation, prior to any intervention. This is a baseline phenotyping experiment and was therefore not repeated as an independent biological replicate. This is now explicitly stated in the revised figure legend.

TREM2 expression across myeloid subsets: murine *versus* human patterns.

The reviewer raises an important point regarding the apparent discrepancy between murine models (where TREM2 appears abundant in TAMs and cDC1 but relatively scarce in microglia) and human datasets (where TREM2 is typically enriched in microglia). To directly address this, we performed a cross-species analysis of TREM2 expression using publicly available single-cell RNA sequencing datasets from SB28 and CT2A murine models and human GBM specimens (new **Supplementary Fig. 2**).

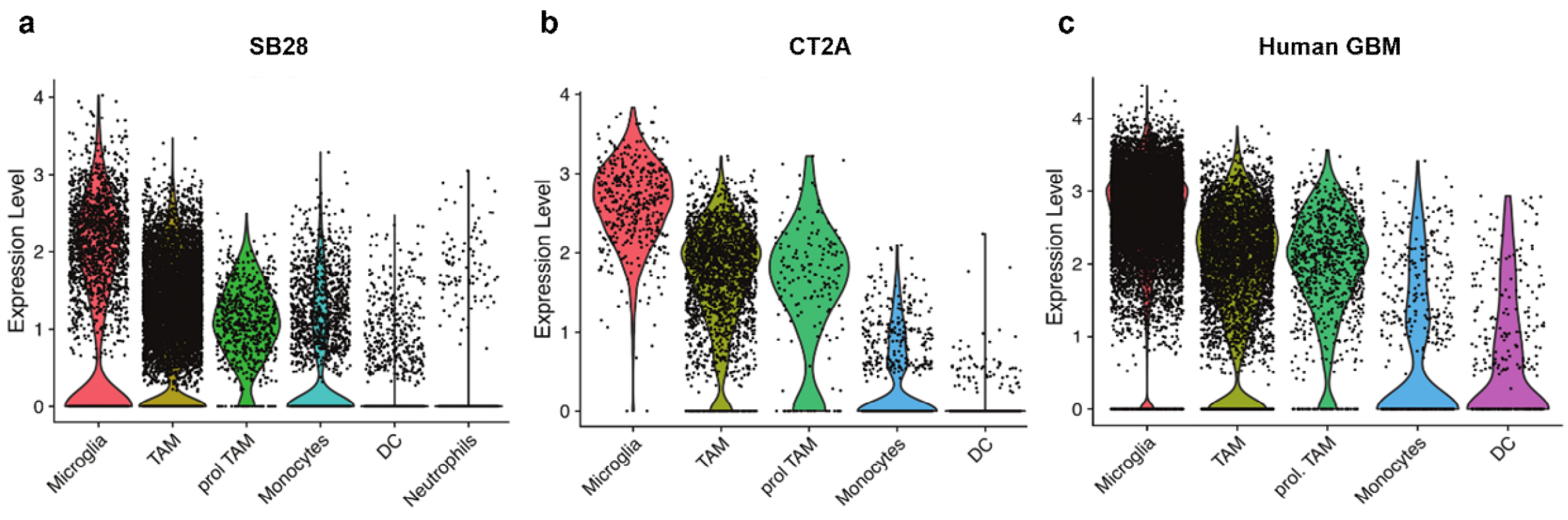

Supplementary Fig. 2 TREM2 expression across myeloid cell subsets in murine and human GBM by single-cell transcriptomic analysis. *Violin plots showing TREM2 (murine) or TREM2 (human) transcript expression levels across annotated myeloid cell populations in publicly available scRNAseq datasets from SB28 (**a**), CT2A (**b**), and human GBM (**c**) tumors. Each dot represents an individual cell. Populations shown include microglia, tumor-associated macrophages (TAM), proliferating TAM (prol TAM), monocytes, dendritic cells (DC), and neutrophils (SB28 only).*

As shown in the new **Supplementary Fig. 2a,b** (murine) and **2c** (human), TREM2 transcript expression was detected across multiple myeloid populations including microglia, macrophages, monocytes, and dendritic cells in both species. These transcriptomic data are broadly consistent with our flow cytometry observations and demonstrate that TREM2 expression is not restricted to a single myeloid lineage in either species.

The following changes have been made to the revised manuscript:

In the Results (**lines 100-104**), the following has been added:

" *To complement these flow cytometry observations, we analyzed TREM2 expression across myeloid cell subsets using publicly available single cell RNA sequencing (scRNAseq) datasets from SB28 and CT2A. Consistent with flow cytometry data, TREM2 transcripts were detected across multiple myeloid cell populations, including microglia, TAM, monocytes, and DC, in both models (**Supplementary Fig. 2a,b**).*"

In the Results (**lines 141-145**), the following has been added:

"*Prior to translating these observations to human tissue, we verified that TREM2 expression was distributed across comparable myeloid cell subsets in human GBM. Analysis of publicly available scRNAseq data from human GBM specimens confirmed TREM2 expression in microglia, TAMs, monocytes, and dendritic cell populations (**Supplementary Fig. 2c**), supporting the relevance of our murine findings to the human setting.*"

In the Discussion (**Lines 452-467**), the following has been added:

"*These findings in murine models are broadly consistent with human GBM single-cell transcriptomic datasets, which have identified a TREM2^{high} macrophage state enriched in TAM, characterized by lipid metabolism gene signatures, immunosuppressive signaling, and spatial association with hypoxic tumor regions[8,49]. The cross-species scRNAseq analysis presented here confirmed that TREM2 transcripts are broadly distributed across microglia, macrophages, monocytes, and DC in both murine and human GBM, supporting conservation of TREM2 expression across myeloid cell lineages. Nevertheless, important differences between species warrant consideration. At the protein level, flow cytometry-based quantification suggests that TREM2 upregulation post-resection is most pronounced in infiltrating monocyte-derived populations in SB28 and CT2A, likely reflecting the greater contribution of blood-borne myeloid cells in rapidly growing syngeneic tumors. Additionally, human GBM harbors a more complex and heterogeneous myeloid cell landscape, including border-associated macrophages and*

ontogenically distinct TAM subpopulations[50]*, which may not be fully recapitulated in transplantable murine models. These caveats should be borne in mind when extrapolating our findings to the clinical setting.*"

In the Methods section, this new paragraph has been added as follows (**Lines 794-809**) "*Publicly available scRNA-seq datasets were obtained from three sources. CT2A data were retrieved from Zenodo (https://doi.org/10.5281/zenodo.6654420), as originally published by Tomaszewski et al.*[80]*. SB28 datasets (GSM8703639 and GSM8703640) were downloaded from the Gene Expression Omnibus (GEO), as described in Ugolini et al.*[81]*. Human GBM immune data, originally published by Pombo Antunes et al.*[8]*, were retrieved from the Brain Immune Atlas (https://www.brainimmuneatlas.org/). All analyses were performed in R (v4.5.1; R Foundation for Statistical Computing) using Seurat (v5.4.0). Data were processed following a standard workflow including quality control filtering, normalization (SCTransform), dimensionality reduction (PCA and UMAP), and graph-based clustering. Cell-type annotation was performed using differential gene expression analysis (FindAllMarkers function) integrated with original annotations from source publications where available. Myeloid cell populations were annotated using curated marker gene signatures: microglia (P2ry12, Ccl4, Tmem119, Igf1), tumor-associated macrophages (Cd68, Spp1, Mrc1), monocytes (Plac8, Chil3, Ccr2, Ly6c2), neutrophils (S100a8, S100a9), dendritic cells (Ccl22, Ccr7, Fscn1, Batf3, Cd36, Flt3, Ffar2, Clec10a), and plasmacytoid dendritic cells (Siglech, Ccr9, Bcl11a, Runx2). Gene expression distributions across annotated myeloid cell populations were visualized using violin plots (VlnPlot).*"

The following references have been added to the reference list:

80 Tomaszewski, W. H. *et al.* Neuronal CaMKK2 promotes immunosuppression and checkpoint blockade resistance in glioblastoma. *Nature communications* **13**, 6483 (2022). https://doi.org/10.1038/s41467-022-34175-y

81 Ugolini, A. *et al.* Functional Reprogramming of Neutrophils within the Brain Tumor Microenvironment by Hypoxia-Driven Histone Lactylation. *Cancer discovery* **15**, 1270-1296 (2025). https://doi.org/10.1158/2159-8290.Cd-24-1056

Line 107-108: Please indicate how many independent biological repeats were performed for the survival experiments.

We thank the reviewer for this comment. To address this point, we performed an additional independent survival experiment in both SB28 (**Fig. R1a**) and CT2A models (**Fig. R1b**). The initial manuscript included one survival experiment per model (SB28: n=7 mice per group; CT2A: n=7 mice per group). For this revision, a second independent experiment was added (SB28: n=4 mice per group; CT2A: n=4 mice per group), yielding a total pooled cohort of n=11 mice per group per model. Both individual experiments and the pooled analysis are presented in the new **Fig. R1**. The updated **Fig. 1b** and **Supplementary Fig. 3b** in the revised manuscript present the pooled survival curves (n=11 per group).

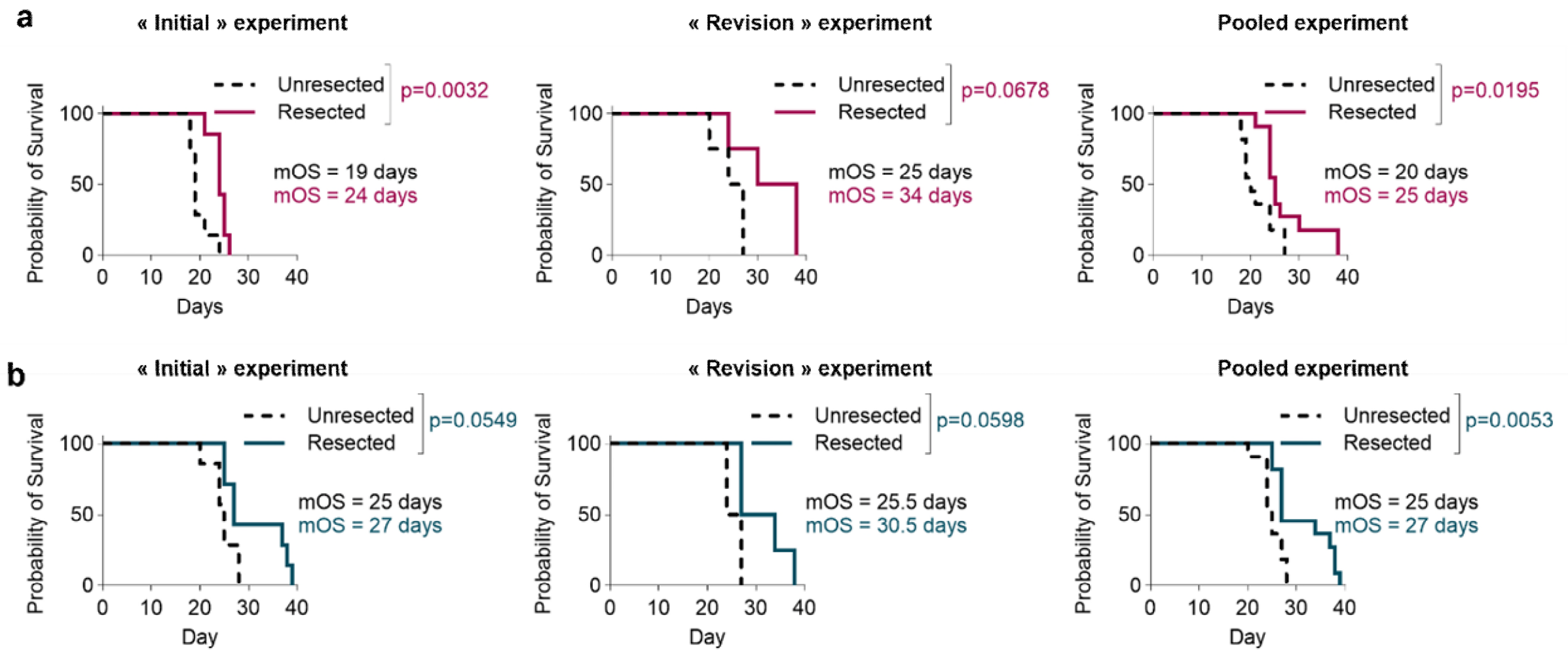

Figure R1. Surgical resection improves survival in SB28 and CT2A murine GBM models across two independent experiments. *(a) Kaplan–Meier survival curves for SB28 tumor-bearing mice comparing unresected (dashed black) and resected (magenta) groups. Left: initial experiment (n=7 mice per group; mOS 19 vs 24 days, p=0.0032, log-rank test). Middle: revision experiment (n=4 mice per group; mOS 25 vs 34 days, p=0.0678, log-rank test). Right: pooled analysis across both experiments (n=11 mice per group; mOS 20 vs 25 days, p=0.0195, log-rank test). (b) Kaplan–Meier survival curves for CT2A tumor-bearing mice comparing unresected (dashed black) and resected (teal) groups. Left: initial experiment (n=7 mice per group; mOS 25 vs 27 days, p=0.0549, log-rank test). Middle: revision experiment (n=4 mice per group; mOS 25.5 vs 30.5 days, p=0.0598, log-rank test). Right: pooled analysis across both experiments (n=11 mice per group; mOS 25 vs 27 days, p=0.0053, log-rank test). The pooled analyses shown in panels (a) and (b), right, correspond to the updated Fig. 1b and Supplementary Fig. 3b in the revised manuscript, respectively. mOS, median overall survival.*

The results and figure legends have been updated accordingly as follow:

Lines 108-109: "*This procedure induced substantial cytoreduction, extending median survival by five and two days in SB28 and CT2A models, respectively (**Fig. 1b, Supplementary Fig. 3b**, two independent experiments*)."

Lines 1099-1103: "*(**b**) Left to right: representative bioluminescence imaging (BLI) at days 9 and 11 post-implantation, longitudinal BLI quantification, comparison of BLI signal between days 10 and 11, and Kaplan–Meier survival analysis of unresected and resected mice pooled from two independent experiments (n=11 mice/condition; log-rank test). mOS=median overall survival.*

Line 122-123: It may be informative to determine whether the increase in TREM2$^+$ cells correlates with tumor burden (e.g., BLI signal) across CT2A and SB28 models. Such analysis could help clarify whether the expansion of TREM2$^+$ cells reflects tumor burden rather than a distinct immunological program.

We thank the reviewer for this suggestion. As shown in **Fig. 1f** and **Supplementary Fig. 3f** and described from lines 123 to 129, we have already assessed the correlation between tumor burden and TREM2$^+$ myeloid cell frequency in both SB28 and CT2A models. We observed a strong positive correlation between the proportion of TREM2$^+$ cells (% CD11b$^+$) and tumor growth (BLI D20/D11 or D20/D13), indicating that TREM2$^+$ expansion closely tracks tumor progression. These results suggest that the accumulation of TREM2$^+$ cells is largely tumor-driven rather than a distinct immunological response.

Line 213 (Fig. 3b): Survival appears modestly prolonged. Please indicate how many independent experiments were performed. It would also be helpful to clarify whether this trend is reproducible, particularly since Fig. 3g appears to show minimal differences between UTD and TREM2-CAR groups.

We thank the reviewer for this comment. The modest survival benefit observed in Fig. 3b is acknowledged in the manuscript, and we agree that replication is essential to assess reproducibility. We have therefore performed an additional independent biological replicate of the TREM2 CAR-T cell

versus UTD T cell experiment. Both individual experiments and the pooled analysis are presented in **Fig. R2** for full transparency. The initial experiment included n=10 mice per group; the revision experiment added n=7 mice per group, yielding a total pooled cohort of n=17 mice per group. Individual experiments and the pooled analysis (n=17 per group, log-rank test, p=0.027) confirms a significant survival benefit of TREM2 CAR over UTD T cell treatment, demonstrating reproducibility of the trend across two independent cohorts. The updated **Fig. 3b** in the revised manuscript presents the pooled Kaplan–Meier curves (n=17 per group), with the figure legend updated to indicate that data are pooled from two independent experiments.

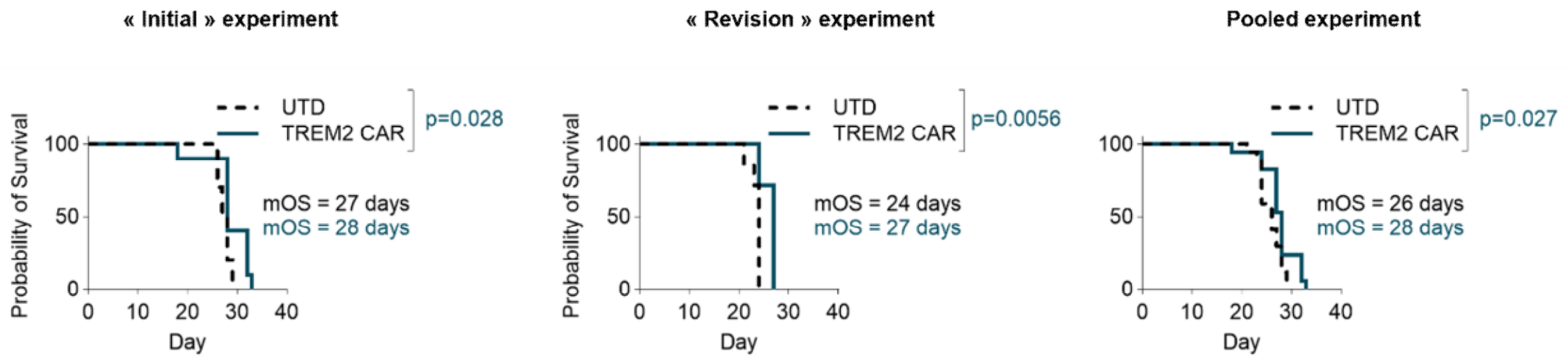

Figure R2. TREM2 CAR T cell treatment modestly but significantly improves survival in two independent experiments. *Kaplan–Meier survival curves comparing UTD (dashed black) and TREM2 CAR (teal) T cell-treated SB28 tumor-bearing mice. Left: initial experiment (n=10 mice per group; mOS 27 vs 28 days, p=0.028, log-rank test). Middle: revision experiment (n=7 mice per group; mOS 24 vs 27 days, p=0.0056, log-rank test). Right: pooled analysis across both experiments (n=17 mice per group; mOS 26 vs 28 days, p=0.027, log-rank test). The pooled analysis shown in the right panel corresponds to the updated Fig. 3b in the revised manuscript. mOS, median overall survival.*

We also wish to highlight that the UTD + isotype versus TREM2 CAR T + isotype arms within the neutrophil depletion experiment (**Fig. 3g**) constitute a third independent replicate of the same comparison, conducted in a distinct cohort of mice, further reinforcing the reproducibility of this finding. The figure legend for Fig. 3b and the relevant text have been updated to reflect that the experiment was performed in two independent biological replicates with consistent results as follows:

(**i**) Lines **223-234** "*TREM2 CAR-T cell administration delayed tumor growth compared to UTD T cells, and survival was slightly prolonged (**Fig. 3b, two independent experiments**).*"

(**ii**) Lines **1131-1133** "*(**b**) Left to right: Longitudinal bioluminescence imaging (BLI) quantification and Kaplan-Meier curves comparing the UTD and TREM2 CAR-T cell-treated groups pooled from two independent experiments (n=17 mice per group; log-rank test).*"

Line 215-216: The manuscript reports increased neutrophil infiltration following targeting with TREM2 CAR-T cells. Please clarify how many times the experiments shown in Fig. 3g–h were repeated and provide further explanation for this observation.

We thank the reviewer for this important comment, which we address in two parts.

To confirm the reproducibility of neutrophil infiltration following TREM2 CAR T cell treatment, we performed an additional independent biological replicate of the flow cytometry experiment (TREM2 CAR vs UTD T cells, 3 days post-resection). Both old individual experiment (**Fig. R3a, n=5 mice per group**) and the pooled analysis (**Fig. R3b, n=8 mice per group**) are presented in the new **Fig. R3** for full transparency. In both independent experiments, TREM2 CAR T cell treatment consistently induced a significant increase in Ly6G^{+} neutrophil infiltration compared to UTD T cell controls, confirming the reproducibility of this observation. The updated **Fig. 3c,d** in the revised manuscript present the pooled UMAP and bar graphs from both independent experiments combined, with the figure legend updated to indicate that data are pooled from two independent experiments (n=8 mice per group total).

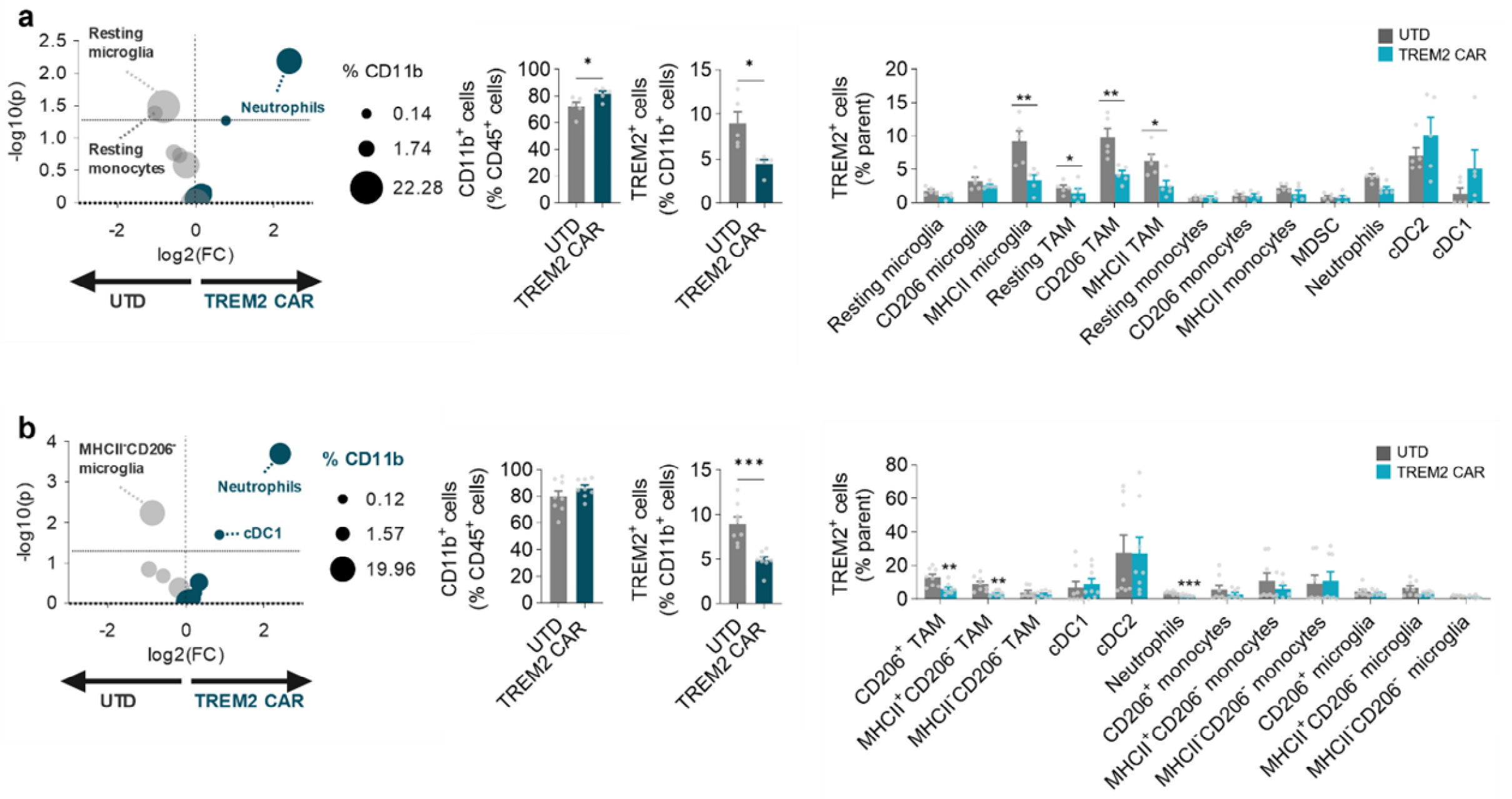

Figure R3. Reproducibility of TREM2 CAR T cell-induced myeloid remodeling and neutrophil infiltration across two independent experiments. Flow cytometry analysis of the myeloid compartment in resected SB28 tumors treated with UTD or TREM2 CAR T cells, collected 3 days post-resection. (**a**) First independent experiment (n=5 mice per group). (**b**) Pooled analysis of both independent experiments (n=8 mice per group).

The revised Results section (**lines 225-229**) now reads: "*Analysis of the TME three days after CAR-T cell injection revealed depletion of TREM2-expressing myeloid cell populations (**Fig. 3d, right panel**) notably TREM2^{+} TAM and TREM2^{+} neutrophils (**Supplementary Fig. 11b**). Remarkably, treatment with TREM2 CAR-T cells induced a loss of CD206^{-}MHCII^{-} microglia and a substantial neutrophil infiltration (**Fig. 3c,d, Supplementary Fig. 11a**).*" The figure legends for **Fig. 3c,d** and **Supplementary Fig. 11** have been updated to indicate that FACS analyses were performed in two independent experiments.

Regarding the anti-Gr1 neutrophil depletion experiment (**Fig. 3g,h**): this experiment was performed once (n=7-8 mice per group). We acknowledge this as a limitation and have explicitly stated it in the revised figure legend. We consider the finding to be mechanistically supported by two recent papers: (**i**) the pro-inflammatory phenotype of the recruited neutrophils, characterized by upregulation of iNOS and CD62L (**Fig. 3e**), is consistent with the antitumoral Sell^{hi} neutrophil subset described by Gungabeesoon et al. (Cell 2023, PMID 37001504), whose functional necessity in T cell-based immunotherapy has been independently validated (Hirschhorn et al., Cell, 2023, PMID: 37001503).

Regarding the mechanistic interpretation of neutrophil infiltration: We agree with the reviewer that the mechanism warrants further discussion and refer to our response to the next comment from the reviewer (below), where we have substantially expanded the interpretation. In brief, we now explicitly acknowledge that neutrophil recruitment following TREM2 CAR T cell treatment likely reflects two complementary mechanisms: (**i**) cytotoxic clearance of TREM2^{+} myeloid cells with associated DAMP release driving neutrophil recruitment, and (**ii**) a TREM2 CAR T cell-specific inflammatory remodeling of the myeloid cell niche. Critically, the functional necessity of neutrophils for therapeutic efficacy, demonstrated by abrogation of the survival benefit upon anti-Gr1 depletion, indicates that their recruitment is not merely a bystander response but an active contributor to antitumor immunity in this context.

Line 231: Increased neutrophil infiltration following TREM2 CAR-T therapy may reflect clearance of dying TREM2^{+} myeloid cells after CAR-mediated killing rather than a TREM2-specific immunological

mechanism. Tissue neutrophilia is commonly observed following cytotoxic interventions and may represent a general response to cell death. This possibility should be considered in the interpretation.

We thank the reviewer for raising this point. We agree that neutrophil recruitment following cytotoxic killing of TREM2$^+$ myeloid cells could in part reflect a general tissue response to cell death and DAMP-mediated clearance, as has been described in various contexts of tissue injury (Ramos et al., Cell Death Discov, 2024, PMID: 38218739). However, two lines of evidence argue against a purely passive clearance role in our setting. First, depletion of neutrophils with anti-Gr1 antibodies completely abrogated the survival benefit conferred by TREM2 CAR T cells (**Fig. 3g**), demonstrating that neutrophils are functionally required for therapeutic efficacy. Second, phenotypic characterization of recruited neutrophils revealed upregulation of iNOS (**Fig. 3e**), consistent with a pro-inflammatory, antitumor polarization rather than a tolerogenic clearance phenotype. These findings are consistent with two recent landmark studies demonstrating that T cell immunotherapies actively recruit antitumor neutrophil subsets with tumoricidal and antigen-presenting functions (Hirschhorn et al., Cell 2023 and Gungabeesoon et al., Cell 2023 [refs 35 and 36 in our manuscript]).

We have added a sentence to the Discussion (**lines 478-482**) to explicitly acknowledge the possibility of a DAMP-driven clearance component while emphasizing the functional evidence for an active antitumor role:

"*While DAMP-mediated clearance of dying TREM2$^+$ cells may partly contribute to neutrophil recruitment[56], the functional requirement for neutrophils in mediating therapeutic efficacy, as demonstrated by anti-Gr1 depletion, and their pro-inflammatory phenotype argue for an active antitumor role rather than passive tissue neutrophilia.*"

The following reference has been added to the reference list:

56 Ramos, C. & Oehler, R. Clearance of apoptotic cells by neutrophils in inflammation and cancer. *Cell Death Discov* **10**, 26 (2024). https://doi.org/10.1038/s41420-024-01809-7

Line 248 (Fig. 4b): Please indicate how many independent experimental repeats were performed.

The experiment shown in Fig. 4b was performed once (n=10 mice per group), which we now explicitly state in the figure legend. We acknowledge this as a limitation and did not repeat the experiment given animal ethics constraints.

Line 259–261: It may be worthwhile to explore cell type-specific targeting of TREM2$^+$ populations (e.g., microglia versus tumor-associated macrophages). Such approaches could help distinguish potentially protective versus pathogenic roles of TREM2 depending on the cellular compartment.

We thank the reviewer for this insightful suggestion. We fully agree that compartment-specific dissection of TREM2 function in microglia versus monocyte-derived tumor-associated macrophages (TAMs) would provide valuable mechanistic insights into the context-dependent roles of TREM2 in glioma.

Indeed, recent studies have highlighted divergent functions of TREM2 in glioma models. Sun et al. (Sci Adv 2023, PMID: 37172094) demonstrated that TREM2 inhibition via antisense oligonucleotide (ASO)-mediated knockdown or germline knockout in SB28 promotes IFNγ-driven myeloid cell reprogramming, including upregulation of Nos2, Cxcl10, and TNF signaling in tumor-associated macrophages and microglia, and enhances antitumor immunity, consistent with an immunosuppressive role in "cold," myeloid cell-dominant tumors. In contrast, Zheng et al. (Neuro-Oncology 2024, PMID: 37941134) reported that TREM2, expressed predominantly in microglia and to a lesser extent in macrophage subsets in the GL261 model, supports MHCII-dependent phagocytosis of tumor debris and promotes CD4$^+$ T-cell responses against glioma, with TREM2 deficiency reducing CD4$^+$ T cell infiltration and accelerating tumor growth. Similarly, Peshoff et al. (Neuro-Oncology 2024, PMID:

38237157), using both CT2A and GL261 models with human glioma cytometry data, showed that TREM2, expressed across microglia, macrophages, and monocyte-derived macrophages, correlates with phagocytosis markers (LYZ, CD163) rather than immunosuppressive genes (Arg1, Mrc1), and that $TREM2^+$ myeloid cells display enhanced phagocytic uptake of tumor cells via a Syk-dependent mechanism.

We believe these apparent discrepancies are likely to reflect differences in tumor immunogenicity (e.g., GL261 *versus* SB28) and the ontogenic origin of the dominant myeloid cell population, specifically, whether microglia (brain-resident, yolk sac-derived) or monocyte-derived macrophages (bone marrow-derived) predominate in the TME. These two populations have distinct transcriptional programs and respond differently to TREM2 perturbation. In GL261 and CT2A, which are more immunogenic and microglia-rich, TREM2 in microglia appears to support MHCII-dependent $CD4^+$ responses and phagocytosis. In SB28, a myeloid cell-dominant, poorly immunogenic model with greater monocyte-derived TAM infiltration (Simonds et al., JITC, 2021, PMID 34083417), $TREM2^+$ cells adopt a predominantly immunosuppressive, lipid-associated program that dampens IFNγ responses. Most prior studies relied on germline or conditional Trem2 deletion, which alters the myeloid cellular landscape throughout tumor development, for example reducing the number and activation state of homeostatic microglia, rather than acutely targeting established $TREM2^+$ populations. In contrast, our approach uses local CAR T cell-mediated targeting in a defined perioperative window, modeling a clinically translatable strategy aimed at depleting TREM2-expressing immunoregulatory niches at the time of surgery. This does not discriminate between microglia and monocyte-derived TAMs but directly addresses the immunosuppressive $TREM2^+$ myeloid cell niche that expands post-resection in our SB28 setting.

Because the primary objective of our study was to evaluate CAR T cell-based perioperative immunomodulation rather than genetically dissect TREM2 function in specific myeloid cell compartments, a cell type-restricted targeting strategy falls beyond the scope of the present work. Nonetheless, we agree this represents an important direction for future investigation and have expanded the Discussion accordingly (**Lines 491-504**) as follows:

"*Notably, recent studies have reported context-dependent roles of TREM2 in glioma, ranging from immunosuppressive functions in myeloid cell-dominant SB28 tumors[33] to MHCII-associated support of $CD4^+$ T cell responses in the GL261 and CT2A models[34], likely reflecting differences in tumor immunogenicity and myeloid cell ontogeny. An important methodological distinction is that prior studies relied on germline or conditional Trem2 deletion, which alters myeloid cell ecology throughout tumor development, whereas the CAR-T cell-based approach used here acutely targets established $TREM2^+$ populations within a defined perioperative window. Moreover, our data reveal that timing is a decisive variable: TREM2 targeting in the adjuvant setting enhanced GD2 CAR-T cell activity, whereas neoadjuvant TREM2 depletion unexpectedly attenuated it, consistent with a temporally dynamic model in which $TREM2^+$ cells may shift from antigen-presenting to predominantly immunosuppressive states following surgical injury. Compartment-specific targeting of $TREM2^+$ populations thus represents an important direction for future investigation.*"

Although several immunotherapy strategies were explored in murine glioma models, the manuscript could benefit from validation in a glioma stem cell–based model that more closely reflects human GBM pathology. Replicating key findings in neoadjuvant and adjuvant settings using TREM2-CAR, TREM2-CAR combined with GD2 CAR-T, and combinations with immune checkpoint blockade would strengthen the translational relevance.

We thank the reviewer for this suggestion. We fully appreciate the value of GSC-based syngeneic models, which more faithfully recapitulate human GBM features such as invasive growth patterns and therapy resistance. We have carefully considered this point and provide the following reasoning.

First, we wish to emphasize that SB28 and CT2A were specifically selected for their complementary and clinically relevant properties. SB28 was generated by sleeping beauty transposon-mediated transduction of oncogenic drivers (NRas, shp53, PDGF) and is well-established as one of the most immunologically cold syngeneic GBM models available, closely mimicking the resistance of human GBM to checkpoint immunotherapy. CT2A provides a second, independently derived model with a distinct TME composition, and the consistency of our key findings across both models strengthens the generalizability of our conclusions.

Second, a critical component of our translational validation strategy relies on the human patient-derived organotypic slice model, in which key findings, i.e. TREM2$^+$ myeloid cell expansion and T cell exhaustion-like phenotypes following cavity creation, were recapitulated in primary human GBM tissue (n=3 independent patients). This *ex vivo* human validation, together with our cross-species scRNAseq analysis confirming conserved TREM2 expression patterns across myeloid cell subsets in murine and human GBM (**Supplementary Fig. 2a-c**), provides direct evidence of translational relevance that complements and extends the murine *in vivo* data.

We acknowledge that validation in a GSC-based syngeneic model such as the 005 line would further strengthen the translational impact of this work. However, this would require stable engineering of GD2 expression, establishment and optimization of a surgical resection protocol in this model, and repetition of the full experimental pipeline including survival studies, immune profiling, CAR T cell therapy, and checkpoint blockade combinations, representing a scope that extends well beyond the current revision. We have added this as an explicit limitation and future direction in the revised Discussion (**Lines 578-581**): "*Future studies should validate these findings in GSC-based syngeneic models that more closely recapitulate the invasive growth and stem cell features of human GBM, and in patient-derived xenograft models where feasible.*"

Discussion: The discussion would benefit from additional context in several areas:

a) comparison of TREM2$^+$ myeloid populations observed in this murine study with published human datasets;

b) key differences between murine glioma models and human GBM immune microenvironments;

c) implications of targeting TREM2$^+$ cells for phagocytosis and antigen presentation, which are central functions associated with TREM2 signaling.

Discussion has been updated as follows:

TREM2 expression across myeloid cell subsets: murine *versus* human patterns.

In response to points a) and b), the following paragraph has been added on **lines 452-467**. "*These findings in murine models are broadly consistent with human GBM single-cell transcriptomic datasets, which have identified a TREM2^{high} macrophage state enriched in TAM, characterized by lipid metabolism gene signatures, immunosuppressive signaling, and spatial association with hypoxic tumor regions[8,49]. The cross-species scRNAseq analysis presented here confirmed that TREM2 transcripts are broadly distributed across microglia, macrophages, monocytes, and DC in both murine and human GBM, supporting conservation of TREM2 expression across myeloid cell lineages. Nevertheless, important differences between species warrant consideration. At the protein level, flow cytometry-based quantification suggests that TREM2 upregulation post-resection is most pronounced in infiltrating monocyte-derived populations in SB28 and CT2A, likely reflecting the greater contribution of blood-borne myeloid cells in rapidly growing syngeneic tumors. Additionally, human GBM harbors a more complex and heterogeneous myeloid cell landscape, including border-associated macrophages and ontogenically distinct TAM subpopulations[50], which may not be fully recapitulated in transplantable murine models. These caveats should be borne in mind when extrapolating our findings to the clinical setting.*"

Implications of targeting TREM2⁺ cells for phagocytosis and antigen presentation

This paragraph (**Lines 561-571**) "*In this regard, eliminating TREM2⁺ cells might deprive the host of antigen-presenting and phagocytic functions that are central to TREM2 biology. TREM2 signaling promotes lipid clearance, efferocytosis, and phagocytosis of tumor debris via Syk-dependent pathways, supporting sustained antigen uptake and MHCII-restricted presentation*[74,75]*. Consistent with this, recent work in GBM showed that TREM2⁺ TAM support MHCII-dependent CD4⁺ T cell responses, and that their depletion can impair rather than enhance antitumor immunity in immunogenic contexts*[32]*. These observations, together with the context-dependence of TREM2 function discussed above, underscore that therapeutic targeting of TREM2⁺ myeloid cells should be carefully timed and combined to avoid dismantling antigen-presenting niches that support endogenous and adoptive T cell responses.*"

The following reference has been added to the reference list:

75 Jaitin, D. A. *et al.* Lipid-Associated Macrophages Control Metabolic Homeostasis in a Trem2-Dependent Manner. *Cell* **178**, 686-698.e614 (2019). https://doi.org/10.1016/j.cell.2019.05.054

Minor Comments

Line 86–89: The combined statement of hypothesis and rationale is somewhat difficult to follow and could benefit from clearer phrasing.

We thank the reviewer for this comment. We have rephrased the hypothesis and rationale statement in the Introduction for clarity (**Lines 82-88**). The original text has been revised to:

" *Here, we first characterize the immunological consequences of surgical resection in two clinically relevant murine GBM models (SB28 and CT2A) and in patient-derived organotypic tissue, revealing a surge of TREM2⁺ myeloid cells and early upregulation of PD1/Tim3/PD-L1 as dominant features of the post-resection TME. Building on these observations, we tested multiple strategies to reshape the perioperative GBM TME, including TREM2 CAR-T cells alone, in combination with GD2-targeting CAR-T cells, and with anti-PD1/Tim3 or anti-PD-L1 checkpoint inhibitors.*"

Supplementary Figure 3: Please include labels identifying SB28 and CT2A datasets in the bar graphs.

We thank the reviewer for this comment. Labels identifying SB28 and CT2A datasets have been added in the revised **New Supplementary Fig. 4**.

Throughout the manuscript, please clearly indicate the number of independent biological repeats or whether datasets represent pooled data.

We thank the reviewer for this comment. We have systematically reviewed all figures and figure legends throughout the revised manuscript to explicitly state the number of independent biological replicates for each experiment, and to indicate whether the data shown represent pooled or single-experiment datasets. These clarifications have been added to all main figure legends (**Figures 1 to 7**) and supplementary figure legends (**Supplementary Figures 1 to 24**). Specific details for key experiments are provided in the relevant point-by-point responses above.

Provide gating strategies distinguishing TREM2⁺ and TREM2⁻ populations where applicable and reference them in the main text.

As detailed in our response to point 2 above, a new panel (**d**) has been added to **Supplementary Fig. 1**, displaying TREM2-stained samples (top row) and FMO controls (bottom row) for each myeloid cell subset (microglia, macrophages, monocytes, neutrophils and dendritic cells). Gates defining TREM2⁺ populations were set based on FMO controls. A reference to this panel has been added to the Results section and to the Methods.

Supplementary Figure 20: For MHCII and CD206 staining, please include FMO controls, particularly given the continuous distribution of CD206 expression.

All FMO controls, including FMO controls for MHCII and CD206 staining have been added to revised **Supplementary Fig. 22**.

Reviewer #1 (Remarks on code availability):

The authors has provided the required README , R script and example data files. However, running the code on example data is giving errors. The authors are suggested to make following changes to the code:

1. On line 17 of R script, change the path of ‘img_dir’ to “Example data/“ folder provided. At differs instances, different paths are given in the script and git hub repository.

We thank the reviewer for the careful testing of our code and for the constructive feedback. We have addressed all three points as follows:

Point 1 - Path of img_dir The hardcoded absolute Windows path on line 17 has been replaced with a portable relative path pointing directly to the Example data/ folder provided in the repository:

img_dir <- "Example data"

This ensures the script runs out-of-the-box regardless of the user's local directory structure, consistent with the paths indicated in the README and the GitHub repository.

2. Debug the R script between lines 225 - 229, as the command is unable to parse the Example data/ input file names and generates empty ‘dtp’ variable (0 rows and 6 columns)

The regular expression used to parse input filenames was too strict and failed to match filenames from the Example data folder. It has been rewritten to be more robust:

- Case-insensitive matching for the series keyword
- Flexible whitespace handling around delimiters
- Support for both standard hyphen (-) and en-dash (–)
- Optional = sign in the channel token (C=0 or C0)
- Added support for .tif and .tiff extensions (common microscopy formats)
- A diagnostic error message is now displayed if no files are matched, showing a sample of the filenames found to facilitate troubleshooting.

3. In Usage section, provide names and description of all 14 output files generated.

The README has been updated to include a complete description of all 14 output files generated by the script, including their filename pattern, format, and content.

We hope these corrections fully address the reviewer's concerns and improve the usability and reproducibility of the code.

Reviewer #2 (Remarks to the Author):

Pedard et al., present a preclinical evaluation of the GD2 CAR T cell efficacy with use of in vivo syngeneic mouse models and patient tumor tissue slice cultures. The authors confirm that surgical resection may lead to increased immunosuppression in the remaining tumor tissue. They suggest that neoadjuvant CAR T cell administration may provide better benefit than an adjuvant setting.

Several points need to be addressed:

1. The overall translational claim of the work at the end of each result section and in the discussion should be revised. While the work shows that TREM2 CAR T cells deplete TREM2$^+$ myeloid cells and recruit neutrophils in vivo, the anti-tumor activity of GD2-CART cells is minimal, based on minimal impact on the survival (Fig 4). Combined TREM2-CAR T cell + GD2 CAR T cell treatment appears to show minimal responses when combined with the surgery, with variable statistical outcomes (Fig. 6 significant, Fig 5 non-significant). Actually, anti-PD1/anti-TIM3 alone shows a higher impact on the survival in the assessed model (Fig 5), though non-curative as well.

We agree with the reviewer that survival data shown in Fig. 4, 5 and 6 show limited increase in survival with the different treatments. However, combination of TREM2 CAR T cells and GD2 CAR T cells as shown in Fig. 5 (UTD + UTD + isotype vs. TREM2 CAR + GD2 CAR + isotype, black vs. light pink lines) and Fig. 6 (UTD + UTD + isotype vs. TREM2 CAR + GD2 CAR + isotype, black vs. light pink lines) are in both instances significant: Fig. 5: $p=0.004$, increase of survival: 10 days; Fig. 6: $p<0.0001$, increase of survival: 6 days.

However, we understand the reviewer's comment as, in Fig. 5, groups were indeed mislabeled in the original submission and the UTD + UTD + anti-PD1/anti-Tim3 and TREM2 CAR + GD2 CAR + isotype groups were inverted. We apologize for this error. The underlying raw data (provided in the Raw Data Excel file, sheet "Fig. 5b") was correct, and the statistical comparisons were performed in the correct groups. The figure has been corrected in the revised manuscript.

Regarding the fact that our treatments cannot cure mice: SB28 is known to be resistant to immune checkpoint inhibitors (Genoud et al., Oncoimmunology 2021) and poorly permissive to CAR T cell therapy (Hatae et al., JCI Insight 2024, Murty et al., Oncoimmunology 2020), consistent with the fact that it closely recapitulates human GBM. We fully acknowledge that limitation and have rephrased the translational claims in the Results and the Discussion.

(**i**) The Results section on the combination of TREM2 and GD2 CAR T cells (**lines 275-277**) now reads: *"Together, these findings suggest that targeting TREM2$^+$ myeloid cells via TREM2 CAR-T cells is associated with improved functional state of the TME and enhanced proliferation, persistence, and differentiation of co-administered GBM-directed CAR-T cells.".*

(**ii**) have also added to the discussion (**lines 572-578**): "*While the survival benefits observed with our combinatorial strategies remain modest in absolute terms, this is consistent with the well-established resistance of the SB28 model to both immune checkpoint blockade and CAR-T cell therapy[21,31,76], features that closely recapitulate the immunosuppressive landscape of human GBM. These results should therefore be interpreted as proof-of-concept evidence that perioperative myeloid remodeling combined with tumor-targeting CAR-T cells can reshape the post-resection TME and extend survival in a highly stringent preclinical setting, rather than as evidence of curative efficacy.*"

The following reference has been added to the reference list:

76 Murty, S. *et al*. Intravital imaging reveals synergistic effect of CAR T-cells and radiation therapy in a preclinical immunocompetent glioblastoma model. *Oncoimmunology* **9**, 1757360 (2020). https://doi.org/10.1080/2162402x.2020.1757360

Later, the authors show that GD2 CAR T cells work better in the neoadjuvant application and TREM2 CAR T cells do not add the benefit. Interestingly, this regimen showed minimal advantage for approx. 70% of the neoadjuvant treated mice, and remaining 30% appeared to be cured (Fig 7). While the authors analyse in depth the immune compartment, no information is provided about the overall tumor structure and the impact on the follow-up surgery in the treatment experiments presented. E.g., do GD2 CAR T cells in the adjuvant setting attack the tumor core and borders? Is tumor structure or invasion capacity affected, allowing for more extensive surgical intervention in 30% of mice? Or do authors expect the systemic or local anti-tumor immunisation? Since the surgery is likely to remove the majority of the intratumoral myeloid cells and injected GD2 CAR T cells, the potential mechanism needs to be assessed in further detail.

We thank the reviewer for this important comment. We fully agree that characterizing tumor structure and CAR T cell localization is essential to understand the mechanisms underlying the differential efficacy of neoadjuvant versus adjuvant delivery. We have performed immunofluorescence analyses that directly address each of the reviewer's questions, now presented as new panels incorporated into **Fig. 7** (**new panels m and n**).

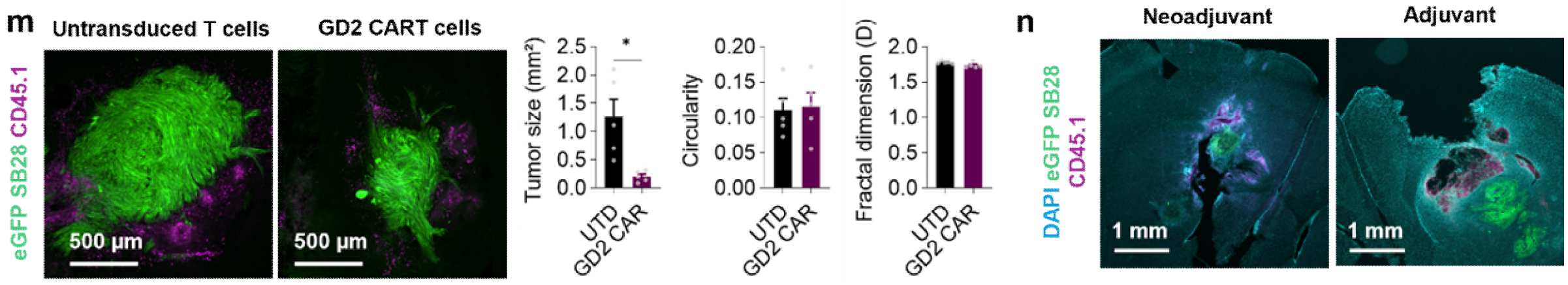

Fig. 7 (***m***) *Representative immunofluorescence images (eGFP SB28, green; CD45.1, magenta; scale bar=500 µm) and quantification of tumor size (mm²), circularity, and fractal dimension in SB28 tumor-bearing mice treated with UTD or GD2 CAR T cells at day 7 post-implantation and sacrificed at day 10 from one experiment (n=5 mice per group, unpaired two-tailed t-test).* (***n***) *Representative immunofluorescence images (DAPI, cyan; eGFP SB28, green; CD45.1, magenta; scale bar=1 mm) showing GD2 CAR T cell distribution relative to the resection cavity at 24 h post-resection in neoadjuvant- versus adjuvant-treated mice (one representative staining from n=5 mice per group).*

(i) Do GD2 CAR T cells affect tumor structure prior to surgery? To address this, we sacrificed mice on day 10 post-implantation, corresponding to the time of surgical resection, following injection of UTD or GD2 CD45.1$^+$ CAR T cells on day 7. Immunofluorescence analysis of GFP$^+$ tumor area and morphology showed a significant reduction in tumor size in GD2 CAR T cell-treated mice compared to UTD controls (**Fig. 7, new panel m**). However, neither tumor circularity nor fractal dimension, two parameters reflecting tumor shape and invasive border complexity, differed significantly between groups, suggesting that neoadjuvant GD2 CAR T cells reduce tumor bulk without affecting invasion capacity. These data indicate that the survival benefit observed in the neoadjuvant setting is not explained by a reduction in tumor invasiveness prior to surgery. CD45.1$^+$ GD2 CAR T cells were detected at the tumor borders in neoadjuvant-treated animals, consistent with active engagement of residual tumor tissue.

(ii) Do neoadjuvant versus adjuvant CAR T cells occupy different niches post-resection? To address whether the timing of delivery affects CAR T cell localization relative to the resection cavity, we performed immunofluorescence 24 h post-resection in both settings (**Fig. 7, new panel n**). In neoadjuvant-treated mice, CD45.1$^+$ GD2 CAR T cells were distributed around the resection cavity and along the residual tumor margins. In contrast, in adjuvant-treated mice, CAR T cells appeared predominantly confined within the resection cavity itself, with limited engagement of the surrounding tumor parenchyma. These findings provide a mechanistic basis for the superior efficacy of neoadjuvant delivery: CAR T cells administered prior to surgery are pre-positioned at the tumor-parenchyma interface, where they can engage residual invasive tumor cells following resection, whereas adjuvant CAR T cells are delivered into a post-surgical cavity that physically limits their access to residual tumor. We have incorporated this interpretation into the revised Results as follows (**Lines 351-364**):

"*To investigate the impact of neoadjuvant GD2 CAR-T cell treatment on tumor structure at the time of resection, we performed immunofluorescence analysis of GFP⁺ tumor area and morphology at day 10 post-implantation. Neoadjuvant GD2 CAR-T cell treatment significantly reduced tumor size compared to UTD controls, while tumor circularity and fractal dimension remained unchanged, indicating a reduction in tumor bulk without alteration of invasion capacity (**Fig. 7m**). CD45.1⁺ GD2 CAR-T cells were detected at tumor borders, consistent with active engagement of residual tumor tissue prior to surgery. Furthermore, immunofluorescence performed 24 h post-resection revealed that neoadjuvant CAR-T cells were distributed around the resection cavity and along residual tumor margins, whereas adjuvant CAR-T cells appeared predominantly confined within the resection cavity, with limited engagement of the surrounding tumor parenchyma (**Fig. 7n**). These data provide a mechanistic basis for the superior efficacy of neoadjuvant delivery, in which CAR-T cells are pre-positioned at the tumor-parenchyma interface prior to surgery.*"

We have added this paragraph to "immunohistofluorescence" section in Methods (**Lines 742-747**) to explain tumor invasiveness assessment as follows "*To assess tumor invasiveness, fractal dimension analysis was performed on GFP⁺ tumor using the box-counting method implemented in FIJI, as previously described*[79]*. Briefly, binary masks of GFP⁺ tumor regions were generated by thresholding, and the fractal dimension (D value) of the tumor was calculated to quantify morphological complexity as a proxy for invasive capacity. Tumor circularity was additionally computed from the same binary masks using the standard FIJI shape descriptor (circularity = 4π × area/perimeter²).*"

We have revised legend from Fig.7 as follows (**Lines 1278-1285**):

"*(**m**) Representative immunofluorescence images (GFP⁺ SB28, green; CD45.1, magenta; scale bar=500 µm) and quantification of tumor size (mm²), circularity, and fractal dimension in SB28 tumor-bearing mice treated with UTD or GD2 CAR-T cells at day 7 post-implantation and sacrificed at day 10 from one experiment (n=5 mice per group, unpaired two-tailed t-test). (**n**) Representative immunofluorescence images (DAPI, cyan; GFP⁺ SB28, green; CD45.1, magenta; scale bar=1 mm) showing GD2 CAR-T cell distribution relative to the resection cavity at 24 h post-resection in neoadjuvant- versus adjuvant-treated mice (one representative staining from n=5 mice per group).*"

The following reference has been added to the reference list:

79 Yadav, V. N. *et al.* CXCR4 increases in-vivo glioma perivascular invasion, and reduces radiation induced apoptosis: A genetic knockdown study. *Oncotarget* **7**, 83701-83719 (2016). https://doi.org/10.18632/oncotarget.13295

2. Selection of GD2 as the target is questionable. The mouse models were modified to express GD2 artificially, while no information is available for the applied patient tumor tissue samples. The GD2 expression in patient samples should be presented and discussed.

We thank the reviewer for raising this important point. We address the question of GD2 expression in human GBM and the rationale for its selection in two parts.

(i) GD2 expression in human GBM, literature evidence.

GD2 is a well-established tumor-associated antigen in GBM supported by a robust and growing body of evidence. Prapa et al. isolated patient-derived GBM cells from primary biopsies and demonstrated high GD2 surface expression by flow cytometry in the majority of samples, as well as extensive GD2 staining in GBM resection specimens by immunohistochemistry and immunofluorescence (Prapa et al., npj Precis Oncol 2021, PMID: 34707200). Gargett et al. confirmed high-level GD2 expression on surgically resected human GBM tissue and showed, using public TCGA datasets, that the two-gene signature of GD2 and GD3 synthases (B4GALNT1/ST8SIA1) is highest in GBM among all cancer types analyzed (Gargett et al., J Immunother Cancer 2022, PMID: 36167468). Liu et al. reported a clinical trial of GD2-specific CAR T cells in eight GD2-positive GBM patients, with four achieving partial responses lasting

3 to 24 months, confirming both the prevalence of GD2 expression and the clinical translatability of this approach (Liu et al., Mol Cancer 2023, PMID: 36617554). Taken together, these studies establish that GD2 is broadly and consistently expressed in human GBM and support its rational selection as a CAR T cell target in this disease.

(ii) GD2 expression in patient-derived GBM cells, *new data*.

To directly address the reviewer's concern, we performed flow cytometry analysis of GD2 surface expression on a patient GBM sample obtained after tumor resection at our institution (HUG, Geneva, Switzerland). After tumor dissociation, GD2 expression was assessed by flow cytometry on live, $CD45^{-}$ cells to exclude infiltrating immune cells. As shown in **Fig. R4** (shown to the reviewer only), robust GD2 surface expression was confirmed on patient-derived tumor cells, consistent with the published literature cited above. These data confirm that the GD2 target is relevant not only to the engineered syngeneic murine models used in this study but also to patient-derived GBM samples.

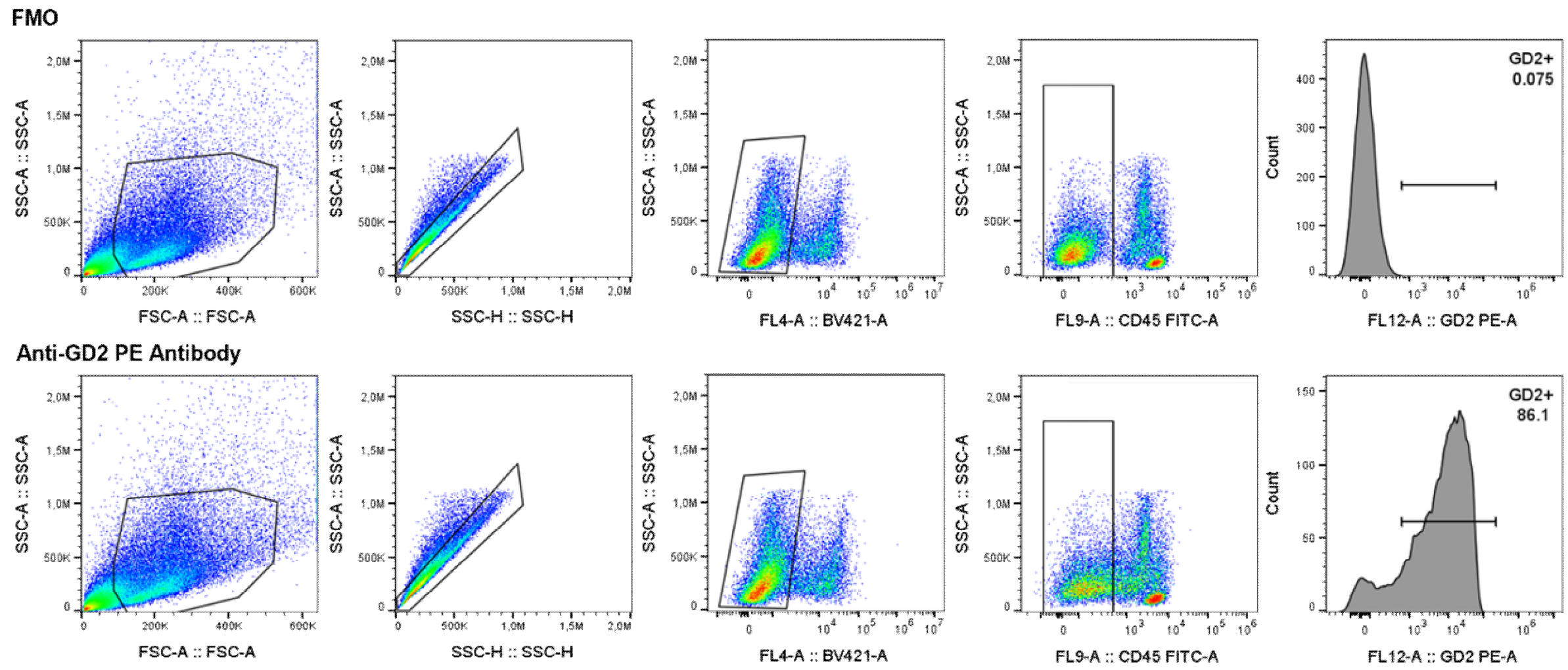

Figure R4: GD2 surface expression on patient-derived GBM tumor cells. *Representative flow cytometry histograms showing GD2 surface expression on dissociated patient-derived GBM tumor cells. Top row: FMO control. Bottom row: anti-GD2 staining. Data demonstrate robust GD2 surface expression on primary human GBM cells, supporting the translational relevance of GD2-targeting strategies.*

(iii) On the use of engineered GD2-expressing murine models.

We acknowledge that the SB28 and CT2A cell lines were engineered to stably express GD2 via lentiviral transduction of GD2 and GD3 synthases, as these syngeneic murine lines do not naturally express this human ganglioside. This approach is a standard and necessary strategy in the preclinical CAR T cell field, where target antigen engineering is required to study efficacy in fully immunocompetent hosts, a limitation shared by virtually all published syngeneic GBM CAR T studies (Murty et al., Oncoimmunology 2020, PMID: 32923113; Hatae et al., JCI Insight 2024, PMID: 38386420). The critical advantage is that this system enables rigorous mechanistic dissection of CAR T cell biology and TME interactions in an immunocompetent setting, which would not be possible in xenograft models.

GD2 expression in engineered SB28 and CT2A cell lines was validated by flow cytometry prior to all *in vivo* experiments. Representative histograms are now provided as a new Supplementary Figure (**Supplementary Fig. 21**), and the corresponding description has been added to the Methods section under "Cell lines" (**Line 589**).

Flow cytometry analysis showed that CT2A-GD2 cells exhibited lower GD2 expression than SB28-GD2 cells (**Supplementary Fig. 21**). GD2 expression was assessed using an anti-GD2 antibody with appropriate isotype controls.

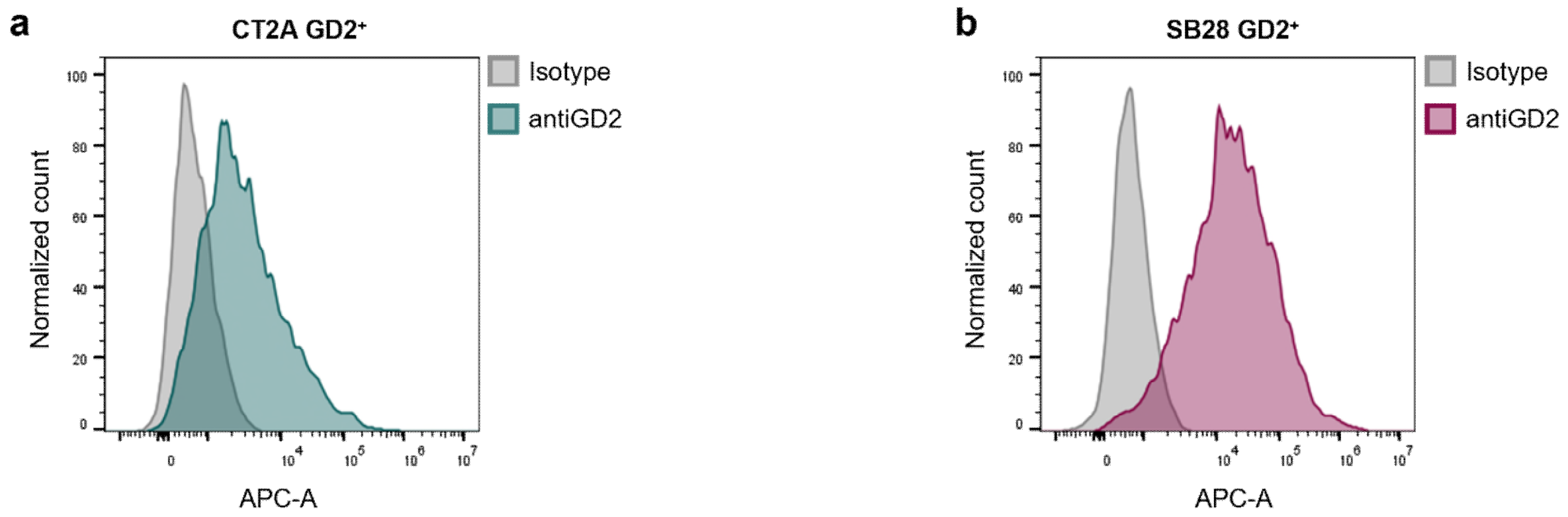

Supplementary figure 21. *GD2 surface expression on murine GBM cell lines. Representative flow cytometry histograms showing GD2 surface expression on CT2A GD2⁺ (**a**) and SB28 GD2⁺ (**b**) cell lines cultured in vitro. Cells were stained with an anti-GD2 antibody (colored) or isotype control (grey). The percentage of GD2⁺ cells within the live CD45⁻ gate is indicated.*

3. The extent of the surgical resection in vivo and ex vivo should be further evaluated and discussed:

- It is unclear if the TREM+ myeloid cells are increased due to activation of the marker expression of pre-existing cells, or influx of the 'fresh' monocytes or microglia to the tissue surrounding the resection cavity.

- Lines 143- 148 and 171-183 - the ex vivo platform was used for validating in vivo findings. Importantly, the authors should comment on the lack of blood flow in patient tumor brain slices, since this limits the influx of the monocytes and T cells from the blood. It is unclear if the changes in the myeloid cells compartment are linked to the influx of normal brain-residing microglia or 'left-over' TAMs.

- Where do the T cells (particularly T naïve cells) come from 24h post-resection? Tumor borders typically left after the surgery are known to contain few T cells. Do the authors expect the influx from the circulation or reactivation of left-over TILs? Since human brain tissue slices showed relevant changes, the latter could be hypothesized.

We thank the reviewer for these important mechanistic questions, which we address one by one.

(i) On the origin of TREM2⁺ myeloid cells after resection: local induction *versus* cell influx.

Distinguishing between local TREM2 upregulation in pre-existing cells and influx of TREM2-expressing monocytes from the circulation is methodologically challenging in standard *in vivo* models. Our human organotypic slice data, however, provides a strong argument in favor of local induction. The organotypic model is entirely avascular, no blood flow, no circulating monocytes can enter the slice, meaning that the significant increase in TREM2⁺/CD68⁺ cells observed after *ex vivo* cavity creation cannot reflect peripheral recruitment and must arise from local reprogramming: transcriptional upregulation of TREM2 in resident microglia and/or pre-existing TAMs in response to injury-associated signals (DAMPs, hypoxia, TGFβ), and/or spatial redistribution of TREM2-expressing cells toward the injury margin. This interpretation is further supported by our spatial data in SB28 tumors *in vivo*, where TREM2⁺ cells were repositioned toward the resection cavity as early as D1, consistent with local migration rather than *de novo* recruitment. We acknowledge that *in vivo*, both local reprogramming and peripheral monocyte influx, the latter being known to upregulate TREM2 upon TAM differentiation, likely co-exist, and that the organotypic model does not fully recapitulate this vascular component. This nuance is explicitly discussed in the revised manuscript.

(ii) On the origin of T cells, particularly naïve T cells, 24 h post-resection.

This is an important question. In the *in vivo* murine setting, circulating T cells can in principle access the tumor via the remaining vasculature, which may explain some of the early lymphoid changes observed at 24 h. However, several observations argue against peripheral recruitment as the primary driver. First, the 24 h timepoint is very early for significant lymphocyte egress and homing in response to surgical injury. Second, and most critically, our human organotypic slice model, entirely avascular, recapitulates the same T cell exhaustion changes as those seen *in vivo* 72 h post-resection (**Fig. 2e-h**), with selective exhaustion of effector and effector memory subsets and relative sparing of T_{naive} and T_{cm} cells. Since no circulating T cells can enter the slice, these changes must arise from modulation of pre-existing TILs retained within the tissue. The emergence of T_{naive}-enriched phenotypes likely reflects phenotypic dedifferentiation or selective retention of less-differentiated TIL subsets under local injury signals rather than true influx of naive T cells from the circulation. We have now discussed this interpretation explicitly in the revised manuscript, while acknowledging that *in vivo*, a contribution from circulating T cells cannot be formally excluded, particularly at later timepoints.

We have addressed each point in the revised Discussion section as follows:

Regarding the origin of TREM2⁺ myeloid cells: local induction *versus* cellular influx (Discussion, lines 437-449): We have added the following passage:

" TREM2 is a damage-sensing receptor that is upregulated in myeloid cells in response to diverse tissue injury signals, including lipid-associated damage-associated molecular patterns (DAMP), hypoxia, and extracellular matrix remodeling[47], all of which are hallmarks of the post-surgical brain microenvironment. The observation that this $TREM2^+$ myeloid cell expansion occurs in the absence of any vascular perfusion in our human organotypic model strongly suggests that local transcriptional reprogramming of tissue-resident populations, rather than peripheral monocyte recruitment, is the primary initiating mechanism. In vivo, blood-borne monocytes are known to infiltrate GBM and contribute to the TREM2-expressing myeloid cell compartment alongside microglia[33,48], and likely further amplify this locally initiated response. We acknowledge that the organotypic model does not allow discrimination between microglia and monocyte-derived macrophages at the ontogenic level and does not fully recapitulate the vascular component of the post-resection niche in vivo, representing an important limitation when extrapolating these findings to the clinical setting."

Regarding the origin of T cells, particularly naïve T cell, after resection (Discussion, lines 533-540): We have added the following passage:

" In our model, the early emergence of exhaustion-like phenotypes in Teff cell subsets is most consistent with local reprogramming of pre-existing tumor-infiltrating lymphocytes rather than de novo recruitment from the circulation. The relative enrichment of less-differentiated T cell subsets likely reflects phenotypic dedifferentiation rather than true influx of naive T cells. In vivo, a contribution from circulating T cells cannot be formally excluded, particularly at later timepoints, but the convergence between our murine and human ex vivo data strongly implicates local reprogramming as the dominant mechanism."

The following references have been added to the reference list:

41 Woroniecka, K. *et al.* T-Cell Exhaustion Signatures Vary with Tumor Type and Are Severe in Glioblastoma. *Clinical cancer research : an official journal of the American Association for Cancer Research* **24**, 4175-4186 (2018). https://doi.org/10.1158/1078-0432.Ccr-17-1846

47 Deczkowska, A., Weiner, A. & Amit, I. The Physiology, Pathology, and Potential Therapeutic Applications of the TREM2 Signaling Pathway. Cell 181, 1207-1217 (2020). https://doi.org/https://doi.org/10.1016/j.cell.2020.05.003

48 Bowman, R. L. *et al.* Macrophage Ontogeny Underlies Differences in Tumor-Specific Education in Brain Malignancies. *Cell reports* **17**, 2445-2459 (2016). https://doi.org/10.1016/j.celrep.2016.10.052

- The in vivo tumors are analyzed 7 days after the resection in the majority of experiments. It is unclear whether the tumor growth had already recovered and the resection cavity was resolved at this stage. It should be analyzed how long the impact of the resection is retained. It is possible that the assessed differences result in the removal of the main tumor mass and assessment of the 'left-over' myeloid cell compartment in the more invasive tumor niche, thus the direct impact of the surgery should be assessed with caution, or the composition of the myeloid cells should be assessed in different tumor areas at the moment of the surgery.

We thank the reviewer for this important point, and we clarify the experimental timepoints below, as the analysis windows differ between our characterization and treatment experiments.

Timepoints used. The myeloid compartment characterization following resection (**Fig. 1c-d, Supplementary Fig. 2c-d, Supplementary Fig. 3**) was performed on day 20 post-implantation, i.e. 10 days post-resection. In contrast, all therapeutic experiments (TREM2 CAR T, GD2 CAR T, and combination treatments) analyzed tumors at day 13 post-implantation, i.e. 3 days post-resection. The immunofluorescence analyses (**Fig. 1g-h**) were performed on day 1 and day 7 post-resection to capture the spatial dynamics of the myeloid compartment during the early post-surgical window.

Tumor regrowth and cavity resolution at the time of analysis. Two lines of evidence directly address the reviewer's concern. First, longitudinal BLI quantification shows that tumor burden in resected mice is comparable to that of unresected controls within 3 days of resection in our models, confirming that tumor regrowth is rapidly re-established. Second, our immunofluorescence data at day 7 post-resection (**Fig. 1g**) show dense GFP^+ tumor cell infiltration within the former resection cavity, demonstrating that the cavity is repopulated by recurrent tumor tissue at this stage. Together, these data establish that at both the 3-day (treatment experiments) and 10-day (characterization experiment) post-resection timepoints, the resection cavity is no longer void but occupied by a recurrent tumor mass. The immune compartment we profile therefore reflects the early recurrence niche rather than a post-surgical wound-healing response in the absence of tumor.

Spatial composition caveat. We fully agree that the observed myeloid differences may in part reflect the altered spatial composition of the residual post-resection tumor, which is enriched for invasive cells at the tumor margin rather than the bulk tumor core.

We now explicitly acknowledge this in the revised Discussion (**Lines 430-436**) as follows: "*It should be noted that, because surgical cytoreduction removes the bulk tumor mass, the myeloid cell compartment profiled after resection is necessarily enriched for cells residing within the invasive tumor margin and the early recurrence niche rather than the original tumor core. The myeloid cell alterations described here may therefore partly reflect this shift in spatial sampling, in addition to genuine surgery-induced reprogramming, a distinction that motivated our spatial immunofluorescence characterization of the post-resection tumor bed.*"

- Neoadjuvant treatment requires intracranial injection to the developed tumor, which also may create a local wound healing response. However, since the main tumor mass has not been removed, the pool of myeloid cells/TILs and their responses may differ. These responses should be assessed correctly in order to claim the advancement of neoadjuvant treatment.

We thank the reviewer for this important methodological point. We fully agree that the intracranial injection required for neoadjuvant CAR T cell delivery could itself induce local wound-healing responses that confound the interpretation of immune changes attributed to CAR T cell activity. To directly address this concern, we have performed an additional experiment in which SB28 tumor-bearing mice were left untreated, injected intracranially with untransduced (UTD) T cells, or injected with GD2 CAR T cells at day 7 post-implantation, the same timepoint used for neoadjuvant delivery in the

therapeutic experiments. Brains were collected on day 10 post-implantation, corresponding to the time of surgical resection in the therapeutic cohorts, without performing resection (n=6-7 mice per group). Flow cytometry analysis of the myeloid cell compartment was performed in all three groups.

This three-arm design allows direct dissection of two distinct effects: (**i**) the impact of intracranial injection *per se* on the TME, assessed by comparing untreated versus UTD T cell-injected mice; and (**ii**) the GD2 CAR T cell-specific effect on the TME, assessed by comparing UTD versus GD2 CAR T cell-injected mice. These data are presented in **Fig. R5** (shown for the reviewer only) and confirm that the myeloid cell remodeling observed in GD2 CAR T cell-treated tumors after resection (**Fig.7h-i**) is not explained by the injection procedure alone, supporting the interpretation that neoadjuvant GD2 CAR T cells exert genuine immunomodulatory activity prior to surgery.

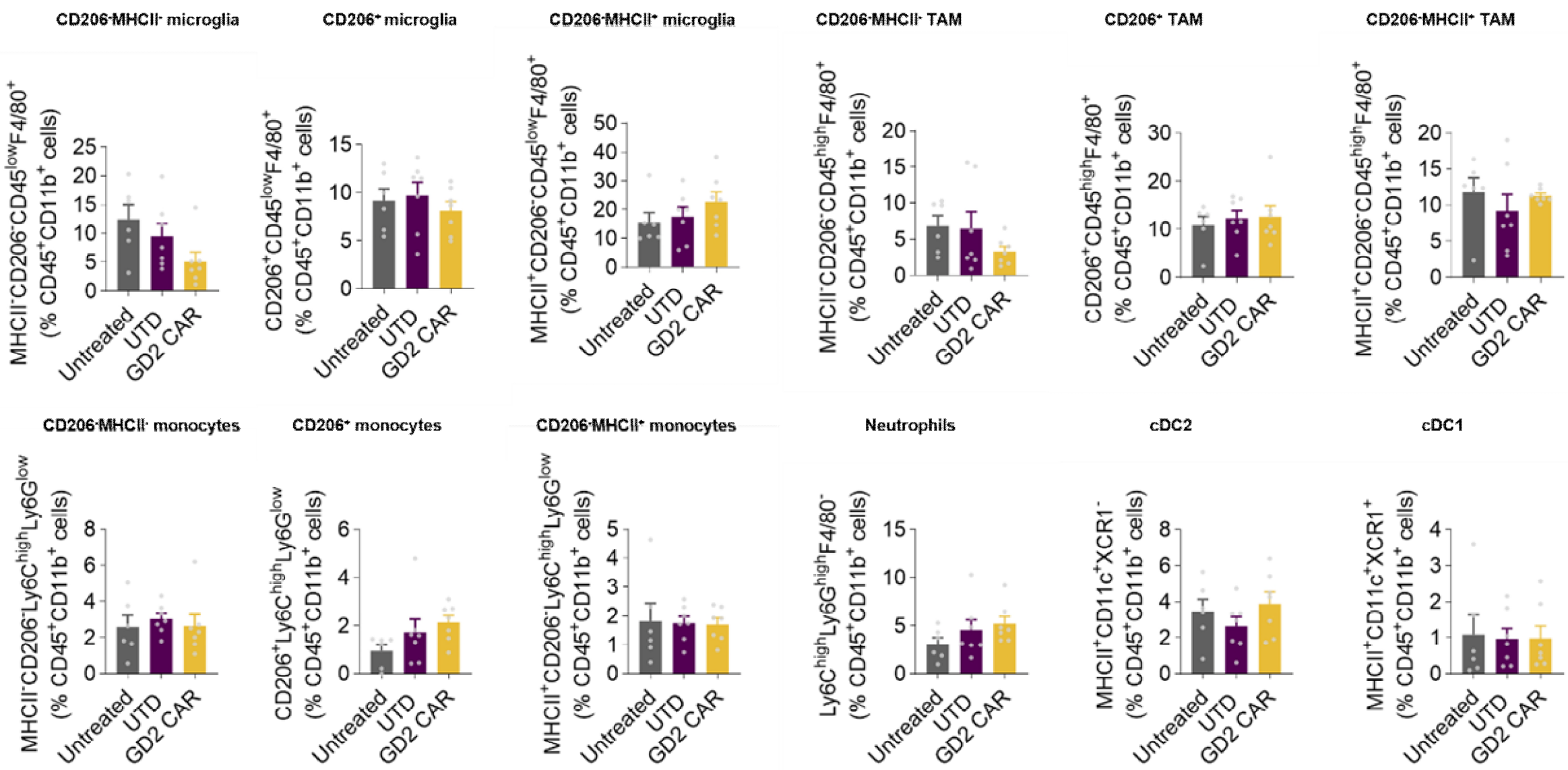

Figure R5: Three-arm myeloid characterization at D10 post-implantation: untreated vs. UTD vs. GD2 CAR T cells, without resection. *Flow cytometry quantification of myeloid cell subset frequencies in SB28 tumor-bearing mice left untreated, injected intracranially with untransduced (UTD) T cells, or injected with GD2 CAR T cells at D7 post-implantation and sacrificed at D10 without surgical resection (n=6-7 mice per group). Subsets are shown as percentage of* $CD45^{+}CD11b^{+}$ *cells and include* $CD45^{low}F4/80^{+}$ *microglia,* $CD45^{high}F4/80^{+}$ *TAMs,* $CD206^{-}MHCII^{-}$ *monocytes, neutrophils, and dendritic cells.*

4. The main difficulty in assessing the obtained responses is the myeloid subset discrimination strategy and nomenclature presented by the authors. The discrimination of the myeloid subsets is not based on the commonly applied discriminations, unclear and inconsistent across the manuscript. In general, many of the myeloid subsets are not well-defined in the gating strategy or another name is used, creating confusion. Moreover, flow graphs should display the fluorescence intensity recorded, to compare the expression levels across the gating strategies. E.g.:

- Lines 54-57 and definitions in 100-101: the authors should correct the description of the myeloid compartment and clarify the various origins of TAMs (microglia, monocytes, border-associated macrophages). In general, both microglia and blood monocytes (including MDSCs) contribute to the pool of so-called TAMs. Microglia, when in contact with GBM cells, upregulate CD45, thus becoming part of the 'macrophage' population in the gating presented.

- The nomenclature 'resting' TAMs, monocytes and MDSCs is unclear (lines 332). While microglia can be in a homeostatic 'resting' state, the terminology does not fit to other myeloid cells in this context.

- In general gating strategy and association of certain myeloid cells is unclear or not correct. E.g.: CD206+ cells were previously annotated as border-associated macrophages (e.g. doi: 10.1038/s41593-020-00789-y, 10.1038/s41467-021-21407-w, 10.1101/2023.03.05.531162). Here the authors divided them into microglia and macrophage subsets. 'CD206+ microglia/macrophages are also positive for

MHCII. Ly6G expression in MDSCs appears more as a background, compared to Ly6G positivity in neutrophils. Ly6ChighLy6Ghigh neutrophil term is applied in the text, while gating indicates granulocytes.

We thank the reviewer for these detailed and constructive comments on myeloid cell subset discrimination and nomenclature. We have addressed each point below and have implemented the corresponding changes throughout the revised manuscript, figure legends, and **Supplementary Figures 22-23**.

TAM origins and myeloid compartment description. We agree that the original description oversimplified the ontogenic diversity of GBM-associated myeloid cells. We have revised lines 54-57 to explicitly acknowledge that the myeloid cell compartment in GBM comprises brain-resident microglia, blood-borne monocyte-derived macrophages, and potentially border-associated macrophages (BAMs), all of which contribute to the broader pool of tumor-associated myeloid cells. We have added the relevant references (doi:10.1038/s41593-020-00789-y; doi:10.1038/s41467-021-21407-w). We have also clarified in the Methods that microglia is identified as $CD45^{low}$ $CD11b^{+}$ $F4/80^{+}$ cells, and that the $CD45^{high}$ macrophage gate encompasses monocyte-derived TAMs that have upregulated CD45 upon contact with GBM cells, as the reviewer correctly notes. This distinction is now explicit in both the text and in **Supplementary Table 3**.

In the Introduction (**lines 48-53**), the sentence: " *The GBM TME is dominated by a heterogeneous myeloid cell compartment comprising brain-resident microglia, monocytes, blood-borne monocyte-derived macrophages, and potentially border-associated macrophages, which can represent up to 40-50% of the tumor mass*[8,9]*. This myeloid cell infiltrate contributes to the suppression of T cell function through cytokine release, expression of inhibitory ligands, and metabolic dysregulation*[10]."

The following references have been added to the reference list:

8 Pombo Antunes, A. R. *et al.* Single-cell profiling of myeloid cells in glioblastoma across species and disease stage reveals macrophage competition and specialization. *Nature neuroscience* **24**, 595-610 (2021). https://doi.org/10.1038/s41593-020-00789-y

9 Ochocka, N. *et al.* Specialized functions and sexual dimorphism explain the functional diversity of the myeloid populations during glioma progression. *Cell reports* **42**, 111971 (2023). https://doi.org/10.1016/j.celrep.2022.111971

"Resting" nomenclature. We fully agree with the reviewer's point. The term "resting" has been removed throughout the manuscript. For microglia, monocytes and macrophages, the $CD206^{-}MHCII^{-}$ subsets are now referred to by their marker-based definition only, avoiding any functional inference. This change is reflected throughout the Results, main figures, figure legends, and Supplementary Figures.

$CD206^{+}$ populations and BAM annotation. We thank the reviewer for raising this important point. CD206 has indeed been used as a marker of border-associated macrophages in certain datasets (doi:10.1038/s41593-020-00789-y; doi:10.1038/s41467-021-21407-w; doi:10.1101/2023.03.05.531162). In our gating strategy, however, CD206 is used as a secondary marker within already-defined microglia ($CD45^{low}$) and macrophage ($CD45^{high}$) parent gates and is not used in isolation to define a BAM population. We acknowledge that $CD206^{+}$ cells within both compartments may include BAM-like phenotypes and have revised the text accordingly, noting that $CD206^{+}$ subsets within our gating may partially overlap with BAM populations. We also acknowledge, as the reviewer notes, that $CD206^{+}$ cells in our dataset co-express MHCII, which we now explicitly state in the revised Methods and legend of **Supplementary Fig. 22**.

MDSC removal and Ly6G nomenclature. We agree with the reviewer that the Ly6G signal in the MDSC gate appears at background levels compared to the clearly $Ly6G^{+}$ neutrophil population, making

this distinction unreliable. We have therefore removed the MDSC category from all figures, legends, and analyses throughout the revised manuscript. Concurrently, the neutrophil population is now consistently referred to as neutrophils ($CD45^{high}$ $CD11b^{+}$ $Ly6G^{+}$ $Ly6C^{+}$) throughout the manuscript, replacing the inconsistent "$Ly6C^{high}$ $Ly6G^{high}$" and "granulocyte" terminology. These changes are reflected in the text, all figure legends, and **Supplementary Table 3**.

Flow graphs and fluorescence intensity display. We have updated the representative flow cytometry panels in **Supplementary Fig. 22-23** to display fluorescence intensity on both axes, allowing direct comparison of marker expression levels across the gating hierarchy. FMO controls for MHCII and CD206 have been added as requested (see also response to minor comment on **Supplementary Fig. 22**).

Reviewer #3 (Remarks to the Author):

Using two immunocompetent orthotopic glioma mouse models and a human glioblastoma organotypic slice model, the authors demonstrate an early induction of TREM2+ myeloid cells and a late induction of exhausted lymphocytes following partial tumor aspiration. Subsequently, they generated TREM2-targeting CAR T cells and evaluated their anti-tumor efficacy in vitro and in vivo. While these cells showed minimal anti-tumor activity as a monotherapy, they had synergistic activity when combined with GD2 CAR T cells. Additional immune checkpoint inhibition did not further enhance survival and neoadjuvant therapy proved was more effective than adjuvant administration.

This is a comprehensive and timely study that provides valuable insights into the effects of dual myeloid/lymphoid targeting and the critical importance of therapeutic timing.

Major

Is the resection-mediated change tumor-specific, or would similar immunological shifts occur following a sham resection of a healthy, non-tumor-bearing brain?

We thank the reviewer for this important question. We fully agree that distinguishing tumor-specific immune changes from the non-specific effects of craniotomy and mechanical brain injury is an important consideration. However, performing a sham resection on healthy, tumor-free mice raises significant ethical concerns: aspirating brain parenchyma in a healthy animal would cause unjustifiable neurological damage without scientific gain that cannot be obtained by alternative means, and our institutional ethics committee does not approve such procedures when equivalent information can be drawn from existing literature. We therefore address this question through a careful review of published data from well-established sham-craniotomy and controlled cortical impact (CCI) models.

A substantial body of literature characterizes the immune response to sham craniotomy and mechanical brain injury in tumor-free animals and provides strong points of comparison with our tumor-bearing resection model. Following sham craniotomy or CCI, resident microglia undergo robust activation with morphological transition from ramified to amoeboid states, upregulation of CD45 and CD86, and transient elevation of pro-inflammatory cytokines (IL-1α, IL-1β, IL-6, TNF-α) that peak within 6–24 h and largely resolve by 72 h to 7 days (Mannix et al., J Neurotrauma 2011; Lopez-Rodriguez et al., 2015; Cole et al., PLoS One 2011). Importantly, these responses are predominantly localized to the ipsilateral cortex and do not reproduce the specific features we describe in our tumor resection model, notably, the sustained TREM2^{+} myeloid cell expansion at day 10 post-surgery, the spatial repositioning of TREM2^{+} cells toward residual tumor, the coordinated late lymphoid exhaustion with PD1/Tim3 co-expression, and the cDC1 depletion.

Furthermore, in standard CCI sham models, the non-specific inflammatory response is transient and does not produce the stable immunosuppressive niche characterized by TREM2^{+} myeloid cell accumulation and T cell exhaustion described here. The time course of our observations (day 10 post-resection), combined with the biological specificity of the changes (TREM2 upregulation, checkpoint co-expression on TILs, spatial localization relative to residual tumor) and the convergence between our murine findings and the human organotypic slice data, which includes a cavity creation on tumor-bearing tissue, strongly supports that the changes we describe are driven primarily by the interaction between surgical injury and residual tumor rather than by craniectomy *per se*.

Have the authors characterized the functional or exhaustion states of the CAR T cells when administered in a neoadjuvant versus adjuvant setting? Analyzing the T-cell state would provide essential mechanistic insights alongside the observed changes in bystander immune cell populations.

We thank the reviewer for this suggestion. We would like to point out that this analysis was in fact performed and is presented in detail in **Fig. 7d-g**. Specifically, using a congenic CD45.1^{+} tracking system, we characterized the phenotype and exhaustion states of GD2 CAR T cells administered in the

neoadjuvant versus adjuvant setting, three days after resection. UMAP visualization and subset quantification revealed that T_{eff} $CD8^+$ cells were significantly reduced in the adjuvant compared to the neoadjuvant group (**Fig. 7d,f**). Furthermore, exhaustion scoring showed that both T_{eff} $CD8^+$ and T_{eff} $CD4^+$ CAR T cells exhibited significantly higher exhaustion scores in the adjuvant setting compared to the neoadjuvant condition (**Fig. 7g**). Of note, CAR T cell persistence was comparable between the two arms (**Fig. 7e**), indicating that the differences are driven by functional state rather than differential elimination or engraftment. In parallel, endogenous $CD45.2^+$ T cells also showed higher exhaustion in the adjuvant group (**Fig. 7l**), suggesting that the post-resection TME broadly impairs T cell fitness regardless of whether cells are exogenously delivered or endogenous. These data collectively support a timing-dependent model in which neoadjuvant delivery positions CAR T cells in a less immunosuppressive, antigen-presenting–competent niche, preserving their effector fitness.

In Figure 3b, the Kaplan-Meier curves cross each other. Are the authors certain that the reported differences remain statistically significant despite this crossing? Please reassess and double-check

All the raw data are provided in the excel files "Raw data". For more transparency, we report here the table corresponding to Fig.3b and the statistical analysis corresponding to these results.

Condition	Mice	Day of death	Untransduced	TREM2 CAR
**UTD**	131	26	1	
	132	27	1	
	133	27	1	
	134	29	1	
	135	29	1	
	136	26	1	
	137	28	1	
	138	28	1	
	141	28	1	
	142	26	1	
**TREM2 CAR**	139	28		1
	140	28		1
	143	28		1
	144	28		1
	145	18		1
	146	33		1
	147	28		1
	148	32		1
	149	32		1
	150	32		1

Log-rank (Mantel-Cox) test	
Chi square	4,813
df	1
P value	0,0282
P value summary	*
Are the survival curves sig different?	Yes

Following comments from reviewer 1, the updated **Fig. 3b** now presents combined Kaplan–Meier curves pooled from both independent experiments (n=17 mice per group; log-rank test).

Reviewer #4 (Remarks to the Author):

In this manuscript Pedard et al. address the clinically relevant question of how surgical resection reshapes the GBM tumor microenvironment (TME) and thereby affects CAR T cell efficacy. Using two syngeneic mouse GBM models, the authors have comprehensively explored the effect of surgical resection on myeloid cells, specifically TREM2+ TAMs, and evaluated the efficacy of TREM2+ CAR T cells combined with GD2 CAR T cells. This study represents a tremendous amount of work and employs quite technically challenging mouse models to understand the impact of surgical resection on the TME. Overall, the data are well presented and support the author's conclusion. Further, the finding that neoadjuvant CAR T cell therapy is superior to adjuvant CAR-T treatment could be important for the design of future clinical trials.

The manuscript would be further strengthened by addressing the following points:

1. The study identified interesting associations between surgical resection, TREM2+ myeloid cells, and T cell exhaustion marker expression, but the mechanism is poorly defined. Do the authors propose that TREM2+ myeloid cells drive T cell exhaustion or that both occur due to a perioperative inflammatory response? Additional studies to support this association would strengthen the paper. Alternatively, authors should temper statements that may imply a direct causal link. For example, the abstract states "marked upregulation of TREM2 in myeloid cells and the subsequent emergence of T cell dysfunction" seems to imply that TREM2+ macrophages contribute to dysfunction.

We thank the reviewer for this important mechanistic point. We agree that our initial data established a temporal association ("TREM2^{+} myeloid cell expansion followed by T cell exhaustion-like phenotypes") but did not demonstrate a direct causal link. To address this, we have performed a new experiment in which SB28 tumor-bearing mice underwent surgical resection on day 10 and received intracavitary injection of either UTD or TREM2 CAR T cells. Tumors were analyzed by flow cytometry three days post-resection (new **Supplementary Fig. 11c,d**).

TREM2 CAR T cell treatment resulted in a significant increase in the frequency of CD3^{+} T cell (as % of CD45^{+} cells, **Supplementary Fig. 11c**) and a reduction in exhaustion-like scores specifically in T_{eff} CD8^{+} cells, compared to UTD T cell-treated controls (**Supplementary Fig. 11d**). These data provide direct evidence that depletion of TREM2^{+} myeloid cells is sufficient to alleviate T cell exhaustion-like phenotypes in the post-resection TME, supporting a causal relationship between TREM2^{+} myeloid cells and T cell dysfunction rather than merely a shared response to perioperative inflammation.

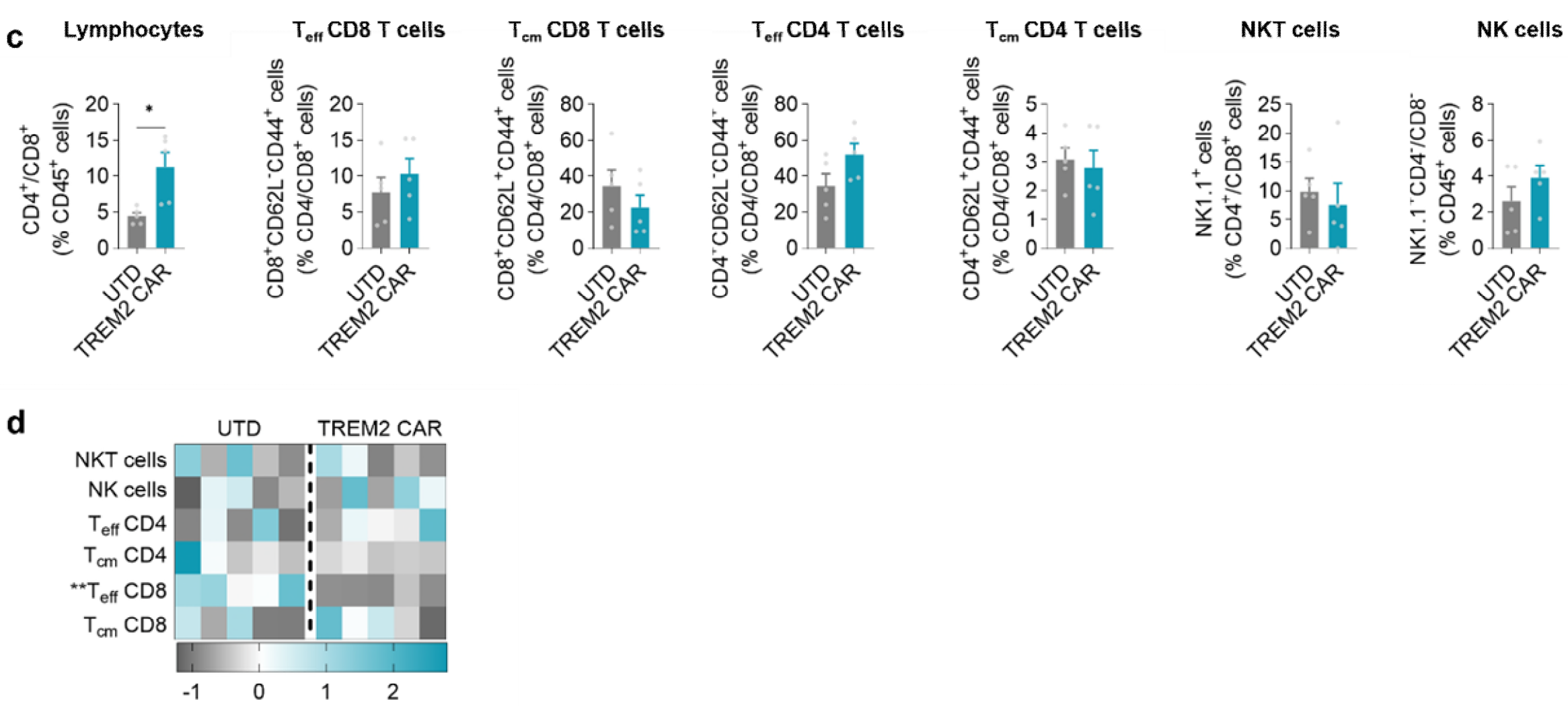

Supplementary Fig. 11c,d *(**c**) Frequency of CD3^{+} T cells (as % of CD45^{+} cells) in resected SB28 tumors treated with UTD or TREM2 CAR T cells three days post-resection (n=5 mice per group, unpaired two-tailed t-test). (**d**) Exhaustion-like scores of*

*Teff $CD8^+$ T cells in resected SB28 tumors treated with UTD or TREM2 CAR T cells three days post-resection (n=5 mice per group, unpaired two-tailed t-test). Data are shown as mean ± SEM. *p < 0.05, **p < 0.01, ***p<0.001. "*

In addition, we have tempered the language throughout the manuscript to avoid implying a direct causal link where only association is demonstrated. The abstract has been revised from "*marked upregulation of TREM2 in myeloid cells and the subsequent emergence of T-cell dysfunction*" to "*marked upregulation of TREM2 in myeloid cells, followed by the emergence of T-cell exhaustion-like phenotypes.*"

The following changes have been made in the revised manuscript:

(**i**) **Supplementary Fig. 11** has been updated with new panels showing $CD3^+$ T cell frequency and T_{eff} $CD8^+$ exhaustion-like scores in UTD versus TREM2 CAR T cell-treated mice three days post-resection (n=5 mice per group, unpaired two-tailed t-test).

(**ii**) In the Results (**lines 240-244**), after the description of TREM2 CAR T cell effects on the myeloid cell compartment, the following sentence has been added: "*In addition to myeloid cell remodeling, TREM2 CAR-T cell treatment increased $CD3^+$ T cell infiltration and reduced exhaustion-like phenotypes in T_{eff} $CD8^+$ cells compared to UTD T cell-treated controls three days post-resection (**Supplementary Fig. 11c,d**), providing evidence that $TREM2^+$ myeloid cells contribute to T cell dysfunction in the post-resection TME.*"

(**iii**) In the Discussion (**lines 540-544**), the following sentence has been added: " *Critically, early depletion of $TREM2^+$ myeloid cells by TREM2 CAR-T cells was sufficient to increase $CD3^+$ T cell infiltration and reduce exhaustion-like phenotypes in $CD8^+$ Teff cells three days post-resection, providing direct evidence that $TREM2^+$ myeloid cells actively contribute to post-surgical T cell dysfunction rather than both effects arising independently from perioperative inflammation.*"

(**iv**) The legend of **Supplementary Fig. 11** has been updated to include the new panels: ***(c)** Frequency of $CD3^+$ T cells (as % of $CD45^+$ cells) in resected SB28 tumors treated with UTD or TREM2 CAR-T cells three days post-resection (n=5 mice per group, unpaired two-tailed t-test). **(d)** Exhaustion-like scores of Teff $CD8^+$ T cells in resected SB28 tumors treated with UTD or TREM2 CAR-T cells three days post-resection (n=5 mice per group, unpaired two-tailed t-test). Data are shown as mean ± SEM. *p<0.05, **p<0.01, ***p<0.001. "*

2. For Figure 1: It is somewhat surprising that 'resting microglia' represent the population most increased following tumor resection. Please comment on this observation. Also, in Fig 1d, please clarify which specific myeloid population corresponds to the highlighted TREM2+ population, as Supp Fig 4 indicates that TREM2 expression is increased across multiple myeloid subsets. Also, the title for Figure 1 refers to "delayed myeloid remodeling," yet the data show changes as early as 24 hrs post resection.

We thank the reviewer for these three thoughtful comments, which we address sequentially below.

(**i**) **On the increase in $CD206^-MHCII^-$ ("resting") microglia following resection.** We acknowledge that this observation may appear counterintuitive and have addressed it through several complementary modifications. First, the term "resting microglia" reflects a strictly phenotypic definition based on $CD45^{int}CD11b^+CD206^-MHCII^-$ marker expression and does not imply functional quiescence. To avoid this misinterpretation, we have replaced "resting microglia" with "$CD206^-MHCII^-$ microglia" throughout the manuscript. Second, the observed increase likely reflects several non-mutually exclusive mechanisms now discussed in the revised manuscript: (**a**) surgical cytoreduction removes a large fraction of TAM-rich tumor mass, potentially enriching for brain-resident microglia in the peri-cavitary tissue sampled by flow cytometry; (**b**) CNS myeloid cell depletion is known to induce microglia proliferation and local repopulation in response to CNS injury (Elmore et al., Neuron, 2014, PMID: 24742461), which may initially manifest as a $CD206^-MHCII^-$ phenotype before subsequent polarization. Importantly, this increase occurs in parallel with broad TREM2 upregulation across

multiple myeloid subsets, indicating that this population is not immunologically inert but participates in the TREM2-associated remodeling program induced by surgery.

(ii) On the identity of the highlighted TREM2$^+$ population in Fig. 1d. We thank the reviewer for noting this ambiguity. As correctly observed, TREM2 upregulation is not restricted to a single myeloid subset but occurs broadly across the myeloid compartment (**shown in Supplementary Fig. 5**). We have revised the legend of **Fig. 1d** to explicitly state that the TREM2$^+$ cells highlighted in the volcano plot represent the aggregate CD45$^+$CD11b$^+$TREM2$^+$ myeloid compartment as follows (**Lines 1089-1092**): "*(**d**) Volcano plot showing differential abundance of myeloid cell populations and TREM2$^+$ cells among CD11b$^+$ myeloid cells between unresected and resected SB28 tumors; dot size reflects mean population frequency from one experiment (n=6-7 mice per group).*"

(iii) On the figure title "delayed myeloid remodeling". We agree with the reviewer that this terminology is misleading given that significant changes are detectable as early as 24h post-resection. The title of **Fig. 1** (**Lines 1080-1081**) has been revised to: "*Tumor resection induces a dynamic and TREM2-driven remodeling of the myeloid cell compartment in murine GBM.*"

3. The claim that resection induces T cell exhaustion/dysfunction seems overstated. The study relies mainly on PD1/Tim3/Lag3 expression by flow cytometry, which is insufficient to define exhaustion. The authors should either soften the language throughout (e.g. exhaustion-like phenotype etc) and/or provide additional supporting data (e.g. transcriptional profiling showing exhaustion signatures and/or functional readouts).

We thank the reviewer for this valid point. We agree that the co-expression of PD-1, Tim-3, and Lag-3 by flow cytometry, while consistent with an exhaustion phenotype, is not sufficient to definitively establish bona fide T cell exhaustion, which would require additional evidence such as transcriptomic profiling of canonical exhaustion programs (e.g., TOX, NR4A, TCF1) and/or functional assays demonstrating impaired cytokine production upon restimulation. We have therefore revised the manuscript throughout to replace "exhaustion" with "exhaustion-like phenotype" or "exhaustion-associated markers" when referring to flow cytometry-based observations and reserve the term "exhaustion" for contexts where it is used in its broader, established sense. All modified instances have been highlighted in yellow in the revised manuscript to facilitate the reviewer's assessment. This change is reflected in the Results, Discussion, and all figure legends where PD-1/Tim3/Lag3-based scoring is described.

4. The human GBM organotypic resection model is quite novel, and it is notable that this ex vivo injury also results in increased TREM2+ myeloid cells and an exhaustion-like T cell phenotype. Can the authors comment on the mechanism underlying these changes in this slice model? Specifically, the data seem more consistent with local induction/upregulation of TREM2 and exhaustion markers versus recruitment of cells. Authors should comment on this, and if possible, provide supporting data regarding induction versus migration. Additionally, please also clarify whether the organotypic replicates are technical replicates (GBM slices from the same patient) or biological replicates (independent patients), and provide basic clinical annotations for the GBM samples, i.e. newly diagnosed versus recurrent, IDH-status or other available features.

We thank the reviewer for these thoughtful observations about our human organotypic resection model.

Mechanisms about local induction *versus* cell migration. We agree with the reviewer's interpretation. The avascular nature of the organotypic slice model precludes any recruitment of circulating monocytes, neutrophils, or T cells from the bloodstream. Consequently, the observed increase in TREM2$^+$/CD68$^+$ myeloid cells along the cavity edge and the emergence of exhaustion-like T cell phenotypes at 72 h must reflect local reprogramming of tissue-resident populations rather than *de novo* cell recruitment. Specifically, for the myeloid cell compartment, the increase in TREM2$^+$ area adjacent to the injury margin most plausibly reflects: (i) transcriptional upregulation of TREM2 in resident microglia and pre-

existing TAMs in response to damage-associated molecular patterns (DAMPs), local hypoxia, and TGFβ released upon tissue injury; and/or (ii) spatial redistribution, i.e. directed migration within the slice, of TREM2^{+} cells already present in the tissue toward the injury margin, as suggested by their focal accumulation at the cavity edge rather than a global increase across the unresected region. The latter is consistent with the spatial repositioning of TREM2^{+} cells toward the tumor margin we observed *in vivo* in SB28 tumors at D1 and D7 post-resection (**Fig. 1h**). For the lymphoid compartment, the delayed kinetics of exhaustion (no change at 24 h, significant shift at 72 h) and the selective involvement of effector and effector memory subsets, populations already present within the tumor tissue, are most consistent with progressive checkpoint upregulation in pre-existing TILs rather than any recruitment event. This interpretation is further supported by the fact that T_{naive} cells, which would be the most likely to infiltrate from a peripheral source, were not enriched in the exhausted fraction. To more directly address induction *versus* migration, we note that the spatial distribution of TREM2^{+}/CD68^{+} cells in our immunofluorescence data shows their preferential accumulation at the cavity margin rather than a diffuse increase across the slice, arguing against a simple proportional expansion and in favor of injury-directed redistribution accompanied by local TREM2 upregulation. A formal distinction between upregulation and redistribution would require live imaging or TREM2 mRNA quantification (e.g. by RNAscope) at the single-cell level, which we acknowledge as a limitation and an avenue for future investigation.

Nature of the replicates. The organotypic experiments were performed on slices derived from independent patients (n=3 patients), with 2 to 3 technical replicates (slices) per patient used to generate paired unresected/resected comparisons. Each patient contributed one paired data point to the statistical analysis.

Clinical annotations. Basic clinical features of the samples used in the organotypic experiments are provided in the reporting summary. One sample was a primary IDHmut grade IV astrocytoma, one a recurrent IDHwt grade IV GBM and the third a primary IDHwt grade IV GBM. We acknowledge the heterogeneity of these samples but are limited by sample availability, as samples cannot be frozen before use.

5. There are general statistical concerns as some of the statistical comparisons seem questionable. Please verify stats for Fig. 3b and 5b. Log-rank test is indicated in figure legend, but please provide more details.

We thank the reviewer for raising this point. We address each figure separately.

Fig. 3b (UTD *versus* TREM2 CAR T cells, 2 groups, n=17 per group): Survival curves were compared using the log-rank (Mantel-Cox) test, which is the appropriate method for two-group survival comparisons. The values are provided in the Raw Data Excel file (sheet "Fig. 3b"). We have now repeated this experiment (first experiment shown in the initial manuscript, n=10 mice per group, additional experiment incorporated in the reviewed manuscript, n=7 mice per group) and data shown represents the pool of the two experiments.

Fig. 5b (4 groups: UTD/UTD+isotype, TREM2/GD2 CAR+isotype, UTD/UTD+aPD1/aTim3, TREM2/GD2 CAR+aPD1/aTim3, n=10 per group): Pre-specified pairwise log-rank (Mantel-Cox) tests were performed between biologically relevant group pairs, consistent with standard practice for preclinical survival studies. The comparisons of interest and their exact p-values are detailed in the Raw Data Excel file (sheet "Fig. 5b")

For clarity, the Methods section has been updated as follows (**Lines 816-819**):

"*Survival data were analyzed using the Kaplan-Meier method. For two-group comparisons, the log-rank (Mantel-Cox) test was used. For experiments with more than two groups, pre-specified pairwise*

log-rank tests were performed between biologically relevant comparisons and exact p-values are reported in the Raw Data Excel file."

All raw survival data and statistical outputs are provided in the Raw Data Excel file.

6. While the methods provide details for the TREM2 CAR, the GD2 CAR design is not described with similar detail. Please include a schematic and/or a table summarizing both CAR constructs, including scFv clone, spacer/hinge, transmembrane, costim, and any tags or other features.

We thank the reviewer for this suggestion. Both the TREM2 and GD2 CAR constructs share an identical backbone architecture and differ only in their scFv domain. Both are second-generation CARs cloned into the pMSGV retroviral vector, incorporating a murine CD28 hinge, transmembrane, and co-stimulatory domain, fused to the murine CD3ζ signaling domain. The TREM2 CAR uses the 37012 scFv derived from patent application WO2020123664A1, while the GD2 CAR uses the 14g2a scFv. CAR surface expression was detected using anti-F(ab')$_2$ staining in both cases. We have added a supplementary table (**New Supplementary Table 1**) summarizing the design of both CAR constructs and updated the Methods section as follow (**Line 636**): "*Design of both CAR constructs are summarized in **Supplementary Table 1.***"

7. Please provide expression levels for the GD2-engineered SB28 and CT2A lines.

We thank the reviewer for this important point. GD2 expression in engineered SB28 and CT2A cell lines was validated by flow cytometry prior to all in vivo experiments. Representative histograms are now provided as a new Supplementary Figure (**Supplementary Fig. 21**), and the corresponding description has been added to the Methods section under *Cell lines*. GD2 expression was assessed using an anti-GD2 antibody with appropriate isotype control.

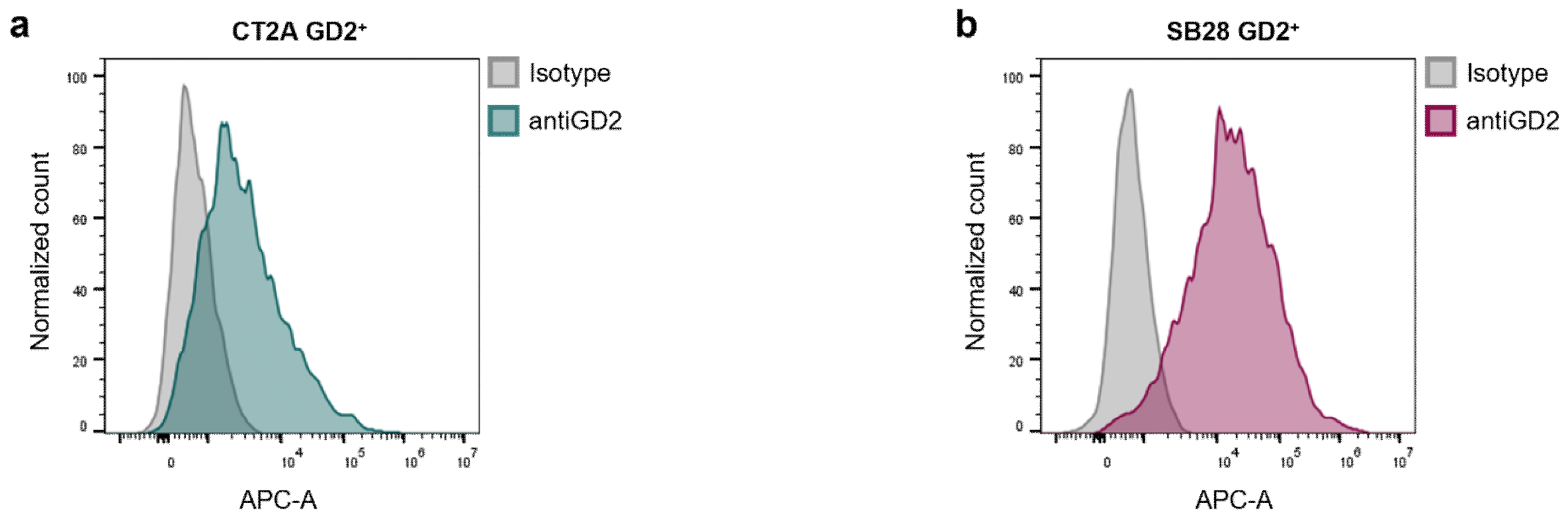

Supplementary figure 21. *GD2 surface expression on murine GBM cell lines. Representative flow cytometry histograms showing GD2 surface expression on CT2A GD2$^+$ (**a**) and SB28 GD2$^+$ (**b**) cell lines cultured in vitro. Cells were stained with an anti-GD2 antibody (colored) or isotype control (grey). The percentage of GD2$^+$ cells within the live CD45$^-$ gate is indicated.*

8. The gating strategy for the CD45.1$^+$ population in Figure 4j appears to include some CD45.1$^-$CD45.2$^-$ cells, which may represent GFP$^+$ tumor cells. This raises the possibility that the CFSE-high population in the UTD group may contain residual tumor cells. A more stringent gating strategy should be used to exclude tumor cells.

We thank the reviewer for this important observation. We agree that stringent exclusion of tumor cells is essential for accurate interpretation of adoptively transferred CD45.1$^+$ T-cell populations.

In response, we have reanalyzed the dataset using a more stringent gating strategy. Specifically, we refined the gating hierarchy to:

- Exclude debris and doublets,
- Strictly exclude CD45.1$^-$CD45.2$^-$ events,

The revised gating strategy is provided in **Fig. R6**

We then repeated the full quantification of the CFSE^{low} population using this stricter gating approach. Importantly, the updated analysis yielded results comparable to those originally reported, and the statistical conclusions remain unchanged (**new Fig. 4k**).

We have clarified the gating strategy in the Methods section and now explicitly reference the revised gating in the figure legend to ensure transparency.

We thank the reviewer for prompting this clarification, which strengthens the rigor of the analysis.

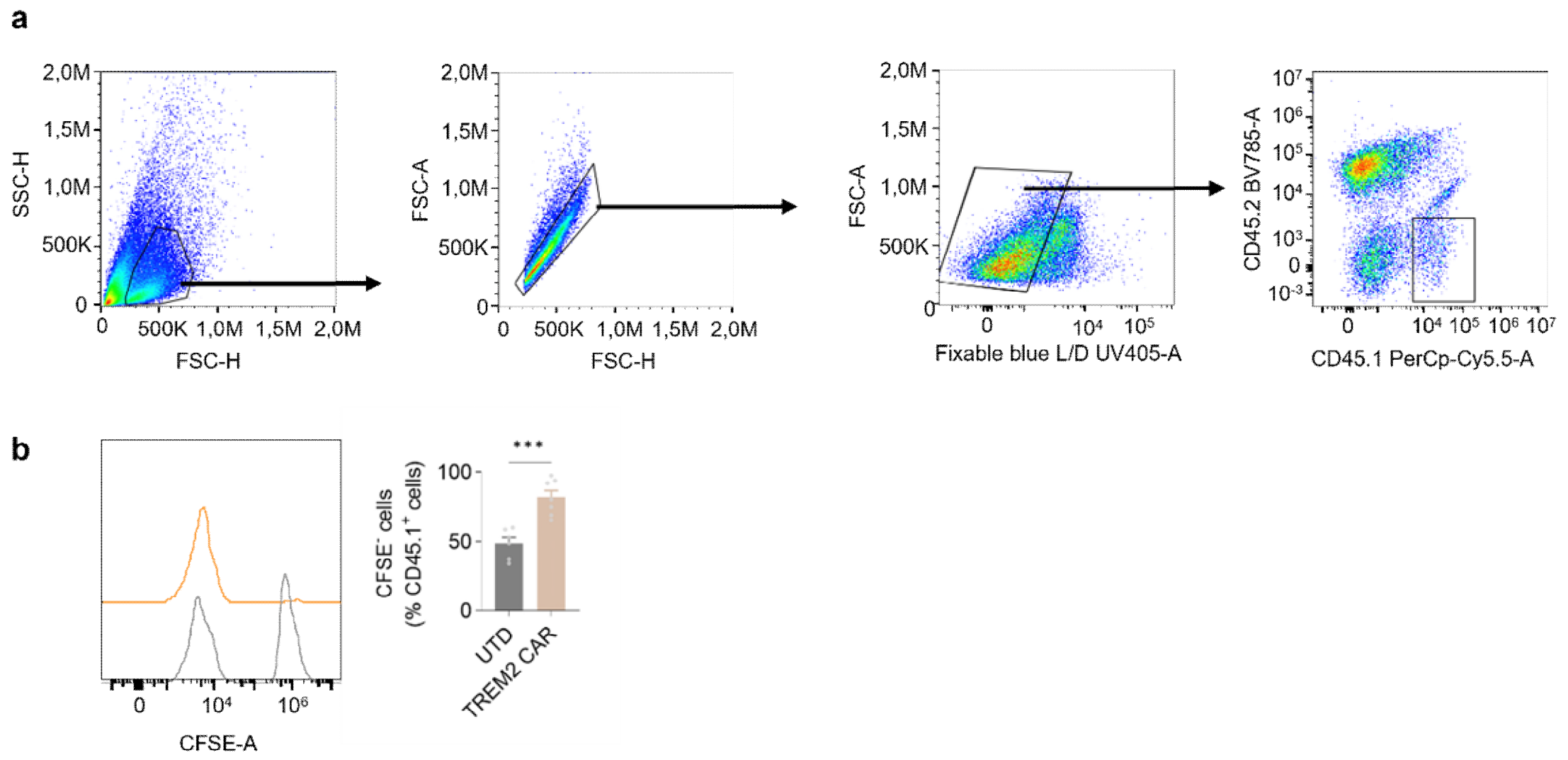

Figure R6: Refined gating strategy for CFSE quantification in adoptively transferred CD45.1^{+} T cells. (***a***) Stringent gating hierarchy used to quantify proliferating (CFSE^{low}) CD45.1^{+} GD2 CAR T cells recovered from the resection cavity, co-administered with UTD or TREM2 CAR T cells. (***b***) Representative CFSE histograms of CD45.1^{+} cells (left) and frequency of CFSE^{low} cells among CD45.1^{+} cells in UTD versus TREM2 CAR T cell co-treated groups (right). This refined analysis corresponds to the updated Fig. 4k. Data are shown as mean ± SEM (***$p<0.001$).

9. Fig. S12 shows that the addition of TREM2+ CAR, compared to UTD T cells, increases IFNy production by GD2 CAR T cells. This observation is consistent with the findings by Sun et al. (PMID: 37172094), which reported that TREM2- knockout microglia exhibit enhanced responsiveness to IFNy. However, Fig S12b also shows that SB28-CM-conditioned treated BMDM suppresses IFNy secretion. It would be helpful to clarify how the proportion of TREM2+ macrophages change following SB28-CM and after TREM2 CAR treatment. This could help determine whether changes in IFNg secretion correlate with modulation of depletion of TREM2+ macrophages.

We thank the reviewer for this insightful comment. To directly address this point, we quantified TREM2 expression at both the transcript (RT-qPCR) and protein (flow cytometry) levels in BMDMs exposed to both CT2A- and SB28-conditioned medium (CM) for 72 h and assessed phenotypic changes following co-culture with TREM2 CAR T cells.

SB28-CM significantly upregulated TREM2 mRNA and surface expression alongside a suppressive phenotypic shift (increased Arg1, CD206, PD-L1; decreased iNOS, CD11c), confirming induction of a tumor-educated macrophage state (**New Supplementary Fig. 10a,b**). Co-culture with TREM2 CAR T cells reversed this phenotype, reducing TREM2, Arg1, PD-L1, and CD206 while restoring iNOS and CD11c expression (**Supplementary Fig. 10e**). These data directly demonstrate that the SB28-CM–induced reprogramming of TREM2^{+} immunosuppressive macrophages correlates with suppression of GD2 CAR T cell IFNγ secretion, and that TREM2 CAR T cell-mediated depletion and reprogramming of this compartment is sufficient to restore GD2 CAR T cell effector function.

We now included these results in **new Supplementary Fig. 10a,b** and described in the revised Results section (**Lines 208-212**) as follows "*. SB28-CM exposure significantly increased TREM2 transcript and surface expression on BMDM, consistent with induction of a tumor-educated suppressive phenotype characterized by decreased iNOS and CD11c and increased Arg1, CD206 and PD-L1 expression (**Supplementary Fig. 10a,b**).*"

The legend of Supplementary Fig. 10 has been updated as follows "*(**a**) Heatmap of RT-qPCR gene expression (Z-score) for Arg1, IL10, CD206, CD163, TGFβ, CD86, iNOS, CXCL10, and TREM2 in BMDM cultured in control medium (DMEM), CT2A-conditioned medium, or SB28-conditioned medium for 72 h. (**b**) Flow cytometry quantification of surface protein levels (MFI) for CD11c, iNOS, Arginase-1, CD206, PDL1, and TREM2 in BMDM cultured in DMEM, CT2A-conditioned medium, or SB28-conditioned medium for 72 h (n=5-16 per group, one-way ANOVA, two independent experiments).*"

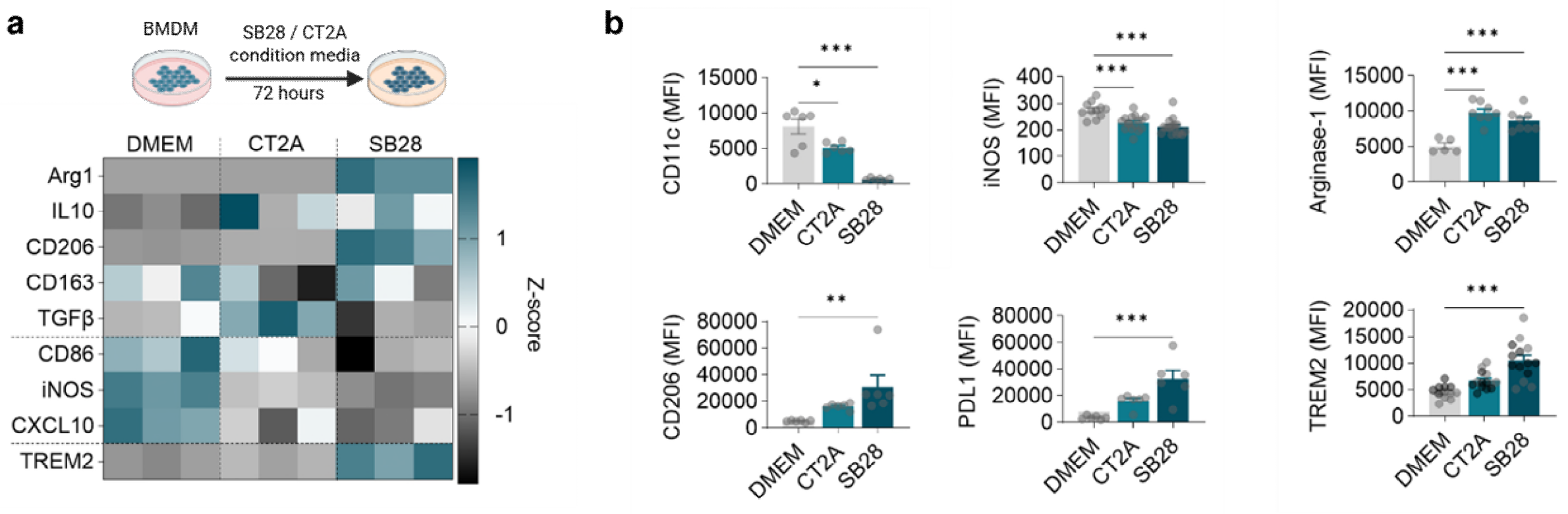

***Supplementary Figure 10a,b** (**a**) Heatmap of RT-qPCR gene expression (Z-score) for Arg1, IL10, CD206, CD163, TGFβ, CD86, iNOS, CXCL10, and TREM2 in BMDM cultured in control medium (DMEM), CT2A-conditioned medium, or SB28-conditioned medium for 72 h. (**b**) Flow cytometry quantification of surface protein levels (MFI) for CD11c, iNOS, Arginase-1, CD206, PDL1, and TREM2 in BMDMs cultured in DMEM, CT2A-conditioned medium, or SB28-conditioned medium for 72 h (n=5-16 per group, one-way ANOVA, two independent experiments).* [Editorial note: graphics in panel a Created in BioRender. Nicola, C. (2026) https://BioRender.com/1lylymz]

10. Prior studies have investigated the role of TREM2 in the TME of gliomas. As referenced in the manuscript (refs 31-33), Zheng et al and Zhong et al suggest that TREM2+ myeloid cells may promote anti-tumor immune responses, whereas Sun et al reports findings more similar to this study, showing that TREM2+ TAMs are immunosuppressive and that targeting this population may be beneficial. Interestingly, Zhong et al used GL261 and CT-2A model, Zheng et al. used GL261; Sun et al. used SB28 and this study used SB28 and CT-2A models. While GL261 has been considered as immune "hot" tumor and may not fully recapitulate the human GBM TME, Zhong et al also reported that TREM2 knockout promotes CT2A tumor growth, indicating an anti-tumor role of TREM2+ TAM. This appears to be inconsistent with the conclusions of the current study. Given the different effects observed when targeting TREM2+ myeloid cells in the neoadjuvant vs adjuvant setting and these other published results, the authors are encouraged to more fully discuss these discrepancies, and to comment on possible explanations, i.e., model specific TME context, timing, and/or methodologies.

We thank the reviewer for this important comment. We agree that the role of TREM2^{+} myeloid cells in glioma appears context-dependent in literature.

Several key factors are likely to reconcile the divergent findings.

First, **model-specific immune context** differs substantially across studies. As mentioned by the reviewer, Zheng et al. and Zhong et al. primarily used the GL261 model, which is widely regarded as more immunogenic and T cell-inflamed compared with SB28. In contrast, SB28 tumors exhibit a myeloid cell-dominant, poorly T cell-infiltrated microenvironment that more closely resembles human GBM. In a T cell–inflamed context (e.g., GL261), TREM2-dependent antigen presentation by microglia

or macrophages may transiently support anti-tumor $CD4^+$ T-cell responses. Conversely, in a poorly immunogenic, wound-healing-skewed environment such as SB28, particularly after surgical injury, $TREM2^+$ tumor-associated macrophages may preferentially adopt immunosuppressive and tissue-remodeling programs that reinforce T-cell dysfunction. We now explicitly discuss these model-dependent immune landscapes.

Second, **methodological differences** are critical. Previous studies relied largely on global or germline TREM2 knockout approaches, which affect myeloid cell differentiation and homeostasis throughout tumor development. In contrast, our strategy uses CAR T cell-mediated targeting to acutely deplete or reprogram established $TREM2^+$ populations within a defined perioperative window. Thus, our approach interrogates the functional contribution of $TREM2^+$ cells in an already formed tumor microenvironment rather than developmental effects of TREM2 deficiency.

Third, and most importantly, our data highlight **timing as a decisive variable**. We show that targeting $TREM2^+$ cells in the adjuvant (post-resection) setting enhances GD2 CAR T-cell activity, whereas neoadjuvant targeting unexpectedly attenuates GD2 CAR efficacy. This observation supports a temporally dynamic model in which $TREM2^+$ myeloid cells may initially contribute to antigen presentation or $CD4^+$ T-cell support prior to resection, but expand into a predominantly suppressive, wound-healing-associated state following surgical injury. This time-dependent shift may explain why TREM2 targeting yields distinct outcomes depending on the therapeutic window.

Finally, we have tempered statements implying a universal immunosuppressive role for $TREM2^+$ cells. Rather than assigning a fixed pro- or anti-tumoral function, we now propose a **context- and time-dependent model** in which $TREM2^+$ myeloid cells exert distinct roles depending on tumor immune state, model system, and perioperative conditions.

We believe these clarifications strengthen the manuscript by positioning our findings within the broader and evolving understanding of TREM2 biology in glioma. Consequently, we have substantially expanded the Discussion to address these discrepancies (**Discussion, Lines 493-505**) as follows: "*Notably, recent studies have reported context-dependent roles of TREM2 in glioma, ranging from immunosuppressive functions in myeloid cell-dominant SB28 tumors*[33] *to MHCII-associated support of* $CD4^+$ *T cell responses in the GL261 and CT2A models*[34]*, likely reflecting differences in tumor immunogenicity and ontogeny of myeloid lineages. An important methodological distinction is that prior studies relied on germline or conditional Trem2 deletion, which alters myeloid cell ecology throughout tumor development, whereas the CAR-T cell-based approach used here acutely targets established* $TREM2^+$ *populations within a defined perioperative window. Moreover, our data reveal that timing is a decisive variable: TREM2 targeting in the adjuvant setting enhanced GD2 CAR-T cell activity, whereas neoadjuvant TREM2 depletion unexpectedly attenuated it, consistent with a temporally dynamic model in which* $TREM2^+$ *cells may shift from antigen-presenting to predominantly immunosuppressive states following surgical injury. Compartment-specific targeting of* $TREM2^+$ *populations thus represents an important direction for future investigation.*"

11. In the discussion, the authors claim that resection induces "myeloid-dominant immunosuppressive programs centered on TREM2+ cells." However, the manuscript does not exhaustively evaluate myeloid functional states and marker expression or establish that TREM2 cells are the primary drivers of immunosuppression. These statements should be qualified throughout the manuscript.

We thank the reviewer for this valid point. We agree that our data demonstrate a consistent and significant upregulation of $TREM2^+$ myeloid cell populations following resection, and that TREM2 CAR T cell-mediated depletion of this compartment alleviates T cell exhaustion-like phenotypes and enhances CAR T cell activity, providing functional evidence linking $TREM2^+$ cells to immune suppression in this context. However, we acknowledge that our study does not exhaustively characterize

myeloid cell functional states at the single-cell level, nor does it formally establish TREM2$^+$ cells as the sole or primary drivers of immunosuppression in the post-resection TME.

We have therefore revised the relevant statements throughout the manuscript to avoid causal overstatement. Specifically:

(i) **Results, lines 196-198:** *"TREM2 upregulation is a consistent feature across models and time points, supporting its prioritization as a target in the immediate post-resection window"* has been revised to *"TREM2 upregulation is a consistent and functionally relevant feature across models and time points, supporting its prioritization as a candidate target in the immediate post-resection window."*

(ii) **Results, lines 275-277:** *"Together, these findings demonstrate that targeting immunosuppressive myeloid cells via TREM2 CAR T cells not only improves the functional state of the TME but also enhances the proliferation, persistence, and differentiation of co-administered GBM-directed CAR T cells"* has been revised to *"Together, these findings suggest that targeting TREM2$^+$ myeloid cells via TREM2 CAR-T cells is associated with improved functional state of the TME and enhanced proliferation, persistence, and differentiation of co-administered GBM-directed CAR-T cells."*

(iii) **Discussion, lines 394-396:** *"resection induces an early, myeloid-dominated immunosuppressive program centered on TREM2$^+$ cells"* has been revised to *"resection induces an early, myeloid cell-dominated immune remodeling program in which TREM2$^+$ cell expansion is a prominent and functionally relevant feature."*

(iv) **Discussion, lines 451-452:** *"establish TREM2$^+$ myeloid cells as a rational target for perioperative immunomodulatory strategies in GBM"* has been revised to *"support TREM2$^+$ myeloid cells as a rational candidate target for perioperative immunomodulatory strategies in GBM."*

Minor comments:

1. It was a bit challenging to follow how cell populations were defined by flow cytometry and required going to the gating strategy in the supp figures. Please add a Table summarizing marker panels used to define the myeloid and lymphocyte populations.

To facilitate interpretation of the flow cytometry data, we have added two new supplementary tables summarizing the complete gating strategy and marker combinations used to define each immune population, with the updated nomenclature. **Supplementary Table 3** covers myeloid cell populations and **Supplementary Table 4** covers lymphoid populations. The Methods section (Flow cytometry) has been updated as follows:

Methods, lines 759-768, section Flow cytometry :

"*Briefly, myeloid cell populations were identified within the CD11b$^+$ CD45$^+$ gate as follows: microglia (CD45^{low} F4/80$^+$ CD11b$^+$), macrophages (CD45^{high} F4/80$^+$ CD11b$^+$), and Ly6C$^+$ monocytes (CD45^{high} F4/80$^-$ CD11b$^+$ Ly6C$^+$ Ly6G$^-$) were each further subdivided into CD206$^-$MHCII$^-$, CD206$^+$MHCII$^{-/+}$, and CD206$^-$MHCII$^+$ subsets. Neutrophils were defined as CD45^{high} CD11b$^+$ Ly6G$^+$ Ly6C$^+$. Conventional dendritic cells were identified as cDC1 (CD11c$^+$ MHCII$^+$ XCR1$^+$) and cDC2 (CD11c$^+$ MHCII$^+$ XCR1$^-$). Lymphoid populations were gated as follows: NKT cells (CD4$^+$/CD8$^+$ NK1.1$^+$), CD4$^+$ T cells (CD8$^-$ CD4$^+$ NK1.1$^-$) and CD8$^+$ T cells (CD4$^-$ CD8$^+$ NK1.1$^-$), each subdivided into T$_{naive}$ (CD44$^-$CD62L$^+$), T$_{cm}$ (CD44$^+$CD62L$^+$) and T$_{eff}$ (CD44$^+$CD62L$^-$) subsets; NK cells (CD4$^-$ CD8$^-$ NK1.1$^+$). A complete description of all marker panels and gating hierarchy is provided in **Supplementary Tables 3 and 4.***"

2. Figure 1g; Unresected panels are titled as D1/7 post-resection. This seems to be a mislabeling.

We thank the reviewer for this careful observation. However, there is no mislabeling in Fig. 1g. The labels "D1 post-resection" and "D7 post-resection" refer to the experimental timepoint at which all animals, both resected and unresected, were sacrificed and analyzed, not to a surgical procedure performed in the unresected group. Unresected mice served as time-matched controls, sacrificed at the same days as their resected counterparts to allow direct paired comparison of TREM2^{+} cell distribution and density. This design is standard for IHF comparative studies and is consistent with the text (lines 135-136): "Immunohistofluorescence analysis of SB28 tumors at early (D1 post-resection) and late (D7 post-resection) time points in resected or unresected mice").

To avoid any future ambiguity, we have nonetheless clarified the figure legend (**Lines 1097-1098**) to specify that D1 and D7 refer to the time elapsed since the date of surgical resection in the resected group, and that unresected mice were sacrificed at the same timepoints as time-matched controls.

3. Figure 5b please check groups, as it seems that TREM2/GD2 CAR+isotype and UTD+PD1/Tim3 may be switched.

We thank the reviewer for this careful observation. We have carefully re-examined the raw data and the figure for Fig. 5b.

Regarding the potential group switch: Upon verification, the groups in Fig. 5b were indeed mislabeled in the original submission. The TREM2/GD2 CAR + isotype and UTD+UTD+anti-PD1/anti-Tim3 curves were switched in the figure display. The underlying raw data (provided in the Raw Data Excel file, sheet "Fig. 5b") were correct, and the statistical comparisons were performed in the correct groups. The figure has been corrected in the revised manuscript to accurately reflect the group assignments. We apologize for this error.

4. Figure legends need proofreading. Illustrations of Fig 3j, Fig 4e-4k and Fig S8c-d are missing, and the legend of Fig 4a is duplicated with that of Fig 5a.

We thank the reviewer for this careful proofreading. We have corrected all four issues in the revised manuscript:

(i) Fig. 3j: The description of panel **(j)** was missing from the legend. It has been added: *"(**j**) Flow cytometry quantification of CD11b^{+} myeloid cell subsets in TREM2 CAR T + isotype versus TREM2 CAR T + anti-Gr1 antibody-treated tumors (n=5 mice per group, unpaired two-tailed t-test)."*

(ii) Fig. 4: The legend of Fig. 4 was entirely incorrect, as it described the experimental design of Fig. 5 (including checkpoint inhibitor arms) rather than the TREM2/GD2 CAR T cell co-injection experiment. The complete legend has been replaced in the revised manuscript as follows: *"(**a**) Schematic of the experiments performed in mice bearing intracranial SB28 tumors and treated with local co-injection of two T cell populations (5×10^{5} each): untransduced (UTD)+UTD, TREM2 CAR+UTD, UTD+GD2 CAR, or TREM2 CAR+GD2 CAR-T cells into the resection cavity. (**b**) Longitudinal bioluminescence imaging (BLI) (left) and Kaplan-Meier survival analysis of resected SB28-bearing mice treated with UTD+UTD, TREM2 CAR+UTD, UTD+GD2 CAR, or TREM2 CAR+GD2 CAR-T cells into the resection cavity from one experiment (n=10 mice per group; log-rank test). mOS=median overall survival. (**c**) Uniform manifold approximation and projection (UMAP) visualization of tumor-infiltrating CD45^{+}CD11b^{+} cells following GD2 CAR+UTD vs GD2+TREM2 CAR-T cell treatment, colored by clusters. (**d**) Volcano plot showing log2 fold-change in population frequencies between groups from one experiment (n=6 mice per group; unpaired two-tailed t-test); positive values indicate enrichment in TREM2 CAR-T cell–treated mice (yellow), negative values indicate enrichment in UTD T cell-treated mice (grey). X-axis=log2 fold-change (log2FC), Y-axis=-log10 p-value, circle size proportional to mean population frequency. (**e**) Experimental design for the adoptive transfer of GD2 CAR-T cells in combination with UTD or TREM2 CAR-T cells. (**f**) UMAP plots of tumor-infiltrating CD4^{+}/CD8+ CD45.1^{+} T cells from GD2 CAR-T cell+UTD T cell-treated or GD2 CAR-T cell+TREM2 CAR-T cell-*

*treated groups, with cell-type cluster annotations. (**g**) Flow cytometry quantification of $CD4^+$/CD8+ $CD45.1^+$ T cells in tumors of UTD vs TREM2 CAR-T cell-treated mice from one experiment (n=6 mice per group, unpaired two-tailed t-test). (**h**) Frequencies of NKT, Teff CD4, Tcm CD4, Teff CD8, and Tcm CD8 cell subsets among $CD3^+CD45.1^+$ GD2 CAR-T cells co-administered with UTD or TREM2 CAR-T cells. (**i**) Heatmap of exhaustion-like scores (z-score; composite of exhaustion-associated markers, see Methods) in T cell subsets from GD2 CAR+UTD and GD2+TREM2 CAR-T cell treated mice. (**j**) Experimental design for the in vivo persistence assay in which CFSE-labeled GD2 CAR-T cells are co-administered with UTD or TREM2 CAR-T cells. (**k**) Representative flow cytometry plots (left) and quantification (right) showing proliferation of GD2 CAR-T cells in combination with UTD or TREM2 CAR-T cells (n=6-7 mice per group, unpaired two-tailed t-test). Data are shown as mean ± SEM. *p < 0.05, **p<0.01, ***p<0.001."*

(iii) Supplementary Fig. 8c–d: The descriptions of panels (**c**) and (**d**) were missing from the legend of Supplementary Fig. 8. In addition, we note that the legend title incorrectly referred to "10 days after tumor resection" whereas these data were collected 24 h post-resection (early timepoint). Both errors have been corrected. Panels (**c**) and (**d**) have been added to the legend as follows: *"(**c-d**) Frequencies of $CD4^+/CD8^+$ T cell subsets (T_{eff} CD8, T_{cm} CD8, T_{eff} CD4, T_{cm} CD4, $NK1.1^+$ cells, and NKT cells) among $CD45^+$ cells in unresected versus resected SB28 (c) and CT2A (d) tumors 24 h post-resection (n=6-7 mice per group, unpaired two-tailed t-test)."* The legend title has been corrected to read: "Supplementary Fig. 8. Quantification of lymphoid cell populations and exhaustion profiles 24 hours after tumor resection in SB28 and CT2A models."

(iv) Fig. 4a duplicated with Fig. 5a: The legend of Fig. 4(a) incorrectly described the experimental scheme of Fig. 5 (which includes checkpoint inhibitors). It has been corrected to accurately reflect the Fig. 4 design: *"(**a**) Schematic of the experiments performed in mice bearing intracranial SB28 glioblastoma and treated with local co-injection of two T-cell populations (5×10^5 each): untransduced (UTD)+UTD, TREM2 CAR+UTD, UTD+GD2 CAR, or TREM2 CAR+GD2 CAR into resection cavity."*

REVIEWER COMMENTS

Reviewer #1 (Remarks to the Author):

We thank the reviewer for this important clarification, which we fully agree with. In the GBM context, the $CD45^{high}F4/80^{+}CD11b^{+}$ subset defined as TAMs in our manuscript comprises both activated microglia, which upregulate CD45 upon tumor-induced activation and monocyte-derived macrophages, which cannot be reliably discriminated using conventional surface markers.

To address this point and ensure consistency throughout the manuscript, we have made the following revisions:

- In the Results section (**lines 96-97**), we have added an explicit definition at the first mention of TAMs: *"...($CD45^{high}F4/80^{+}CD11b^{+}$, defined as including both activated microglia and monocyte-derived macrophages; see Methods)."*
- In the Methods section (Flow cytometry, **lines 764-767**), we have added the following clarification immediately after the description of the gating strategy: *"Of note, the $CD45^{high}$ TAM gate comprises both activated microglia, which upregulate CD45 upon tumor-induced activation, and monocyte-derived macrophages, which cannot be reliably discriminated by conventional flow cytometry in the GBM context (refs 48, 80)."*

The two references cited (Bowman et al., *Cell Rep* 2016, PMID 27840052; Pombo Antunes et al., *Nat Neurosci* 2021, PMID 33782623) are established works on the limitations of conventional flow cytometry-based discrimination between activated microglia and monocyte-derived macrophages in brain tumors and were already included in our reference list.

We hope these clarifications adequately address the reviewer's concern and ensure uniform interpretation of the TAM designation throughout the manuscript.

Reviewer #2 (Remarks to the Author):

We thank the reviewer for carefully testing the code and for identifying this issue. The error at line 260 was caused by a naming inconsistency in the example data files provided in the GitHub repository: the DAPI channel file (Anonymous.nd2 (series 12) - C=0.jpg) lacked the descriptive condition suffix (Unresected 1-2.nd2) that was present in the other three channel files (C=1, C=2, C=3). As a result, the grouping step, which relied on the base filename to match channels belonging to the same imaging field, failed to identify a complete triplet and returned the stop message as designed.

To resolve this, we have renamed the DAPI example file in the GitHub repository (https://github.com/pedarmar-git/TREM2_GFP_analysis) to ensure that all four channel files of the same image share a consistent base filename: Anonymous.nd2 - Unresected 1-2.nd2 (series 12) - C=0.jpg, ... C=1.jpg, ... C=2.jpg, and ... C=3.jpg. The R script itself did not require modification and now runs successfully on the corrected example data.

We have also verified that the pipeline produces the expected output on the corrected example dataset.

We thank the reviewer again for this careful validation, which has directly improved the reproducibility and clarity of our shared code.
